# Supplementary material for: Cross-sectional centiles of blood pressure by age and sex: a four-hospital database retrospective observational analysis
Source: BMJ Open. 2020 May 5;10(5):e033618. doi: 10.1136/bmjopen-2019-033618 (PMC7223140; doi:10.1136/bmjopen-2019-033618)
Supplement: Supplementary data [file bmjopen-2019-033618supp001.pdf]

Supplementary Material

Appendix A – centiles and confidence intervals

Male systolic blood pressure centiles with 95% CI

	Age	_1st	_5th	_10th	_25th	_50th	_75th	_90th	_95th	_99th
1	20	95.12 (93.60, 96.65)	102.73 (101.90, 103.6)	106.8 (106.1, 107.5)	113.7 (113.1, 114.2)	121.6 (121.1, 122.1)	130.0 (129.4, 130.6)	138.3 (137.5, 139.1)	143.7 (142.7, 144.7)	154.9 (152.9, 157.0)
2	22	95.30 (94.02, 96.57)	102.91 (102.22, 103.6)	107.0 (106.4, 107.6)	113.9 (113.5, 114.4)	122.0 (121.6, 122.4)	130.6 (130.2, 131.1)	139.2 (138.6, 139.8)	144.8 (144.1, 145.5)	156.6 (154.9, 158.3)
3	24	95.46 (94.34, 96.58)	103.08 (102.48, 103.7)	107.2 (106.7, 107.7)	114.2 (113.8, 114.6)	122.4 (122.0, 122.7)	131.2 (130.9, 131.6)	140.1 (139.6, 140.6)	145.9 (145.2, 146.6)	158.3 (156.6, 160.0)
4	26	95.60 (94.56, 96.65)	103.22 (102.67, 103.8)	107.3 (106.9, 107.8)	114.4 (114.1, 114.8)	122.7 (122.5, 123.0)	131.8 (131.5, 132.2)	140.9 (140.4, 141.5)	147.0 (146.2, 147.8)	159.9 (158.0, 161.9)
5	28	95.71 (94.73, 96.69)	103.33 (102.78, 103.9)	107.5 (107.0, 107.9)	114.6 (114.2, 115.0)	123.1 (122.7, 123.5)	132.4 (131.9, 132.9)	141.8 (141.1, 142.5)	148.0 (147.1, 149.0)	161.6 (159.5, 163.6)
6	30	95.78 (94.85, 96.71)	103.42 (102.83, 104.0)	107.6 (107.1, 108.1)	114.8 (114.3, 115.3)	123.4 (122.9, 123.9)	132.9 (132.3, 133.6)	142.6 (141.8, 143.5)	149.1 (148.0, 150.2)	163.2 (161.1, 165.3)
7	32	95.80 (94.90, 96.69)	103.48 (102.88, 104.1)	107.7 (107.1, 108.2)	115.0 (114.5, 115.5)	123.8 (123.2, 124.3)	133.5 (132.8, 134.2)	143.5 (142.6, 144.4)	150.2 (149.0, 151.4)	164.9 (162.8, 167.1)
8	34	95.78 (94.91, 96.65)	103.51 (102.94, 104.1)	107.8 (107.2, 108.3)	115.2 (114.7, 115.7)	124.1 (123.6, 124.6)	134.1 (133.4, 134.8)	144.4 (143.5, 145.3)	151.4 (150.2, 152.5)	166.7 (164.5, 168.9)
9	36	95.72 (94.85, 96.58)	103.52 (102.98, 104.1)	107.8 (107.4, 108.3)	115.4 (114.9, 115.8)	124.5 (124.0, 124.9)	134.7 (134.1, 135.3)	145.3 (144.4, 146.2)	152.5 (151.3, 153.7)	168.6 (166.4, 170.7)
10	38	95.62 (94.74, 96.49)	103.50 (102.97, 104.0)	107.9 (107.4, 108.3)	115.5 (115.1, 115.9)	124.8 (124.4, 125.2)	135.3 (134.7, 135.8)	146.2 (145.3, 147.1)	153.7 (152.5, 154.9)	170.5 (168.2, 172.7)
11	40	95.47 (94.57, 96.36)	103.46 (102.92, 104.0)	107.9 (107.4, 108.3)	115.7 (115.3, 116.0)	125.1 (124.7, 125.5)	135.8 (135.3, 136.4)	147.1 (146.2, 148.0)	154.9 (153.7, 156.0)	172.4 (170.1, 174.6)
12	42	95.27 (94.33, 96.20)	103.38 (102.84, 103.9)	107.9 (107.4, 108.3)	115.8 (115.4, 116.2)	125.4 (125.1, 125.8)	136.4 (135.9, 137.0)	148.0 (147.2, 148.8)	156.0 (154.9, 157.1)	174.2 (172.0, 176.4)
13	44	95.03 (93.99, 96.07)	103.28 (102.72, 103.8)	107.9 (107.4, 108.3)	115.9 (115.6, 116.3)	125.8 (125.4, 126.1)	137.0 (136.5, 137.5)	148.9 (148.1, 149.6)	157.1 (156.0, 158.2)	175.9 (173.5, 178.2)

Wong D, *et al. BMJ Open* 2020; 10:e033618. doi: 10.1136/bmjopen-2019-033618

	Age	_1st	_5th	_10th	_25th	_50th	_75th	_90th	_95th	_99th
14	46	94.79 (93.62, 95.96)	103.18 (102.59, 103.8)	107.8 (107.4, 108.3)	116.0 (115.7, 116.4)	126.1 (125.7, 126.4)	137.5 (137.0, 138.0)	149.7 (148.9, 150.4)	158.1 (157.0, 159.1)	177.3 (174.7, 179.8)
15	48	94.56 (93.36, 95.76)	103.07 (102.48, 103.7)	107.8 (107.4, 108.2)	116.2 (115.8, 116.5)	126.4 (126.1, 126.7)	138.1 (137.6, 138.5)	150.4 (149.7, 151.1)	158.9 (157.9, 160.0)	178.4 (175.7, 181.0)
16	50	94.35 (93.26, 95.44)	102.97 (102.42, 103.5)	107.8 (107.4, 108.2)	116.3 (116.0, 116.6)	126.7 (126.4, 127.0)	138.6 (138.1, 139.0)	151.1 (150.4, 151.8)	159.7 (158.7, 160.7)	179.1 (176.7, 181.6)
17	52	94.16 (93.22, 95.10)	102.87 (102.37, 103.4)	107.8 (107.4, 108.1)	116.4 (116.1, 116.7)	127.0 (126.7, 127.3)	139.0 (138.6, 139.5)	151.7 (151.0, 152.4)	160.4 (159.4, 161.3)	179.7 (177.6, 181.9)
18	54	93.98 (93.14, 94.82)	102.78 (102.29, 103.3)	107.7 (107.3, 108.1)	116.5 (116.2, 116.8)	127.3 (127.0, 127.6)	139.5 (139.1, 139.9)	152.3 (151.6, 152.9)	161.0 (160.1, 161.9)	180.1 (178.2, 182.1)
19	56	93.79 (92.98, 94.60)	102.68 (102.19, 103.2)	107.7 (107.3, 108.1)	116.6 (116.3, 116.9)	127.6 (127.3, 127.8)	140.0 (139.6, 140.4)	152.8 (152.2, 153.5)	161.5 (160.6, 162.4)	180.5 (178.6, 182.4)
20	58	93.58 (92.79, 94.38)	102.57 (102.09, 103.0)	107.7 (107.3, 108.0)	116.7 (116.4, 117.0)	127.9 (127.6, 128.1)	140.4 (140.1, 140.8)	153.4 (152.8, 154.0)	162.1 (161.2, 163.0)	180.9 (179.0, 182.7)
21	60	93.36 (92.60, 94.13)	102.45 (101.98, 102.9)	107.6 (107.2, 108.0)	116.8 (116.5, 117.1)	128.1 (127.8, 128.4)	140.9 (140.5, 141.3)	153.9 (153.3, 154.5)	162.7 (161.8, 163.5)	181.3 (179.5, 183.1)
22	62	93.12 (92.40, 93.85)	102.31 (101.88, 102.7)	107.6 (107.2, 107.9)	116.9 (116.6, 117.2)	128.4 (128.1, 128.7)	141.3 (141.0, 141.7)	154.5 (153.9, 155.1)	163.2 (162.4, 164.1)	181.7 (180.0, 183.5)
23	64	92.86 (92.16, 93.56)	102.17 (101.76, 102.6)	107.5 (107.1, 107.8)	117.0 (116.7, 117.3)	128.7 (128.4, 129.0)	141.8 (141.4, 142.1)	155.0 (154.5, 155.6)	163.8 (163.0, 164.6)	182.2 (180.6, 183.9)
24	66	92.59 (91.89, 93.30)	102.03 (101.60, 102.4)	107.4 (107.1, 107.8)	117.1 (116.8, 117.4)	128.9 (128.6, 129.3)	142.2 (141.8, 142.6)	155.6 (155.0, 156.2)	164.4 (163.6, 165.2)	182.8 (181.2, 184.4)
25	68	92.31 (91.58, 93.04)	101.87 (101.43, 102.3)	107.3 (107.0, 107.7)	117.2 (116.8, 117.5)	129.2 (128.9, 129.5)	142.6 (142.3, 143.0)	156.2 (155.6, 156.7)	165.0 (164.2, 165.8)	183.4 (181.8, 185.0)
26	70	92.02 (91.25, 92.79)	101.71 (101.24, 102.2)	107.3 (106.9, 107.7)	117.2 (116.9, 117.6)	129.5 (129.1, 129.8)	143.1 (142.7, 143.5)	156.7 (156.1, 157.3)	165.6 (164.8, 166.4)	184.0 (182.5, 185.6)
27	72	91.72 (90.91, 92.54)	101.55 (101.04, 102.1)	107.2 (106.8, 107.6)	117.3 (116.9, 117.7)	129.7 (129.3, 130.1)	143.5 (143.1, 143.9)	157.3 (156.7, 157.9)	166.2 (165.4, 167.1)	184.7 (183.2, 186.3)
28	74	91.42 (90.58, 92.27)	101.39 (100.85, 101.9)	107.1 (106.7, 107.5)	117.4 (117.0, 117.8)	129.9 (129.6, 130.3)	143.9 (143.5, 144.3)	157.8 (157.2, 158.4)	166.9 (166.1, 167.7)	185.4 (183.9, 187.0)

Wong D, *et al. BMJ Open* 2020; 10:e033618. doi: 10.1136/bmjopen-2019-033618

	Age	_1st	_5th	_10th	_25th	_50th	_75th	_90th	_95th	_99th
29	76	91.12 (90.27, 91.97)	101.22 (100.68, 101.8)	107.0 (106.6, 107.5)	117.4 (117.1, 117.8)	130.2 (129.8, 130.6)	144.3 (143.9, 144.8)	158.4 (157.8, 159.0)	167.5 (166.7, 168.4)	186.2 (184.7, 187.7)
30	78	90.82 (89.99, 91.64)	101.05 (100.51, 101.6)	106.9 (106.5, 107.4)	117.5 (117.1, 117.9)	130.4 (130.0, 130.8)	144.7 (144.2, 145.2)	159.0 (158.3, 159.7)	168.2 (167.3, 169.1)	187.0 (185.4, 188.5)
31	80	90.51 (89.70, 91.31)	100.87 (100.33, 101.4)	106.8 (106.4, 107.3)	117.5 (117.2, 117.9)	130.6 (130.2, 131.1)	145.1 (144.6, 145.7)	159.5 (158.8, 160.3)	168.8 (167.8, 169.8)	187.8 (186.1, 189.5)
32	82	90.19 (89.39, 91.00)	100.70 (100.14, 101.3)	106.7 (106.3, 107.2)	117.6 (117.2, 118.0)	130.9 (130.4, 131.3)	145.5 (145.0, 146.1)	160.1 (159.3, 161.0)	169.5 (168.4, 170.6)	188.6 (186.7, 190.5)
33	84	89.87 (89.05, 90.68)	100.51 (99.94, 101.1)	106.6 (106.1, 107.1)	117.6 (117.2, 118.1)	131.1 (130.6, 131.6)	145.9 (145.3, 146.6)	160.7 (159.7, 161.6)	170.1 (168.9, 171.4)	189.4 (187.3, 191.5)
34	86	89.54 (88.70, 90.37)	100.32 (99.71, 100.9)	106.5 (106.0, 107.1)	117.7 (117.1, 118.2)	131.3 (130.7, 131.9)	146.3 (145.6, 147.0)	161.2 (160.2, 162.2)	170.8 (169.5, 172.1)	190.2 (187.9, 192.5)
35	88	89.20 (88.31, 90.08)	100.13 (99.44, 100.8)	106.4 (105.8, 107.1)	117.7 (117.1, 118.4)	131.5 (130.8, 132.2)	146.7 (146.0, 147.5)	161.8 (160.7, 162.9)	171.5 (170.0, 172.9)	191.0 (188.5, 193.6)
36	90	88.85 (87.84, 89.86)	99.93 (99.11, 100.7)	106.3 (105.5, 107.1)	117.8 (117.0, 118.5)	131.7 (130.9, 132.5)	147.1 (146.2, 148.0)	162.3 (161.1, 163.6)	172.1 (170.5, 173.7)	191.8 (189.0, 194.6)

Female systolic blood pressure centiles with 95% CI

	Age	_1st	_5th	_10th	_25th	_50th	_75th	_90th	_95th	_99th
1	20	88.97 (87.67, 90.27)	95.95 (95.19, 96.70)	99.69 (99.04, 100.34)	106.1 (105.5, 106.7)	113.8 (113.1, 114.4)	122.3 (121.6, 123.1)	131.3 (130.4, 132.1)	137.4 (136.5, 138.4)	151.4 (149.5, 153.4)
2	22	89.22 (88.16, 90.28)	95.99 (95.37, 96.62)	99.69 (99.17, 100.21)	106.1 (105.7, 106.6)	113.8 (113.3, 114.3)	122.5 (121.9, 123.1)	131.6 (130.9, 132.4)	137.9 (137.0, 138.8)	152.2 (150.6, 153.8)
3	24	89.37 (88.44, 90.30)	95.99 (95.40, 96.58)	99.64 (99.13, 100.15)	106.0 (105.6, 106.5)	113.8 (113.3, 114.3)	122.6 (122.0, 123.3)	132.0 (131.1, 132.8)	138.4 (137.4, 139.4)	153.0 (151.2, 154.9)
4	26	89.41 (88.60, 90.22)	95.91 (95.37, 96.45)	99.53 (99.03, 100.02)	105.9 (105.5, 106.4)	113.8 (113.3, 114.3)	122.8 (122.1, 123.4)	132.3 (131.4, 133.1)	138.9 (137.9, 139.9)	153.9 (152.0, 155.9)
5	28	89.35 (88.64, 90.06)	95.76 (95.31, 96.20)	99.36 (98.95, 99.76)	105.8 (105.4, 106.2)	113.7 (113.3, 114.2)	122.9 (122.3, 123.5)	132.6 (131.8, 133.4)	139.4 (138.4, 140.4)	154.9 (153.0, 156.9)
6	30	89.26 (88.58, 89.93)	95.61 (95.22, 96.01)	99.22 (98.84, 99.59)	105.7 (105.3, 106.1)	113.7 (113.3, 114.2)	123.1 (122.5, 123.7)	133.1 (132.3, 133.9)	140.1 (139.1, 141.1)	156.2 (154.1, 158.2)

Wong D, *et al. BMJ Open* 2020; 10:e033618. doi: 10.1136/bmjopen-2019-033618

	Age	_1st	_5th	_10th	_25th	_50th	_75th	_90th	_95th	_99th
7	32	89.20 (88.51, 89.88)	95.55 (95.13, 95.96)	99.17 (98.78, 99.55)	105.7 (105.3, 106.1)	113.9 (113.4, 114.4)	123.5 (122.9, 124.1)	133.8 (133.0, 134.6)	141.0 (140.0, 142.1)	157.7 (155.5, 159.8)
8	34	89.21 (88.52, 89.89)	95.59 (95.17, 96.01)	99.25 (98.87, 99.63)	105.9 (105.5, 106.3)	114.3 (113.8, 114.7)	124.1 (123.5, 124.7)	134.8 (134.0, 135.6)	142.3 (141.2, 143.3)	159.5 (157.3, 161.7)
9	36	89.28 (88.61, 89.95)	95.73 (95.33, 96.14)	99.45 (99.09, 99.81)	106.2 (105.9, 106.6)	114.8 (114.4, 115.2)	125.0 (124.5, 125.5)	136.0 (135.2, 136.7)	143.7 (142.7, 144.8)	161.7 (159.4, 163.9)
10	38	89.42 (88.77, 90.07)	95.97 (95.58, 96.36)	99.77 (99.42, 100.11)	106.7 (106.3, 107.1)	115.5 (115.1, 116.0)	126.0 (125.5, 126.5)	137.4 (136.7, 138.1)	145.4 (144.4, 146.5)	164.0 (161.7, 166.3)
11	40	89.61 (88.97, 90.25)	96.29 (95.91, 96.68)	100.18 (99.84, 100.53)	107.3 (106.9, 107.7)	116.4 (116.0, 116.8)	127.2 (126.7, 127.7)	139.0 (138.2, 139.7)	147.3 (146.2, 148.3)	166.4 (164.1, 168.8)
12	42	89.85 (89.20, 90.49)	96.69 (96.29, 97.09)	100.68 (100.32, 101.04)	108.0 (107.6, 108.4)	117.4 (116.9, 117.8)	128.5 (128.0, 129.1)	140.7 (139.9, 141.5)	149.2 (148.1, 150.3)	168.9 (166.6, 171.2)
13	44	90.12 (89.45, 90.79)	97.13 (96.70, 97.56)	101.23 (100.84, 101.62)	108.8 (108.4, 109.2)	118.4 (118.0, 118.9)	129.9 (129.3, 130.5)	142.4 (141.6, 143.2)	151.2 (150.1, 152.2)	171.3 (169.0, 173.5)
14	46	90.40 (89.70, 91.09)	97.60 (97.14, 98.06)	101.81 (101.40, 102.22)	109.6 (109.2, 110.0)	119.5 (119.1, 119.9)	131.3 (130.7, 131.9)	144.1 (143.3, 144.9)	153.0 (152.0, 154.1)	173.4 (171.3, 175.6)
15	48	90.66 (89.97, 91.36)	98.06 (97.57, 98.54)	102.38 (101.95, 102.81)	110.4 (109.9, 110.8)	120.6 (120.1, 121.0)	132.6 (132.1, 133.2)	145.7 (144.9, 146.4)	154.8 (153.8, 155.7)	175.3 (173.3, 177.4)
16	50	90.91 (90.23, 91.58)	98.50 (98.01, 98.98)	102.94 (102.50, 103.38)	111.1 (110.7, 111.5)	121.6 (121.1, 122.0)	133.9 (133.4, 134.4)	147.1 (146.4, 147.9)	156.3 (155.4, 157.3)	177.0 (175.0, 178.9)
17	52	91.13 (90.48, 91.77)	98.92 (98.45, 99.39)	103.48 (103.03, 103.93)	111.9 (111.4, 112.3)	122.5 (122.0, 123.1)	135.1 (134.5, 135.7)	148.5 (147.8, 149.2)	157.8 (156.8, 158.7)	178.4 (176.6, 180.2)
18	54	91.32 (90.69, 91.96)	99.32 (98.86, 99.79)	104.00 (103.55, 104.45)	112.6 (112.1, 113.1)	123.5 (123.0, 124.0)	136.3 (135.7, 136.9)	149.8 (149.1, 150.6)	159.1 (158.2, 160.0)	179.6 (177.8, 181.4)
19	56	91.51 (90.83, 92.19)	99.72 (99.24, 100.21)	104.52 (104.07, 104.98)	113.3 (112.9, 113.8)	124.5 (124.0, 125.0)	137.4 (136.9, 138.0)	151.1 (150.4, 151.8)	160.4 (159.5, 161.3)	180.7 (178.9, 182.5)
20	58	91.68 (90.92, 92.45)	100.12 (99.60, 100.64)	105.04 (104.58, 105.50)	114.0 (113.6, 114.5)	125.4 (124.9, 125.9)	138.5 (138.0, 139.0)	152.3 (151.7, 152.9)	161.6 (160.7, 162.5)	181.8 (179.9, 183.7)
21	60	91.86 (91.01, 92.71)	100.52 (99.95, 101.08)	105.56 (105.07, 106.04)	114.8 (114.3, 115.2)	126.3 (125.9, 126.7)	139.7 (139.2, 140.1)	153.5 (152.9, 154.1)	162.8 (162.0, 163.7)	182.8 (180.8, 184.8)

Wong D, *et al. BMJ Open* 2020; 10:e033618. doi: 10.1136/bmjopen-2019-033618

	Age	_1st	_5th	_10th	_25th	_50th	_75th	_90th	_95th	_99th
22	62	92.04 (91.15, 92.92)	100.91 (100.32, 101.50)	106.07 (105.56, 106.59)	115.5 (115.0, 115.9)	127.3 (126.8, 127.7)	140.7 (140.4, 141.1)	154.7 (154.1, 155.2)	164.0 (163.2, 164.9)	183.9 (181.9, 186.0)
23	64	92.22 (91.31, 93.12)	101.30 (100.69, 101.91)	106.58 (106.03, 107.12)	116.2 (115.7, 116.7)	128.2 (127.7, 128.6)	141.8 (141.4, 142.3)	155.9 (155.3, 156.5)	165.2 (164.4, 166.1)	185.1 (183.1, 187.1)
24	66	92.39 (91.43, 93.36)	101.68 (101.06, 102.30)	107.07 (106.50, 107.64)	116.8 (116.3, 117.4)	129.0 (128.5, 129.5)	142.9 (142.4, 143.4)	157.1 (156.4, 157.7)	166.4 (165.6, 167.3)	186.3 (184.3, 188.2)
25	68	92.57 (91.56, 93.58)	102.05 (101.41, 102.69)	107.54 (106.95, 108.13)	117.5 (116.9, 118.1)	129.9 (129.3, 130.4)	143.9 (143.3, 144.5)	158.2 (157.5, 158.9)	167.6 (166.7, 168.6)	187.5 (185.5, 189.4)
26	70	92.73 (91.78, 93.68)	102.39 (101.77, 103.02)	107.99 (107.40, 108.58)	118.1 (117.5, 118.7)	130.7 (130.1, 131.3)	144.9 (144.3, 145.5)	159.3 (158.6, 160.1)	168.8 (167.9, 169.7)	188.7 (186.9, 190.5)
27	72	92.88 (92.07, 93.68)	102.71 (102.13, 103.29)	108.40 (107.83, 108.97)	118.7 (118.1, 119.3)	131.5 (130.9, 132.0)	145.8 (145.2, 146.5)	160.4 (159.6, 161.2)	170.0 (169.0, 170.9)	189.9 (188.3, 191.5)
28	74	93.00 (92.30, 93.69)	102.99 (102.44, 103.54)	108.77 (108.23, 109.32)	119.2 (118.7, 119.8)	132.2 (131.6, 132.7)	146.7 (146.1, 147.4)	161.4 (160.6, 162.3)	171.1 (170.0, 172.1)	191.1 (189.4, 192.8)
29	76	93.09 (92.40, 93.77)	103.23 (102.69, 103.77)	109.10 (108.57, 109.63)	119.7 (119.2, 120.2)	132.8 (132.2, 133.4)	147.5 (146.9, 148.2)	162.4 (161.5, 163.2)	172.1 (171.0, 173.2)	192.2 (190.4, 194.1)
30	78	93.15 (92.39, 93.91)	103.43 (102.86, 103.99)	109.37 (108.84, 109.90)	120.1 (119.6, 120.6)	133.4 (132.8, 133.9)	148.3 (147.6, 148.9)	163.2 (162.4, 164.1)	173.0 (171.9, 174.2)	193.3 (191.3, 195.3)
31	80	93.18 (92.34, 94.01)	103.58 (102.98, 104.17)	109.59 (109.06, 110.12)	120.5 (120.0, 120.9)	133.9 (133.4, 134.4)	148.9 (148.3, 149.5)	164.0 (163.2, 164.9)	173.9 (172.7, 175.0)	194.3 (192.2, 196.3)
32	82	93.18 (92.35, 94.02)	103.69 (103.09, 104.28)	109.76 (109.24, 110.28)	120.7 (120.3, 121.2)	134.3 (133.8, 134.8)	149.5 (148.9, 150.0)	164.7 (163.9, 165.5)	174.7 (173.5, 175.8)	195.2 (193.2, 197.2)
33	84	93.17 (92.34, 93.99)	103.76 (103.16, 104.36)	109.89 (109.36, 110.42)	121.0 (120.5, 121.5)	134.6 (134.1, 135.1)	149.9 (149.3, 150.5)	165.3 (164.5, 166.2)	175.3 (174.2, 176.5)	196.0 (194.0, 198.0)
34	86	93.13 (92.30, 93.96)	103.81 (103.18, 104.43)	109.98 (109.42, 110.55)	121.1 (120.6, 121.7)	134.9 (134.3, 135.5)	150.4 (149.6, 151.1)	165.8 (164.9, 166.8)	175.9 (174.7, 177.2)	196.7 (194.7, 198.8)
35	88	93.08 (92.23, 93.92)	103.82 (103.19, 104.46)	110.05 (109.44, 110.65)	121.3 (120.7, 121.9)	135.2 (134.4, 135.9)	150.7 (149.8, 151.6)	166.3 (165.2, 167.4)	176.5 (175.1, 177.9)	197.4 (195.1, 199.7)
36	90	93.01 (92.14, 93.88)	103.82 (103.16, 104.48)	110.08 (109.44, 110.72)	121.4 (120.7, 122.1)	135.4 (134.5, 136.2)	151.0 (150.0, 152.0)	166.7 (165.4, 168.0)	176.9 (175.3, 178.6)	198.0 (195.3, 200.7)

Male diastolic blood pressure centiles with 95% CI

	Age	_1st	_5th	_10th	_25th	_50th	_75th	_90th	_95th	_99th
1	20	45.53 (44.75, 46.31)	51.56 (50.97, 52.14)	54.58 (54.06, 55.10)	59.55 (59.08, 60.02)	65.51 (65.02, 65.99)	72.36 (71.77, 72.95)	79.18 (78.42, 79.94)	83.60 (82.70, 84.49)	92.75 (91.48, 94.02)
2	22	45.83 (45.20, 46.46)	52.09 (51.66, 52.53)	55.23 (54.85, 55.60)	60.39 (60.06, 60.72)	66.58 (66.23, 66.92)	73.69 (73.25, 74.13)	80.78 (80.20, 81.35)	85.36 (84.67, 86.05)	94.87 (93.85, 95.89)
3	24	46.18 (45.62, 46.75)	52.66 (52.29, 53.03)	55.90 (55.59, 56.22)	61.25 (60.98, 61.51)	67.65 (67.37, 67.92)	75.00 (74.65, 75.36)	82.33 (81.85, 82.81)	87.08 (86.50, 87.66)	96.91 (96.03, 97.79)
4	26	46.58 (46.02, 47.14)	53.26 (52.88, 53.63)	56.60 (56.28, 56.91)	62.10 (61.83, 62.37)	68.70 (68.42, 68.97)	76.28 (75.93, 76.62)	83.83 (83.37, 84.28)	88.72 (88.17, 89.26)	98.85 (98.02, 99.67)
5	28	47.01 (46.43, 47.59)	53.86 (53.46, 54.26)	57.29 (56.95, 57.64)	62.94 (62.64, 63.24)	69.71 (69.41, 70.02)	77.49 (77.13, 77.86)	85.24 (84.78, 85.71)	90.26 (89.71, 90.81)	100.66 (99.85, 101.47)
6	30	47.47 (46.87, 48.07)	54.47 (54.05, 54.89)	57.98 (57.61, 58.35)	63.76 (63.43, 64.09)	70.68 (70.35, 71.01)	78.64 (78.25, 79.02)	86.56 (86.08, 87.04)	91.69 (91.14, 92.25)	102.33 (101.52, 103.14)
7	32	47.93 (47.32, 48.54)	55.07 (54.63, 55.50)	58.64 (58.26, 59.03)	64.53 (64.19, 64.88)	71.59 (71.24, 71.93)	79.69 (79.30, 80.09)	87.77 (87.29, 88.26)	93.00 (92.44, 93.56)	103.84 (103.03, 104.65)
8	34	48.40 (47.80, 49.00)	55.65 (55.22, 56.08)	59.28 (58.89, 59.66)	65.26 (64.91, 65.60)	72.42 (72.08, 72.77)	80.66 (80.27, 81.05)	88.86 (88.38, 89.34)	94.17 (93.62, 94.72)	105.18 (104.38, 105.97)
9	36	48.85 (48.27, 49.43)	56.19 (55.78, 56.61)	59.87 (59.50, 60.24)	65.92 (65.59, 66.26)	73.17 (72.84, 73.51)	81.51 (81.14, 81.89)	89.82 (89.36, 90.27)	95.19 (94.66, 95.72)	106.34 (105.56, 107.11)
10	38	49.29 (48.73, 49.84)	56.70 (56.31, 57.09)	60.41 (60.06, 60.76)	66.52 (66.20, 66.83)	73.84 (73.53, 74.15)	82.25 (81.91, 82.60)	90.64 (90.21, 91.06)	96.06 (95.57, 96.56)	107.31 (106.56, 108.06)
11	40	49.70 (49.17, 50.22)	57.15 (56.79, 57.52)	60.89 (60.57, 61.21)	67.04 (66.75, 67.33)	74.41 (74.13, 74.69)	82.88 (82.57, 83.19)	91.32 (90.93, 91.71)	96.78 (96.32, 97.24)	108.10 (107.38, 108.82)
12	42	50.06 (49.56, 50.56)	57.55 (57.21, 57.89)	61.30 (61.00, 61.60)	67.48 (67.21, 67.74)	74.88 (74.62, 75.13)	83.38 (83.10, 83.66)	91.85 (91.50, 92.21)	97.34 (96.91, 97.77)	108.71 (108.01, 109.40)
13	44	50.38 (49.90, 50.86)	57.88 (57.56, 58.21)	61.64 (61.36, 61.92)	67.83 (67.58, 68.07)	75.24 (75.01, 75.47)	83.76 (83.51, 84.01)	92.25 (91.92, 92.57)	97.74 (97.34, 98.14)	109.13 (108.45, 109.80)
14	46	50.65 (50.18, 51.12)	58.15 (57.84, 58.46)	61.90 (61.63, 62.18)	68.09 (67.85, 68.33)	75.50 (75.28, 75.71)	84.02 (83.79, 84.24)	92.50 (92.20, 92.80)	97.99 (97.61, 98.37)	109.38 (108.71, 110.04)
15	48	50.85 (50.39, 51.31)	58.34 (58.03, 58.65)	62.08 (61.81, 62.36)	68.26 (68.01, 68.50)	75.65 (75.44, 75.86)	84.15 (83.93, 84.37)	92.62 (92.33, 92.91)	98.10 (97.73, 98.47)	109.46 (108.79, 110.12)

	Age	_1st	_5th	_10th	_25th	_50th	_75th	_90th	_95th	_99th
16	50	50.99 (50.53, 51.45)	58.45 (58.13, 58.77)	62.18 (61.90, 62.47)	68.33 (68.08, 68.58)	75.70 (75.48, 75.92)	84.16 (83.94, 84.39)	92.60 (92.31, 92.89)	98.06 (97.69, 98.43)	109.38 (108.71, 110.05)
17	52	51.06 (50.60, 51.52)	58.48 (58.15, 58.81)	62.19 (61.89, 62.49)	68.31 (68.04, 68.57)	75.64 (75.41, 75.87)	84.07 (83.83, 84.30)	92.46 (92.16, 92.76)	97.89 (97.52, 98.27)	109.15 (108.48, 109.83)
18	54	51.05 (50.59, 51.52)	58.43 (58.09, 58.76)	62.12 (61.81, 62.43)	68.20 (67.92, 68.48)	75.48 (75.24, 75.73)	83.86 (83.62, 84.10)	92.20 (91.90, 92.51)	97.60 (97.22, 97.99)	108.80 (108.12, 109.48)
19	56	50.97 (50.50, 51.44)	58.29 (57.95, 58.64)	61.96 (61.64, 62.28)	68.00 (67.71, 68.29)	75.23 (74.98, 75.49)	83.55 (83.30, 83.81)	91.84 (91.53, 92.15)	97.20 (96.81, 97.59)	108.32 (107.63, 109.00)
20	58	50.81 (50.35, 51.28)	58.08 (57.73, 58.43)	61.72 (61.40, 62.04)	67.72 (67.42, 68.01)	74.90 (74.63, 75.16)	83.15 (82.89, 83.41)	91.38 (91.06, 91.70)	96.70 (96.31, 97.10)	107.74 (107.05, 108.42)
21	60	50.58 (50.13, 51.04)	57.79 (57.45, 58.14)	61.41 (61.09, 61.72)	67.35 (67.06, 67.64)	74.48 (74.22, 74.74)	82.67 (82.41, 82.93)	90.83 (90.52, 91.15)	96.12 (95.72, 96.51)	107.06 (106.37, 107.76)
22	62	50.28 (49.84, 50.73)	57.44 (57.11, 57.77)	61.02 (60.72, 61.33)	66.92 (66.64, 67.20)	73.99 (73.74, 74.25)	82.12 (81.87, 82.38)	90.22 (89.90, 90.54)	95.46 (95.07, 95.85)	106.32 (105.64, 107.01)
23	64	49.91 (49.48, 50.35)	57.02 (56.70, 57.33)	60.57 (60.28, 60.86)	66.43 (66.17, 66.69)	73.45 (73.21, 73.68)	81.51 (81.27, 81.75)	89.55 (89.24, 89.86)	94.75 (94.36, 95.14)	105.53 (104.85, 106.22)
24	66	49.49 (49.06, 49.91)	56.54 (56.24, 56.84)	60.07 (59.80, 60.34)	65.88 (65.64, 66.13)	72.85 (72.63, 73.07)	80.86 (80.63, 81.09)	88.84 (88.54, 89.14)	94.00 (93.62, 94.39)	104.71 (104.02, 105.39)
25	68	49.01 (48.59, 49.42)	56.01 (55.73, 56.29)	59.52 (59.27, 59.77)	65.30 (65.07, 65.52)	72.22 (72.02, 72.42)	80.18 (79.96, 80.39)	88.10 (87.81, 88.40)	93.24 (92.85, 93.62)	103.87 (103.19, 104.55)
26	70	48.48 (48.07, 48.89)	55.45 (55.18, 55.72)	58.94 (58.70, 59.17)	64.68 (64.48, 64.89)	71.57 (71.38, 71.75)	79.48 (79.27, 79.69)	87.37 (87.07, 87.66)	92.47 (92.09, 92.85)	103.04 (102.37, 103.72)
27	72	47.92 (47.51, 48.34)	54.86 (54.59, 55.13)	58.33 (58.10, 58.56)	64.05 (63.85, 64.25)	70.91 (70.73, 71.09)	78.79 (78.58, 79.00)	86.64 (86.34, 86.94)	91.72 (91.34, 92.10)	102.25 (101.57, 102.93)
28	74	47.34 (46.92, 47.76)	54.26 (53.99, 54.53)	57.72 (57.49, 57.96)	63.43 (63.22, 63.63)	70.26 (70.07, 70.46)	78.12 (77.90, 78.34)	85.95 (85.64, 86.26)	91.01 (90.62, 91.41)	101.51 (100.84, 102.19)
29	76	46.75 (46.31, 47.19)	53.66 (53.37, 53.95)	57.12 (56.87, 57.37)	62.82 (62.59, 63.04)	69.64 (69.43, 69.86)	77.49 (77.25, 77.74)	85.31 (84.99, 85.64)	90.37 (89.97, 90.78)	100.86 (100.18, 101.54)
30	78	46.16 (45.71, 46.62)	53.08 (52.77, 53.38)	56.54 (56.27, 56.81)	62.24 (61.99, 62.49)	69.08 (68.83, 69.32)	76.93 (76.66, 77.20)	84.76 (84.41, 85.10)	89.82 (89.40, 90.24)	100.32 (99.63, 101.00)

Wong D, *et al. BMJ Open* 2020; 10:e033618. doi: 10.1136/bmjopen-2019-033618

	Age	_1st	_5th	_10th	_25th	_50th	_75th	_90th	_95th	_99th
31	80	45.59 (45.12, 46.07)	52.53 (52.20, 52.85)	56.00 (55.71, 56.29)	61.72 (61.45, 61.99)	68.57 (68.30, 68.85)	76.45 (76.15, 76.76)	84.30 (83.93, 84.67)	89.38 (88.94, 89.82)	99.91 (99.22, 100.60)
32	82	45.06 (44.56, 45.55)	52.03 (51.69, 52.37)	55.52 (55.21, 55.83)	61.27 (60.98, 61.57)	68.16 (67.87, 68.46)	76.09 (75.75, 76.42)	83.98 (83.57, 84.38)	89.08 (88.61, 89.55)	99.67 (98.95, 100.39)
33	84	44.57 (44.05, 45.09)	51.60 (51.24, 51.97)	55.12 (54.80, 55.45)	60.92 (60.61, 61.23)	67.87 (67.55, 68.19)	75.85 (75.49, 76.22)	83.81 (83.36, 84.26)	88.96 (88.44, 89.48)	99.63 (98.86, 100.40)
34	86	44.16 (43.59, 44.72)	51.27 (50.87, 51.66)	54.83 (54.48, 55.17)	60.69 (60.36, 61.01)	67.71 (67.36, 68.06)	75.79 (75.37, 76.20)	83.83 (83.30, 84.35)	89.03 (88.42, 89.65)	99.83 (98.94, 100.71)
35	88	43.83 (43.18, 44.47)	51.04 (50.59, 51.49)	54.65 (54.25, 55.04)	60.59 (60.23, 60.95)	67.72 (67.32, 68.11)	75.91 (75.40, 76.41)	84.06 (83.42, 84.71)	89.35 (88.58, 90.11)	100.29 (99.22, 101.36)
36	90	43.60 (42.81, 44.39)	50.94 (50.37, 51.50)	54.61 (54.12, 55.10)	60.66 (60.22, 61.10)	67.91 (67.43, 68.40)	76.25 (75.61, 76.88)	84.55 (83.72, 85.39)	89.92 (88.94, 90.91)	101.06 (99.70, 102.43)

Female diastolic blood pressure centiles with 95% CI

	Age	_1st	_5th	_10th	_25th	_50th	_75th	_90th	_95th	_99th
1	20	45.22 (44.71, 45.73)	50.96 (50.58, 51.33)	53.83 (53.49, 54.16)	58.60 (58.29, 58.92)	64.63 (64.29, 64.97)	71.84 (71.40, 72.28)	79.17 (78.57, 79.78)	83.99 (83.25, 84.73)	94.15 (93.05, 95.26)
2	22	45.28 (44.83, 45.73)	51.13 (50.80, 51.46)	54.05 (53.76, 54.35)	58.92 (58.64, 59.21)	65.07 (64.77, 65.37)	72.42 (72.05, 72.79)	79.90 (79.39, 80.40)	84.81 (84.19, 85.42)	95.17 (94.23, 96.11)
3	24	45.35 (44.93, 45.77)	51.31 (51.02, 51.61)	54.29 (54.03, 54.56)	59.25 (59.00, 59.51)	65.51 (65.25, 65.78)	73.00 (72.68, 73.32)	80.62 (80.19, 81.05)	85.62 (85.09, 86.15)	96.18 (95.34, 97.01)
4	26	45.44 (45.04, 45.84)	51.50 (51.23, 51.77)	54.53 (54.29, 54.77)	59.58 (59.36, 59.81)	65.95 (65.72, 66.19)	73.57 (73.28, 73.85)	81.32 (80.94, 81.70)	86.41 (85.94, 86.89)	97.15 (96.38, 97.92)
5	28	45.53 (45.14, 45.92)	51.69 (51.44, 51.95)	54.77 (54.56, 54.99)	59.91 (59.70, 60.11)	66.38 (66.17, 66.59)	74.12 (73.86, 74.38)	82.00 (81.64, 82.36)	87.17 (86.73, 87.62)	98.09 (97.35, 98.82)
6	30	45.63 (45.24, 46.02)	51.89 (51.64, 52.13)	55.01 (54.81, 55.21)	60.22 (60.04, 60.40)	66.79 (66.59, 66.99)	74.65 (74.39, 74.90)	82.64 (82.30, 82.99)	87.89 (87.46, 88.33)	98.97 (98.25, 99.69)
7	32	45.74 (45.35, 46.13)	52.08 (51.84, 52.32)	55.25 (55.06, 55.44)	60.52 (60.35, 60.69)	67.18 (66.99, 67.37)	75.14 (74.90, 75.38)	83.24 (82.91, 83.58)	88.57 (88.14, 88.99)	99.79 (99.09, 100.49)
8	34	45.85 (45.46, 46.25)	52.27 (52.03, 52.50)	55.47 (55.29, 55.66)	60.81 (60.65, 60.97)	67.55 (67.36, 67.73)	75.60 (75.36, 75.84)	83.80 (83.46, 84.13)	89.18 (88.77, 89.59)	100.54 (99.85, 101.23)

Wong D, *et al. BMJ Open* 2020; 10:e033618. doi: 10.1136/bmjopen-2019-033618

	Age	_1st	_5th	_10th	_25th	_50th	_75th	_90th	_95th	_99th
9	36	45.97 (45.58, 46.37)	52.45 (52.21, 52.68)	55.68 (55.50, 55.87)	61.08 (60.91, 61.24)	67.88 (67.70, 68.07)	76.01 (75.78, 76.25)	84.29 (83.97, 84.62)	89.73 (89.33, 90.14)	101.20 (100.53, 101.88)
10	38	46.09 (45.69, 46.48)	52.62 (52.38, 52.86)	55.88 (55.69, 56.07)	61.32 (61.15, 61.49)	68.18 (68.00, 68.37)	76.38 (76.15, 76.62)	84.73 (84.42, 85.05)	90.22 (89.83, 90.61)	101.78 (101.13, 102.44)
11	40	46.20 (45.81, 46.60)	52.78 (52.54, 53.02)	56.07 (55.87, 56.26)	61.54 (61.37, 61.72)	68.45 (68.26, 68.64)	76.70 (76.47, 76.94)	85.11 (84.80, 85.42)	90.63 (90.25, 91.01)	102.28 (101.64, 102.92)
12	42	46.32 (45.91, 46.72)	52.93 (52.68, 53.18)	56.23 (56.03, 56.43)	61.73 (61.55, 61.92)	68.68 (68.48, 68.87)	76.98 (76.74, 77.21)	85.42 (85.12, 85.73)	90.98 (90.60, 91.35)	102.68 (102.05, 103.31)
13	44	46.43 (46.02, 46.83)	53.06 (52.80, 53.32)	56.37 (56.16, 56.59)	61.90 (61.71, 62.09)	68.87 (68.67, 69.06)	77.19 (76.96, 77.43)	85.67 (85.38, 85.97)	91.24 (90.88, 91.61)	102.99 (102.36, 103.62)
14	46	46.53 (46.11, 46.94)	53.17 (52.91, 53.44)	56.50 (56.28, 56.71)	62.03 (61.83, 62.23)	69.01 (68.81, 69.22)	77.36 (77.13, 77.59)	85.86 (85.56, 86.16)	91.44 (91.08, 91.80)	103.21 (102.59, 103.84)
15	48	46.62 (46.20, 47.04)	53.27 (53.00, 53.54)	56.59 (56.37, 56.82)	62.13 (61.93, 62.33)	69.12 (68.92, 69.33)	77.48 (77.24, 77.71)	85.98 (85.68, 86.28)	91.57 (91.20, 91.93)	103.35 (102.72, 103.98)
16	50	46.69 (46.27, 47.12)	53.34 (53.07, 53.62)	56.67 (56.44, 56.90)	62.20 (62.00, 62.41)	69.19 (68.99, 69.40)	77.54 (77.31, 77.77)	86.04 (85.74, 86.34)	91.63 (91.26, 92.00)	103.41 (102.76, 104.05)
17	52	46.76 (46.33, 47.18)	53.40 (53.12, 53.67)	56.72 (56.49, 56.95)	62.24 (62.04, 62.45)	69.22 (69.02, 69.43)	77.56 (77.32, 77.79)	86.05 (85.74, 86.35)	91.62 (91.25, 92.00)	103.38 (102.73, 104.04)
18	54	46.80 (46.38, 47.23)	53.43 (53.16, 53.70)	56.74 (56.51, 56.97)	62.25 (62.05, 62.46)	69.21 (69.01, 69.42)	77.53 (77.30, 77.77)	86.00 (85.69, 86.31)	91.56 (91.18, 91.95)	103.30 (102.63, 103.96)
19	56	46.83 (46.41, 47.25)	53.44 (53.17, 53.70)	56.74 (56.51, 56.96)	62.23 (62.04, 62.43)	69.17 (68.97, 69.37)	77.46 (77.23, 77.70)	85.91 (85.60, 86.22)	91.45 (91.06, 91.84)	103.15 (102.47, 103.83)
20	58	46.84 (46.43, 47.25)	53.42 (53.16, 53.68)	56.71 (56.49, 56.92)	62.19 (61.99, 62.38)	69.10 (68.90, 69.30)	77.36 (77.13, 77.60)	85.77 (85.46, 86.09)	91.30 (90.90, 91.69)	102.95 (102.27, 103.64)
21	60	46.82 (46.43, 47.22)	53.38 (53.13, 53.63)	56.65 (56.45, 56.86)	62.11 (61.92, 62.30)	69.00 (68.80, 69.19)	77.23 (76.99, 77.46)	85.61 (85.29, 85.92)	91.11 (90.71, 91.51)	102.72 (102.02, 103.41)
22	62	46.79 (46.40, 47.17)	53.32 (53.08, 53.55)	56.58 (56.38, 56.77)	62.01 (61.83, 62.19)	68.87 (68.68, 69.06)	77.07 (76.83, 77.30)	85.41 (85.09, 85.73)	90.89 (90.49, 91.30)	102.45 (101.76, 103.15)
23	64	46.72 (46.35, 47.09)	53.23 (53.00, 53.45)	56.48 (56.29, 56.66)	61.89 (61.72, 62.06)	68.72 (68.53, 68.91)	76.89 (76.65, 77.12)	85.20 (84.88, 85.52)	90.66 (90.26, 91.07)	102.18 (101.48, 102.88)

Wong D, *et al. BMJ Open* 2020; 10:e033618. doi: 10.1136/bmjopen-2019-033618

	Age	_1st	_5th	_10th	_25th	_50th	_75th	_90th	_95th	_99th
24	66	46.63 (46.28, 46.99)	53.12 (52.90, 53.33)	56.35 (56.18, 56.53)	61.75 (61.58, 61.92)	68.56 (68.37, 68.75)	76.70 (76.46, 76.93)	84.98 (84.66, 85.31)	90.43 (90.02, 90.83)	101.90 (101.20, 102.61)
25	68	46.52 (46.17, 46.87)	52.98 (52.78, 53.19)	56.21 (56.04, 56.38)	61.60 (61.43, 61.76)	68.39 (68.20, 68.58)	76.50 (76.26, 76.74)	84.77 (84.44, 85.09)	90.19 (89.78, 90.61)	101.64 (100.93, 102.35)
26	70	46.38 (46.03, 46.73)	52.83 (52.63, 53.04)	56.06 (55.89, 56.22)	61.43 (61.27, 61.59)	68.21 (68.02, 68.40)	76.31 (76.07, 76.56)	84.56 (84.23, 84.90)	89.98 (89.56, 90.40)	101.41 (100.69, 102.13)
27	72	46.21 (45.85, 46.57)	52.66 (52.45, 52.87)	55.89 (55.71, 56.06)	61.26 (61.09, 61.42)	68.03 (67.85, 68.22)	76.13 (75.89, 76.38)	84.38 (84.04, 84.72)	89.80 (89.37, 90.23)	101.23 (100.50, 101.95)
28	74	46.02 (45.65, 46.38)	52.48 (52.25, 52.70)	55.70 (55.52, 55.88)	61.08 (60.92, 61.25)	67.87 (67.68, 68.06)	75.98 (75.73, 76.23)	84.24 (83.89, 84.58)	89.66 (89.23, 90.10)	101.10 (100.37, 101.84)
29	76	45.79 (45.40, 46.18)	52.28 (52.04, 52.51)	55.51 (55.32, 55.71)	60.91 (60.74, 61.08)	67.72 (67.53, 67.91)	75.86 (75.61, 76.11)	84.14 (83.79, 84.50)	89.59 (89.14, 90.03)	101.07 (100.32, 101.81)
30	78	45.54 (45.13, 45.96)	52.06 (51.80, 52.32)	55.32 (55.11, 55.53)	60.75 (60.57, 60.92)	67.60 (67.41, 67.78)	75.78 (75.53, 76.03)	84.11 (83.76, 84.47)	89.59 (89.14, 90.04)	101.13 (100.38, 101.88)
31	80	45.27 (44.83, 45.71)	51.84 (51.56, 52.13)	55.13 (54.89, 55.36)	60.60 (60.41, 60.79)	67.51 (67.32, 67.69)	75.76 (75.51, 76.00)	84.16 (83.81, 84.51)	89.68 (89.24, 90.13)	101.32 (100.57, 102.08)
32	82	44.96 (44.49, 45.44)	51.61 (51.30, 51.93)	54.94 (54.68, 55.19)	60.47 (60.27, 60.67)	67.46 (67.27, 67.65)	75.81 (75.56, 76.05)	84.31 (83.96, 84.66)	89.89 (89.45, 90.33)	101.67 (100.92, 102.41)
33	84	44.63 (44.12, 45.14)	51.38 (51.03, 51.73)	54.75 (54.47, 55.04)	60.37 (60.15, 60.59)	67.46 (67.27, 67.66)	75.94 (75.69, 76.18)	84.57 (84.22, 84.91)	90.23 (89.79, 90.67)	102.19 (101.45, 102.92)
34	86	44.27 (43.72, 44.81)	51.14 (50.76, 51.53)	54.58 (54.26, 54.90)	60.31 (60.06, 60.55)	67.53 (67.31, 67.75)	76.17 (75.91, 76.43)	84.96 (84.60, 85.32)	90.73 (90.29, 91.18)	102.91 (102.18, 103.65)
35	88	43.87 (43.27, 44.46)	50.90 (50.47, 51.34)	54.42 (54.06, 54.79)	60.28 (59.99, 60.57)	67.67 (67.42, 67.93)	76.51 (76.21, 76.81)	85.51 (85.11, 85.90)	91.42 (90.93, 91.90)	103.88 (103.12, 104.64)
36	90	43.43 (42.76, 44.10)	50.66 (50.17, 51.16)	54.28 (53.86, 54.70)	60.30 (59.96, 60.64)	67.90 (67.59, 68.22)	76.99 (76.61, 77.36)	86.24 (85.75, 86.73)	92.32 (91.73, 92.90)	105.13 (104.27, 105.99)

Wong D, *et al. BMJ Open* 2020; 10:e033618. doi: 10.1136/bmjopen-2019-033618

Male pulse pressure centiles with 95% CI

	Age	1st	5th	10th	25th	50th	75th	90th	95th	99th
1	20	31.75 (30.29, 33.22)	39.16 (38.33, 39.99)	42.78 (42.07, 43.49)	48.59 (47.94, 49.25)	55.24 (54.63, 55.85)	62.62 (62.06, 63.19)	69.94 (69.29, 70.58)	74.70 (73.92, 75.48)	84.71 (83.29, 86.13)
2	22	31.54 (30.33, 32.75)	38.84 (38.21, 39.48)	42.40 (41.87, 42.93)	48.12 (47.62, 48.61)	54.69 (54.21, 55.18)	62.06 (61.55, 62.56)	69.38 (68.77, 69.99)	74.17 (73.44, 74.90)	84.28 (83.02, 85.54)
3	24	31.28 (30.24, 32.32)	38.47 (37.96, 38.99)	41.97 (41.55, 42.38)	47.59 (47.19, 47.99)	54.09 (53.67, 54.51)	61.43 (60.94, 61.93)	68.78 (68.12, 69.43)	73.59 (72.79, 74.39)	83.81 (82.46, 85.15)
4	26	30.95 (29.96, 31.95)	38.04 (37.51, 38.57)	41.47 (41.04, 41.91)	47.00 (46.59, 47.41)	53.43 (53.00, 53.87)	60.75 (60.22, 61.29)	68.12 (67.41, 68.82)	72.96 (72.10, 73.82)	83.29 (81.85, 84.74)
5	28	30.57 (29.63, 31.52)	37.56 (37.05, 38.06)	40.93 (40.52, 41.35)	46.37 (45.99, 46.74)	52.74 (52.34, 53.14)	60.05 (59.56, 60.54)	67.43 (66.77, 68.09)	72.31 (71.49, 73.14)	82.78 (81.31, 84.24)
6	30	30.16 (29.32, 30.99)	37.05 (36.62, 37.47)	40.37 (40.02, 40.72)	45.72 (45.38, 46.06)	52.04 (51.66, 52.41)	59.34 (58.88, 59.80)	66.76 (66.15, 67.38)	71.69 (70.89, 72.49)	82.30 (80.80, 83.80)
7	32	29.72 (28.96, 30.48)	36.53 (36.12, 36.94)	39.81 (39.44, 40.18)	45.08 (44.69, 45.47)	51.36 (50.93, 51.80)	58.68 (58.18, 59.18)	66.15 (65.50, 66.80)	71.13 (70.31, 71.96)	81.92 (80.35, 83.49)
8	34	29.29 (28.56, 30.02)	36.04 (35.64, 36.43)	39.28 (38.93, 39.62)	44.49 (44.14, 44.84)	50.75 (50.37, 51.13)	58.10 (57.65, 58.54)	65.64 (65.04, 66.24)	70.69 (69.90, 71.47)	81.67 (80.10, 83.24)
9	36	28.89 (28.17, 29.62)	35.60 (35.19, 36.02)	38.81 (38.45, 39.17)	43.98 (43.65, 44.31)	50.24 (49.90, 50.58)	57.64 (57.24, 58.04)	65.27 (64.72, 65.83)	70.40 (69.65, 71.14)	81.62 (80.09, 83.15)
10	38	28.53 (27.82, 29.25)	35.23 (34.78, 35.67)	38.42 (38.04, 38.81)	43.57 (43.22, 43.92)	49.85 (49.50, 50.21)	57.32 (56.91, 57.74)	65.07 (64.51, 65.63)	70.29 (69.56, 71.03)	81.79 (80.31, 83.26)
11	40	28.18 (27.51, 28.85)	34.89 (34.44, 35.34)	38.09 (37.69, 38.49)	43.24 (42.87, 43.61)	49.57 (49.19, 49.96)	57.15 (56.70, 57.60)	65.04 (64.44, 65.64)	70.38 (69.62, 71.14)	82.18 (80.74, 83.63)
12	42	27.82 (27.18, 28.45)	34.58 (34.13, 35.03)	37.79 (37.38, 38.20)	42.98 (42.59, 43.37)	49.40 (48.98, 49.81)	57.11 (56.62, 57.61)	65.18 (64.54, 65.82)	70.66 (69.86, 71.46)	82.82 (81.38, 84.26)
13	44	27.44 (26.81, 28.07)	34.29 (33.84, 34.75)	37.54 (37.13, 37.96)	42.79 (42.40, 43.18)	49.33 (48.91, 49.74)	57.22 (56.72, 57.72)	65.50 (64.85, 66.15)	71.14 (70.33, 71.94)	83.69 (82.25, 85.13)
14	46	27.09 (26.43, 27.74)	34.05 (33.59, 34.51)	37.35 (36.94, 37.76)	42.69 (42.32, 43.06)	49.38 (48.99, 49.77)	57.49 (57.02, 57.96)	66.00 (65.39, 66.62)	71.81 (71.04, 72.58)	84.77 (83.35, 86.19)

Wong D, *et al. BMJ Open* 2020; 10:e033618. doi: 10.1136/bmjopen-2019-033618

	Age	1st	5th	10th	25th	50th	75th	90th	95th	99th
15	48	26.77 (26.09, 27.46)	33.86 (33.40, 34.32)	37.23 (36.83, 37.63)	42.69 (42.34, 43.05)	49.56 (49.20, 49.93)	57.91 (57.47, 58.35)	66.69 (66.11, 67.26)	72.67 (71.95, 73.40)	86.02 (84.64, 87.40)
16	50	26.52 (25.81, 27.23)	33.75 (33.30, 34.21)	37.20 (36.81, 37.59)	42.80 (42.46, 43.15)	49.88 (49.52, 50.24)	58.49 (58.06, 58.93)	67.54 (66.98, 68.09)	73.69 (73.00, 74.38)	87.38 (86.02, 88.74)
17	52	26.37 (25.65, 27.09)	33.73 (33.29, 34.18)	37.26 (36.89, 37.64)	43.03 (42.69, 43.36)	50.33 (49.97, 50.69)	59.21 (58.77, 59.66)	68.52 (67.96, 69.08)	74.83 (74.14, 75.52)	88.79 (87.43, 90.15)
18	54	26.33 (25.62, 27.04)	33.81 (33.37, 34.24)	37.42 (37.05, 37.79)	43.36 (43.03, 43.68)	50.90 (50.54, 51.26)	60.06 (59.60, 60.51)	69.61 (69.03, 70.18)	76.05 (75.34, 76.75)	90.15 (88.78, 91.52)
19	56	26.39 (25.70, 27.09)	33.96 (33.53, 34.39)	37.66 (37.30, 38.02)	43.78 (43.46, 44.09)	51.56 (51.20, 51.91)	60.98 (60.53, 61.43)	70.74 (70.16, 71.33)	77.28 (76.55, 78.00)	91.43 (90.06, 92.79)
20	58	26.49 (25.80, 27.17)	34.14 (33.71, 34.58)	37.93 (37.57, 38.30)	44.23 (43.91, 44.55)	52.25 (51.91, 52.60)	61.93 (61.49, 62.37)	71.89 (71.30, 72.48)	78.49 (77.75, 79.23)	92.59 (91.25, 93.93)
21	60	26.58 (25.90, 27.26)	34.32 (33.88, 34.76)	38.19 (37.82, 38.56)	44.68 (44.36, 45.00)	52.95 (52.61, 53.29)	62.88 (62.44, 63.32)	73.01 (72.41, 73.61)	79.66 (78.91, 80.42)	93.68 (92.38, 94.98)
22	62	26.71 (26.04, 27.39)	34.54 (34.09, 34.99)	38.51 (38.13, 38.89)	45.20 (44.87, 45.52)	53.71 (53.38, 54.05)	63.89 (63.46, 64.33)	74.18 (73.58, 74.79)	80.88 (80.13, 81.63)	94.79 (93.54, 96.04)
23	64	26.94 (26.28, 27.60)	34.86 (34.41, 35.32)	38.93 (38.54, 39.32)	45.82 (45.49, 46.15)	54.58 (54.26, 54.91)	65.01 (64.59, 65.42)	75.46 (74.88, 76.04)	82.21 (81.49, 82.93)	96.03 (94.86, 97.20)
24	66	27.24 (26.62, 27.86)	35.28 (34.84, 35.73)	39.45 (39.07, 39.84)	46.55 (46.21, 46.88)	55.57 (55.24, 55.89)	66.23 (65.84, 66.63)	76.86 (76.31, 77.40)	83.66 (82.98, 84.33)	97.42 (96.33, 98.52)
25	68	27.58 (26.98, 28.18)	35.76 (35.33, 36.19)	40.04 (39.66, 40.41)	47.35 (47.02, 47.68)	56.62 (56.30, 56.94)	67.53 (67.14, 67.92)	78.33 (77.81, 78.86)	85.19 (84.55, 85.84)	98.95 (97.89, 100.01)
26	70	27.85 (27.23, 28.47)	36.19 (35.76, 36.62)	40.59 (40.21, 40.96)	48.11 (47.79, 48.44)	57.63 (57.31, 57.95)	68.80 (68.41, 69.18)	79.78 (79.25, 80.31)	86.71 (86.05, 87.37)	100.50 (99.41, 101.59)
27	72	27.98 (27.30, 28.65)	36.50 (36.04, 36.96)	41.01 (40.62, 41.40)	48.76 (48.43, 49.10)	58.54 (58.20, 58.87)	69.94 (69.53, 70.35)	81.11 (80.55, 81.67)	88.13 (87.43, 88.82)	101.97 (100.83, 103.12)
28	74	27.93 (27.22, 28.63)	36.65 (36.17, 37.13)	41.29 (40.88, 41.70)	49.27 (48.90, 49.64)	59.29 (58.92, 59.67)	70.95 (70.51, 71.39)	82.32 (81.73, 82.90)	89.42 (88.70, 90.15)	103.37 (102.17, 104.56)
29	76	27.72 (27.03, 28.42)	36.65 (36.18, 37.12)	41.42 (41.01, 41.82)	49.62 (49.25, 50.00)	59.91 (59.52, 60.29)	71.82 (71.37, 72.26)	83.38 (82.78, 83.98)	90.60 (89.84, 91.35)	104.67 (103.41, 105.92)

	Age	1st	5th	10th	25th	50th	75th	90th	95th	99th
30	78	27.42 (26.71, 28.12)	36.54 (36.05, 37.04)	41.43 (41.01, 41.85)	49.86 (49.47, 50.24)	60.38 (59.98, 60.78)	72.54 (72.07, 73.02)	84.32 (83.65, 84.99)	91.64 (90.78, 92.50)	105.86 (104.46, 107.27)
31	80	27.13 (26.35, 27.91)	36.43 (35.85, 37.00)	41.42 (40.91, 41.93)	50.04 (49.57, 50.50)	60.79 (60.32, 61.26)	73.19 (72.63, 73.75)	85.17 (84.39, 85.95)	92.60 (91.61, 93.59)	106.99 (105.41, 108.57)
32	82	26.96 (26.14, 27.78)	36.40 (35.77, 37.02)	41.47 (40.91, 42.04)	50.25 (49.73, 50.77)	61.21 (60.69, 61.73)	73.83 (73.23, 74.42)	86.00 (85.18, 86.83)	93.54 (92.50, 94.58)	108.10 (106.45, 109.75)
33	84	26.93 (26.07, 27.78)	36.46 (35.83, 37.09)	41.60 (41.04, 42.16)	50.50 (49.98, 51.03)	61.63 (61.11, 62.16)	74.46 (73.86, 75.05)	86.82 (86.00, 87.64)	94.46 (93.43, 95.49)	109.19 (107.55, 110.82)
34	86	26.97 (25.99, 27.95)	36.57 (35.90, 37.24)	41.76 (41.19, 42.33)	50.76 (50.23, 51.29)	62.04 (61.49, 62.59)	75.05 (74.41, 75.69)	87.58 (86.70, 88.47)	95.32 (94.21, 96.44)	110.21 (108.50, 111.92)
35	88	27.03 (25.79, 28.27)	36.67 (35.88, 37.45)	41.89 (41.26, 42.51)	50.95 (50.42, 51.49)	62.35 (61.79, 62.92)	75.52 (74.80, 76.24)	88.20 (87.15, 89.25)	96.02 (94.71, 97.34)	111.05 (109.09, 113.02)
36	90	27.07 (25.45, 28.70)	36.72 (35.70, 37.74)	41.96 (41.18, 42.74)	51.07 (50.45, 51.69)	62.56 (61.94, 63.18)	75.85 (75.03, 76.66)	88.65 (87.41, 89.89)	96.54 (94.96, 98.11)	111.68 (109.35, 114.00)

Female PP centiles with 95% CI

	Age	1st	5th	10th	25th	50th	75th	90th	95th	99th
1	20	27.29 (26.29, 28.28)	33.96 (33.35, 34.58)	37.06 (36.57, 37.55)	41.94 (41.54, 42.34)	47.71 (47.21, 48.21)	54.50 (53.81, 55.19)	61.58 (60.71, 62.45)	66.42 (65.43, 67.42)	77.33 (75.64, 79.01)
2	22	27.09 (26.25, 27.93)	33.72 (33.20, 34.24)	36.80 (36.38, 37.22)	41.65 (41.32, 41.98)	47.40 (47.04, 47.76)	54.18 (53.70, 54.65)	61.26 (60.63, 61.88)	66.10 (65.34, 66.86)	77.01 (75.55, 78.46)
3	24	26.89 (26.14, 27.64)	33.48 (33.02, 33.94)	36.54 (36.17, 36.91)	41.37 (41.08, 41.66)	47.11 (46.82, 47.40)	53.88 (53.52, 54.25)	60.97 (60.45, 61.49)	65.82 (65.14, 66.49)	76.74 (75.40, 78.09)
4	26	26.70 (26.00, 27.40)	33.26 (32.84, 33.68)	36.31 (35.97, 36.65)	41.12 (40.85, 41.39)	46.86 (46.59, 47.13)	53.64 (53.30, 53.98)	60.74 (60.23, 61.25)	65.61 (64.92, 66.29)	76.57 (75.26, 77.88)
5	28	26.54 (25.86, 27.21)	33.08 (32.68, 33.48)	36.12 (35.80, 36.43)	40.93 (40.66, 41.19)	46.67 (46.40, 46.94)	53.49 (53.13, 53.84)	60.63 (60.09, 61.16)	65.52 (64.81, 66.23)	76.54 (75.25, 77.84)
6	30	26.41 (25.75, 27.07)	32.95 (32.56, 33.34)	35.99 (35.68, 36.30)	40.81 (40.55, 41.07)	46.59 (46.32, 46.86)	53.46 (53.10, 53.82)	60.67 (60.12, 61.21)	65.60 (64.88, 66.32)	76.73 (75.46, 78.01)
7	32	26.30 (25.66, 26.95)	32.87 (32.48, 33.26)	35.92 (35.61, 36.24)	40.77 (40.50, 41.04)	46.61 (46.34, 46.88)	53.57 (53.23, 53.91)	60.87 (60.35, 61.39)	65.88 (65.19, 66.57)	77.17 (75.91, 78.43)

Wong D, *et al. BMJ Open* 2020; 10:e033618. doi: 10.1136/bmjopen-2019-033618

	Age	1st	5th	10th	25th	50th	75th	90th	95th	99th
8	34	26.20 (25.58, 26.83)	32.82 (32.43, 33.21)	35.90 (35.57, 36.23)	40.80 (40.51, 41.09)	46.73 (46.45, 47.01)	53.81 (53.49, 54.14)	61.26 (60.77, 61.74)	66.36 (65.71, 67.01)	77.86 (76.62, 79.11)
9	36	26.09 (25.46, 26.71)	32.79 (32.39, 33.18)	35.91 (35.57, 36.26)	40.89 (40.57, 41.20)	46.94 (46.65, 47.23)	54.19 (53.88, 54.51)	61.82 (61.37, 62.27)	67.05 (66.44, 67.66)	78.83 (77.56, 80.11)
10	38	25.96 (25.29, 26.62)	32.77 (32.36, 33.17)	35.95 (35.59, 36.31)	41.03 (40.70, 41.36)	47.25 (46.95, 47.54)	54.71 (54.41, 55.01)	62.56 (62.15, 62.98)	67.95 (67.35, 68.55)	80.07 (78.66, 81.49)
11	40	25.81 (25.07, 26.55)	32.76 (32.35, 33.17)	36.02 (35.65, 36.38)	41.23 (40.90, 41.56)	47.64 (47.35, 47.94)	55.36 (55.06, 55.66)	63.48 (63.05, 63.92)	69.05 (68.38, 69.71)	81.56 (79.84, 83.29)
12	42	25.68 (24.86, 26.51)	32.79 (32.37, 33.21)	36.13 (35.77, 36.49)	41.50 (41.18, 41.82)	48.14 (47.85, 48.43)	56.14 (55.82, 56.47)	64.57 (64.05, 65.09)	70.33 (69.52, 71.15)	83.27 (81.15, 85.39)
13	44	25.62 (24.75, 26.50)	32.89 (32.47, 33.31)	36.33 (35.98, 36.68)	41.86 (41.56, 42.17)	48.74 (48.45, 49.03)	57.06 (56.69, 57.43)	65.80 (65.19, 66.42)	71.77 (70.82, 72.72)	85.12 (82.74, 87.50)
14	46	25.65 (24.75, 26.55)	33.08 (32.65, 33.52)	36.61 (36.26, 36.96)	42.33 (42.03, 42.62)	49.46 (49.15, 49.76)	58.09 (57.67, 58.51)	67.15 (66.47, 67.83)	73.32 (72.30, 74.33)	87.04 (84.66, 89.43)
15	48	25.79 (24.85, 26.72)	33.37 (32.89, 33.84)	36.99 (36.62, 37.36)	42.88 (42.58, 43.18)	50.27 (49.94, 50.59)	59.21 (58.74, 59.67)	68.56 (67.83, 69.30)	74.91 (73.89, 75.94)	88.95 (86.75, 91.16)
16	50	26.01 (25.03, 27.00)	33.73 (33.21, 34.26)	37.45 (37.05, 37.84)	43.51 (43.21, 43.82)	51.15 (50.81, 51.48)	60.38 (59.88, 60.89)	70.01 (69.23, 70.79)	76.51 (75.47, 77.55)	90.80 (88.81, 92.78)
17	52	26.31 (25.27, 27.35)	34.16 (33.59, 34.73)	37.97 (37.55, 38.39)	44.22 (43.91, 44.52)	52.09 (51.76, 52.43)	61.61 (61.08, 62.14)	71.48 (70.66, 72.30)	78.12 (77.06, 79.17)	92.58 (90.77, 94.38)
18	54	26.65 (25.59, 27.71)	34.64 (34.03, 35.24)	38.54 (38.09, 38.99)	44.97 (44.65, 45.29)	53.09 (52.76, 53.42)	62.87 (62.34, 63.40)	72.98 (72.15, 73.80)	79.72 (78.67, 80.78)	94.31 (92.64, 95.97)
19	56	27.01 (25.99, 28.03)	35.14 (34.54, 35.74)	39.15 (38.69, 39.60)	45.77 (45.43, 46.11)	54.13 (53.79, 54.46)	64.17 (63.64, 64.69)	74.48 (73.66, 75.30)	81.33 (80.30, 82.36)	96.00 (94.44, 97.56)
20	58	27.38 (26.43, 28.33)	35.66 (35.10, 36.23)	39.78 (39.33, 40.22)	46.60 (46.24, 46.96)	55.20 (54.85, 55.55)	65.49 (64.97, 66.00)	76.00 (75.21, 76.79)	82.94 (81.94, 83.93)	97.66 (96.17, 99.16)
21	60	27.76 (26.88, 28.63)	36.21 (35.68, 36.73)	40.43 (40.00, 40.87)	47.47 (47.10, 47.84)	56.31 (55.94, 56.68)	66.84 (66.34, 67.35)	77.55 (76.81, 78.28)	84.57 (83.65, 85.49)	99.34 (97.93, 100.75)
22	62	28.14 (27.32, 28.96)	36.78 (36.28, 37.28)	41.12 (40.70, 41.55)	48.38 (47.99, 48.77)	57.46 (57.08, 57.85)	68.24 (67.75, 68.73)	79.13 (78.45, 79.80)	86.23 (85.40, 87.05)	101.05 (99.76, 102.35)

	Age	1st	5th	10th	25th	50th	75th	90th	95th	99th
23	64	28.54 (27.74, 29.34)	37.37 (36.88, 37.86)	41.84 (41.42, 42.26)	49.31 (48.92, 49.70)	58.64 (58.24, 59.03)	69.64 (69.16, 70.12)	80.70 (80.07, 81.33)	87.87 (87.12, 88.63)	102.75 (101.55, 103.94)
24	66	28.96 (28.17, 29.76)	37.99 (37.51, 38.47)	42.58 (42.17, 42.98)	50.26 (49.88, 50.63)	59.80 (59.42, 60.19)	71.01 (70.55, 71.47)	82.22 (81.64, 82.81)	89.47 (88.78, 90.15)	104.37 (103.28, 105.46)
25	68	29.38 (28.58, 30.19)	38.60 (38.12, 39.07)	43.30 (42.91, 43.69)	51.19 (50.84, 51.54)	60.95 (60.58, 61.31)	72.35 (71.92, 72.79)	83.71 (83.16, 84.26)	91.01 (90.37, 91.65)	105.95 (104.93, 106.96)
26	70	29.75 (28.92, 30.58)	39.16 (38.68, 39.65)	43.99 (43.59, 44.39)	52.08 (51.73, 52.44)	62.05 (61.68, 62.42)	73.65 (73.20, 74.10)	85.15 (84.59, 85.72)	92.52 (91.85, 93.18)	107.50 (106.43, 108.58)
27	72	30.01 (29.12, 30.89)	39.63 (39.10, 40.16)	44.57 (44.13, 45.02)	52.88 (52.47, 53.30)	63.07 (62.64, 63.51)	74.88 (74.37, 75.38)	86.54 (85.90, 87.18)	93.98 (93.22, 94.74)	109.05 (107.74, 110.35)
28	74	30.08 (29.12, 31.04)	39.92 (39.34, 40.51)	45.00 (44.50, 45.50)	53.53 (53.06, 54.01)	63.96 (63.47, 64.46)	75.99 (75.42, 76.56)	87.84 (87.11, 88.56)	95.37 (94.48, 96.26)	110.55 (108.98, 112.12)
29	76	29.93 (28.92, 30.95)	40.01 (39.37, 40.65)	45.22 (44.68, 45.76)	53.99 (53.50, 54.47)	64.66 (64.15, 65.17)	76.93 (76.33, 77.53)	88.97 (88.19, 89.74)	96.60 (95.65, 97.56)	111.93 (110.29, 113.57)
30	78	29.60 (28.51, 30.70)	39.90 (39.18, 40.63)	45.25 (44.65, 45.85)	54.24 (53.75, 54.73)	65.16 (64.65, 65.66)	77.67 (77.05, 78.28)	89.90 (89.10, 90.71)	97.65 (96.65, 98.64)	113.13 (111.50, 114.76)
31	80	29.18 (27.99, 30.36)	39.69 (38.87, 40.50)	45.15 (44.48, 45.83)	54.35 (53.83, 54.88)	65.51 (65.00, 66.03)	78.26 (77.64, 78.87)	90.69 (89.88, 91.50)	98.54 (97.55, 99.53)	114.18 (112.57, 115.79)
32	82	28.73 (27.53, 29.93)	39.43 (38.58, 40.28)	45.01 (44.30, 45.71)	54.40 (53.86, 54.95)	65.79 (65.28, 66.29)	78.75 (78.17, 79.34)	91.38 (90.63, 92.13)	99.33 (98.41, 100.25)	115.12 (113.58, 116.66)
33	84	28.31 (27.16, 29.47)	39.18 (38.36, 40.01)	44.86 (44.17, 45.55)	54.44 (53.91, 54.98)	66.03 (65.55, 66.51)	79.21 (78.67, 79.75)	92.02 (91.31, 92.72)	100.06 (99.19, 100.93)	116.00 (114.50, 117.51)
34	86	27.92 (26.76, 29.07)	38.94 (38.14, 39.74)	44.72 (44.04, 45.39)	54.46 (53.93, 55.00)	66.25 (65.76, 66.74)	79.62 (79.06, 80.19)	92.60 (91.85, 93.35)	100.73 (99.80, 101.67)	116.81 (115.23, 118.39)
35	88	27.50 (26.22, 28.78)	38.67 (37.81, 39.53)	44.53 (43.81, 45.26)	54.44 (53.84, 55.05)	66.41 (65.84, 66.98)	79.97 (79.31, 80.62)	93.09 (92.22, 93.96)	101.31 (100.24, 102.38)	117.50 (115.72, 119.27)
36	90	27.00 (25.48, 28.51)	38.33 (37.33, 39.33)	44.28 (43.44, 45.12)	54.35 (53.63, 55.07)	66.49 (65.80, 67.18)	80.22 (79.47, 80.96)	93.48 (92.50, 94.46)	101.76 (100.54, 102.98)	118.05 (116.04, 120.07)

Wong D, *et al. BMJ Open* 2020; 10:e033618. doi: 10.1136/bmjopen-2019-033618

Appendix B – post-hoc analysis: centiles generate from population including readmissions

Male systolic blood pressure centiles

	Age	_1st	_5th	_10th	_25th	_50th	_75th	_90th	_95th	_99th
1	20	95.90	103.09	107.0	113.8	121.7	130.2	138.5	143.8	154.7
2	22	95.90	103.15	107.1	114.0	122.1	130.8	139.4	144.9	156.3
3	24	95.89	103.21	107.2	114.2	122.4	131.4	140.3	146.0	158.0
4	26	95.88	103.26	107.3	114.4	122.8	132.0	141.1	147.1	159.7
5	28	95.85	103.30	107.4	114.6	123.1	132.5	142.0	148.2	161.5
6	30	95.82	103.33	107.5	114.8	123.5	133.1	142.9	149.3	163.2
7	32	95.77	103.36	107.6	114.9	123.8	133.7	143.7	150.5	165.0
8	34	95.71	103.38	107.6	115.1	124.1	134.2	144.6	151.6	166.8
9	36	95.62	103.38	107.7	115.3	124.5	134.8	145.5	152.7	168.7
10	38	95.50	103.37	107.7	115.4	124.8	135.4	146.4	153.9	170.6
11	40	95.34	103.34	107.8	115.6	125.1	135.9	147.3	155.1	172.6
12	42	95.14	103.28	107.8	115.8	125.4	136.5	148.1	156.2	174.5
13	44	94.89	103.20	107.8	115.9	125.8	137.0	149.0	157.3	176.3
14	46	94.65	103.11	107.8	116.0	126.1	137.6	149.8	158.3	177.8
15	48	94.42	103.03	107.8	116.2	126.4	138.1	150.5	159.2	179.1
16	50	94.23	102.95	107.8	116.3	126.7	138.6	151.2	159.9	179.9
17	52	94.09	102.89	107.8	116.4	127.0	139.1	151.8	160.6	180.5
18	54	93.97	102.83	107.8	116.6	127.3	139.5	152.4	161.2	180.8
19	56	93.85	102.77	107.8	116.7	127.6	140.0	152.9	161.7	181.1
20	58	93.71	102.69	107.8	116.8	127.9	140.4	153.4	162.2	181.3
21	60	93.55	102.60	107.7	116.9	128.2	140.9	154.0	162.8	181.7
22	62	93.36	102.49	107.7	117.0	128.4	141.3	154.5	163.3	182.0
23	64	93.13	102.36	107.6	117.1	128.7	141.8	155.1	163.9	182.5
24	66	92.88	102.22	107.6	117.2	129.0	142.2	155.6	164.5	183.0
25	68	92.60	102.07	107.5	117.3	129.2	142.7	156.2	165.1	183.5
26	70	92.30	101.90	107.4	117.3	129.5	143.1	156.8	165.7	184.2
27	72	91.97	101.71	107.3	117.4	129.8	143.5	157.3	166.3	184.9
28	74	91.63	101.52	107.2	117.5	130.0	144.0	157.9	167.0	185.6
29	76	91.27	101.32	107.1	117.5	130.2	144.4	158.5	167.7	186.4
30	78	90.90	101.11	107.0	117.5	130.5	144.8	159.1	168.3	187.2
31	80	90.52	100.89	106.9	117.6	130.7	145.3	159.7	169.0	188.0
32	82	90.13	100.66	106.7	117.6	131.0	145.7	160.3	169.7	188.8
33	84	89.73	100.43	106.6	117.7	131.2	146.1	160.9	170.4	189.7
34	86	89.33	100.19	106.5	117.7	131.4	146.6	161.5	171.1	190.6
35	88	88.91	99.95	106.3	117.7	131.6	147.0	162.1	171.8	191.4
36	90	88.48	99.70	106.2	117.7	131.9	147.4	162.7	172.5	192.3

Wong D, *et al. BMJ Open* 2020; 10:e033618. doi: 10.1136/bmjopen-2019-033618

Female systolic blood pressure centiles

	Age	_1st	_5th	_10th	_25th	_50th	_75th	_90th	_95th	_99th
1	20	88.93	95.90	99.64	106.1	113.7	122.3	131.3	137.5	151.8
2	22	89.28	96.03	99.71	106.1	113.8	122.5	131.7	138.0	152.5
3	24	89.51	96.09	99.72	106.1	113.9	122.7	132.0	138.5	153.2
4	26	89.58	96.03	99.63	106.0	113.8	122.8	132.3	138.9	154.0
5	28	89.51	95.87	99.45	105.8	113.8	122.9	132.6	139.4	154.9
6	30	89.39	95.70	99.28	105.7	113.8	123.1	133.1	140.1	156.1
7	32	89.30	95.61	99.22	105.7	113.9	123.5	133.8	141.0	157.6
8	34	89.30	95.64	99.29	105.9	114.3	124.2	134.8	142.3	159.5
9	36	89.37	95.79	99.50	106.3	114.9	125.0	136.1	143.8	161.7
10	38	89.50	96.03	99.83	106.8	115.6	126.1	137.5	145.6	164.1
11	40	89.69	96.36	100.25	107.4	116.5	127.4	139.1	147.4	166.6
12	42	89.93	96.76	100.75	108.1	117.5	128.7	140.8	149.4	169.1
13	44	90.20	97.20	101.30	108.9	118.5	130.1	142.6	151.4	171.4
14	46	90.48	97.67	101.88	109.6	119.6	131.4	144.2	153.2	173.6
15	48	90.76	98.13	102.46	110.4	120.7	132.8	145.8	154.9	175.5
16	50	91.02	98.58	103.02	111.2	121.7	134.0	147.3	156.5	177.1
17	52	91.26	99.01	103.56	111.9	122.6	135.2	148.7	157.9	178.6
18	54	91.49	99.44	104.11	112.7	123.6	136.4	150.0	159.3	179.8
19	56	91.70	99.87	104.65	113.4	124.6	137.6	151.3	160.6	181.0
20	58	91.91	100.29	105.19	114.2	125.5	138.7	152.5	161.9	182.2
21	60	92.12	100.71	105.72	114.9	126.5	139.8	153.7	163.1	183.3
22	62	92.32	101.12	106.25	115.6	127.4	140.9	155.0	164.4	184.5
23	64	92.52	101.52	106.76	116.3	128.3	142.0	156.2	165.6	185.7
24	66	92.70	101.90	107.25	117.0	129.2	143.1	157.3	166.8	186.9
25	68	92.88	102.27	107.72	117.6	130.0	144.1	158.5	168.0	188.1
26	70	93.03	102.60	108.16	118.3	130.8	145.1	159.6	169.2	189.3
27	72	93.15	102.91	108.57	118.8	131.6	146.0	160.7	170.3	190.5
28	74	93.25	103.17	108.93	119.4	132.3	146.9	161.7	171.4	191.6
29	76	93.31	103.39	109.24	119.8	132.9	147.7	162.6	172.4	192.6
30	78	93.33	103.56	109.49	120.2	133.5	148.4	163.4	173.3	193.6
31	80	93.31	103.68	109.68	120.5	134.0	149.0	164.2	174.1	194.5
32	82	93.27	103.76	109.83	120.8	134.4	149.6	164.8	174.8	195.3
33	84	93.21	103.81	109.94	121.0	134.7	150.0	165.4	175.4	196.0
34	86	93.13	103.83	110.02	121.2	135.0	150.4	165.9	176.0	196.7
35	88	93.05	103.84	110.08	121.4	135.3	150.8	166.4	176.5	197.3
36	90	92.96	103.83	110.12	121.5	135.5	151.1	166.8	177.0	197.9

Wong D, *et al. BMJ Open* 2020; 10:e033618. doi: 10.1136/bmjopen-2019-033618

Male diastolic blood pressure centiles

	Age	_1st	_5th	_10th	_25th	_50th	_75th	_90th	_95th	_99th
1	20	45.48	51.54	54.57	59.58	65.58	72.48	79.34	83.77	92.94
2	22	45.79	52.07	55.23	60.42	66.66	73.81	80.94	85.54	95.06
3	24	46.15	52.65	55.91	61.28	67.73	75.13	82.49	87.25	97.09
4	26	46.55	53.24	56.60	62.14	68.78	76.40	83.99	88.89	99.03
5	28	46.99	53.86	57.30	62.99	69.80	77.62	85.40	90.43	100.83
6	30	47.45	54.47	57.99	63.80	70.77	78.76	86.72	91.86	102.50
7	32	47.92	55.08	58.66	64.58	71.67	79.82	87.92	93.16	103.99
8	34	48.40	55.66	59.30	65.31	72.51	80.78	89.01	94.32	105.32
9	36	48.86	56.21	59.90	65.98	73.26	81.63	89.96	95.34	106.47
10	38	49.31	56.72	60.44	66.58	73.93	82.37	90.77	96.20	107.43
11	40	49.73	57.19	60.93	67.10	74.50	82.99	91.45	96.91	108.20
12	42	50.11	57.59	61.35	67.54	74.96	83.49	91.97	97.46	108.79
13	44	50.44	57.93	61.69	67.90	75.33	83.87	92.36	97.85	109.20
14	46	50.71	58.20	61.96	68.16	75.58	84.12	92.60	98.09	109.43
15	48	50.92	58.40	62.15	68.33	75.74	84.25	92.71	98.18	109.50
16	50	51.07	58.51	62.25	68.40	75.78	84.26	92.69	98.14	109.41
17	52	51.14	58.54	62.26	68.38	75.72	84.15	92.54	97.96	109.17
18	54	51.14	58.50	62.19	68.27	75.56	83.94	92.27	97.66	108.80
19	56	51.07	58.37	62.03	68.07	75.31	83.63	91.90	97.25	108.31
20	58	50.91	58.16	61.79	67.79	74.97	83.22	91.44	96.74	107.71
21	60	50.68	57.87	61.48	67.43	74.55	82.74	90.88	96.15	107.03
22	62	50.38	57.52	61.09	66.99	74.06	82.18	90.26	95.49	106.28
23	64	50.02	57.09	60.64	66.50	73.51	81.57	89.59	94.77	105.48
24	66	49.59	56.61	60.14	65.95	72.91	80.91	88.87	94.02	104.65
25	68	49.11	56.09	59.59	65.36	72.28	80.23	88.14	93.25	103.81
26	70	48.58	55.52	59.00	64.74	71.63	79.53	87.39	92.47	102.98
27	72	48.02	54.93	58.40	64.11	70.96	78.84	86.67	91.73	102.19
28	74	47.43	54.32	57.78	63.48	70.32	78.16	85.97	91.02	101.46
29	76	46.84	53.72	57.17	62.87	69.70	77.54	85.34	90.38	100.81
30	78	46.24	53.13	56.59	62.29	69.13	76.97	84.78	89.83	100.27
31	80	45.66	52.58	56.05	61.77	68.62	76.50	84.33	89.40	99.87
32	82	45.12	52.08	55.57	61.32	68.21	76.14	84.02	89.11	99.64
33	84	44.63	51.64	55.16	60.96	67.92	75.91	83.86	88.99	99.61
34	86	44.20	51.30	54.86	60.73	67.76	75.84	83.88	89.08	99.82
35	88	43.86	51.06	54.67	60.63	67.77	75.97	84.13	89.41	100.31
36	90	43.62	50.95	54.63	60.70	67.97	76.32	84.63	90.00	101.11

Wong D, *et al. BMJ Open* 2020; 10:e033618. doi: 10.1136/bmjopen-2019-033618

Female diastolic blood pressure centiles

	Age	_1st	_5th	_10th	_25th	_50th	_75th	_90th	_95th	_99th
1	20	45.26	50.97	53.83	58.62	64.67	71.90	79.25	84.06	94.18
2	22	45.33	51.15	54.07	58.94	65.11	72.49	79.98	84.88	95.19
3	24	45.41	51.34	54.31	59.28	65.56	73.07	80.70	85.70	96.19
4	26	45.50	51.53	54.56	59.61	66.00	73.64	81.40	86.49	97.17
5	28	45.61	51.73	54.80	59.94	66.44	74.20	82.08	87.25	98.10
6	30	45.72	51.93	55.05	60.26	66.85	74.73	82.73	87.97	98.98
7	32	45.84	52.13	55.29	60.57	67.25	75.22	83.33	88.64	99.80
8	34	45.96	52.33	55.52	60.86	67.62	75.69	83.89	89.26	100.54
9	36	46.09	52.52	55.74	61.14	67.96	76.10	84.39	89.81	101.21
10	38	46.21	52.70	55.95	61.39	68.26	76.48	84.83	90.30	101.79
11	40	46.34	52.87	56.14	61.61	68.53	76.80	85.21	90.71	102.28
12	42	46.46	53.02	56.31	61.81	68.77	77.08	85.52	91.06	102.68
13	44	46.58	53.16	56.46	61.98	68.96	77.30	85.77	91.32	102.98
14	46	46.69	53.28	56.59	62.12	69.11	77.47	85.96	91.52	103.20
15	48	46.79	53.38	56.70	62.23	69.22	77.59	86.08	91.65	103.34
16	50	46.87	53.47	56.78	62.30	69.30	77.65	86.14	91.71	103.39
17	52	46.94	53.53	56.83	62.35	69.33	77.67	86.15	91.71	103.37
18	54	47.00	53.56	56.86	62.36	69.33	77.65	86.10	91.64	103.28
19	56	47.03	53.57	56.86	62.35	69.29	77.58	86.01	91.53	103.13
20	58	47.04	53.56	56.83	62.30	69.22	77.48	85.88	91.38	102.93
21	60	47.03	53.52	56.78	62.23	69.12	77.34	85.71	91.19	102.69
22	62	46.99	53.46	56.71	62.13	68.99	77.18	85.51	90.97	102.43
23	64	46.93	53.37	56.61	62.01	68.84	77.00	85.30	90.74	102.15
24	66	46.84	53.26	56.48	61.87	68.68	76.81	85.08	90.50	101.87
25	68	46.72	53.13	56.34	61.71	68.50	76.61	84.86	90.26	101.61
26	70	46.58	52.97	56.18	61.54	68.32	76.42	84.65	90.04	101.37
27	72	46.40	52.79	56.00	61.36	68.14	76.24	84.47	89.86	101.18
28	74	46.20	52.60	55.81	61.18	67.96	76.07	84.32	89.72	101.06
29	76	45.97	52.39	55.61	61.00	67.81	75.94	84.21	89.63	101.01
30	78	45.71	52.17	55.41	60.82	67.67	75.86	84.17	89.62	101.07
31	80	45.42	51.93	55.20	60.66	67.57	75.82	84.21	89.71	101.25
32	82	45.09	51.69	54.99	60.52	67.51	75.86	84.35	89.91	101.58
33	84	44.74	51.43	54.79	60.40	67.50	75.98	84.59	90.24	102.09
34	86	44.36	51.18	54.60	60.31	67.55	76.19	84.97	90.72	102.80
35	88	43.93	50.91	54.41	60.27	67.67	76.51	85.50	91.39	103.76
36	90	43.46	50.64	54.25	60.26	67.88	76.97	86.22	92.27	104.99

Wong D, *et al. BMJ Open* 2020; 10:e033618. doi: 10.1136/bmjopen-2019-033618

Male Pulse Pressure Centiles

	Age	_1st	_5th	_10th	_25th	_50th	_75th	_90th	_95th	_99th
1	20	31.79	39.16	42.78	48.61	55.26	62.63	69.90	74.61	84.46
2	22	31.58	38.85	42.41	48.14	54.72	62.07	69.35	74.10	84.06
3	24	31.31	38.48	41.98	47.61	54.12	61.44	68.75	73.53	83.62
4	26	30.97	38.04	41.48	47.01	53.45	60.76	68.09	72.91	83.14
5	28	30.57	37.55	40.93	46.37	52.75	60.04	67.40	72.26	82.65
6	30	30.14	37.03	40.36	45.71	52.03	59.33	66.73	71.64	82.20
7	32	29.68	36.50	39.78	45.06	51.34	58.65	66.11	71.08	81.84
8	34	29.23	36.00	39.24	44.46	50.72	58.06	65.59	70.64	81.63
9	36	28.82	35.56	38.78	43.95	50.21	57.60	65.23	70.36	81.63
10	38	28.45	35.19	38.39	43.55	49.82	57.29	65.04	70.29	81.86
11	40	28.10	34.87	38.07	43.23	49.55	57.13	65.03	70.40	82.33
12	42	27.73	34.56	37.79	42.98	49.38	57.10	65.19	70.71	83.03
13	44	27.36	34.28	37.54	42.79	49.31	57.21	65.51	71.20	83.94
14	46	26.99	34.03	37.34	42.68	49.35	57.46	66.01	71.86	85.03
15	48	26.67	33.83	37.21	42.67	49.52	57.86	66.67	72.70	86.27
16	50	26.43	33.72	37.17	42.77	49.83	58.43	67.51	73.72	87.63
17	52	26.28	33.70	37.24	43.00	50.29	59.18	68.53	74.90	89.09
18	54	26.26	33.80	37.42	43.35	50.89	60.07	69.68	76.19	90.55
19	56	26.35	33.98	37.69	43.80	51.59	61.05	70.89	77.51	91.92
20	58	26.47	34.18	37.98	44.28	52.32	62.05	72.10	78.79	93.17
21	60	26.58	34.36	38.25	44.74	53.03	63.02	73.25	79.99	94.27
22	62	26.72	34.58	38.55	45.24	53.78	64.02	74.41	81.19	95.34
23	64	26.94	34.89	38.96	45.84	54.63	65.11	75.65	82.47	96.50
24	66	27.25	35.30	39.46	46.55	55.58	66.30	77.00	83.86	97.80
25	68	27.60	35.78	40.05	47.35	56.62	67.58	78.44	85.36	99.25
26	70	27.90	36.23	40.61	48.13	57.65	68.84	79.88	86.85	100.74
27	72	28.06	36.57	41.07	48.80	58.57	70.00	81.21	88.25	102.17
28	74	28.02	36.72	41.35	49.31	59.33	71.01	82.40	89.53	103.52
29	76	27.78	36.69	41.45	49.65	59.93	71.85	83.44	90.66	104.77
30	78	27.42	36.54	41.42	49.84	60.38	72.55	84.34	91.67	105.91
31	80	27.06	36.36	41.36	49.98	60.75	73.16	85.15	92.59	106.99
32	82	26.81	36.26	41.35	50.15	61.12	73.75	85.93	93.47	108.03
33	84	26.71	36.27	41.43	50.35	61.50	74.34	86.70	94.34	109.05
34	86	26.71	36.35	41.56	50.59	61.89	74.89	87.42	95.15	110.01
35	88	26.78	36.46	41.70	50.80	62.21	75.36	88.01	95.82	110.80
36	90	26.89	36.58	41.84	50.98	62.47	75.73	88.48	96.34	111.41

Wong D, *et al. BMJ Open* 2020; 10:e033618. doi: 10.1136/bmjopen-2019-033618

Female pulse pressure centiles

	Age	_1st	_5th	_10th	_25th	_50th	_75th	_90th	_95th	_99th
1	20	27.36	33.96	37.05	41.94	47.74	54.55	61.63	66.45	77.21
2	22	27.16	33.71	36.78	41.62	47.39	54.17	61.24	66.05	76.83
3	24	26.95	33.47	36.51	41.32	47.06	53.82	60.89	65.71	76.53
4	26	26.76	33.25	36.27	41.06	46.78	53.55	60.63	65.47	76.34
5	28	26.60	33.07	36.08	40.86	46.59	53.38	60.49	65.36	76.32
6	30	26.48	32.95	35.97	40.75	46.50	53.35	60.53	65.45	76.53
7	32	26.40	32.90	35.93	40.74	46.55	53.49	60.77	65.77	77.02
8	34	26.32	32.88	35.93	40.80	46.70	53.77	61.21	66.31	77.80
9	36	26.21	32.85	35.95	40.90	46.94	54.19	61.82	67.05	78.85
10	38	26.06	32.82	35.99	41.05	47.25	54.73	62.60	68.00	80.16
11	40	25.89	32.80	36.04	41.24	47.65	55.40	63.55	69.14	81.71
12	42	25.75	32.82	36.15	41.51	48.15	56.19	64.66	70.45	83.44
13	44	25.69	32.92	36.34	41.87	48.76	57.11	65.89	71.89	85.28
14	46	25.73	33.11	36.63	42.33	49.48	58.14	67.23	73.41	87.15
15	48	25.89	33.41	37.01	42.89	50.28	59.25	68.62	74.97	88.98
16	50	26.14	33.79	37.48	43.52	51.16	60.41	70.05	76.54	90.76
17	52	26.45	34.21	38.00	44.22	52.10	61.63	71.50	78.12	92.49
18	54	26.78	34.68	38.56	44.97	53.09	62.88	72.98	79.71	94.19
19	56	27.14	35.18	39.16	45.76	54.13	64.18	74.49	81.32	95.89
20	58	27.51	35.71	39.79	46.60	55.21	65.52	76.04	82.96	97.60
21	60	27.91	36.27	40.47	47.49	56.34	66.90	77.62	84.63	99.34
22	62	28.33	36.87	41.18	48.41	57.51	68.31	79.22	86.32	101.09
23	64	28.76	37.49	41.92	49.36	58.69	69.73	80.81	87.99	102.82
24	66	29.20	38.11	42.66	50.31	59.86	71.12	82.37	89.62	104.51
25	68	29.60	38.71	43.38	51.23	61.01	72.47	83.88	91.21	106.16
26	70	29.92	39.23	44.02	52.09	62.09	73.76	85.34	92.75	107.79
27	72	30.11	39.65	44.57	52.85	63.09	74.98	86.74	94.23	109.38
28	74	30.14	39.91	44.96	53.48	63.96	76.08	88.03	95.63	110.91
29	76	29.98	39.99	45.18	53.93	64.66	77.02	89.17	96.86	112.30
30	78	29.66	39.91	45.23	54.21	65.18	77.78	90.11	97.91	113.50
31	80	29.24	39.71	45.16	54.36	65.56	78.38	90.90	98.80	114.53
32	82	28.77	39.45	45.02	54.43	65.84	78.87	91.56	99.55	115.42
33	84	28.31	39.18	44.86	54.45	66.07	79.30	92.15	100.23	116.22
34	86	27.85	38.91	44.69	54.46	66.28	79.69	92.69	100.84	116.95
35	88	27.40	38.62	44.51	54.45	66.44	80.02	93.16	101.39	117.60
36	90	26.92	38.31	44.29	54.40	66.57	80.30	93.57	101.86	118.16

Appendix C - Diastolic and Pulse Pressure Centiles for Emergency/Elective cohorts

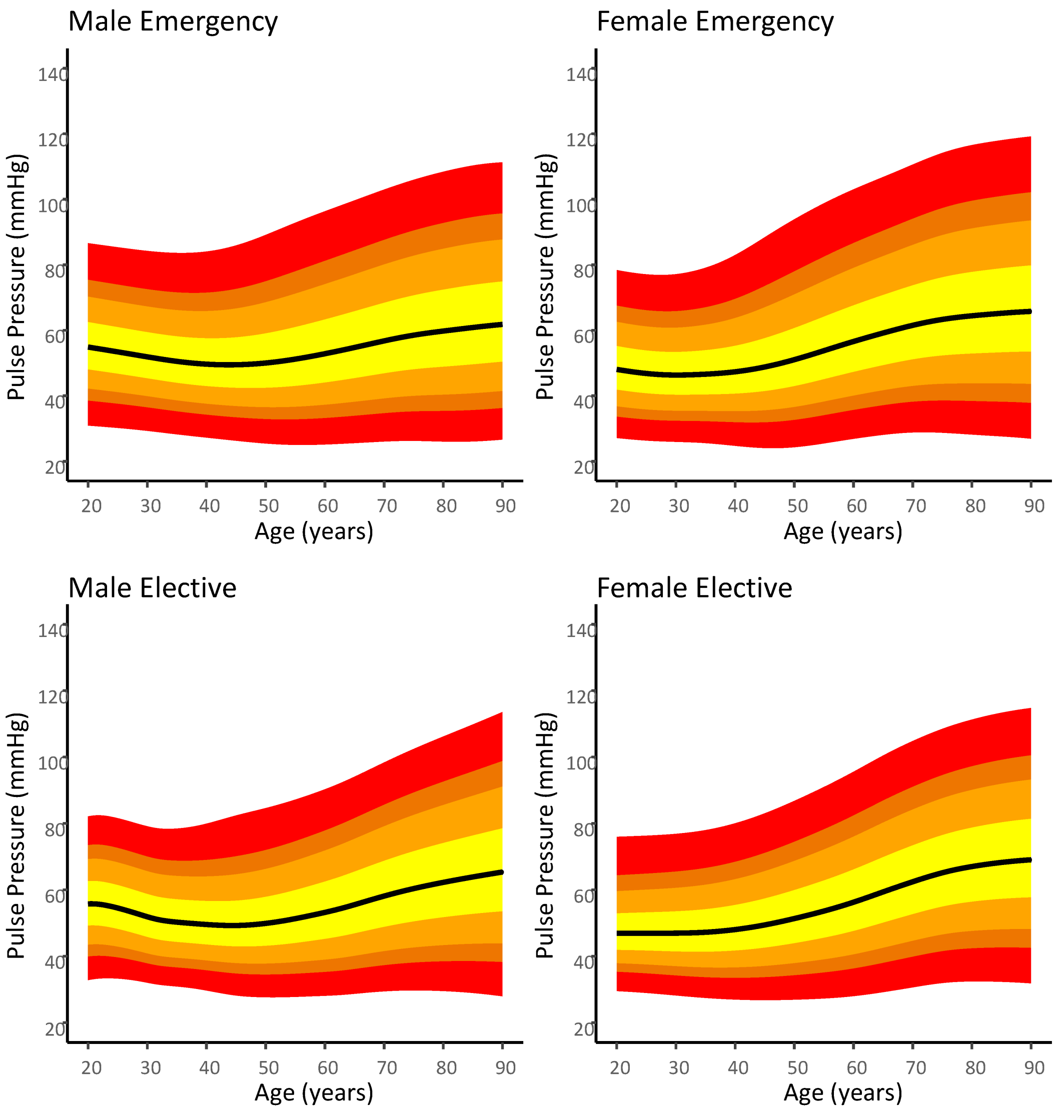

Wong D, *et al. BMJ Open* 2020; 10:e033618. doi: 10.1136/bmjopen-2019-033618

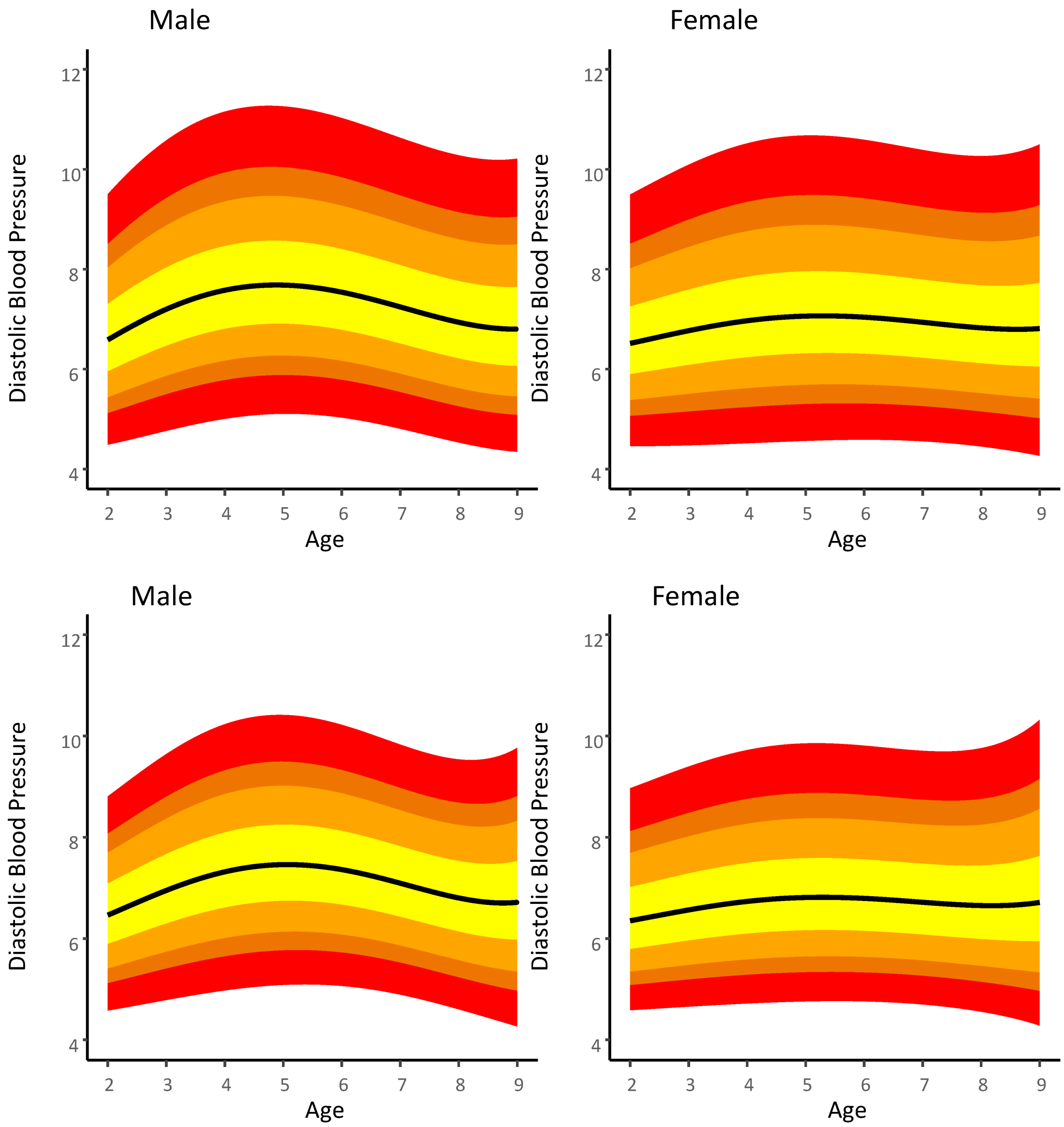

Wong D, *et al. BMJ Open* 2020; 10:e033618. doi: 10.1136/bmjopen-2019-033618
