# Supplementary material for: Cross-sectional centiles of blood pressure by age and sex: a four-hospital database retrospective observational analysis
Source: BMJ Open. 2020 May 5;10(5):e033618. doi: 10.1136/bmjopen-2019-033618 (PMC7223140; doi:10.1136/bmjopen-2019-033618)
Supplement: Author's manuscript [file bmjopen-2019-033618.draft_revisions.pdf]

BMJ Open

BMJ Open is committed to open peer review. As part of this commitment we make the peer review history of every article we publish publicly available.

When an article is published we post the peer reviewers' comments and the authors' responses online. We also post the versions of the paper that were used during peer review. These are the versions that the peer review comments apply to.

The versions of the paper that follow are the versions that were submitted during the peer review process. They are not the versions of record or the final published versions. They should not be cited or distributed as the published version of this manuscript.

BMJ Open is an open access journal and the full, final, typeset and author-corrected version of record of the manuscript is available on our site with no access controls, subscription charges or pay-per-view fees (http://bmjopen.bmj.com).

If you have any questions on BMJ Open's open peer review process please email info.bmjopen@bmj.com

BMJ Open

Cross-Sectional Centiles of Blood Pressure by Age and Sex: a four-hospital database retrospective observational analysis.

Journal:	*BMJ Open*
Manuscript ID	bmjopen-2019-033618
Article Type:	Research
Date Submitted by the Author:	13-Aug-2019

Complete List of Authors:	Wong, David; The University of Manchester, Centre for Health Informatics<br>Gerry, Stephen; University of Oxford, Centre for Statistics in Medicine<br>Pimentel, Marco; University of Oxford,<br>Shamout, Farah; University of Oxford, Institute of Biomedical Engineering<br>Clifton, David; University of Oxford, Engineering Science<br>Watkinson, Peter; Oxford University Hospitals NHS Trust, Kadoorie Centre for Critical Care research and Education

Keywords:	blood pressure, hospitals, ageing, Physiology < BASIC SCIENCES

SCHOLARONE™
Manuscripts

I, the Submitting Author has the right to grant and does grant on behalf of all authors of the Work (as defined in the below author licence), an exclusive licence and/or a non-exclusive licence for contributions from authors who are: i) UK Crown employees; ii) where BMJ has agreed a CC-BY licence shall apply, and/or iii) in accordance with the terms applicable for US Federal Government officers or employees acting as part of their official duties; on a worldwide, perpetual, irrevocable, royalty-free basis to BMJ Publishing Group Ltd ("BMJ") its licensees and where the relevant Journal is co-owned by BMJ to the co-owners of the Journal, to publish the Work in this journal and any other BMJ products and to exploit all rights, as set out in our licence.

The Submitting Author accepts and understands that any supply made under these terms is made by BMJ to the Submitting Author unless you are acting as an employee on behalf of your employer or a postgraduate student of an affiliated institution which is paying any applicable article publishing charge ("APC") for Open Access articles. Where the Submitting Author wishes to make the Work available on an Open Access basis (and intends to pay the relevant APC), the terms of reuse of such Open Access shall be governed by a Creative Commons licence – details of these licences and which Creative Commons licence will apply to this Work are set out in our licence referred to above.

Other than as permitted in any relevant BMJ Author's Self Archiving Policies, I confirm this Work has not been accepted for publication elsewhere, is not being considered for publication elsewhere and does not duplicate material already published. I confirm all authors consent to publication of this Work and authorise the granting of this licence.

For peer review only

Cross-Sectional Centiles of Blood Pressure by Age and Sex: a four-hospital database retrospective observational analysis.

David C Wong – corresponding author
Centre for Health Informatics
Vaughan House
University of Manchester,
Manchester,
UK,
M13 9PR
email: david.wong@manchester.ac.uk

Stephen Gerry
Centre for Statistics in Medicine
Nuffield Department of Orthopaedics, Rheumatology and Musculoskeletal Sciences,
University of Oxford,
Oxford,
UK,
OX1 4AJ
email: stephen.gerry@csm.ox.ac.uk

Farah Shamout
Institute of Biomedical Engineering,
Old Road Campus Research Building,
Old Road,
Oxford,
UK,
OX3 7DQ
email: farah.shamout@eng.ox.ac.uk

David A Clifton
Institute of Biomedical Engineering,
Old Road Campus Research Building,
Old Road,
Oxford,

For peer review only

UK
OX3 7DQ
email: davidc@robots.ox.ac.uk

Marco Pimentel
Institute of Biomedical Engineering,
Old Road Campus Research Building,
Old Road,
Oxford,
UK
email: marco.pimentel@eng.ox.ac.uk

Peter Watkinson
Kadoorie Centre for Critical Care Research and Education,
Level 3, John Radcliffe Hospital,
Headley Way,
Oxford,
UK,
OX3 9DU
email: peter.watkinson@ndcn.ox.ac.uk

Abstract: 298 words

Manuscript: 2282 Words

For peer review only

Abstract

objectives: National guidelines for identifying physiological deterioration and sepsis in hospitals depend on thresholds for blood pressure that do not account for age or sex. In populations outside hospital, differences in blood pressure are known to occur with both variables. Whether these differences remain in the hospitalised population is unknown. This database analysis study aims to generate representative centiles to quantify variations in blood pressure by age and sex in hospitalised patients.

design: retrospective cross-sectional observational database analysis.

setting: four near-sea-level hospitals between April 2015 and April 2017

participants: 75342 adult patients who were admitted to the hospitals and had at least one set of documented vital sign observations within 24 hours before discharge were eligible for inclusion. Patients were excluded if they died in hospital, had no vital signs 24 hours prior to discharge, were readmitted within 7 days of discharge, had missing age or sex or had no blood pressure recorded.

results: Systolic blood pressure for hospitalised patients increases with age for both sexes. Median systolic blood pressure increases from 122 (CI: 121.1 – 122.1) mmHg to 132 (CI: 130.9 – 132.2) mmHg in men, and 114 (CI: 113.1-114.4) mmHg to 135 (CI: 134.5-136.2) mmHg for women, between the ages of 20 and 90. Diastolic blood pressure peaked around 50 years for men 76 (CI: 75.5-75.9)mmHg and women 69 (CI: 69.0-69.4) mmHg. The blood pressure criterion for sepsis, systolic < 100mmHg, was met by 2.3% of younger (20-30yrs) men and 3.5% of older men (81-90yrs). In comparison, the criterion was met by 9.7% of younger women and 2.6% of older women.

Conclusion: We have quantified variations in blood pressure by age and sex in hospitalised patients that have implications for recognition of deterioration. Nearly 10% of younger women met the blood pressure criterion for sepsis at hospital discharge

Key Words: Blood Pressure; Hospitals; Ageing; Physiology

For peer review only

Strengths and Limitations of this study

- Changes in Blood Pressure by age and sex are currently unknown for the hospitalised population
- A large cross-sectional database of 75342 patients were used to derive blood pressure centiles
- Results have implications on how sepsis and other in-hospital deterioration are detected in routine care
- Though patterns match those seen in out-of-hospital longitudinal studies, cross-sectional analysis can be affected by survival bias and birth cohort effects.

For peer review only

For peer review only - http://bmjopen.bmj.com/site/about/guidelines.xhtml

INTRODUCTION

Routine measurement of blood pressure is a key component of patient surveillance and diagnosis in hospitals worldwide. Currently, in-hospital assessment of blood pressure is undertaken by comparison to generic population normal ranges.

The ability to use an individual's physiology to monitor them for signs of deterioration may be improved by taking into account factors that affect these normal ranges.[1] For instance, in paediatric medicine, it is accepted that the normal ranges of vital signs vary with age and patients are managed in light of their age-specific normal ranges.[2-3] However, none of the published physiology-based systems for recognising deterioration in hospitalised adults take account of variations in vital signs by age or sex,[4] despite growing evidence that these factors may provide additional information for accurately identifying deteriration.[5-6] As examples, the National Early Warning Score (NEWS2)[7] scores blood pressure below 90 mmHg as requiring emergency attention and current sepsis guidelines blood pressure criterion are met when systolic blood pressure is less than 100 mmHg,[8] both regardless of age or sex.

In populations outside hospital, differences in blood pressure are known to occur with both age and sex.[9] If clinically significant differences also exist in hospitalised adult populations, opportunities for earlier identification and management of patient deterioration may be being missed.

To quantify these differences, our objective was to define representative centiles for blood pressure by age and sex via an analysis of a large near-sea-level database of routinely-collected vital signs.

For peer review only - http://bmjopen.bmj.com/site/about/guidelines.xhtml

METHODS

This paper is reported according to RECORD guidelines. Approval for obtaining the data used in this study was obtained from the Oxford Research Ethics Committee (REC ref: 16/SC/0264). We conducted a cross-sectional analysis from a database collated at Oxford University Hospitals (OUH) NHS foundation trust group of hospitals. The OUH consists of four hospitals: an urban teaching hospital, a general district hospital, and two specialist hospitals. Our data set included patients admitted to the OUH between April 2015 and April 2017.

All adult patients who were admitted to OUH and had at least one set of documented vital sign observations within 24 hours prior to discharge were eligible for inclusion. Patients were excluded from the analysis if they (1) died in hospital, (2) had no recorded vital signs 24 hours prior to discharge, (3) were readmitted within 7 days of discharge, (4) had missing recordings for age or sex or (5) had no blood pressure recorded.

We collected the final recorded set of blood pressure observations from a patient's first attendance to the OUH hospital group during the study period. This ensured that the centiles were not biased towards repeat attenders or patients with longer hospital stays. Blood pressure was measured using automated devices ratified for clinical use as part of routine clinical care and electronically documented using the SEND e-Obs software.[10] Data were validated for plausible range at the point of entry. Hospital admission time, discharge time, discharge status, ethnicity, Admission Method and Main Specialty were also collected for each patient from the hospital electronic patient record (Cerner Millennium, Cerner, Kansas City, MO). One investigator (PW) had access to a small proportion of the database population as part of routine clinical care responsibilities.

For peer review only - http://bmjopen.bmj.com/site/about/guidelines.xhtml

Admissions were typed as either Elective, Emergency or Other, according to Admission Method code. Codes are defined in full within the NHS data dictionary.[11] The set of ICD-10 codes {I10, I11, I12, I13, I14, I15} were used to determine patients with a primary diagnosis of hypertension.[12]

Analysis

The characteristics of the study population were described using medians and quartiles for the continuous variables, and frequencies otherwise. We calculated median and representative centiles (1st, 5th, 10th, 25th, 75th, 90th, 95th, 99th) for blood pressure at all ages between 20 and 90, for males and females. Systolic blood pressure (SBP) and diastolic blood pressure (DBP) are presented separately. One further measure, the pulse pressure (PP) was derived as the arithmetic difference between SBP and DBP and was also analysed using the same methods.

In subgroup analyses, we produced separate representative centiles by age and sex for Emergency and Elective Admissions, and for patients without a diagnostic code for hypertension.

Centiles were estimated using the Generalised Additive Models for Location, Scale, and Shape framework (GAMLSS).[13] This semi-parametric method provides various options for fitting non-normal distributions to the data. To create smooth centiles across the age range, penalised splines and fractional polynomials were used as smoothing functions. For each vital sign, we assessed six different distributions within the GAMLSS framework: Box-Cox Cole and Green, Box-Cox Power Exponential, Box-Cox-t, Skew Power Exponential type 3, Skew t type 3, and Power Exponential. The best fitting distribution was chosen based on a

For peer review only - http://bmjopen.bmj.com/site/about/guidelines.xhtml

combination of model fit (Akaike information criterion and Bayesian information criterion)[14-15] and a comparison of fitted versus empirical centiles. To ensure fair comparison, the same distribution was chosen for all subgroups within any given vital sign.

All analyses were undertaken using R and the GAMLSS package.[16]

Sample size

We used all the available data and therefore no formal sample size calculation was undertaken. To ensure that the sample was sufficient, the precision of the centiles was estimated via a bootstrapping procedure, whereby the dataset was sampled with replacement to create a new dataset and the analysis was carried out.[17] This was repeated 50 times. The standard deviation of these bootstrapped estimates was used to calculate the 95% confidence interval for each centile at two-yearly intervals. Full details of centile values and confidence intervals are provided in Appendix A.

For peer review only

RESULTS

83004 patients were admitted to the hospital trust during the period of study and received at least one observation on the SEND e-Obs system. In total, 75342 patients were included in the study. Blood pressure was missing in 885 (1.17%) records. Other reasons for exclusion are shown in Figure 1. Patient and hospital descriptors are shown in Table 1.

Blood pressure centiles

Centiles by age for SBP, DBP and PP are shown in Figure 2 for each sex. Figure 2a shows progressive increase in median SBP from 122 (CI: 121.1 – 122.1) mmHg to 132 (CI: 130.9 – 132.2) mmHg for males between the ages of 20 and 90 years. Younger females, had a lower

For peer review only - http://bmjopen.bmj.com/site/about/guidelines.xhtml

median SBP than younger males (114 (CI: 113.1-114.4) mmHg at age 20). By the age of 90, median SBP was higher for females than for males (135 (CI: 134.5-136.2) mmHg).

Table 1 - Characteristics of the study population

	**Female**	**Male**	**Total**
**Total (N=75342)**	39157 (52.0%)	36185 (48.0%)	75342 (100.0 %)
**Patient Characteristics**			
**Ethnicity**			
White	30274	26580	56854 (75.5 %)
Mixed	263	261	524 (0.7 %)
Asian or Asian British	942	836	1778 (2.4 %)
Black or Black British	388	363	751 (1.0 %)
Other	361	341	702 (0.9 %)
Not known or stated	6929	7804	14733 (19.6 %)
**Age**			
<20	1082	918	2000 (2.7 %)
20-29	4137	3456	7593 (10.1 %)
30-39	4401	3391	7792 (10.3 %)
40-49	4995	4131	9126 (12.1 %)
50-59	5706	5676	11382 (15.1 %)
60-69	5815	6538	12353 (16.4 %)
70-79	6081	6674	12755 (16.9 %)
80-89	5084	4412	9496 (12.6 %)
>89	1856	989	2845 (3.8 %)

For peer review only

**Median Age (IQR)**	58 (40-75)	60 (43 – 74)	59 (41 - 74)
**Admission Characteristics**			
**Main Specialty**			
Medical	17023	13027	30050 (39.9 %)
Surgical	21202	22014	43216 (57.4 %)
Other	932	1144	2076 (2.8 %)
**Admission Method**			
Emergency	21542	19586	41383 (54.9 %)
Elective	17323	16596	33919 (45.0 %)
Other	37	3	40 (0.1 %)
**Hypertension Code**			
Yes	9622	10047	19669 (26.1 %)
No	29535	26138	55673 (73.9 %)

Figure 2b shows that median male DBP peaked at age 50 (76 (CI: 75.5-75.9) mmHg) with lower median DBP at age 20 (66 (CI: 65.0 – 66.0) mmHg) and age 90 (68 (CI: 67.9-68.4) mmHg). In the female cohort, the median DBP was 65 (CI: 64.6-65.0) mmHg, 69 (CI: 69.0-69.4) mmHg, and 68 (CI: 67.6-68.2) mmHg at ages 20, 50 and 90 respectively.

For males, there was a modest reduction in median PP between the ages of 20 and 40 from 55 mmHg (CI: 54.6-55.9) to 50 mmHg (CI: 49.2-50.0), whereas for females PP remained constant at 47mmHg (Figure 2c). Median PP increases for both sexes between the ages of 40 and 90, from 50mmHg (CI: 49.2-50.0) to 63mmHg for males, and 48mmHg (CI: 47.6-48.0)

For peer review only - http://bmjopen.bmj.com/site/about/guidelines.xhtml

to 66mmHg (CI: 65.8.6-67.2) for females. Bootstrapped confidence intervals for SBP, DBP and PP are tabulated in supplementary material (Appendix A).

Figure 3 shows the differences in medians for SBP, DBP and PP between the ages of 20 and 90 for the whole study population in comparison to the subset without an ICD-10 diagnostic code for hypertension.

Figure 4 shows SBP centiles for the Emergency vs Elective sub-populations. DBP and PP centiles are included in Appendix B. In the 24 hours prior to discharge the 95th centile for systolic blood pressure for emergency male admissions at 50 years was 163 mmHg versus 155 mmHg for elective male admissions. Similarly the 95th centile for systolic blood pressure for emergency female admissions at 50 years was 160 mmHg versus 152 mmHg for elective female admissions.

Proportion of patients with blood pressure below published alert thresholds

Table 2 shows the cumulative percentages of male and female patients who had SBP less than 90, 100, and 110 mmHg. These values correspond to the NEWS2 thresholds for hypotension.[7] 100mmHg is also a threshold used to assist in identifying sepsis.[8] For the 100mmHg threshold, 2.3% of younger (20-30yrs) men and 3.5% of older men (81-90yrs) fell below the threshold using their final reading in the 24 hours prior to discharge. In comparison, the criterion was met by 9.7% of younger women and 2.6% of older women.

For peer review only - http://bmjopen.bmj.com/site/about/guidelines.xhtml

Table 2 – percentages of male (N = 36185) and female (N=39157) patients with low systolic blood pressure within each decade

SBP	Gender (N,%)	Age (decade)								
		18-20	21-30	31-40	41-50	51-60	61-70	71-80	81-90	>90
<90	Male (120, 0.3%)	0.2	0.3	0.1	0.2	0.2	0.3	0.5	0.5	0.8
	Female (218, 0.6%)	0.8	1.0	0.9	0.9	0.4	0.4	0.3	0.2	0.2
<100	Male (1063, 2.9%)	2.9	2.3	2.4	2.2	2.6	3.2	3.5	3.5	4.6
	Female (2060, 5.3%)	11.1	9.7	9.4	6.5	4.4	3.1	2.6	2.6	2.0
<110	Male (4817, 13.3%)	16.2	13.2	13.6	12.7	12.9	13.1	12.7	14.5	15.7
	Female (8081, 20.6%)	37.7	35.7	34.7	25.8	18.7	13.2	11.1	10.4	10.8

DISCUSSION

We have generated blood pressure centiles for age and sex from a large multi-hospital patient database.

Discharge blood pressures (SBP, DBP, PP) showed clinically significant differences across age ranges and by sex. SBP progressively increased with age for both sexes, but progression was greater in females. DBP increased and then decreased across the life course for both sexes. The fluctuation in DBP was greater for males than for females. These overall trends were visible in both the whole population, and for the cohort that did not have a diagnostic code for hypertension.

In populations outside hospital these patterns are known to exist.[18-20] The Framingham studies showed that, for a healthy adult population, the mean arterial blood pressure

For peer review only - http://bmjopen.bmj.com/site/about/guidelines.xhtml

increases throughout adulthood and that DBP decreases over the age of 50 as SBP continues to rise.[21] Similar trends in both SBP and DBP have been shown in large cross-sectional cohorts from multiple countries.[22-24]

While the overall patterns for blood pressure are known to exist for the general population outside hospital, we believe that this is the first time that centiles have been derived from an in-hospital setting.

Limitations

Vital signs were recorded as part of standard clinical practice, so the conditions for data recording were not controlled. This may have directly impacted the measured values. For instance, the state of wakefulness of the patient, which is known to be associated with blood pressure and pulse rate, was unknown.[25] However, it seems likley that such effects will be averaged out in a data set of this size.

We used the last recorded blood pressure in the 24 hours prior to discharge. Whilst this lessens many biases in comparison to other methods, and may represent the blood pressure recording when the patient is most stable, there may be other patterns at different points during a hospital admission.

Finally, this study uses a cross-sectional cohort so the derived centiles may be affected by survival bias and birth cohort effects.[26-27] While the influence of these effects cannot be determined, we note that the overall trends follow those previously seen for longitudinal data in healthy populations.[28]

For peer review only - http://bmjopen.bmj.com/site/about/guidelines.xhtml

For peer review only

Interpretation

The differences in normal vital sign ranges due to age and sex could have substantial implications for hospital practice. For example, Table 2 showed that current Systolic Blood Pressure criteria for identifying sepsis (SBP<100 mmHg) would be met by women much more frequently than by men up to age 50. Despite this, current evidence shows that males are more prone to develop sepsis than women.[29] A more accurate identification of patients at risk of sepsis may be possible through sex and age-stratified criteria.

Another important application for age and sex stratification is Early Warning Scores (EWS). In these systems, integer scores are assigned to each vital sign according to deviation from normality. The aggregate score is then used to guide appropriate clinical care. One such EWS, the National Early Warning Score (NEWS), has been validated in a large in-hospital population and is widely used in the United Kingdom and the Republic of Ireland.[30]

Based on the results presented, an age-stratified score could strongly affect the quality of care a patient receives. For instance, from Table 2, we see that 34.7% of women aged 31-40 years have a NEWS score of 1 or greater due to low SBP (SBP ≤ 110). In comparison, only 11.1% of women aged 71-80 years would meet the same blood pressure criterion. In contrast, 13.6% of men aged 31-40 and 12.7% of men aged 71-80 would have a NEWS score of 1 or more. These difference suggest it may be possible to improve discrimination between stability and deterioration by taking account of age and sex.

Until now, age and sex have not been included within any adult EWS, despite evidence indicating its limitations in predicting deterioration of elderly patients.[6-7] An update to the NEWS score to include these additional variables may be difficult to achieve in practice. In

For peer review only - http://bmjopen.bmj.com/site/about/guidelines.xhtml

particular, the NEWS score was validated using in-hospital mortality. Adequate validation of the stratified score would require reasonable numbers of in-hospital mortality for each combination of sex and age-range, where death is relatively rare in younger cohorts. One alternative approach may be to derive a model that directly uses the representative centiles for each vital sign to provide EWS scores.[31]

Generalisability

The data set was collected from all four hospitals, but we note that there are high proportions of white Caucasian patients. Previous studies have shown correlation between ethnicity and differences in blood pressure trajectory.[32] Whether inclusion of ethnicity could also improve discrimiation requires further research.

Our work shows for the first time that meeting current criteria for sepsis or early warning system alerts is substantially more likely in younger women than in all other groups in the 24 hours prior to discharge. Exploration of this finding is needed to determine whether adjustment for age and sex can improve discrimination and prevent overdiagnosis.

CONCLUSION

Substantial variations in the final blood pressure recorded in the 24 hours prior to hospital discharge occur with age and sex. These result in large differences in the proportions of patients meeting blood pressure criterion for sepsis and early warning score alerts. These factors should be examined to understand whether these factors could be used to improve discrimination between stability and deterioration.

For peer review only - http://bmjopen.bmj.com/site/about/guidelines.xhtml

For peer review only

COMPETING INTERESTS

DW and PW co-developed the SEND e-Obs system (for which Sensyne Health has purchased sole license) from which the study database was collected. and. The company has a research agreement with the University of Oxford and royalty agreements with Oxford University Hospitals NHS Trust and the University of Oxford. DAC is Research Director of Sensyne Health. PW is employed part-time and holds shares in Sensyne Health. DW undertakes consultancy for Sensyne Health.

FUNDING

This publication presents independent research funded by the Health Innovation Challenge Fund (HICF-R9-524; WT-103703/Z/14/Z), a parallel funding partnership between the Department of Health and Wellcome Trust. The views expressed in this publication are those of the author(s) and not necessarily those of the Department of Health or Wellcome Trust. PW and DAC are funded by the Health Innovation Challenge Fund and Wellcome Trust (HICF-R9-524 and WT-103703/Z/14/Z). PW is supported by the NIHR Biomedical Research Centre, Oxford. SG is funded via an NIHR Doctoral Research Fellowship. FS is funded by the Rhodes Trust.

For peer review only

AUTHOR CONTRIBUTIONS

DW, DAC, PW conceived and designed the study; MP, DW, SG acquired the data; SG, DW, FS analysed the data. DW, SG, DAC, MP, FS, PW were in involved in drafting and revising the manuscript and have approved the final submitted version.

DATA SHARING STATEMENT

The raw data for this research are not openly available. Any requests regarding data access should be made to ccrg@ndcn.ox.ac.uk

REFERENCES

1. Cuthbertson BH, Boroujerdi M, Mckie L, et al. Can physiological variables and early warning scoring systems allow early recognition of the deteriorating surgical patient? *Crit Care Med* 2007;35:402-9.

2. Duncan H, Hutchison J, Parshuram CS. The Pediatric Early Warning System score: a severity of illness score to predict urgent medical need in hospitalized children. *J Crit Care* 2006;21(3):271-8.

3. Fleming S, Thompson M, Stevens R, et al. Normal ranges of heart rate and respiratory rate in children from birth to 18 years of age: a systematic review of observational studies. *Lancet* 2011;377:1011-8.

4. Smith GB, Prytherch DR, Meredith P, et al. The ability of the National Early Warning Score (NEWS) to discriminate patients at risk of early cardiac arrest, unanticipated intensive care unit admission, and death. *Resuscitation* 2013;84:465-70.

5. Smith GB, Prytherch DR, Schmidt PE, et al. Should age be included as a component of track and trigger systems used to identify sick adult patients? *Resuscitation* 2008;78(2):109-15.

6. Churpek MM, Yuen TC, Winslow C, et al. Differences in vital signs between elderly and non-elderly patients prior to ward cardiac arrest. *Crit Care Med* 2015;43(4):816.

7. Royal College of Physicians (London). National Early Warning Score (NEWS) 2 standardising the assessment of acute-illness severity in the NHS. 2017

For peer review only - http://bmjopen.bmj.com/site/about/guidelines.xhtml

8. Singer M, Deutschman CS, Seymour CW, et al. The third international consensus definitions for sepsis and septic shock (sepsis-3). *Jama* 2016;315(8):801-10.

9. Franklin SS, Gustin W, Wong ND, et al. Hemodynamic patterns of age-related changes in blood pressure - The framingham heart study. *Circulation* 1997;96:308-15.

10. Wong D, Bonnici T, Knight J, et al. SEND: a system for electronic notification and documentation of vital sign observations. *BMC Med Inform Decis Mak* 2015;15(1):1.

11. NHS Data Model and Dictionary. NHS Data Model and Dictionary Version 3 http://www.datadictionary.nhs.uk/ (date accessed: 13 November 2018)

12. Beckman KD. How to document and code for hypertensive diseases in ICD-10. *Family practice management* 2014 Apr;21(2):5-9.

13. Rigby, RA, Stasinopoulos, DM. Generalized additive models for Location, Scale and Shape (GAMLSS) in R. *J Stat Soft* 2007; 23: 1–46

14. Akaike, H. A new look at the statistical model identification. *IEEE Trans Automat Contr* 1974;19:716–723

15. Schwarz, G. Estimating the dimension of a model. *Ann Stats* 1978;6:461–464.

16. R Development Core Team (2008). R: A language and environment for statistical computing. R Foundation for Statistical Computing, Vienna, Austria. ISBN 3-900051-07-0, URL http://www.R-project.org.

17. Bland JM, Altman DG. Statistics notes: bootstrap resampling methods. *BMJ* 2015;350:h2622.

For peer review only - http://bmjopen.bmj.com/site/about/guidelines.xhtml

18. Pinto E. Blood pressure and ageing. *Postgrad Med J* 2007;83(976):109-14.

19. Baker SE, Limberg JK, Ranadive SM, et al. Neurovascular control of blood pressure is influenced by aging, sex, and sex hormones. *Am J Physiol* 2016;311(6):R1271-5.

20. Joyner MJ, Wallin BG, Charkoudian N. Sex differences and blood pressure regulation in humans. *Exp Physiol* 2016;101(3):349-55.

21. Franklin SS, Khan SA, Wong ND, et al. Is pulse pressure useful in predicting risk for coronary heart disease? *Circulation* 1999;100(4):354-60.

22. Hosseini M, Baikpour M, Yousefifard M, et al. Blood pressure percentiles by age and body mass index for adults. *EXCLI J*. 2015;14:465-77

23. Balijepalli C, Lösch C, Bramlage P, et al. Percentile distribution of blood pressure readings in 35683 men and women aged 18 to 99 years. *J Hum Hypertens* 2014;28(3):193-200

24. Wright JD, Hughes JP, Ostchega Y,et al. Mean systolic and diastolic blood pressure in adults aged 18 and over in the United States, 2001–2008. *Natl Health Stat Report* 2011;35(1-22):24.

25. D. Mancia G, Ferrari A, Gregorini L, et al. Blood pressure and heart rate variabilities in normotensive and hypertensive human beings. *Circ Res* 1983;53(1):96-104.

26. Delgado-Rodriguez M, Llorca J. Bias. *J Epidemiol Community Health* 2004;58(8):635-41.

27. Keyes KM, Utz RL, Robinson W, Li G. What is a cohort effect? Comparison of three statistical methods for modeling cohort effects in obesity prevalence in the United States, 1971–2006. *Soc Sci Med* 2010;70(7):1100-8.

For peer review only - http://bmjopen.bmj.com/site/about/guidelines.xhtml

28. Wills AK, Lawlor DA, Matthews FE, et al. Life course trajectories of systolic blood pressure using longitudinal data from eight UK cohorts. PLoS Med 2011;8(6):e1000440.

29. Sakr Y, Elia C, Mascia L, et al. The influence of gender on the epidemiology of and outcome from severe sepsis. *Critical Care* 2013;17(2):R50.

30. Prytherch DR, Smith GB, Schmidt PE, et al. ViEWS—towards a national early warning score for detecting adult inpatient deterioration. *Resuscitation* 2010;81(8):932-7.

31. Tarassenko L, Clifton DA, Pinsky MR, et al. Centile-based early warning scores derived from statistical distributions of vital signs. *Resuscitation* 2011;82(8):1013-8.

32. Agyemang C, Humphry RW, Bhopal R. Divergence with age in blood pressure in African-Caribbean and white populations in England: implications for screening for hypertension. *Am J Hypertens*. 2012; 25(1):89-96

For peer review only

For peer review only - http://bmjopen.bmj.com/site/about/guidelines.xhtml

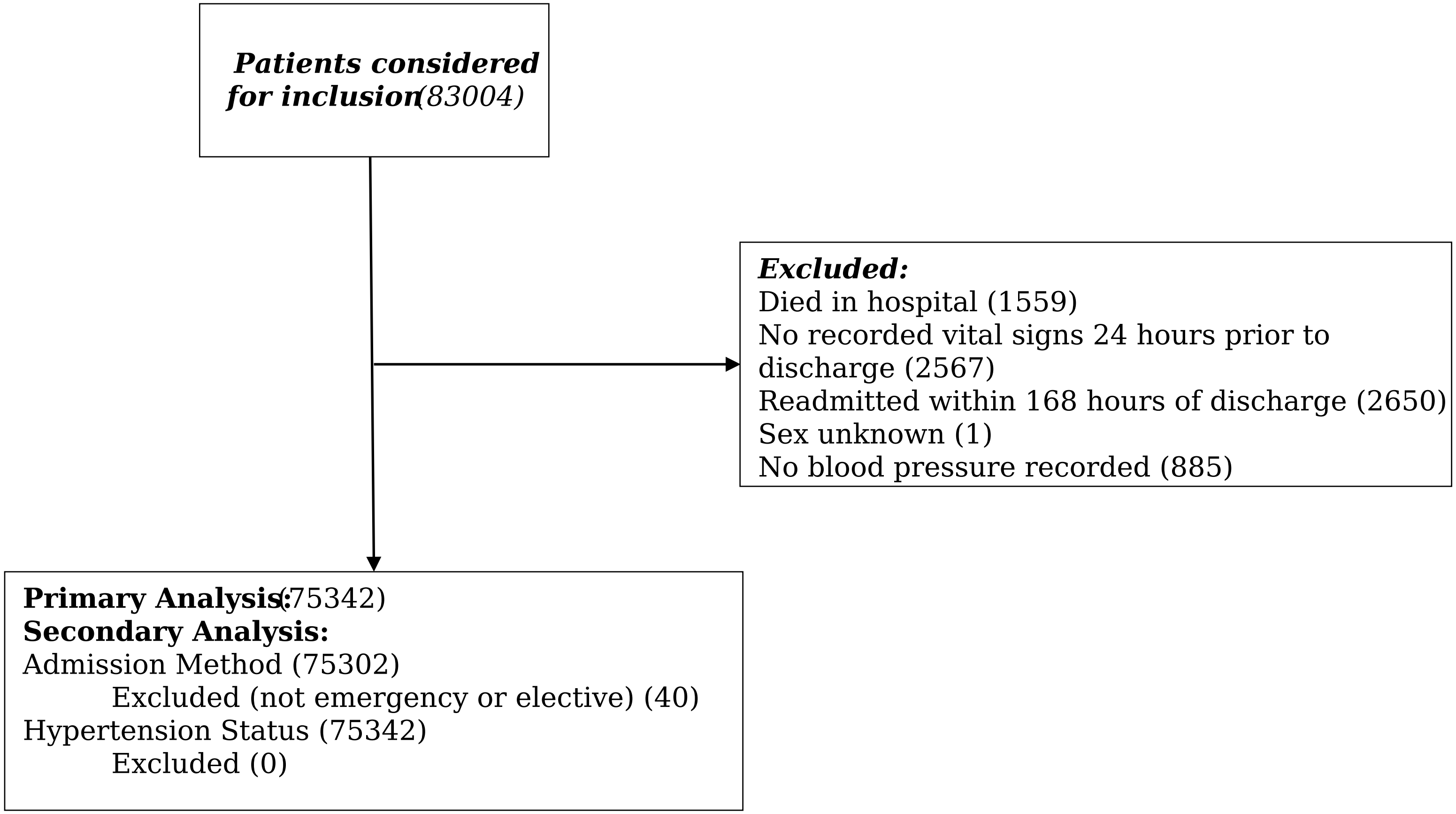

For peer review only - http://bmjopen.bmj.com/site/about/guidelines.xhtml

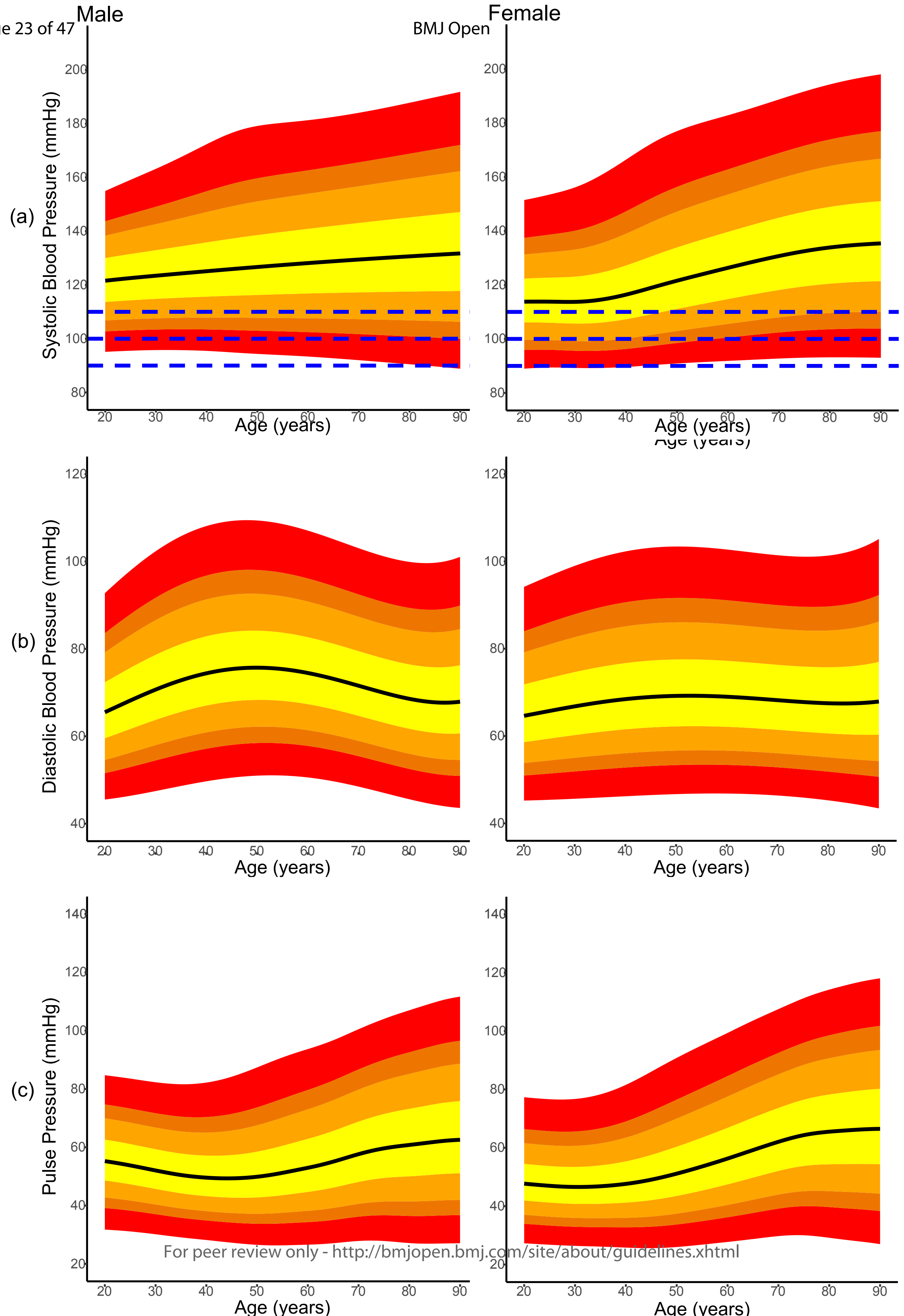

Male
Female
(a)
Systolic Blood Pressure (mmHg)
200
180
160
140
120
100
80
20
30
40
50
60
70
80
90
Age (years)
(b)
Diastolic Blood Pressure (mmHg)
120
100
80
60
40
Age (years)
(c)
Pulse Pressure (mmHg)
140
120
100
80
60
40
20
Age (years)

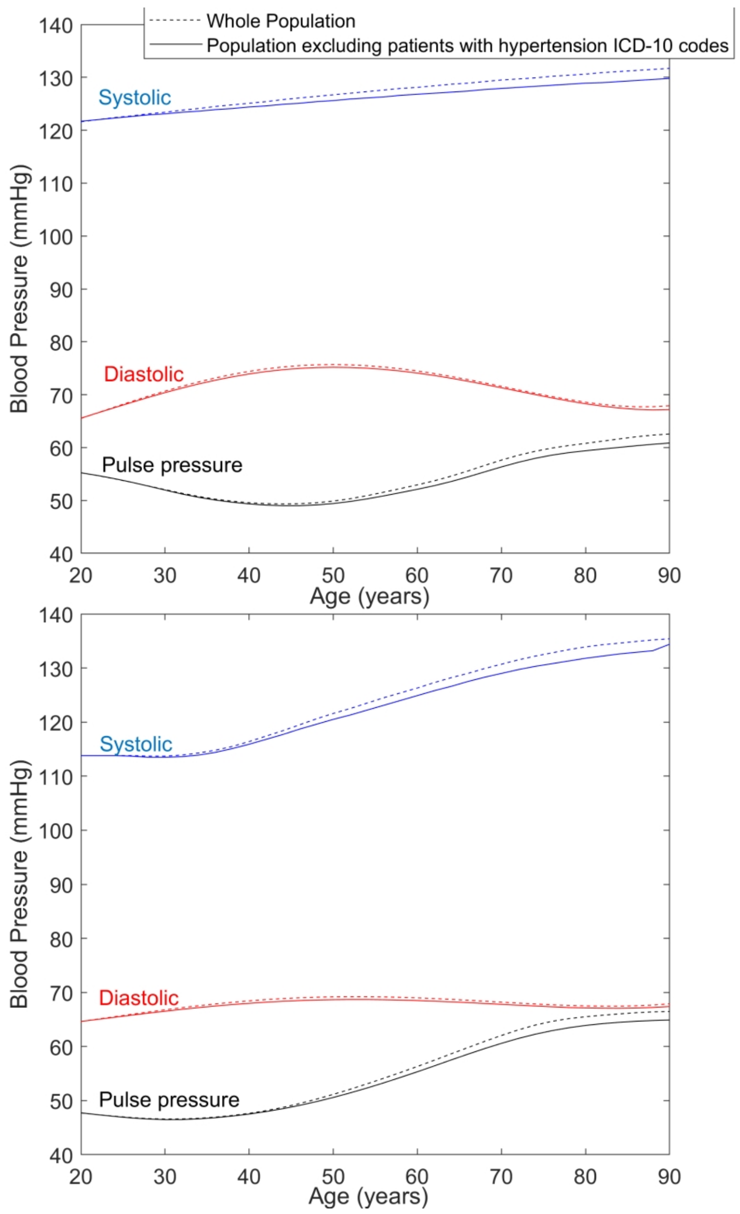

Figure 3. Medians of systolic, diastolic and pulse blood pressure for all males and females between the ages of 20 and 90 (dashed lines) and the sub-group excluding patients with ICD codes for hypertension (solid lines).

152x239mm (300 x 300 DPI)

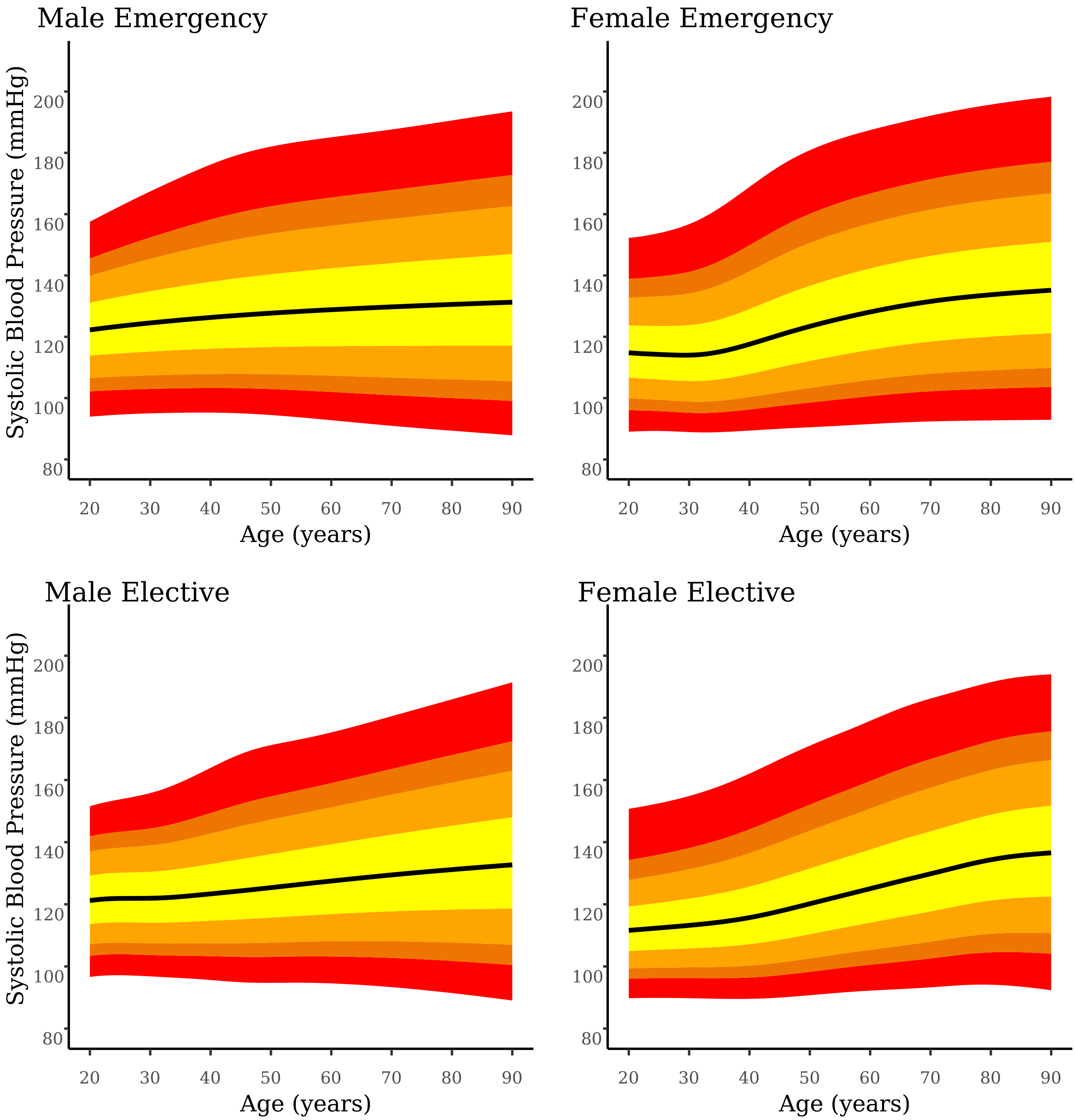
Male Emergency
Female Emergency
Male Elective
Female Elective
Systolic Blood Pressure (mmHg)
80
100
120
140
160
180
200
20
30
40
50
60
70
80
90
Age (years)

Supplementary Material

Appendix A – centiles and confidence intervals

Male systolic blood pressure centiles with 95% CI

	Age	_1st	_5th	_10th	_25th	_50th	_75th	_90th	_95th	_99th
1	20	95.12 (93.60, 96.65)	102.73 (101.90, 103.6)	106.8 (106.1, 107.5)	113.7 (113.1, 114.2)	121.6 (121.1, 122.1)	130.0 (129.4, 130.6)	138.3 (137.5, 139.1)	143.7 (142.7, 144.7)	154.9 (152.9, 157.0)
2	22	95.30 (94.02, 96.57)	102.91 (102.22, 103.6)	107.0 (106.4, 107.6)	113.9 (113.5, 114.4)	122.0 (121.6, 122.4)	130.6 (130.2, 131.1)	139.2 (138.6, 139.8)	144.8 (144.1, 145.5)	156.6 (154.9, 158.3)
3	24	95.46 (94.34, 96.58)	103.08 (102.48, 103.7)	107.2 (106.7, 107.7)	114.2 (113.8, 114.6)	122.4 (122.0, 122.7)	131.2 (130.9, 131.6)	140.1 (139.6, 140.6)	145.9 (145.2, 146.6)	158.3 (156.6, 160.0)
4	26	95.60 (94.56, 96.65)	103.22 (102.67, 103.8)	107.3 (106.9, 107.8)	114.4 (114.1, 114.8)	122.7 (122.5, 123.0)	131.8 (131.5, 132.2)	140.9 (140.4, 141.5)	147.0 (146.2, 147.8)	159.9 (158.0, 161.9)
5	28	95.71 (94.73, 96.69)	103.33 (102.78, 103.9)	107.5 (107.0, 107.9)	114.6 (114.2, 115.0)	123.1 (122.7, 123.5)	132.4 (131.9, 132.9)	141.8 (141.1, 142.5)	148.0 (147.1, 149.0)	161.6 (159.5, 163.6)
6	30	95.78 (94.85, 96.71)	103.42 (102.83, 104.0)	107.6 (107.1, 108.1)	114.8 (114.3, 115.3)	123.4 (122.9, 123.9)	132.9 (132.3, 133.6)	142.6 (141.8, 143.5)	149.1 (148.0, 150.2)	163.2 (161.1, 165.3)
7	32	95.80 (94.90, 96.69)	103.48 (102.88, 104.1)	107.7 (107.1, 108.2)	115.0 (114.5, 115.5)	123.8 (123.2, 124.3)	133.5 (132.8, 134.2)	143.5 (142.6, 144.4)	150.2 (149.0, 151.4)	164.9 (162.8, 167.1)
8	34	95.78 (94.91, 96.65)	103.51 (102.94, 104.1)	107.8 (107.2, 108.3)	115.2 (114.7, 115.7)	124.1 (123.6, 124.6)	134.1 (133.4, 134.8)	144.4 (143.5, 145.3)	151.4 (150.2, 152.5)	166.7 (164.5, 168.9)
9	36	95.72 (94.85, 96.58)	103.52 (102.98, 104.1)	107.8 (107.4, 108.3)	115.4 (114.9, 115.8)	124.5 (124.0, 124.9)	134.7 (134.1, 135.3)	145.3 (144.4, 146.2)	152.5 (151.3, 153.7)	168.6 (166.4, 170.7)
10	38	95.62 (94.74, 96.49)	103.50 (102.97, 104.0)	107.9 (107.4, 108.3)	115.5 (115.1, 115.9)	124.8 (124.4, 125.2)	135.3 (134.7, 135.8)	146.2 (145.3, 147.1)	153.7 (152.5, 154.9)	170.5 (168.2, 172.7)
11	40	95.47 (94.57, 96.36)	103.46 (102.92, 104.0)	107.9 (107.4, 108.3)	115.7 (115.3, 116.0)	125.1 (124.7, 125.5)	135.8 (135.3, 136.4)	147.1 (146.2, 148.0)	154.9 (153.7, 156.0)	172.4 (170.1, 174.6)
12	42	95.27 (94.33, 96.20)	103.38 (102.84, 103.9)	107.9 (107.4, 108.3)	115.8 (115.4, 116.2)	125.4 (125.1, 125.8)	136.4 (135.9, 137.0)	148.0 (147.2, 148.8)	156.0 (154.9, 157.1)	174.2 (172.0, 176.4)

For peer review only - http://bmjopen.bmj.com/site/about/guidelines.xhtml

	Age	_1st	_5th	_10th	_25th	_50th	_75th	_90th	_95th	_99th
13	44	95.03 (93.99, 96.07)	103.28 (102.72, 103.8)	107.9 (107.4, 108.3)	115.9 (115.6, 116.3)	125.8 (125.4, 126.1)	137.0 (136.5, 137.5)	148.9 (148.1, 149.6)	157.1 (156.0, 158.2)	175.9 (173.5, 178.2)
14	46	94.79 (93.62, 95.96)	103.18 (102.59, 103.8)	107.8 (107.4, 108.3)	116.0 (115.7, 116.4)	126.1 (125.7, 126.4)	137.5 (137.0, 138.0)	149.7 (148.9, 150.4)	158.1 (157.0, 159.1)	177.3 (174.7, 179.8)
15	48	94.56 (93.36, 95.76)	103.07 (102.48, 103.7)	107.8 (107.4, 108.2)	116.2 (115.8, 116.5)	126.4 (126.1, 126.7)	138.1 (137.6, 138.5)	150.4 (149.7, 151.1)	158.9 (157.9, 160.0)	178.4 (175.7, 181.0)
16	50	94.35 (93.26, 95.44)	102.97 (102.42, 103.5)	107.8 (107.4, 108.2)	116.3 (116.0, 116.6)	126.7 (126.4, 127.0)	138.6 (138.1, 139.0)	151.1 (150.4, 151.8)	159.7 (158.7, 160.7)	179.1 (176.7, 181.6)
17	52	94.16 (93.22, 95.10)	102.87 (102.37, 103.4)	107.8 (107.4, 108.1)	116.4 (116.1, 116.7)	127.0 (126.7, 127.3)	139.0 (138.6, 139.5)	151.7 (151.0, 152.4)	160.4 (159.4, 161.3)	179.7 (177.6, 181.9)
18	54	93.98 (93.14, 94.82)	102.78 (102.29, 103.3)	107.7 (107.3, 108.1)	116.5 (116.2, 116.8)	127.3 (127.0, 127.6)	139.5 (139.1, 139.9)	152.3 (151.6, 152.9)	161.0 (160.1, 161.9)	180.1 (178.2, 182.1)
19	56	93.79 (92.98, 94.60)	102.68 (102.19, 103.2)	107.7 (107.3, 108.1)	116.6 (116.3, 116.9)	127.6 (127.3, 127.8)	140.0 (139.6, 140.4)	152.8 (152.2, 153.5)	161.5 (160.6, 162.4)	180.5 (178.6, 182.4)
20	58	93.58 (92.79, 94.38)	102.57 (102.09, 103.0)	107.7 (107.3, 108.0)	116.7 (116.4, 117.0)	127.9 (127.6, 128.1)	140.4 (140.1, 140.8)	153.4 (152.8, 154.0)	162.1 (161.2, 163.0)	180.9 (179.0, 182.7)
21	60	93.36 (92.60, 94.13)	102.45 (101.98, 102.9)	107.6 (107.2, 108.0)	116.8 (116.5, 117.1)	128.1 (127.8, 128.4)	140.9 (140.5, 141.3)	153.9 (153.3, 154.5)	162.7 (161.8, 163.5)	181.3 (179.5, 183.1)
22	62	93.12 (92.40, 93.85)	102.31 (101.88, 102.7)	107.6 (107.2, 107.9)	116.9 (116.6, 117.2)	128.4 (128.1, 128.7)	141.3 (141.0, 141.7)	154.5 (153.9, 155.1)	163.2 (162.4, 164.1)	181.7 (180.0, 183.5)
23	64	92.86 (92.16, 93.56)	102.17 (101.76, 102.6)	107.5 (107.1, 107.8)	117.0 (116.7, 117.3)	128.7 (128.4, 129.0)	141.8 (141.4, 142.1)	155.0 (154.5, 155.6)	163.8 (163.0, 164.6)	182.2 (180.6, 183.9)
24	66	92.59 (91.89, 93.30)	102.03 (101.60, 102.4)	107.4 (107.1, 107.8)	117.1 (116.8, 117.4)	128.9 (128.6, 129.3)	142.2 (141.8, 142.6)	155.6 (155.0, 156.2)	164.4 (163.6, 165.2)	182.8 (181.2, 184.4)
25	68	92.31 (91.58, 93.04)	101.87 (101.43, 102.3)	107.3 (107.0, 107.7)	117.2 (116.8, 117.5)	129.2 (128.9, 129.5)	142.6 (142.3, 143.0)	156.2 (155.6, 156.7)	165.0 (164.2, 165.8)	183.4 (181.8, 185.0)
26	70	92.02 (91.25, 92.79)	101.71 (101.24, 102.2)	107.3 (106.9, 107.7)	117.2 (116.9, 117.6)	129.5 (129.1, 129.8)	143.1 (142.7, 143.5)	156.7 (156.1, 157.3)	165.6 (164.8, 166.4)	184.0 (182.5, 185.6)
27	72	91.72 (90.91, 92.54)	101.55 (101.04, 102.1)	107.2 (106.8, 107.6)	117.3 (116.9, 117.7)	129.7 (129.3, 130.1)	143.5 (143.1, 143.9)	157.3 (156.7, 157.9)	166.2 (165.4, 167.1)	184.7 (183.2, 186.3)

	Age	_1st	_5th	_10th	_25th	_50th	_75th	_90th	_95th	_99th
28	74	91.42 (90.58, 92.27)	101.39 (100.85, 101.9)	107.1 (106.7, 107.5)	117.4 (117.0, 117.8)	129.9 (129.6, 130.3)	143.9 (143.5, 144.3)	157.8 (157.2, 158.4)	166.9 (166.1, 167.7)	185.4 (183.9, 187.0)
29	76	91.12 (90.27, 91.97)	101.22 (100.68, 101.8)	107.0 (106.6, 107.5)	117.4 (117.1, 117.8)	130.2 (129.8, 130.6)	144.3 (143.9, 144.8)	158.4 (157.8, 159.0)	167.5 (166.7, 168.4)	186.2 (184.7, 187.7)
30	78	90.82 (89.99, 91.64)	101.05 (100.51, 101.6)	106.9 (106.5, 107.4)	117.5 (117.1, 117.9)	130.4 (130.0, 130.8)	144.7 (144.2, 145.2)	159.0 (158.3, 159.7)	168.2 (167.3, 169.1)	187.0 (185.4, 188.5)
31	80	90.51 (89.70, 91.31)	100.87 (100.33, 101.4)	106.8 (106.4, 107.3)	117.5 (117.2, 117.9)	130.6 (130.2, 131.1)	145.1 (144.6, 145.7)	159.5 (158.8, 160.3)	168.8 (167.8, 169.8)	187.8 (186.1, 189.5)
32	82	90.19 (89.39, 91.00)	100.70 (100.14, 101.3)	106.7 (106.3, 107.2)	117.6 (117.2, 118.0)	130.9 (130.4, 131.3)	145.5 (145.0, 146.1)	160.1 (159.3, 161.0)	169.5 (168.4, 170.6)	188.6 (186.7, 190.5)
33	84	89.87 (89.05, 90.68)	100.51 (99.94, 101.1)	106.6 (106.1, 107.1)	117.6 (117.2, 118.1)	131.1 (130.6, 131.6)	145.9 (145.3, 146.6)	160.7 (159.7, 161.6)	170.1 (168.9, 171.4)	189.4 (187.3, 191.5)
34	86	89.54 (88.70, 90.37)	100.32 (99.71, 100.9)	106.5 (106.0, 107.1)	117.7 (117.1, 118.2)	131.3 (130.7, 131.9)	146.3 (145.6, 147.0)	161.2 (160.2, 162.2)	170.8 (169.5, 172.1)	190.2 (187.9, 192.5)
35	88	89.20 (88.31, 90.08)	100.13 (99.44, 100.8)	106.4 (105.8, 107.1)	117.7 (117.1, 118.4)	131.5 (130.8, 132.2)	146.7 (146.0, 147.5)	161.8 (160.7, 162.9)	171.5 (170.0, 172.9)	191.0 (188.5, 193.6)
36	90	88.85 (87.84, 89.86)	99.93 (99.11, 100.7)	106.3 (105.5, 107.1)	117.8 (117.0, 118.5)	131.7 (130.9, 132.5)	147.1 (146.2, 148.0)	162.3 (161.1, 163.6)	172.1 (170.5, 173.7)	191.8 (189.0, 194.6)

Female systolic blood pressure centiles with 95% CI

	Age	_1st	_5th	_10th	_25th	_50th	_75th	_90th	_95th	_99th
1	20	88.97 (87.67, 90.27)	95.95 (95.19, 96.70)	99.69 (99.04, 100.34)	106.1 (105.5, 106.7)	113.8 (113.1, 114.4)	122.3 (121.6, 123.1)	131.3 (130.4, 132.1)	137.4 (136.5, 138.4)	151.4 (149.5, 153.4)
2	22	89.22 (88.16, 90.28)	95.99 (95.37, 96.62)	99.69 (99.17, 100.21)	106.1 (105.7, 106.6)	113.8 (113.3, 114.3)	122.5 (121.9, 123.1)	131.6 (130.9, 132.4)	137.9 (137.0, 138.8)	152.2 (150.6, 153.8)
3	24	89.37 (88.44, 90.30)	95.99 (95.40, 96.58)	99.64 (99.13, 100.15)	106.0 (105.6, 106.5)	113.8 (113.3, 114.3)	122.6 (122.0, 123.3)	132.0 (131.1, 132.8)	138.4 (137.4, 139.4)	153.0 (151.2, 154.9)
4	26	89.41 (88.60, 90.22)	95.91 (95.37, 96.45)	99.53 (99.03, 100.02)	105.9 (105.5, 106.4)	113.8 (113.3, 114.3)	122.8 (122.1, 123.4)	132.3 (131.4, 133.1)	138.9 (137.9, 139.9)	153.9 (152.0, 155.9)

For peer review only - http://bmjopen.bmj.com/site/about/guidelines.xhtml

	Age	_1st	_5th	_10th	_25th	_50th	_75th	_90th	_95th	_99th
5	28	89.35 (88.64, 90.06)	95.76 (95.31, 96.20)	99.36 (98.95, 99.76)	105.8 (105.4, 106.2)	113.7 (113.3, 114.2)	122.9 (122.3, 123.5)	132.6 (131.8, 133.4)	139.4 (138.4, 140.4)	154.9 (153.0, 156.9)
6	30	89.26 (88.58, 89.93)	95.61 (95.22, 96.01)	99.22 (98.84, 99.59)	105.7 (105.3, 106.1)	113.7 (113.3, 114.2)	123.1 (122.5, 123.7)	133.1 (132.3, 133.9)	140.1 (139.1, 141.1)	156.2 (154.1, 158.2)
7	32	89.20 (88.51, 89.88)	95.55 (95.13, 95.96)	99.17 (98.78, 99.55)	105.7 (105.3, 106.1)	113.9 (113.4, 114.4)	123.5 (122.9, 124.1)	133.8 (133.0, 134.6)	141.0 (140.0, 142.1)	157.7 (155.5, 159.8)
8	34	89.21 (88.52, 89.89)	95.59 (95.17, 96.01)	99.25 (98.87, 99.63)	105.9 (105.5, 106.3)	114.3 (113.8, 114.7)	124.1 (123.5, 124.7)	134.8 (134.0, 135.6)	142.3 (141.2, 143.3)	159.5 (157.3, 161.7)
9	36	89.28 (88.61, 89.95)	95.73 (95.33, 96.14)	99.45 (99.09, 99.81)	106.2 (105.9, 106.6)	114.8 (114.4, 115.2)	125.0 (124.5, 125.5)	136.0 (135.2, 136.7)	143.7 (142.7, 144.8)	161.7 (159.4, 163.9)
10	38	89.42 (88.77, 90.07)	95.97 (95.58, 96.36)	99.77 (99.42, 100.11)	106.7 (106.3, 107.1)	115.5 (115.1, 116.0)	126.0 (125.5, 126.5)	137.4 (136.7, 138.1)	145.4 (144.4, 146.5)	164.0 (161.7, 166.3)
11	40	89.61 (88.97, 90.25)	96.29 (95.91, 96.68)	100.18 (99.84, 100.53)	107.3 (106.9, 107.7)	116.4 (116.0, 116.8)	127.2 (126.7, 127.7)	139.0 (138.2, 139.7)	147.3 (146.2, 148.3)	166.4 (164.1, 168.8)
12	42	89.85 (89.20, 90.49)	96.69 (96.29, 97.09)	100.68 (100.32, 101.04)	108.0 (107.6, 108.4)	117.4 (116.9, 117.8)	128.5 (128.0, 129.1)	140.7 (139.9, 141.5)	149.2 (148.1, 150.3)	168.9 (166.6, 171.2)
13	44	90.12 (89.45, 90.79)	97.13 (96.70, 97.56)	101.23 (100.84, 101.62)	108.8 (108.4, 109.2)	118.4 (118.0, 118.9)	129.9 (129.3, 130.5)	142.4 (141.6, 143.2)	151.2 (150.1, 152.2)	171.3 (169.0, 173.5)
14	46	90.40 (89.70, 91.09)	97.60 (97.14, 98.06)	101.81 (101.40, 102.22)	109.6 (109.2, 110.0)	119.5 (119.1, 119.9)	131.3 (130.7, 131.9)	144.1 (143.3, 144.9)	153.0 (152.0, 154.1)	173.4 (171.3, 175.6)
15	48	90.66 (89.97, 91.36)	98.06 (97.57, 98.54)	102.38 (101.95, 102.81)	110.4 (109.9, 110.8)	120.6 (120.1, 121.0)	132.6 (132.1, 133.2)	145.7 (144.9, 146.4)	154.8 (153.8, 155.7)	175.3 (173.3, 177.4)
16	50	90.91 (90.23, 91.58)	98.50 (98.01, 98.98)	102.94 (102.50, 103.38)	111.1 (110.7, 111.5)	121.6 (121.1, 122.0)	133.9 (133.4, 134.4)	147.1 (146.4, 147.9)	156.3 (155.4, 157.3)	177.0 (175.0, 178.9)
17	52	91.13 (90.48, 91.77)	98.92 (98.45, 99.39)	103.48 (103.03, 103.93)	111.9 (111.4, 112.3)	122.5 (122.0, 123.1)	135.1 (134.5, 135.7)	148.5 (147.8, 149.2)	157.8 (156.8, 158.7)	178.4 (176.6, 180.2)
18	54	91.32 (90.69, 91.96)	99.32 (98.86, 99.79)	104.00 (103.55, 104.45)	112.6 (112.1, 113.1)	123.5 (123.0, 124.0)	136.3 (135.7, 136.9)	149.8 (149.1, 150.6)	159.1 (158.2, 160.0)	179.6 (177.8, 181.4)
19	56	91.51 (90.83, 92.19)	99.72 (99.24, 100.21)	104.52 (104.07, 104.98)	113.3 (112.9, 113.8)	124.5 (124.0, 125.0)	137.4 (136.9, 138.0)	151.1 (150.4, 151.8)	160.4 (159.5, 161.3)	180.7 (178.9, 182.5)

For peer review only - http://bmjopen.bmj.com/site/about/guidelines.xhtml

	Age	_1st	_5th	_10th	_25th	_50th	_75th	_90th	_95th	_99th
20	58	91.68 (90.92, 92.45)	100.12 (99.60, 100.64)	105.04 (104.58, 105.50)	114.0 (113.6, 114.5)	125.4 (124.9, 125.9)	138.5 (138.0, 139.0)	152.3 (151.7, 152.9)	161.6 (160.7, 162.5)	181.8 (179.9, 183.7)
21	60	91.86 (91.01, 92.71)	100.52 (99.95, 101.08)	105.56 (105.07, 106.04)	114.8 (114.3, 115.2)	126.3 (125.9, 126.7)	139.7 (139.2, 140.1)	153.5 (152.9, 154.1)	162.8 (162.0, 163.7)	182.8 (180.8, 184.8)
22	62	92.04 (91.15, 92.92)	100.91 (100.32, 101.50)	106.07 (105.56, 106.59)	115.5 (115.0, 115.9)	127.3 (126.8, 127.7)	140.7 (140.4, 141.1)	154.7 (154.1, 155.2)	164.0 (163.2, 164.9)	183.9 (181.9, 186.0)
23	64	92.22 (91.31, 93.12)	101.30 (100.69, 101.91)	106.58 (106.03, 107.12)	116.2 (115.7, 116.7)	128.2 (127.7, 128.6)	141.8 (141.4, 142.3)	155.9 (155.3, 156.5)	165.2 (164.4, 166.1)	185.1 (183.1, 187.1)
24	66	92.39 (91.43, 93.36)	101.68 (101.06, 102.30)	107.07 (106.50, 107.64)	116.8 (116.3, 117.4)	129.0 (128.5, 129.5)	142.9 (142.4, 143.4)	157.1 (156.4, 157.7)	166.4 (165.6, 167.3)	186.3 (184.3, 188.2)
25	68	92.57 (91.56, 93.58)	102.05 (101.41, 102.69)	107.54 (106.95, 108.13)	117.5 (116.9, 118.1)	129.9 (129.3, 130.4)	143.9 (143.3, 144.5)	158.2 (157.5, 158.9)	167.6 (166.7, 168.6)	187.5 (185.5, 189.4)
26	70	92.73 (91.78, 93.68)	102.39 (101.77, 103.02)	107.99 (107.40, 108.58)	118.1 (117.5, 118.7)	130.7 (130.1, 131.3)	144.9 (144.3, 145.5)	159.3 (158.6, 160.1)	168.8 (167.9, 169.7)	188.7 (186.9, 190.5)
27	72	92.88 (92.07, 93.68)	102.71 (102.13, 103.29)	108.40 (107.83, 108.97)	118.7 (118.1, 119.3)	131.5 (130.9, 132.0)	145.8 (145.2, 146.5)	160.4 (159.6, 161.2)	170.0 (169.0, 170.9)	189.9 (188.3, 191.5)
28	74	93.00 (92.30, 93.69)	102.99 (102.44, 103.54)	108.77 (108.23, 109.32)	119.2 (118.7, 119.8)	132.2 (131.6, 132.7)	146.7 (146.1, 147.4)	161.4 (160.6, 162.3)	171.1 (170.0, 172.1)	191.1 (189.4, 192.8)
29	76	93.09 (92.40, 93.77)	103.23 (102.69, 103.77)	109.10 (108.57, 109.63)	119.7 (119.2, 120.2)	132.8 (132.2, 133.4)	147.5 (146.9, 148.2)	162.4 (161.5, 163.2)	172.1 (171.0, 173.2)	192.2 (190.4, 194.1)
30	78	93.15 (92.39, 93.91)	103.43 (102.86, 103.99)	109.37 (108.84, 109.90)	120.1 (119.6, 120.6)	133.4 (132.8, 133.9)	148.3 (147.6, 148.9)	163.2 (162.4, 164.1)	173.0 (171.9, 174.2)	193.3 (191.3, 195.3)
31	80	93.18 (92.34, 94.01)	103.58 (102.98, 104.17)	109.59 (109.06, 110.12)	120.5 (120.0, 120.9)	133.9 (133.4, 134.4)	148.9 (148.3, 149.5)	164.0 (163.2, 164.9)	173.9 (172.7, 175.0)	194.3 (192.2, 196.3)
32	82	93.18 (92.35, 94.02)	103.69 (103.09, 104.28)	109.76 (109.24, 110.28)	120.7 (120.3, 121.2)	134.3 (133.8, 134.8)	149.5 (148.9, 150.0)	164.7 (163.9, 165.5)	174.7 (173.5, 175.8)	195.2 (193.2, 197.2)
33	84	93.17 (92.34, 93.99)	103.76 (103.16, 104.36)	109.89 (109.36, 110.42)	121.0 (120.5, 121.5)	134.6 (134.1, 135.1)	149.9 (149.3, 150.5)	165.3 (164.5, 166.2)	175.3 (174.2, 176.5)	196.0 (194.0, 198.0)
34	86	93.13 (92.30, 93.96)	103.81 (103.18, 104.43)	109.98 (109.42, 110.55)	121.1 (120.6, 121.7)	134.9 (134.3, 135.5)	150.4 (149.6, 151.1)	165.8 (164.9, 166.8)	175.9 (174.7, 177.2)	196.7 (194.7, 198.8)

	Age	_1st	_5th	_10th	_25th	_50th	_75th	_90th	_95th	_99th
35	88	93.08 (92.23, 93.92)	103.82 (103.19, 104.46)	110.05 (109.44, 110.65)	121.3 (120.7, 121.9)	135.2 (134.4, 135.9)	150.7 (149.8, 151.6)	166.3 (165.2, 167.4)	176.5 (175.1, 177.9)	197.4 (195.1, 199.7)
36	90	93.01 (92.14, 93.88)	103.82 (103.16, 104.48)	110.08 (109.44, 110.72)	121.4 (120.7, 122.1)	135.4 (134.5, 136.2)	151.0 (150.0, 152.0)	166.7 (165.4, 168.0)	176.9 (175.3, 178.6)	198.0 (195.3, 200.7)

Male diastolic blood pressure centiles with 95% CI

	Age	_1st	_5th	_10th	_25th	_50th	_75th	_90th	_95th	_99th
1	20	45.53 (44.75, 46.31)	51.56 (50.97, 52.14)	54.58 (54.06, 55.10)	59.55 (59.08, 60.02)	65.51 (65.02, 65.99)	72.36 (71.77, 72.95)	79.18 (78.42, 79.94)	83.60 (82.70, 84.49)	92.75 (91.48, 94.02)
2	22	45.83 (45.20, 46.46)	52.09 (51.66, 52.53)	55.23 (54.85, 55.60)	60.39 (60.06, 60.72)	66.58 (66.23, 66.92)	73.69 (73.25, 74.13)	80.78 (80.20, 81.35)	85.36 (84.67, 86.05)	94.87 (93.85, 95.89)
3	24	46.18 (45.62, 46.75)	52.66 (52.29, 53.03)	55.90 (55.59, 56.22)	61.25 (60.98, 61.51)	67.65 (67.37, 67.92)	75.00 (74.65, 75.36)	82.33 (81.85, 82.81)	87.08 (86.50, 87.66)	96.91 (96.03, 97.79)
4	26	46.58 (46.02, 47.14)	53.26 (52.88, 53.63)	56.60 (56.28, 56.91)	62.10 (61.83, 62.37)	68.70 (68.42, 68.97)	76.28 (75.93, 76.62)	83.83 (83.37, 84.28)	88.72 (88.17, 89.26)	98.85 (98.02, 99.67)
5	28	47.01 (46.43, 47.59)	53.86 (53.46, 54.26)	57.29 (56.95, 57.64)	62.94 (62.64, 63.24)	69.71 (69.41, 70.02)	77.49 (77.13, 77.86)	85.24 (84.78, 85.71)	90.26 (89.71, 90.81)	100.66 (99.85, 101.47)
6	30	47.47 (46.87, 48.07)	54.47 (54.05, 54.89)	57.98 (57.61, 58.35)	63.76 (63.43, 64.09)	70.68 (70.35, 71.01)	78.64 (78.25, 79.02)	86.56 (86.08, 87.04)	91.69 (91.14, 92.25)	102.33 (101.52, 103.14)
7	32	47.93 (47.32, 48.54)	55.07 (54.63, 55.50)	58.64 (58.26, 59.03)	64.53 (64.19, 64.88)	71.59 (71.24, 71.93)	79.69 (79.30, 80.09)	87.77 (87.29, 88.26)	93.00 (92.44, 93.56)	103.84 (103.03, 104.65)
8	34	48.40 (47.80, 49.00)	55.65 (55.22, 56.08)	59.28 (58.89, 59.66)	65.26 (64.91, 65.60)	72.42 (72.08, 72.77)	80.66 (80.27, 81.05)	88.86 (88.38, 89.34)	94.17 (93.62, 94.72)	105.18 (104.38, 105.97)
9	36	48.85 (48.27, 49.43)	56.19 (55.78, 56.61)	59.87 (59.50, 60.24)	65.92 (65.59, 66.26)	73.17 (72.84, 73.51)	81.51 (81.14, 81.89)	89.82 (89.36, 90.27)	95.19 (94.66, 95.72)	106.34 (105.56, 107.11)
10	38	49.29 (48.73, 49.84)	56.70 (56.31, 57.09)	60.41 (60.06, 60.76)	66.52 (66.20, 66.83)	73.84 (73.53, 74.15)	82.25 (81.91, 82.60)	90.64 (90.21, 91.06)	96.06 (95.57, 96.56)	107.31 (106.56, 108.06)
11	40	49.70 (49.17, 50.22)	57.15 (56.79, 57.52)	60.89 (60.57, 61.21)	67.04 (66.75, 67.33)	74.41 (74.13, 74.69)	82.88 (82.57, 83.19)	91.32 (90.93, 91.71)	96.78 (96.32, 97.24)	108.10 (107.38, 108.82)

	Age	_1st	_5th	_10th	_25th	_50th	_75th	_90th	_95th	_99th
12	42	50.06 (49.56, 50.56)	57.55 (57.21, 57.89)	61.30 (61.00, 61.60)	67.48 (67.21, 67.74)	74.88 (74.62, 75.13)	83.38 (83.10, 83.66)	91.85 (91.50, 92.21)	97.34 (96.91, 97.77)	108.71 (108.01, 109.40)
13	44	50.38 (49.90, 50.86)	57.88 (57.56, 58.21)	61.64 (61.36, 61.92)	67.83 (67.58, 68.07)	75.24 (75.01, 75.47)	83.76 (83.51, 84.01)	92.25 (91.92, 92.57)	97.74 (97.34, 98.14)	109.13 (108.45, 109.80)
14	46	50.65 (50.18, 51.12)	58.15 (57.84, 58.46)	61.90 (61.63, 62.18)	68.09 (67.85, 68.33)	75.50 (75.28, 75.71)	84.02 (83.79, 84.24)	92.50 (92.20, 92.80)	97.99 (97.61, 98.37)	109.38 (108.71, 110.04)
15	48	50.85 (50.39, 51.31)	58.34 (58.03, 58.65)	62.08 (61.81, 62.36)	68.26 (68.01, 68.50)	75.65 (75.44, 75.86)	84.15 (83.93, 84.37)	92.62 (92.33, 92.91)	98.10 (97.73, 98.47)	109.46 (108.79, 110.12)
16	50	50.99 (50.53, 51.45)	58.45 (58.13, 58.77)	62.18 (61.90, 62.47)	68.33 (68.08, 68.58)	75.70 (75.48, 75.92)	84.16 (83.94, 84.39)	92.60 (92.31, 92.89)	98.06 (97.69, 98.43)	109.38 (108.71, 110.05)
17	52	51.06 (50.60, 51.52)	58.48 (58.15, 58.81)	62.19 (61.89, 62.49)	68.31 (68.04, 68.57)	75.64 (75.41, 75.87)	84.07 (83.83, 84.30)	92.46 (92.16, 92.76)	97.89 (97.52, 98.27)	109.15 (108.48, 109.83)
18	54	51.05 (50.59, 51.52)	58.43 (58.09, 58.76)	62.12 (61.81, 62.43)	68.20 (67.92, 68.48)	75.48 (75.24, 75.73)	83.86 (83.62, 84.10)	92.20 (91.90, 92.51)	97.60 (97.22, 97.99)	108.80 (108.12, 109.48)
19	56	50.97 (50.50, 51.44)	58.29 (57.95, 58.64)	61.96 (61.64, 62.28)	68.00 (67.71, 68.29)	75.23 (74.98, 75.49)	83.55 (83.30, 83.81)	91.84 (91.53, 92.15)	97.20 (96.81, 97.59)	108.32 (107.63, 109.00)
20	58	50.81 (50.35, 51.28)	58.08 (57.73, 58.43)	61.72 (61.40, 62.04)	67.72 (67.42, 68.01)	74.90 (74.63, 75.16)	83.15 (82.89, 83.41)	91.38 (91.06, 91.70)	96.70 (96.31, 97.10)	107.74 (107.05, 108.42)
21	60	50.58 (50.13, 51.04)	57.79 (57.45, 58.14)	61.41 (61.09, 61.72)	67.35 (67.06, 67.64)	74.48 (74.22, 74.74)	82.67 (82.41, 82.93)	90.83 (90.52, 91.15)	96.12 (95.72, 96.51)	107.06 (106.37, 107.76)
22	62	50.28 (49.84, 50.73)	57.44 (57.11, 57.77)	61.02 (60.72, 61.33)	66.92 (66.64, 67.20)	73.99 (73.74, 74.25)	82.12 (81.87, 82.38)	90.22 (89.90, 90.54)	95.46 (95.07, 95.85)	106.32 (105.64, 107.01)
23	64	49.91 (49.48, 50.35)	57.02 (56.70, 57.33)	60.57 (60.28, 60.86)	66.43 (66.17, 66.69)	73.45 (73.21, 73.68)	81.51 (81.27, 81.75)	89.55 (89.24, 89.86)	94.75 (94.36, 95.14)	105.53 (104.85, 106.22)
24	66	49.49 (49.06, 49.91)	56.54 (56.24, 56.84)	60.07 (59.80, 60.34)	65.88 (65.64, 66.13)	72.85 (72.63, 73.07)	80.86 (80.63, 81.09)	88.84 (88.54, 89.14)	94.00 (93.62, 94.39)	104.71 (104.02, 105.39)
25	68	49.01 (48.59, 49.42)	56.01 (55.73, 56.29)	59.52 (59.27, 59.77)	65.30 (65.07, 65.52)	72.22 (72.02, 72.42)	80.18 (79.96, 80.39)	88.10 (87.81, 88.40)	93.24 (92.85, 93.62)	103.87 (103.19, 104.55)
26	70	48.48 (48.07, 48.89)	55.45 (55.18, 55.72)	58.94 (58.70, 59.17)	64.68 (64.48, 64.89)	71.57 (71.38, 71.75)	79.48 (79.27, 79.69)	87.37 (87.07, 87.66)	92.47 (92.09, 92.85)	103.04 (102.37, 103.72)

	Age	_1st	_5th	_10th	_25th	_50th	_75th	_90th	_95th	_99th
27	72	47.92 (47.51, 48.34)	54.86 (54.59, 55.13)	58.33 (58.10, 58.56)	64.05 (63.85, 64.25)	70.91 (70.73, 71.09)	78.79 (78.58, 79.00)	86.64 (86.34, 86.94)	91.72 (91.34, 92.10)	102.25 (101.57, 102.93)
28	74	47.34 (46.92, 47.76)	54.26 (53.99, 54.53)	57.72 (57.49, 57.96)	63.43 (63.22, 63.63)	70.26 (70.07, 70.46)	78.12 (77.90, 78.34)	85.95 (85.64, 86.26)	91.01 (90.62, 91.41)	101.51 (100.84, 102.19)
29	76	46.75 (46.31, 47.19)	53.66 (53.37, 53.95)	57.12 (56.87, 57.37)	62.82 (62.59, 63.04)	69.64 (69.43, 69.86)	77.49 (77.25, 77.74)	85.31 (84.99, 85.64)	90.37 (89.97, 90.78)	100.86 (100.18, 101.54)
30	78	46.16 (45.71, 46.62)	53.08 (52.77, 53.38)	56.54 (56.27, 56.81)	62.24 (61.99, 62.49)	69.08 (68.83, 69.32)	76.93 (76.66, 77.20)	84.76 (84.41, 85.10)	89.82 (89.40, 90.24)	100.32 (99.63, 101.00)
31	80	45.59 (45.12, 46.07)	52.53 (52.20, 52.85)	56.00 (55.71, 56.29)	61.72 (61.45, 61.99)	68.57 (68.30, 68.85)	76.45 (76.15, 76.76)	84.30 (83.93, 84.67)	89.38 (88.94, 89.82)	99.91 (99.22, 100.60)
32	82	45.06 (44.56, 45.55)	52.03 (51.69, 52.37)	55.52 (55.21, 55.83)	61.27 (60.98, 61.57)	68.16 (67.87, 68.46)	76.09 (75.75, 76.42)	83.98 (83.57, 84.38)	89.08 (88.61, 89.55)	99.67 (98.95, 100.39)
33	84	44.57 (44.05, 45.09)	51.60 (51.24, 51.97)	55.12 (54.80, 55.45)	60.92 (60.61, 61.23)	67.87 (67.55, 68.19)	75.85 (75.49, 76.22)	83.81 (83.36, 84.26)	88.96 (88.44, 89.48)	99.63 (98.86, 100.40)
34	86	44.16 (43.59, 44.72)	51.27 (50.87, 51.66)	54.83 (54.48, 55.17)	60.69 (60.36, 61.01)	67.71 (67.36, 68.06)	75.79 (75.37, 76.20)	83.83 (83.30, 84.35)	89.03 (88.42, 89.65)	99.83 (98.94, 100.71)
35	88	43.83 (43.18, 44.47)	51.04 (50.59, 51.49)	54.65 (54.25, 55.04)	60.59 (60.23, 60.95)	67.72 (67.32, 68.11)	75.91 (75.40, 76.41)	84.06 (83.42, 84.71)	89.35 (88.58, 90.11)	100.29 (99.22, 101.36)
36	90	43.60 (42.81, 44.39)	50.94 (50.37, 51.50)	54.61 (54.12, 55.10)	60.66 (60.22, 61.10)	67.91 (67.43, 68.40)	76.25 (75.61, 76.88)	84.55 (83.72, 85.39)	89.92 (88.94, 90.91)	101.06 (99.70, 102.43)

Female diastolic blood pressure centiles with 95% CI

	Age	_1st	_5th	_10th	_25th	_50th	_75th	_90th	_95th	_99th
1	20	45.22 (44.71, 45.73)	50.96 (50.58, 51.33)	53.83 (53.49, 54.16)	58.60 (58.29, 58.92)	64.63 (64.29, 64.97)	71.84 (71.40, 72.28)	79.17 (78.57, 79.78)	83.99 (83.25, 84.73)	94.15 (93.05, 95.26)
2	22	45.28 (44.83, 45.73)	51.13 (50.80, 51.46)	54.05 (53.76, 54.35)	58.92 (58.64, 59.21)	65.07 (64.77, 65.37)	72.42 (72.05, 72.79)	79.90 (79.39, 80.40)	84.81 (84.19, 85.42)	95.17 (94.23, 96.11)
3	24	45.35 (44.93, 45.77)	51.31 (51.02, 51.61)	54.29 (54.03, 54.56)	59.25 (59.00, 59.51)	65.51 (65.25, 65.78)	73.00 (72.68, 73.32)	80.62 (80.19, 81.05)	85.62 (85.09, 86.15)	96.18 (95.34, 97.01)

For peer review only - http://bmjopen.bmj.com/site/about/guidelines.xhtml

	Age	_1st	_5th	_10th	_25th	_50th	_75th	_90th	_95th	_99th
4	26	45.44 (45.04, 45.84)	51.50 (51.23, 51.77)	54.53 (54.29, 54.77)	59.58 (59.36, 59.81)	65.95 (65.72, 66.19)	73.57 (73.28, 73.85)	81.32 (80.94, 81.70)	86.41 (85.94, 86.89)	97.15 (96.38, 97.92)
5	28	45.53 (45.14, 45.92)	51.69 (51.44, 51.95)	54.77 (54.56, 54.99)	59.91 (59.70, 60.11)	66.38 (66.17, 66.59)	74.12 (73.86, 74.38)	82.00 (81.64, 82.36)	87.17 (86.73, 87.62)	98.09 (97.35, 98.82)
6	30	45.63 (45.24, 46.02)	51.89 (51.64, 52.13)	55.01 (54.81, 55.21)	60.22 (60.04, 60.40)	66.79 (66.59, 66.99)	74.65 (74.39, 74.90)	82.64 (82.30, 82.99)	87.89 (87.46, 88.33)	98.97 (98.25, 99.69)
7	32	45.74 (45.35, 46.13)	52.08 (51.84, 52.32)	55.25 (55.06, 55.44)	60.52 (60.35, 60.69)	67.18 (66.99, 67.37)	75.14 (74.90, 75.38)	83.24 (82.91, 83.58)	88.57 (88.14, 88.99)	99.79 (99.09, 100.49)
8	34	45.85 (45.46, 46.25)	52.27 (52.03, 52.50)	55.47 (55.29, 55.66)	60.81 (60.65, 60.97)	67.55 (67.36, 67.73)	75.60 (75.36, 75.84)	83.80 (83.46, 84.13)	89.18 (88.77, 89.59)	100.54 (99.85, 101.23)
9	36	45.97 (45.58, 46.37)	52.45 (52.21, 52.68)	55.68 (55.50, 55.87)	61.08 (60.91, 61.24)	67.88 (67.70, 68.07)	76.01 (75.78, 76.25)	84.29 (83.97, 84.62)	89.73 (89.33, 90.14)	101.20 (100.53, 101.88)
10	38	46.09 (45.69, 46.48)	52.62 (52.38, 52.86)	55.88 (55.69, 56.07)	61.32 (61.15, 61.49)	68.18 (68.00, 68.37)	76.38 (76.15, 76.62)	84.73 (84.42, 85.05)	90.22 (89.83, 90.61)	101.78 (101.13, 102.44)
11	40	46.20 (45.81, 46.60)	52.78 (52.54, 53.02)	56.07 (55.87, 56.26)	61.54 (61.37, 61.72)	68.45 (68.26, 68.64)	76.70 (76.47, 76.94)	85.11 (84.80, 85.42)	90.63 (90.25, 91.01)	102.28 (101.64, 102.92)
12	42	46.32 (45.91, 46.72)	52.93 (52.68, 53.18)	56.23 (56.03, 56.43)	61.73 (61.55, 61.92)	68.68 (68.48, 68.87)	76.98 (76.74, 77.21)	85.42 (85.12, 85.73)	90.98 (90.60, 91.35)	102.68 (102.05, 103.31)
13	44	46.43 (46.02, 46.83)	53.06 (52.80, 53.32)	56.37 (56.16, 56.59)	61.90 (61.71, 62.09)	68.87 (68.67, 69.06)	77.19 (76.96, 77.43)	85.67 (85.38, 85.97)	91.24 (90.88, 91.61)	102.99 (102.36, 103.62)
14	46	46.53 (46.11, 46.94)	53.17 (52.91, 53.44)	56.50 (56.28, 56.71)	62.03 (61.83, 62.23)	69.01 (68.81, 69.22)	77.36 (77.13, 77.59)	85.86 (85.56, 86.16)	91.44 (91.08, 91.80)	103.21 (102.59, 103.84)
15	48	46.62 (46.20, 47.04)	53.27 (53.00, 53.54)	56.59 (56.37, 56.82)	62.13 (61.93, 62.33)	69.12 (68.92, 69.33)	77.48 (77.24, 77.71)	85.98 (85.68, 86.28)	91.57 (91.20, 91.93)	103.35 (102.72, 103.98)
16	50	46.69 (46.27, 47.12)	53.34 (53.07, 53.62)	56.67 (56.44, 56.90)	62.20 (62.00, 62.41)	69.19 (68.99, 69.40)	77.54 (77.31, 77.77)	86.04 (85.74, 86.34)	91.63 (91.26, 92.00)	103.41 (102.76, 104.05)
17	52	46.76 (46.33, 47.18)	53.40 (53.12, 53.67)	56.72 (56.49, 56.95)	62.24 (62.04, 62.45)	69.22 (69.02, 69.43)	77.56 (77.32, 77.79)	86.05 (85.74, 86.35)	91.62 (91.25, 92.00)	103.38 (102.73, 104.04)
18	54	46.80 (46.38, 47.23)	53.43 (53.16, 53.70)	56.74 (56.51, 56.97)	62.25 (62.05, 62.46)	69.21 (69.01, 69.42)	77.53 (77.30, 77.77)	86.00 (85.69, 86.31)	91.56 (91.18, 91.95)	103.30 (102.63, 103.96)

For peer review only - http://bmjopen.bmj.com/site/about/guidelines.xhtml

	Age	_1st	_5th	_10th	_25th	_50th	_75th	_90th	_95th	_99th
19	56	46.83 (46.41, 47.25)	53.44 (53.17, 53.70)	56.74 (56.51, 56.96)	62.23 (62.04, 62.43)	69.17 (68.97, 69.37)	77.46 (77.23, 77.70)	85.91 (85.60, 86.22)	91.45 (91.06, 91.84)	103.15 (102.47, 103.83)
20	58	46.84 (46.43, 47.25)	53.42 (53.16, 53.68)	56.71 (56.49, 56.92)	62.19 (61.99, 62.38)	69.10 (68.90, 69.30)	77.36 (77.13, 77.60)	85.77 (85.46, 86.09)	91.30 (90.90, 91.69)	102.95 (102.27, 103.64)
21	60	46.82 (46.43, 47.22)	53.38 (53.13, 53.63)	56.65 (56.45, 56.86)	62.11 (61.92, 62.30)	69.00 (68.80, 69.19)	77.23 (76.99, 77.46)	85.61 (85.29, 85.92)	91.11 (90.71, 91.51)	102.72 (102.02, 103.41)
22	62	46.79 (46.40, 47.17)	53.32 (53.08, 53.55)	56.58 (56.38, 56.77)	62.01 (61.83, 62.19)	68.87 (68.68, 69.06)	77.07 (76.83, 77.30)	85.41 (85.09, 85.73)	90.89 (90.49, 91.30)	102.45 (101.76, 103.15)
23	64	46.72 (46.35, 47.09)	53.23 (53.00, 53.45)	56.48 (56.29, 56.66)	61.89 (61.72, 62.06)	68.72 (68.53, 68.91)	76.89 (76.65, 77.12)	85.20 (84.88, 85.52)	90.66 (90.26, 91.07)	102.18 (101.48, 102.88)
24	66	46.63 (46.28, 46.99)	53.12 (52.90, 53.33)	56.35 (56.18, 56.53)	61.75 (61.58, 61.92)	68.56 (68.37, 68.75)	76.70 (76.46, 76.93)	84.98 (84.66, 85.31)	90.43 (90.02, 90.83)	101.90 (101.20, 102.61)
25	68	46.52 (46.17, 46.87)	52.98 (52.78, 53.19)	56.21 (56.04, 56.38)	61.60 (61.43, 61.76)	68.39 (68.20, 68.58)	76.50 (76.26, 76.74)	84.77 (84.44, 85.09)	90.19 (89.78, 90.61)	101.64 (100.93, 102.35)
26	70	46.38 (46.03, 46.73)	52.83 (52.63, 53.04)	56.06 (55.89, 56.22)	61.43 (61.27, 61.59)	68.21 (68.02, 68.40)	76.31 (76.07, 76.56)	84.56 (84.23, 84.90)	89.98 (89.56, 90.40)	101.41 (100.69, 102.13)
27	72	46.21 (45.85, 46.57)	52.66 (52.45, 52.87)	55.89 (55.71, 56.06)	61.26 (61.09, 61.42)	68.03 (67.85, 68.22)	76.13 (75.89, 76.38)	84.38 (84.04, 84.72)	89.80 (89.37, 90.23)	101.23 (100.50, 101.95)
28	74	46.02 (45.65, 46.38)	52.48 (52.25, 52.70)	55.70 (55.52, 55.88)	61.08 (60.92, 61.25)	67.87 (67.68, 68.06)	75.98 (75.73, 76.23)	84.24 (83.89, 84.58)	89.66 (89.23, 90.10)	101.10 (100.37, 101.84)
29	76	45.79 (45.40, 46.18)	52.28 (52.04, 52.51)	55.51 (55.32, 55.71)	60.91 (60.74, 61.08)	67.72 (67.53, 67.91)	75.86 (75.61, 76.11)	84.14 (83.79, 84.50)	89.59 (89.14, 90.03)	101.07 (100.32, 101.81)
30	78	45.54 (45.13, 45.96)	52.06 (51.80, 52.32)	55.32 (55.11, 55.53)	60.75 (60.57, 60.92)	67.60 (67.41, 67.78)	75.78 (75.53, 76.03)	84.11 (83.76, 84.47)	89.59 (89.14, 90.04)	101.13 (100.38, 101.88)
31	80	45.27 (44.83, 45.71)	51.84 (51.56, 52.13)	55.13 (54.89, 55.36)	60.60 (60.41, 60.79)	67.51 (67.32, 67.69)	75.76 (75.51, 76.00)	84.16 (83.81, 84.51)	89.68 (89.24, 90.13)	101.32 (100.57, 102.08)
32	82	44.96 (44.49, 45.44)	51.61 (51.30, 51.93)	54.94 (54.68, 55.19)	60.47 (60.27, 60.67)	67.46 (67.27, 67.65)	75.81 (75.56, 76.05)	84.31 (83.96, 84.66)	89.89 (89.45, 90.33)	101.67 (100.92, 102.41)
33	84	44.63 (44.12, 45.14)	51.38 (51.03, 51.73)	54.75 (54.47, 55.04)	60.37 (60.15, 60.59)	67.46 (67.27, 67.66)	75.94 (75.69, 76.18)	84.57 (84.22, 84.91)	90.23 (89.79, 90.67)	102.19 (101.45, 102.92)

For peer review only - http://bmjopen.bmj.com/site/about/guidelines.xhtml

	Age	_1st	_5th	_10th	_25th	_50th	_75th	_90th	_95th	_99th
34	86	44.27 (43.72, 44.81)	51.14 (50.76, 51.53)	54.58 (54.26, 54.90)	60.31 (60.06, 60.55)	67.53 (67.31, 67.75)	76.17 (75.91, 76.43)	84.96 (84.60, 85.32)	90.73 (90.29, 91.18)	102.91 (102.18, 103.65)
35	88	43.87 (43.27, 44.46)	50.90 (50.47, 51.34)	54.42 (54.06, 54.79)	60.28 (59.99, 60.57)	67.67 (67.42, 67.93)	76.51 (76.21, 76.81)	85.51 (85.11, 85.90)	91.42 (90.93, 91.90)	103.88 (103.12, 104.64)
36	90	43.43 (42.76, 44.10)	50.66 (50.17, 51.16)	54.28 (53.86, 54.70)	60.30 (59.96, 60.64)	67.90 (67.59, 68.22)	76.99 (76.61, 77.36)	86.24 (85.75, 86.73)	92.32 (91.73, 92.90)	105.13 (104.27, 105.99)

Male pulse pressure centiles with 95% CI

	Age	1st	5th	10th	25th	50th	75th	90th	95th	99th
1	20	31.75 (30.29, 33.22)	39.16 (38.33, 39.99)	42.78 (42.07, 43.49)	48.59 (47.94, 49.25)	55.24 (54.63, 55.85)	62.62 (62.06, 63.19)	69.94 (69.29, 70.58)	74.70 (73.92, 75.48)	84.71 (83.29, 86.13)
2	22	31.54 (30.33, 32.75)	38.84 (38.21, 39.48)	42.40 (41.87, 42.93)	48.12 (47.62, 48.61)	54.69 (54.21, 55.18)	62.06 (61.55, 62.56)	69.38 (68.77, 69.99)	74.17 (73.44, 74.90)	84.28 (83.02, 85.54)
3	24	31.28 (30.24, 32.32)	38.47 (37.96, 38.99)	41.97 (41.55, 42.38)	47.59 (47.19, 47.99)	54.09 (53.67, 54.51)	61.43 (60.94, 61.93)	68.78 (68.12, 69.43)	73.59 (72.79, 74.39)	83.81 (82.46, 85.15)
4	26	30.95 (29.96, 31.95)	38.04 (37.51, 38.57)	41.47 (41.04, 41.91)	47.00 (46.59, 47.41)	53.43 (53.00, 53.87)	60.75 (60.22, 61.29)	68.12 (67.41, 68.82)	72.96 (72.10, 73.82)	83.29 (81.85, 84.74)
5	28	30.57 (29.63, 31.52)	37.56 (37.05, 38.06)	40.93 (40.52, 41.35)	46.37 (45.99, 46.74)	52.74 (52.34, 53.14)	60.05 (59.56, 60.54)	67.43 (66.77, 68.09)	72.31 (71.49, 73.14)	82.78 (81.31, 84.24)
6	30	30.16 (29.32, 30.99)	37.05 (36.62, 37.47)	40.37 (40.02, 40.72)	45.72 (45.38, 46.06)	52.04 (51.66, 52.41)	59.34 (58.88, 59.80)	66.76 (66.15, 67.38)	71.69 (70.89, 72.49)	82.30 (80.80, 83.80)
7	32	29.72 (28.96, 30.48)	36.53 (36.12, 36.94)	39.81 (39.44, 40.18)	45.08 (44.69, 45.47)	51.36 (50.93, 51.80)	58.68 (58.18, 59.18)	66.15 (65.50, 66.80)	71.13 (70.31, 71.96)	81.92 (80.35, 83.49)
8	34	29.29 (28.56, 30.02)	36.04 (35.64, 36.43)	39.28 (38.93, 39.62)	44.49 (44.14, 44.84)	50.75 (50.37, 51.13)	58.10 (57.65, 58.54)	65.64 (65.04, 66.24)	70.69 (69.90, 71.47)	81.67 (80.10, 83.24)
9	36	28.89 (28.17, 29.62)	35.60 (35.19, 36.02)	38.81 (38.45, 39.17)	43.98 (43.65, 44.31)	50.24 (49.90, 50.58)	57.64 (57.24, 58.04)	65.27 (64.72, 65.83)	70.40 (69.65, 71.14)	81.62 (80.09, 83.15)
10	38	28.53 (27.82, 29.25)	35.23 (34.78, 35.67)	38.42 (38.04, 38.81)	43.57 (43.22, 43.92)	49.85 (49.50, 50.21)	57.32 (56.91, 57.74)	65.07 (64.51, 65.63)	70.29 (69.56, 71.03)	81.79 (80.31, 83.26)

For peer review only - http://bmjopen.bmj.com/site/about/guidelines.xhtml

	Age	1st	5th	10th	25th	50th	75th	90th	95th	99th
11	40	28.18 (27.51, 28.85)	34.89 (34.44, 35.34)	38.09 (37.69, 38.49)	43.24 (42.87, 43.61)	49.57 (49.19, 49.96)	57.15 (56.70, 57.60)	65.04 (64.44, 65.64)	70.38 (69.62, 71.14)	82.18 (80.74, 83.63)
12	42	27.82 (27.18, 28.45)	34.58 (34.13, 35.03)	37.79 (37.38, 38.20)	42.98 (42.59, 43.37)	49.40 (48.98, 49.81)	57.11 (56.62, 57.61)	65.18 (64.54, 65.82)	70.66 (69.86, 71.46)	82.82 (81.38, 84.26)
13	44	27.44 (26.81, 28.07)	34.29 (33.84, 34.75)	37.54 (37.13, 37.96)	42.79 (42.40, 43.18)	49.33 (48.91, 49.74)	57.22 (56.72, 57.72)	65.50 (64.85, 66.15)	71.14 (70.33, 71.94)	83.69 (82.25, 85.13)
14	46	27.09 (26.43, 27.74)	34.05 (33.59, 34.51)	37.35 (36.94, 37.76)	42.69 (42.32, 43.06)	49.38 (48.99, 49.77)	57.49 (57.02, 57.96)	66.00 (65.39, 66.62)	71.81 (71.04, 72.58)	84.77 (83.35, 86.19)
15	48	26.77 (26.09, 27.46)	33.86 (33.40, 34.32)	37.23 (36.83, 37.63)	42.69 (42.34, 43.05)	49.56 (49.20, 49.93)	57.91 (57.47, 58.35)	66.69 (66.11, 67.26)	72.67 (71.95, 73.40)	86.02 (84.64, 87.40)
16	50	26.52 (25.81, 27.23)	33.75 (33.30, 34.21)	37.20 (36.81, 37.59)	42.80 (42.46, 43.15)	49.88 (49.52, 50.24)	58.49 (58.06, 58.93)	67.54 (66.98, 68.09)	73.69 (73.00, 74.38)	87.38 (86.02, 88.74)
17	52	26.37 (25.65, 27.09)	33.73 (33.29, 34.18)	37.26 (36.89, 37.64)	43.03 (42.69, 43.36)	50.33 (49.97, 50.69)	59.21 (58.77, 59.66)	68.52 (67.96, 69.08)	74.83 (74.14, 75.52)	88.79 (87.43, 90.15)
18	54	26.33 (25.62, 27.04)	33.81 (33.37, 34.24)	37.42 (37.05, 37.79)	43.36 (43.03, 43.68)	50.90 (50.54, 51.26)	60.06 (59.60, 60.51)	69.61 (69.03, 70.18)	76.05 (75.34, 76.75)	90.15 (88.78, 91.52)
19	56	26.39 (25.70, 27.09)	33.96 (33.53, 34.39)	37.66 (37.30, 38.02)	43.78 (43.46, 44.09)	51.56 (51.20, 51.91)	60.98 (60.53, 61.43)	70.74 (70.16, 71.33)	77.28 (76.55, 78.00)	91.43 (90.06, 92.79)
20	58	26.49 (25.80, 27.17)	34.14 (33.71, 34.58)	37.93 (37.57, 38.30)	44.23 (43.91, 44.55)	52.25 (51.91, 52.60)	61.93 (61.49, 62.37)	71.89 (71.30, 72.48)	78.49 (77.75, 79.23)	92.59 (91.25, 93.93)
21	60	26.58 (25.90, 27.26)	34.32 (33.88, 34.76)	38.19 (37.82, 38.56)	44.68 (44.36, 45.00)	52.95 (52.61, 53.29)	62.88 (62.44, 63.32)	73.01 (72.41, 73.61)	79.66 (78.91, 80.42)	93.68 (92.38, 94.98)
22	62	26.71 (26.04, 27.39)	34.54 (34.09, 34.99)	38.51 (38.13, 38.89)	45.20 (44.87, 45.52)	53.71 (53.38, 54.05)	63.89 (63.46, 64.33)	74.18 (73.58, 74.79)	80.88 (80.13, 81.63)	94.79 (93.54, 96.04)
23	64	26.94 (26.28, 27.60)	34.86 (34.41, 35.32)	38.93 (38.54, 39.32)	45.82 (45.49, 46.15)	54.58 (54.26, 54.91)	65.01 (64.59, 65.42)	75.46 (74.88, 76.04)	82.21 (81.49, 82.93)	96.03 (94.86, 97.20)
24	66	27.24 (26.62, 27.86)	35.28 (34.84, 35.73)	39.45 (39.07, 39.84)	46.55 (46.21, 46.88)	55.57 (55.24, 55.89)	66.23 (65.84, 66.63)	76.86 (76.31, 77.40)	83.66 (82.98, 84.33)	97.42 (96.33, 98.52)
25	68	27.58 (26.98, 28.18)	35.76 (35.33, 36.19)	40.04 (39.66, 40.41)	47.35 (47.02, 47.68)	56.62 (56.30, 56.94)	67.53 (67.14, 67.92)	78.33 (77.81, 78.86)	85.19 (84.55, 85.84)	98.95 (97.89, 100.01)

For peer review only - http://bmjopen.bmj.com/site/about/guidelines.xhtml

	Age	1st	5th	10th	25th	50th	75th	90th	95th	99th
26	70	27.85 (27.23, 28.47)	36.19 (35.76, 36.62)	40.59 (40.21, 40.96)	48.11 (47.79, 48.44)	57.63 (57.31, 57.95)	68.80 (68.41, 69.18)	79.78 (79.25, 80.31)	86.71 (86.05, 87.37)	100.50 (99.41, 101.59)
27	72	27.98 (27.30, 28.65)	36.50 (36.04, 36.96)	41.01 (40.62, 41.40)	48.76 (48.43, 49.10)	58.54 (58.20, 58.87)	69.94 (69.53, 70.35)	81.11 (80.55, 81.67)	88.13 (87.43, 88.82)	101.97 (100.83, 103.12)
28	74	27.93 (27.22, 28.63)	36.65 (36.17, 37.13)	41.29 (40.88, 41.70)	49.27 (48.90, 49.64)	59.29 (58.92, 59.67)	70.95 (70.51, 71.39)	82.32 (81.73, 82.90)	89.42 (88.70, 90.15)	103.37 (102.17, 104.56)
29	76	27.72 (27.03, 28.42)	36.65 (36.18, 37.12)	41.42 (41.01, 41.82)	49.62 (49.25, 50.00)	59.91 (59.52, 60.29)	71.82 (71.37, 72.26)	83.38 (82.78, 83.98)	90.60 (89.84, 91.35)	104.67 (103.41, 105.92)
30	78	27.42 (26.71, 28.12)	36.54 (36.05, 37.04)	41.43 (41.01, 41.85)	49.86 (49.47, 50.24)	60.38 (59.98, 60.78)	72.54 (72.07, 73.02)	84.32 (83.65, 84.99)	91.64 (90.78, 92.50)	105.86 (104.46, 107.27)
31	80	27.13 (26.35, 27.91)	36.43 (35.85, 37.00)	41.42 (40.91, 41.93)	50.04 (49.57, 50.50)	60.79 (60.32, 61.26)	73.19 (72.63, 73.75)	85.17 (84.39, 85.95)	92.60 (91.61, 93.59)	106.99 (105.41, 108.57)
32	82	26.96 (26.14, 27.78)	36.40 (35.77, 37.02)	41.47 (40.91, 42.04)	50.25 (49.73, 50.77)	61.21 (60.69, 61.73)	73.83 (73.23, 74.42)	86.00 (85.18, 86.83)	93.54 (92.50, 94.58)	108.10 (106.45, 109.75)
33	84	26.93 (26.07, 27.78)	36.46 (35.83, 37.09)	41.60 (41.04, 42.16)	50.50 (49.98, 51.03)	61.63 (61.11, 62.16)	74.46 (73.86, 75.05)	86.82 (86.00, 87.64)	94.46 (93.43, 95.49)	109.19 (107.55, 110.82)
34	86	26.97 (25.99, 27.95)	36.57 (35.90, 37.24)	41.76 (41.19, 42.33)	50.76 (50.23, 51.29)	62.04 (61.49, 62.59)	75.05 (74.41, 75.69)	87.58 (86.70, 88.47)	95.32 (94.21, 96.44)	110.21 (108.50, 111.92)
35	88	27.03 (25.79, 28.27)	36.67 (35.88, 37.45)	41.89 (41.26, 42.51)	50.95 (50.42, 51.49)	62.35 (61.79, 62.92)	75.52 (74.80, 76.24)	88.20 (87.15, 89.25)	96.02 (94.71, 97.34)	111.05 (109.09, 113.02)
36	90	27.07 (25.45, 28.70)	36.72 (35.70, 37.74)	41.96 (41.18, 42.74)	51.07 (50.45, 51.69)	62.56 (61.94, 63.18)	75.85 (75.03, 76.66)	88.65 (87.41, 89.89)	96.54 (94.96, 98.11)	111.68 (109.35, 114.00)

Female PP centiles with 95% CI

	Age	1st	5th	10th	25th	50th	75th	90th	95th	99th
1	20	27.29 (26.29, 28.28)	33.96 (33.35, 34.58)	37.06 (36.57, 37.55)	41.94 (41.54, 42.34)	47.71 (47.21, 48.21)	54.50 (53.81, 55.19)	61.58 (60.71, 62.45)	66.42 (65.43, 67.42)	77.33 (75.64, 79.01)
2	22	27.09 (26.25, 27.93)	33.72 (33.20, 34.24)	36.80 (36.38, 37.22)	41.65 (41.32, 41.98)	47.40 (47.04, 47.76)	54.18 (53.70, 54.65)	61.26 (60.63, 61.88)	66.10 (65.34, 66.86)	77.01 (75.55, 78.46)

	Age	1st	5th	10th	25th	50th	75th	90th	95th	99th
3	24	26.89 (26.14, 27.64)	33.48 (33.02, 33.94)	36.54 (36.17, 36.91)	41.37 (41.08, 41.66)	47.11 (46.82, 47.40)	53.88 (53.52, 54.25)	60.97 (60.45, 61.49)	65.82 (65.14, 66.49)	76.74 (75.40, 78.09)
4	26	26.70 (26.00, 27.40)	33.26 (32.84, 33.68)	36.31 (35.97, 36.65)	41.12 (40.85, 41.39)	46.86 (46.59, 47.13)	53.64 (53.30, 53.98)	60.74 (60.23, 61.25)	65.61 (64.92, 66.29)	76.57 (75.26, 77.88)
5	28	26.54 (25.86, 27.21)	33.08 (32.68, 33.48)	36.12 (35.80, 36.43)	40.93 (40.66, 41.19)	46.67 (46.40, 46.94)	53.49 (53.13, 53.84)	60.63 (60.09, 61.16)	65.52 (64.81, 66.23)	76.54 (75.25, 77.84)
6	30	26.41 (25.75, 27.07)	32.95 (32.56, 33.34)	35.99 (35.68, 36.30)	40.81 (40.55, 41.07)	46.59 (46.32, 46.86)	53.46 (53.10, 53.82)	60.67 (60.12, 61.21)	65.60 (64.88, 66.32)	76.73 (75.46, 78.01)
7	32	26.30 (25.66, 26.95)	32.87 (32.48, 33.26)	35.92 (35.61, 36.24)	40.77 (40.50, 41.04)	46.61 (46.34, 46.88)	53.57 (53.23, 53.91)	60.87 (60.35, 61.39)	65.88 (65.19, 66.57)	77.17 (75.91, 78.43)
8	34	26.20 (25.58, 26.83)	32.82 (32.43, 33.21)	35.90 (35.57, 36.23)	40.80 (40.51, 41.09)	46.73 (46.45, 47.01)	53.81 (53.49, 54.14)	61.26 (60.77, 61.74)	66.36 (65.71, 67.01)	77.86 (76.62, 79.11)
9	36	26.09 (25.46, 26.71)	32.79 (32.39, 33.18)	35.91 (35.57, 36.26)	40.89 (40.57, 41.20)	46.94 (46.65, 47.23)	54.19 (53.88, 54.51)	61.82 (61.37, 62.27)	67.05 (66.44, 67.66)	78.83 (77.56, 80.11)
10	38	25.96 (25.29, 26.62)	32.77 (32.36, 33.17)	35.95 (35.59, 36.31)	41.03 (40.70, 41.36)	47.25 (46.95, 47.54)	54.71 (54.41, 55.01)	62.56 (62.15, 62.98)	67.95 (67.35, 68.55)	80.07 (78.66, 81.49)
11	40	25.81 (25.07, 26.55)	32.76 (32.35, 33.17)	36.02 (35.65, 36.38)	41.23 (40.90, 41.56)	47.64 (47.35, 47.94)	55.36 (55.06, 55.66)	63.48 (63.05, 63.92)	69.05 (68.38, 69.71)	81.56 (79.84, 83.29)
12	42	25.68 (24.86, 26.51)	32.79 (32.37, 33.21)	36.13 (35.77, 36.49)	41.50 (41.18, 41.82)	48.14 (47.85, 48.43)	56.14 (55.82, 56.47)	64.57 (64.05, 65.09)	70.33 (69.52, 71.15)	83.27 (81.15, 85.39)
13	44	25.62 (24.75, 26.50)	32.89 (32.47, 33.31)	36.33 (35.98, 36.68)	41.86 (41.56, 42.17)	48.74 (48.45, 49.03)	57.06 (56.69, 57.43)	65.80 (65.19, 66.42)	71.77 (70.82, 72.72)	85.12 (82.74, 87.50)
14	46	25.65 (24.75, 26.55)	33.08 (32.65, 33.52)	36.61 (36.26, 36.96)	42.33 (42.03, 42.62)	49.46 (49.15, 49.76)	58.09 (57.67, 58.51)	67.15 (66.47, 67.83)	73.32 (72.30, 74.33)	87.04 (84.66, 89.43)
15	48	25.79 (24.85, 26.72)	33.37 (32.89, 33.84)	36.99 (36.62, 37.36)	42.88 (42.58, 43.18)	50.27 (49.94, 50.59)	59.21 (58.74, 59.67)	68.56 (67.83, 69.30)	74.91 (73.89, 75.94)	88.95 (86.75, 91.16)
16	50	26.01 (25.03, 27.00)	33.73 (33.21, 34.26)	37.45 (37.05, 37.84)	43.51 (43.21, 43.82)	51.15 (50.81, 51.48)	60.38 (59.88, 60.89)	70.01 (69.23, 70.79)	76.51 (75.47, 77.55)	90.80 (88.81, 92.78)
17	52	26.31 (25.27, 27.35)	34.16 (33.59, 34.73)	37.97 (37.55, 38.39)	44.22 (43.91, 44.52)	52.09 (51.76, 52.43)	61.61 (61.08, 62.14)	71.48 (70.66, 72.30)	78.12 (77.06, 79.17)	92.58 (90.77, 94.38)

For peer review only - http://bmjopen.bmj.com/site/about/guidelines.xhtml

	Age	1st	5th	10th	25th	50th	75th	90th	95th	99th
18	54	26.65 (25.59, 27.71)	34.64 (34.03, 35.24)	38.54 (38.09, 38.99)	44.97 (44.65, 45.29)	53.09 (52.76, 53.42)	62.87 (62.34, 63.40)	72.98 (72.15, 73.80)	79.72 (78.67, 80.78)	94.31 (92.64, 95.97)
19	56	27.01 (25.99, 28.03)	35.14 (34.54, 35.74)	39.15 (38.69, 39.60)	45.77 (45.43, 46.11)	54.13 (53.79, 54.46)	64.17 (63.64, 64.69)	74.48 (73.66, 75.30)	81.33 (80.30, 82.36)	96.00 (94.44, 97.56)
20	58	27.38 (26.43, 28.33)	35.66 (35.10, 36.23)	39.78 (39.33, 40.22)	46.60 (46.24, 46.96)	55.20 (54.85, 55.55)	65.49 (64.97, 66.00)	76.00 (75.21, 76.79)	82.94 (81.94, 83.93)	97.66 (96.17, 99.16)
21	60	27.76 (26.88, 28.63)	36.21 (35.68, 36.73)	40.43 (40.00, 40.87)	47.47 (47.10, 47.84)	56.31 (55.94, 56.68)	66.84 (66.34, 67.35)	77.55 (76.81, 78.28)	84.57 (83.65, 85.49)	99.34 (97.93, 100.75)
22	62	28.14 (27.32, 28.96)	36.78 (36.28, 37.28)	41.12 (40.70, 41.55)	48.38 (47.99, 48.77)	57.46 (57.08, 57.85)	68.24 (67.75, 68.73)	79.13 (78.45, 79.80)	86.23 (85.40, 87.05)	101.05 (99.76, 102.35)
23	64	28.54 (27.74, 29.34)	37.37 (36.88, 37.86)	41.84 (41.42, 42.26)	49.31 (48.92, 49.70)	58.64 (58.24, 59.03)	69.64 (69.16, 70.12)	80.70 (80.07, 81.33)	87.87 (87.12, 88.63)	102.75 (101.55, 103.94)
24	66	28.96 (28.17, 29.76)	37.99 (37.51, 38.47)	42.58 (42.17, 42.98)	50.26 (49.88, 50.63)	59.80 (59.42, 60.19)	71.01 (70.55, 71.47)	82.22 (81.64, 82.81)	89.47 (88.78, 90.15)	104.37 (103.28, 105.46)
25	68	29.38 (28.58, 30.19)	38.60 (38.12, 39.07)	43.30 (42.91, 43.69)	51.19 (50.84, 51.54)	60.95 (60.58, 61.31)	72.35 (71.92, 72.79)	83.71 (83.16, 84.26)	91.01 (90.37, 91.65)	105.95 (104.93, 106.96)
26	70	29.75 (28.92, 30.58)	39.16 (38.68, 39.65)	43.99 (43.59, 44.39)	52.08 (51.73, 52.44)	62.05 (61.68, 62.42)	73.65 (73.20, 74.10)	85.15 (84.59, 85.72)	92.52 (91.85, 93.18)	107.50 (106.43, 108.58)
27	72	30.01 (29.12, 30.89)	39.63 (39.10, 40.16)	44.57 (44.13, 45.02)	52.88 (52.47, 53.30)	63.07 (62.64, 63.51)	74.88 (74.37, 75.38)	86.54 (85.90, 87.18)	93.98 (93.22, 94.74)	109.05 (107.74, 110.35)
28	74	30.08 (29.12, 31.04)	39.92 (39.34, 40.51)	45.00 (44.50, 45.50)	53.53 (53.06, 54.01)	63.96 (63.47, 64.46)	75.99 (75.42, 76.56)	87.84 (87.11, 88.56)	95.37 (94.48, 96.26)	110.55 (108.98, 112.12)
29	76	29.93 (28.92, 30.95)	40.01 (39.37, 40.65)	45.22 (44.68, 45.76)	53.99 (53.50, 54.47)	64.66 (64.15, 65.17)	76.93 (76.33, 77.53)	88.97 (88.19, 89.74)	96.60 (95.65, 97.56)	111.93 (110.29, 113.57)
30	78	29.60 (28.51, 30.70)	39.90 (39.18, 40.63)	45.25 (44.65, 45.85)	54.24 (53.75, 54.73)	65.16 (64.65, 65.66)	77.67 (77.05, 78.28)	89.90 (89.10, 90.71)	97.65 (96.65, 98.64)	113.13 (111.50, 114.76)
31	80	29.18 (27.99, 30.36)	39.69 (38.87, 40.50)	45.15 (44.48, 45.83)	54.35 (53.83, 54.88)	65.51 (65.00, 66.03)	78.26 (77.64, 78.87)	90.69 (89.88, 91.50)	98.54 (97.55, 99.53)	114.18 (112.57, 115.79)
32	82	28.73 (27.53, 29.93)	39.43 (38.58, 40.28)	45.01 (44.30, 45.71)	54.40 (53.86, 54.95)	65.79 (65.28, 66.29)	78.75 (78.17, 79.34)	91.38 (90.63, 92.13)	99.33 (98.41, 100.25)	115.12 (113.58, 116.66)

For peer review only - http://bmjopen.bmj.com/site/about/guidelines.xhtml

	Age	1st	5th	10th	25th	50th	75th	90th	95th	99th
33	84	28.31 (27.16, 29.47)	39.18 (38.36, 40.01)	44.86 (44.17, 45.55)	54.44 (53.91, 54.98)	66.03 (65.55, 66.51)	79.21 (78.67, 79.75)	92.02 (91.31, 92.72)	100.06 (99.19, 100.93)	116.00 (114.50, 117.51)
34	86	27.92 (26.76, 29.07)	38.94 (38.14, 39.74)	44.72 (44.04, 45.39)	54.46 (53.93, 55.00)	66.25 (65.76, 66.74)	79.62 (79.06, 80.19)	92.60 (91.85, 93.35)	100.73 (99.80, 101.67)	116.81 (115.23, 118.39)
35	88	27.50 (26.22, 28.78)	38.67 (37.81, 39.53)	44.53 (43.81, 45.26)	54.44 (53.84, 55.05)	66.41 (65.84, 66.98)	79.97 (79.31, 80.62)	93.09 (92.22, 93.96)	101.31 (100.24, 102.38)	117.50 (115.72, 119.27)
36	90	27.00 (25.48, 28.51)	38.33 (37.33, 39.33)	44.28 (43.44, 45.12)	54.35 (53.63, 55.07)	66.49 (65.80, 67.18)	80.22 (79.47, 80.96)	93.48 (92.50, 94.46)	101.76 (100.54, 102.98)	118.05 (116.04, 120.07)

Appendix B - Diastolic and Pulse Pressure Centiles for Emergency/Elective cohorts

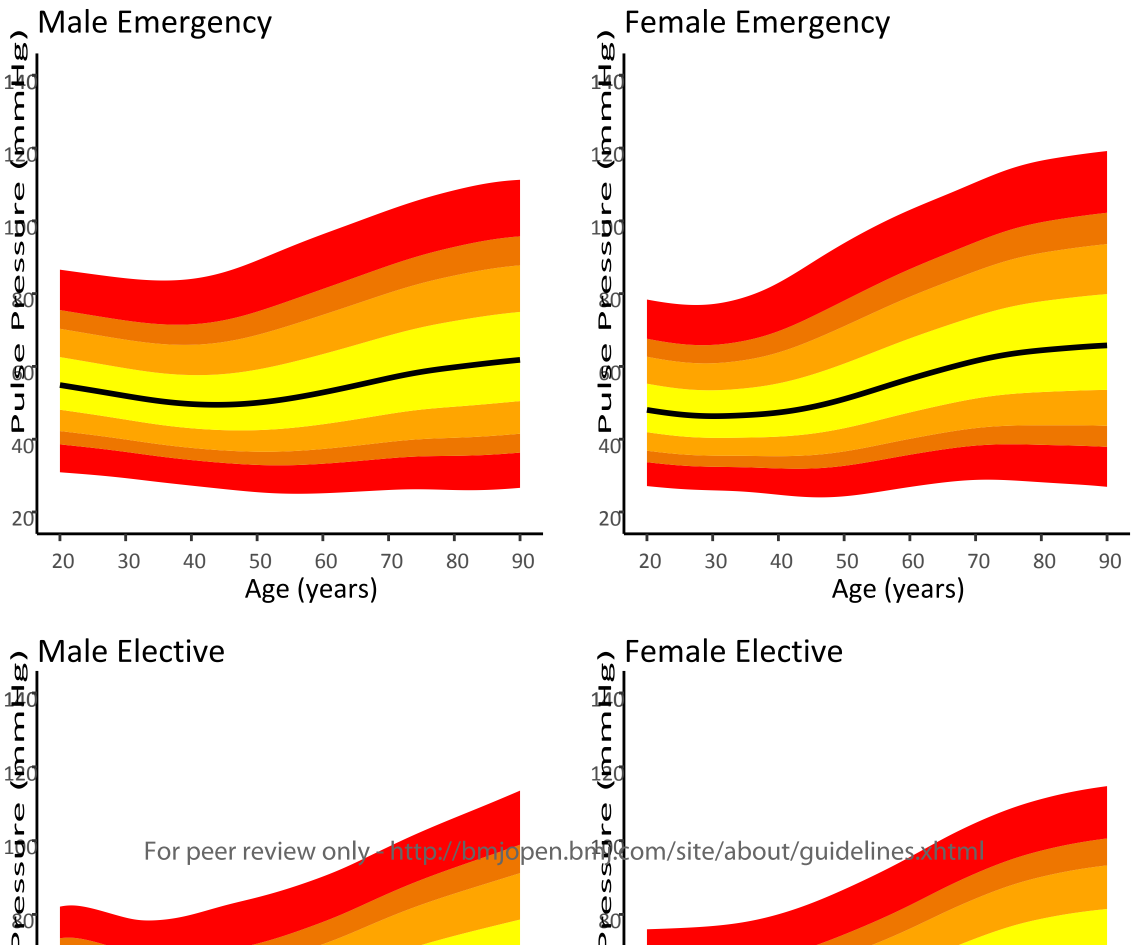

For peer review only - http://bmjopen.bmj.com/site/about/guidelines.xhtml

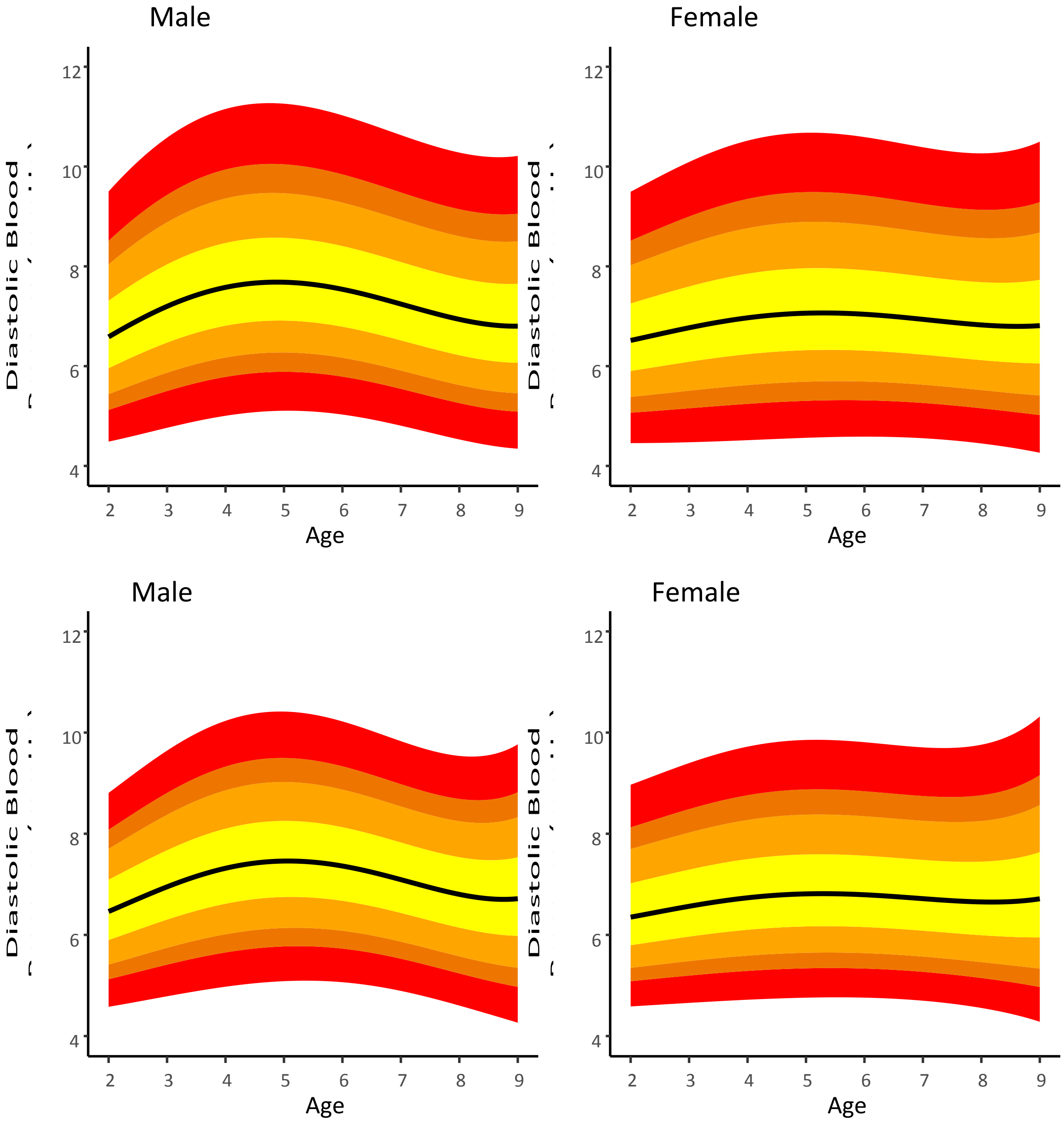

For peer review only - http://bmjopen.bmj.com/site/about/guidelines.xhtml

The RECORD statement – checklist of items, extended from the STROBE statement, that should be reported in observational studies using routinely collected health data.

	Item No.	STROBE items	Location in manuscript where items are reported	RECORD items	Location in manuscript where items are reported
**Title and abstract**					
	1	(a) Indicate the study's design with a commonly used term in the title or the abstract (b) Provide in the abstract an informative and balanced summary of what was done and what was found	yes	RECORD 1.1: The type of data used should be specified in the title or abstract. When possible, the name of the databases used should be included.<br><br>RECORD 1.2: If applicable, the geographic region and timeframe within which the study took place should be reported in the title or abstract.<br><br>RECORD 1.3: If linkage between databases was conducted for the study, this should be clearly stated in the title or abstract.	yes<br><br>Abstract<br><br>N/A
**Introduction**					
Background rationale	2	Explain the scientific background and rationale for the investigation being reported			Introduction
Objectives	3	State specific objectives, including any prespecified hypotheses			Introduction
**Methods**					
Study Design	4	Present key elements of study design early in the paper			Methods – paragraph 1
Setting	5	Describe the setting, locations, and relevant dates, including periods of recruitment, exposure, follow-up, and data collection			Methods – paragraph 1, 3

For peer review only

Participants	6	*(a) Cohort study* - Give the eligibility criteria, and the sources and methods of selection of participants. Describe methods of follow-up<br>*Case-control study* - Give the eligibility criteria, and the sources and methods of case ascertainment and control selection. Give the rationale for the choice of cases and controls<br>*Cross-sectional study* - Give the eligibility criteria, and the sources and methods of selection of participants<br><br>*(b) Cohort study* - For matched studies, give matching criteria and number of exposed and unexposed<br>*Case-control study* - For matched studies, give matching criteria and the number of controls per case	Methods – paragraph 2	RECORD 6.1: The methods of study population selection (such as codes or algorithms used to identify subjects) should be listed in detail. If this is not possible, an explanation should be provided.<br><br>RECORD 6.2: Any validation studies of the codes or algorithms used to select the population should be referenced. If validation was conducted for this study and not published elsewhere, detailed methods and results should be provided.<br><br>RECORD 6.3: If the study involved linkage of databases, consider use of a flow diagram or other graphical display to demonstrate the data linkage process, including the number of individuals with linked data at each stage.	Methods – paragraph 2, 4<br><br>Method – paragraph 4 (ref 12)<br><br>N/A
Variables	7	Clearly define all outcomes, exposures, predictors, potential confounders, and effect modifiers. Give diagnostic criteria, if applicable.		RECORD 7.1: A complete list of codes and algorithms used to classify exposures, outcomes, confounders, and effect modifiers should be provided. If these cannot be reported, an explanation should be provided.	Methods paragraph 3-4 (no codes required for blood pressure, as retrieved from curated system, which was itself taken from our own in-house system, SEND)
Data sources/ measurement	8	For each variable of interest, give sources of data and details of methods of assessment (measurement).			Methods Paragraph 3

		Describe comparability of assessment methods if there is more than one group			
Bias	9	Describe any efforts to address potential sources of bias			N/A – whole population is used (no sampling)
Study size	10	Explain how the study size was arrived at			Methods -> sample size
Quantitative variables	11	Explain how quantitative variables were handled in the analyses. If applicable, describe which groupings were chosen, and why			Methods -> Analysis
Statistical methods	12	(a) Describe all statistical methods, including those used to control for confounding<br>(b) Describe any methods used to examine subgroups and interactions<br>(c) Explain how missing data were addressed<br>(d) *Cohort study* - If applicable, explain how loss to follow-up was addressed<br>*Case-control study* - If applicable, explain how matching of cases and controls was addressed<br>*Cross-sectional study* - If applicable, describe analytical methods taking account of sampling strategy<br>(e) Describe any sensitivity analyses			Methods -> Analysis
Data access and cleaning methods		..		RECORD 12.1: Authors should describe the extent to which the investigators had access to the database	Methods: Paragraph 3

For peer review only

				population used to create the study population.<br><br>RECORD 12.2: Authors should provide information on the data cleaning methods used in the study.	 <br><br>Methods: Paragraph 3
Linkage		..		RECORD 12.3: State whether the study included person-level, institutional-level, or other data linkage across two or more databases. The methods of linkage and methods of linkage quality evaluation should be provided.	N/A
**Results**					
Participants	13	(a) Report the numbers of individuals at each stage of the study (*e.g.*, numbers potentially eligible, examined for eligibility, confirmed eligible, included in the study, completing follow-up, and analysed)<br>(b) Give reasons for non-participation at each stage.<br>(c) Consider use of a flow diagram		RECORD 13.1: Describe in detail the selection of the persons included in the study (*i.e.,* study population selection) including filtering based on data quality, data availability and linkage. The selection of included persons can be described in the text and/or by means of the study flow diagram.	Figure 1
Descriptive data	14	(a) Give characteristics of study participants (*e.g.*, demographic, clinical, social) and information on exposures and potential confounders<br>(b) Indicate the number of participants with missing data for each variable of interest<br>(c) *Cohort study* - summarise follow-up time (*e.g.*, average and total amount)			Table 1

For peer review only

Outcome data	15	*Cohort study* - Report numbers of outcome events or summary measures over time<br>*Case-control study* - Report numbers in each exposure category, or summary measures of exposure<br>*Cross-sectional study* - Report numbers of outcome events or summary measures			N/A (one measurement per admission)<br><br>Primary result: Fig 1
Main results	16	(a) Give unadjusted estimates and, if applicable, confounder-adjusted estimates and their precision (e.g., 95% confidence interval). Make clear which confounders were adjusted for and why they were included<br>(b) Report category boundaries when continuous variables were categorized<br>(c) If relevant, consider translating estimates of relative risk into absolute risk for a meaningful time period			(a) 95CIs reported throughout Results for pertinent values. Full table of CIs in supplementary material<br>(b) No categorisation for centile plots<br>(c) N/A
Other analyses	17	Report other analyses done—e.g., analyses of subgroups and interactions, and sensitivity analyses			Results Fig 3, 4
**Discussion**					
Key results	18	Summarise key results with reference to study objectives			Discussion
Limitations	19	Discuss limitations of the study, taking into account sources of		RECORD 19.1: Discuss the implications of using data that were not	Discussion -> Limitations

For peer review only

		potential bias or imprecision. Discuss both direction and magnitude of any potential bias		created or collected to answer the specific research question(s). Include discussion of misclassification bias, unmeasured confounding, missing data, and changing eligibility over time, as they pertain to the study being reported.	
Interpretation	20	Give a cautious overall interpretation of results considering objectives, limitations, multiplicity of analyses, results from similar studies, and other relevant evidence			Discussion -> Interpretation
Generalisability	21	Discuss the generalisability (external validity) of the study results			Discussion -> Generalisability
**Other Information**					
Funding	22	Give the source of funding and the role of the funders for the present study and, if applicable, for the original study on which the present article is based			Sources of Funding
Accessibility of protocol, raw data, and programming code		..		RECORD 22.1: Authors should provide information on how to access any supplemental information such as the study protocol, raw data, or programming code.	The raw data used for this research are not openly available.

*Reference: Benchimol EI, Smeeth L, Guttmann A, Harron K, Moher D, Petersen I, Sørensen HT, von Elm E, Langan SM, the RECORD Working Committee. The REporting of studies Conducted using Observational Routinely-collected health Data (RECORD) Statement. *PLoS Medicine* 2015; in press.

*Checklist is protected under Creative Commons Attribution (CC BY) license.

For peer review only - http://bmjopen.bmj.com/site/about/guidelines.xhtml

BMJ Open

Cross-Sectional Centiles of Blood Pressure by Age and Sex: a four-hospital database retrospective observational analysis.

Journal:	*BMJ Open*
Manuscript ID	bmjopen-2019-033618.R1
Article Type:	Original research
Date Submitted by the Author:	10-Feb-2020
Complete List of Authors:	Wong, David; The University of Manchester, Centre for Health Informatics; The University of Manchester, Computer Science<br>Gerry, Stephen; University of Oxford, Centre for Statistics in Medicine<br>Shamout, Farah; University of Oxford, Institute of Biomedical Engineering<br>Clifton, David; University of Oxford, Institute of Biomedical Engineering<br>Pimentel, Marco; University of Oxford, Institute of Biomedical Engineering<br>Watkinson, Peter; Oxford University Hospitals NHS Trust, Kadoorie Centre for Critical Care research and Education
**Primary Subject Heading**:	Emergency medicine
Secondary Subject Heading:	Health services research, Health informatics
Keywords:	blood pressure, hospitals, ageing, Physiology < BASIC SCIENCES

SCHOLARONE™
Manuscripts

I, the Submitting Author has the right to grant and does grant on behalf of all authors of the Work (as defined in the below author licence), an exclusive licence and/or a non-exclusive licence for contributions from authors who are: i) UK Crown employees; ii) where BMJ has agreed a CC-BY licence shall apply, and/or iii) in accordance with the terms applicable for US Federal Government officers or employees acting as part of their official duties; on a worldwide, perpetual, irrevocable, royalty-free basis to BMJ Publishing Group Ltd ("BMJ") its licensees and where the relevant Journal is co-owned by BMJ to the co-owners of the Journal, to publish the Work in this journal and any other BMJ products and to exploit all rights, as set out in our licence.

The Submitting Author accepts and understands that any supply made under these terms is made by BMJ to the Submitting Author unless you are acting as an employee on behalf of your employer or a postgraduate student of an affiliated institution which is paying any applicable article publishing charge ("APC") for Open Access articles. Where the Submitting Author wishes to make the Work available on an Open Access basis (and intends to pay the relevant APC), the terms of reuse of such Open Access shall be governed by a Creative Commons licence – details of these licences and which Creative Commons licence will apply to this Work are set out in our licence referred to above.

Other than as permitted in any relevant BMJ Author's Self Archiving Policies, I confirm this Work has not been accepted for publication elsewhere, is not being considered for publication elsewhere and does not duplicate material already published. I confirm all authors consent to publication of this Work and authorise the granting of this licence.

For peer review only

Cross-Sectional Centiles of Blood Pressure by Age and Sex: a four-hospital database retrospective observational analysis.

David C Wong – corresponding author
Centre for Health Informatics
Vaughan House
University of Manchester,
Manchester,
UK,
M13 9PR
email: david.wong@manchester.ac.uk

Stephen Gerry
Centre for Statistics in Medicine
Nuffield Department of Orthopaedics, Rheumatology and Musculoskeletal Sciences,
University of Oxford,
Oxford,
UK,
OX1 4AJ
email: stephen.gerry@csm.ox.ac.uk

Farah Shamout
Institute of Biomedical Engineering,
Old Road Campus Research Building,
Old Road,
Oxford,
UK,
OX3 7DQ
email: farah.shamout@eng.ox.ac.uk

David A Clifton
Institute of Biomedical Engineering,
Old Road Campus Research Building,
Old Road,
Oxford,

For peer review only

UK
OX3 7DQ
email: davidc@robots.ox.ac.uk

Marco Pimentel
Institute of Biomedical Engineering,
Old Road Campus Research Building,
Old Road,
Oxford,
UK
email: marco.pimentel@eng.ox.ac.uk

Peter Watkinson
Kadoorie Centre for Critical Care Research and Education,
Level 3, John Radcliffe Hospital,
Headley Way,
Oxford,
UK,
OX3 9DU
email: peter.watkinson@ndcn.ox.ac.uk

For peer review only

Abstract: 300 words

Manuscript: 2515 Words

Abstract

objectives: National guidelines for identifying physiological deterioration and sepsis in hospitals depend on thresholds for blood pressure that do not account for age or sex. In populations outside hospital, differences in blood pressure are known to occur with both variables. Whether these differences remain in the hospitalised population is unknown. This database analysis study aims to generate representative centiles to quantify variations in blood pressure by age and sex in hospitalised patients.

design: retrospective cross-sectional observational database analysis.

setting: four near-sea-level hospitals between April 2015 and April 2017

participants: 75342 adult patients who were admitted to the hospitals and had at least one set of documented vital sign observations within 24 hours before discharge were eligible for inclusion. Patients were excluded if they died in hospital, had no vital signs 24 hours prior to discharge, were readmitted within 7 days of discharge, had missing age or sex or had no blood pressure recorded.

results: Systolic blood pressure for hospitalised patients increases with age for both sexes. Median systolic blood pressure increases from 122 (CI: 121.1 – 122.1) mmHg to 132 (CI: 130.9 – 132.2) mmHg in men, and 114 (CI: 113.1-114.4) mmHg to 135 (CI: 134.5-136.2) mmHg for women, between the ages of 20 and 90. Diastolic blood pressure peaked around 50 years for men 76 (CI: 75.5-75.9)mmHg and women 69 (CI: 69.0-69.4) mmHg. The blood pressure criterion for sepsis, systolic < 100mmHg, was met by 2.3% of younger (20-30yrs) men and 3.5% of older men (81-90yrs). In comparison, the criterion was met by 9.7% of younger women and 2.6% of older women.

Conclusion: We have quantified variations in blood pressure by age and sex in hospitalised patients that have implications for recognition of deterioration. Nearly 10% of younger women met the blood pressure criterion for sepsis at hospital discharge

Key Words: Blood Pressure; Hospitals; Ageing; Physiology

For peer review only

Strengths and Limitations of this study

- Changes in Blood Pressure by age and sex are currently unknown for the hospitalised population
- A large cross-sectional database of 75342 patients were used to derive blood pressure centiles
- Results have implications on how sepsis and other in-hospital deterioration are detected in routine care
- Though patterns match those seen in out-of-hospital longitudinal studies, cross-sectional analysis can be affected by survival bias and birth cohort effects.

For peer review only

For peer review only - http://bmjopen.bmj.com/site/about/guidelines.xhtml

INTRODUCTION

Routine measurement of blood pressure is a key component of patient surveillance and diagnosis in hospitals worldwide. Currently, in-hospital assessment of blood pressure is undertaken by comparison to generic population normal ranges.

The ability to use an individual's physiology to monitor them for signs of deterioration may be improved by taking into account factors that affect these normal ranges.[1] For instance, in paediatric medicine, it is accepted that the normal ranges of vital signs vary with age and patients are managed in light of their age-specific normal ranges.[2-3] However, none of the published physiology-based systems for recognising deterioration in hospitalised adults take account of variations in vital signs by age or sex,[4] despite growing evidence that these factors may provide additional information for accurately identifying deterioration.[5-6] As examples, the National Early Warning Score (NEWS2)[7] scores systolic blood pressure below 90 mmHg as requiring emergency attention and current sepsis guidelines blood pressure criterion are met when systolic blood pressure is less than 100 mmHg,[8] both regardless of age or sex.

In populations outside hospital, differences in blood pressure are known to occur with both age and sex.[9] If clinically significant differences also exist in hospitalised adult populations, opportunities for earlier identification and management of patient deterioration may be being missed.

To quantify these differences, our objective was to define representative centiles of the stable hospitalized population for systolic, diastolic and pulse pressure by age and sex via an analysis of a large near-sea-level database of routinely-collected vital signs. Description of

For peer review only - http://bmjopen.bmj.com/site/about/guidelines.xhtml

For peer review only

this group allows inference about unstable patients via one class classification (novelty detection), which has previously been used when a clinical outcome of interest is relatively uncommon.

METHODS

This paper is reported according to RECORD guidelines. Approval for obtaining the data used in this study was obtained from the Oxford Research Ethics Committee (REC ref: 16/SC/0264). We conducted a cross-sectional analysis from a database collated at Oxford University Hospitals (OUH) NHS foundation trust group of hospitals. The OUH consists of four hospitals: an urban teaching hospital, a general district hospital, and two specialist hospitals. Our data set included patients admitted to the OUH between April 2015 and April 2017.

All adult patients who were admitted to OUH and had at least one set of documented vital sign observations within 24 hours prior to discharge were eligible for inclusion. Patients were excluded from the analysis if they (1) died in hospital, (2) had no recorded vital signs 24 hours prior to discharge, (3) were readmitted within 7 days of discharge, (4) had missing recordings for age or sex or (5) had no blood pressure recorded.

We collected the final recorded set of blood pressure observations from a patient's first attendance to the OUH hospital group during the study period. This ensured that the centiles were not biased towards repeat attenders or patients with longer hospital stays. Blood pressure was measured using automated devices ratified for clinical use as part of routine clinical care and electronically documented using the SEND e-Obs software.[10] Data were validated for plausible range at the point of entry. Hospital admission time, discharge

For peer review only - http://bmjopen.bmj.com/site/about/guidelines.xhtml

time, discharge status, ethnicity, Admission Method and Main Specialty were also collected for each patient from the hospital electronic patient record (Cerner Millennium, Cerner, Kansas City, MO). One investigator (PW) had access to a small proportion of the database population as part of routine clinical care responsibilities.

Admissions were typed as either Elective, Emergency or Other, according to Admission Method code. Codes are defined in full within the NHS data dictionary.[11] The set of ICD-10 codes {I10, I11, I12, I13, I14, I15} were used to determine patients with a primary diagnosis of hypertension.[12]

Analysis

The characteristics of the study population were described using medians and quartiles for the continuous variables, and frequencies otherwise. We calculated median and representative centiles (1st, 5th, 10th, 25th, 75th, 90th, 95th, 99th) for blood pressure at all ages between 20 and 90, for males and females. Systolic blood pressure (SBP) and diastolic blood pressure (DBP) are presented separately. One further measure, the pulse pressure (PP) was derived as the arithmetic difference between SBP and DBP and was also analysed using the same methods.

In subgroup analyses, we produced separate representative centiles by age and sex for Emergency and Elective Admissions, and for patients without a diagnostic code for hypertension.

Centiles were estimated using the Generalised Additive Models for Location, Scale, and Shape framework (GAMLSS).[13] This semi-parametric method provides various options for fitting non-normal distributions to the data. To create smooth centiles across the age range,

For peer review only - http://bmjopen.bmj.com/site/about/guidelines.xhtml

For peer review only

penalised splines and fractional polynomials were used as smoothing functions. For each vital sign, we assessed six different distributions within the GAMLSS framework: Box-Cox Cole and Green, Box-Cox Power Exponential, Box-Cox-t, Skew Power Exponential type 3, Skew t type 3, and Power Exponential. The best fitting distribution was chosen based on a combination of model fit (Akaike information criterion and Bayesian information criterion)[14-15] and a comparison of fitted versus empirical centiles. Box-Cox t distribution was the best fit for male and female SBP, the Skew t type 3 distribution was chosen for male DBP and male and female PP, and the Skew power exponential distribution was chosen for female DBP. SBP and PP models used penalised-splines as a smoother, whilst the DBP models used fractional polynomials as a smoother. To ensure fair comparison, the same distribution was chosen for all subgroups within any given vital sign.

All analyses were undertaken using R and the GAMLSS package.[16]

Sample size

We used all the available data and therefore no formal sample size calculation was undertaken. To ensure that the sample was sufficient, the precision of the centiles was estimated via a bootstrapping procedure, whereby the dataset was sampled with replacement to create a new dataset and the analysis was carried out.[17] This was repeated 50 times. The standard deviation of these bootstrapped estimates was used to calculate the 95% confidence interval for each centile at two-yearly intervals. Full details of centile values and confidence intervals are provided in Appendix A.

For peer review only - http://bmjopen.bmj.com/site/about/guidelines.xhtml

Patient and Public Involvement

Patients or the public were not involved in the design, or conduct, or reporting, or dissemination plans of our research

RESULTS

83004 patients were admitted to the hospital trust during the period of study and received at least one observation on the SEND e-Obs system. In total, 75342 patients were included in the study. Blood pressure was missing in 885 (1.17%) records. Other reasons for exclusion are shown in Figure 1. Patient and hospital descriptors are shown in Table 1.

Blood pressure centiles

Centiles by age for SBP, DBP and PP are shown in Figure 2 for each sex. Figure 2a shows progressive increase in median SBP from 122 (CI: 121.1 – 122.1) mmHg to 132 (CI: 130.9 – 132.2) mmHg for males between the ages of 20 and 90 years. Younger females had a lower median SBP than younger males (114 (CI: 113.1-114.4) mmHg at age 20). By the age of 90, median SBP was higher for females than for males (135 (CI: 134.5-136.2) mmHg).

Table 1 - Characteristics of the study population

	**Female**	**Male**	**Total**
**Total (N=75342)**	39157 (52.0%)	36185 (48.0%)	75342 (100.0 %)
**Patient Characteristics**			
**Ethnicity**			
White	30274	26580	56854 (75.5 %)
Mixed	263	261	524 (0.7 %)

For peer review only - http://bmjopen.bmj.com/site/about/guidelines.xhtml

Asian or Asian British	942	836	1778 (2.4 %)
Black or Black British	388	363	751 (1.0 %)
Other	361	341	702 (0.9 %)
Not known or stated	6929	7804	14733 (19.6 %)
**Age**			
<20	1082	918	2000 (2.7 %)
20-29	4137	3456	7593 (10.1 %)
30-39	4401	3391	7792 (10.3 %)
40-49	4995	4131	9126 (12.1 %)
50-59	5706	5676	11382 (15.1 %)
60-69	5815	6538	12353 (16.4 %)
70-79	6081	6674	12755 (16.9 %)
80-89	5084	4412	9496 (12.6 %)
>89	1856	989	2845 (3.8 %)
**Median Age (IQR)**	58 (40-75)	60 (43 – 74)	59 (41 - 74)
**Admission Characteristics**			
**Main Specialty**			
Medical	17023	13027	30050 (39.9 %)
Surgical	21202	22014	43216 (57.4 %)
Other	932	1144	2076 (2.8 %)
**Admission Method**			
Emergency	21542	19586	41383 (54.9 %)
Elective	17323	16596	33919 (45.0 %)

For peer review only - http://bmjopen.bmj.com/site/about/guidelines.xhtml

Other	37	3	40 (0.1 %)
**Hypertension Code**			
Yes	9622	10047	19669 (26.1 %)
No	29535	26138	55673 (73.9 %)

Figure 2b shows that median male DBP peaked at age 50 (76 (CI: 75.5-75.9) mmHg) with lower median DBP at age 20 (66 (CI: 65.0 – 66.0) mmHg) and age 90 (68 (CI: 67.9-68.4) mmHg). In the female cohort, the median DBP was 65 (CI: 64.6-65.0) mmHg, 69 (CI: 69.0-69.4) mmHg, and 68 (CI: 67.6-68.2) mmHg at ages 20, 50 and 90 respectively.

For males, there was a modest reduction in median PP between the ages of 20 and 40 from 55 mmHg (CI: 54.6-55.9) to 50 mmHg (CI: 49.2-50.0), whereas for females PP remained constant at 47mmHg (Figure 2c). Median PP increases for both sexes between the ages of 40 and 90, from 50mmHg (CI: 49.2-50.0) to 63mmHg for males, and 48mmHg (CI: 47.6-48.0) to 66mmHg (CI: 65.8.6-67.2) for females. Bootstrapped confidence intervals for SBP, DBP and PP are tabulated in supplementary material (Appendix A).

Figure 3 shows the differences in medians for SBP, DBP and PP between the ages of 20 and 90 for the whole study population in comparison to the subset without an ICD-10 diagnostic code for hypertension.

19669 patients had an ICD-10 diagnostic code for hypertension. Of these, 24.0 % (4711) an SBP of <120 mmHg and 2.3% (453) had a low SBP of <100 mmHg at the time of discharge. Per-decade percentages were not calculated due as small numbers of patients means that confidence intervals are wider than any differences between decades.

For peer review only - http://bmjopen.bmj.com/site/about/guidelines.xhtml

Figure 4 shows SBP centiles for the Emergency vs Elective sub-populations. DBP and PP centiles are included in Appendix B. In the 24 hours prior to discharge the 95th centile for systolic blood pressure for emergency male admissions at 50 years was 163 mmHg versus 155 mmHg for elective male admissions. Similarly, the 95th centile for systolic blood pressure for emergency female admissions at 50 years was 160 mmHg versus 152 mmHg for elective female admissions.

Proportion of patients with blood pressure below published alert thresholds

Table 2 shows the cumulative percentages of male and female patients who had SBP less than 90, 100, and 110 mmHg. These values correspond to the NEWS2 thresholds for hypotension.[7] 100mmHg is also a threshold used to assist in identifying sepsis.[8] For the 100mmHg threshold, 2.3% of younger (20-30yrs) men and 3.5% of older men (81-90yrs) fell below the threshold using their final reading in the 24 hours prior to discharge. In comparison, the criterion was met by 9.7% of younger women and 2.6% of older women.

Table 2 – percentages of male (N = 36185) and female (N=39157) patients with low systolic blood pressure within each decade

SBP	Gender (N,%)	Age (decade)								
		18-20	21-30	31-40	41-50	51-60	61-70	71-80	81-90	>90

For peer review only - http://bmjopen.bmj.com/site/about/guidelines.xhtml

For peer review only

**<90**	**Male** (120, 0.3%)	0.2	0.3	0.1	0.2	0.2	0.3	0.5	0.5	0.8
	**Female** (218, 0.6%)	0.8	1.0	0.9	0.9	0.4	0.4	0.3	0.2	0.2
**<100**	**Male** (1063, 2.9%)	2.9	2.3	2.4	2.2	2.6	3.2	3.5	3.5	4.6
	**Female** (2060, 5.3%)	11.1	9.7	9.4	6.5	4.4	3.1	2.6	2.6	2.0
**<110**	**Male** (4817, 13.3%)	16.2	13.2	13.6	12.7	12.9	13.1	12.7	14.5	15.7
	**Female** (8081, 20.6%)	37.7	35.7	34.7	25.8	18.7	13.2	11.1	10.4	10.8

DISCUSSION

We have generated blood pressure centiles for age and sex from a large multi-hospital patient database.

Discharge blood pressures (SBP, DBP, PP) showed clinically significant differences across age ranges and by sex. SBP progressively increased with age for both sexes, but progression was greater in females. DBP increased and then decreased across the life course for both sexes. The fluctuation in DBP was greater for males than for females. These overall trends were visible in both the whole population, and for the cohort that did not have a diagnostic code for hypertension.

In populations outside hospital these patterns are known to exist.[18-20] The Framingham studies showed that, for a healthy adult population, the mean arterial blood pressure increases throughout adulthood and that DBP decreases over the age of 60 as SBP continues to rise.[21] Similar trends in both SBP and DBP have been shown in large cross-sectional cohorts from multiple countries.[22-24]

For peer review only - http://bmjopen.bmj.com/site/about/guidelines.xhtml

While the overall patterns for blood pressure are known to exist for the general population outside hospital, we believe that this is the first time that centiles have been derived from an in-hospital setting.

Limitations

Vital signs were recorded as part of standard clinical practice, so the conditions for data recording were not controlled. This may have directly impacted the measured values. For instance, the state of wakefulness of the patient, which is known to be associated with blood pressure and pulse rate, was unknown.[25] However, it seems likely that such effects will be averaged out in a data set of this size.

We used the last recorded blood pressure in the 24 hours prior to discharge. Whilst this lessens many biases in comparison to other methods and may represent the blood pressure recording when the patient is most stable, there may be other patterns at different points during a hospital admission.

Finally, this study uses a cross-sectional cohort so the derived centiles may be affected by survival bias and birth cohort effects.[26-27] While the influence of these effects cannot be determined, we note that the overall trends follow those previously seen for longitudinal data in healthy populations.[28]

Interpretation

The differences in normal vital sign ranges due to age and sex could have substantial implications for hospital practice. For example, Table 2 showed that current Systolic Blood

For peer review only - http://bmjopen.bmj.com/site/about/guidelines.xhtml

Pressure criteria for identifying sepsis (SBP<100 mmHg) would be met by women much more frequently than by men up to age 50. Despite this, current evidence shows that males are more prone to develop sepsis than women.[29] A more accurate identification of patients at risk of sepsis may be possible through sex and age-stratified criteria.

19669 of patients diagnosed with hypertension had normal or low SBP immediately prior to discharge. This cohort may be reasonably assumed to be prescribed with anti-hypertensives for the purpose of managing blood pressure. Whilst we do not have information on blood pressure medication following discharge, this group may be considered an estimate of the upper-bound of those at risk of medication withdrawal. Inappropriate blood pressure medication withdrawal has been associated with higher risk of further complications. [30]

Another important application for age and sex stratification is Early Warning Scores (EWS). In these systems, integer scores are assigned to each vital sign according to deviation from normality. The aggregate score is then used to guide appropriate clinical care. One such EWS, the National Early Warning Score (NEWS), has been validated in a large in-hospital population and is widely used in the United Kingdom and the Republic of Ireland.[31]

Based on the results presented, an age-stratified score could strongly affect the quality of care a patient receives. For instance, from Table 2, we see that 34.7% of women aged 31-40 years have a NEWS score of 1 or greater due to low SBP (SBP ≤ 110). In comparison, only 11.1% of women aged 71-80 years would meet the same blood pressure criterion. In contrast, 13.6% of men aged 31-40 and 12.7% of men aged 71-80 would have a NEWS score of 1 or more. These differences suggest it may be possible to improve discrimination between stability and deterioration by taking account of age and sex.

For peer review only - http://bmjopen.bmj.com/site/about/guidelines.xhtml

Until now, age and sex have not been included within any adult EWS, despite evidence indicating its limitations in predicting deterioration of elderly patients.[6-7] An update to the NEWS score to include these additional variables may be difficult to achieve in practice. In particular, the NEWS score was validated using in-hospital mortality. Adequate validation of the stratified score would require reasonable numbers of in-hospital mortality for each combination of sex and age-range, where death is relatively rare in younger cohorts. One alternative approach may be to derive a model that directly uses the representative centiles for each vital sign to provide EWS scores.[32]

Generalisability

The data set was collected from all four hospitals, but we note that there are high proportions of white Caucasian patients. Previous studies have shown correlation between ethnicity and differences in blood pressure trajectory.[33] Whether inclusion of ethnicity could also improve discrimination requires further research.

Our work shows for the first time that meeting current blood pressure criteria for sepsis or early warning system alerts is substantially more likely in younger women than in all other groups in the 24 hours prior to discharge. Exploration of this finding is needed to determine whether adjustment for age and sex can improve discrimination and prevent overdiagnosis.

CONCLUSION

Substantial variations in the final blood pressure recorded in the 24 hours prior to hospital discharge occur with age and sex. These result in large differences in the proportions of patients meeting blood pressure criterion for sepsis and early warning score alerts. These

For peer review only - http://bmjopen.bmj.com/site/about/guidelines.xhtml

For peer review only

factors should be examined to understand whether these factors could be used to improve discrimination between stability and deterioration.

COMPETING INTERESTS

DW and PW co-developed the SEND e-Obs system (for which Sensyne Health has purchased sole license) from which the study database was collected. and. The company has a research agreement with the University of Oxford and royalty agreements with Oxford University Hospitals NHS Trust and the University of Oxford. DAC is Research Director of Sensyne Health. PW is employed part-time and holds shares in Sensyne Health. DW undertakes consultancy for Sensyne Health.

FUNDING

This publication presents independent research funded by the Health Innovation Challenge Fund (HICF-R9-524; WT-103703/Z/14/Z), a parallel funding partnership between the Department of Health and Wellcome Trust. The views expressed in this publication are those of the author(s) and not necessarily those of the Department of Health or Wellcome Trust. PW and DAC are funded by the Health Innovation Challenge Fund and Wellcome Trust (HICF-R9-524 and WT-103703/Z/14/Z). PW is supported by the NIHR Biomedical Research Centre, Oxford. SG is funded via an NIHR Doctoral Research Fellowship. FS is funded by the Rhodes Trust.

AUTHOR CONTRIBUTIONS

DW, DAC, PW conceived and designed the study; MP, DW, SG acquired the data; SG, DW, FS analysed the data. DW, SG, DAC, MP, FS, PW were in involved in drafting and revising the manuscript and have approved the final submitted version.

For peer review only - http://bmjopen.bmj.com/site/about/guidelines.xhtml

DATA SHARING STATEMENT

The raw data for this research are not openly available. Researchers who present a sound analysis plan for any valid research can apply to ccrg@ndcn.ox.ac.uk. Proposals considered valid by the Kadoorie Critical Care Research Group Data Access Committee (which comprises independent researchers, clinicians, patient and public representatives) will be provided with data using the group's current system that complies with data governance requirements. R code and results in csv format are available at https://github.com/davcom2/BP_centiles.

For peer review only

REFERENCES

1. Cuthbertson BH, Boroujerdi M, Mckie L, et al. Can physiological variables and early warning scoring systems allow early recognition of the deteriorating surgical patient? *Crit Care Med* 2007;35(2):402-9.

2. Duncan H, Hutchison J, Parshuram CS. The Pediatric Early Warning System score: a severity of illness score to predict urgent medical need in hospitalized children. *J Crit Care* 2006;21(3):271-8.

3. Fleming S, Thompson M, Stevens R, et al. Normal ranges of heart rate and respiratory rate in children from birth to 18 years of age: a systematic review of observational studies. *Lancet* 2011;377(9770):1011-8.

For peer review only - http://bmjopen.bmj.com/site/about/guidelines.xhtml

4. Smith GB, Prytherch DR, Meredith P, et al. The ability of the National Early Warning Score (NEWS) to discriminate patients at risk of early cardiac arrest, unanticipated intensive care unit admission, and death. *Resuscitation* 2013;84(4):465-70.

5. Smith GB, Prytherch DR, Schmidt PE, et al. Should age be included as a component of track and trigger systems used to identify sick adult patients? *Resuscitation* 2008;78(2):109-15.

6. Churpek MM, Yuen TC, Winslow C, et al. Differences in vital signs between elderly and non-elderly patients prior to ward cardiac arrest. *Crit Care Med* 2015;43(4):816.

7. Royal College of Physicians (London). National Early Warning Score (NEWS) 2 standardising the assessment of acute-illness severity in the NHS. 2017

8. Singer M, Deutschman CS, Seymour CW, et al. The third international consensus definitions for sepsis and septic shock (sepsis-3). *Jama* 2016;315(8):801-10.

9. Franklin SS, Gustin W, Wong ND, et al. Hemodynamic patterns of age-related changes in blood pressure - The Framingham Heart study. *Circulation* 1997;96(1):308-15.

10. Wong D, Bonnici T, Knight J, et al. SEND: a system for electronic notification and documentation of vital sign observations. *BMC Med Inform Decis Mak* 2015;15(1):1.

11. NHS Data Model and Dictionary. NHS Data Model and Dictionary Version 3 http://www.datadictionary.nhs.uk/ (date accessed: 13 November 2018)

12. Beckman KD. How to document and code for hypertensive diseases in ICD-10. *Family practice management* 2014 Apr;21(2):5-9.

For peer review only - http://bmjopen.bmj.com/site/about/guidelines.xhtml

13. Rigby, RA, Stasinopoulos, DM. Generalized additive models for Location, Scale and Shape (GAMLSS) in R. *J Stat Soft* 2007; 23(7):1–46

14. Akaike, H. A new look at the statistical model identification. *IEEE Trans Automat Contr* 1974;19(6):716–723

15. Schwarz, G. Estimating the dimension of a model. *Ann Stats* 1978;6:461–464.

16. R Development Core Team (2008). R: A language and environment for statistical computing. R Foundation for Statistical Computing, Vienna, Austria. ISBN 3-900051-07-0, URL http://www.R-project.org.

17. Bland JM, Altman DG. Statistics notes: bootstrap resampling methods. *BMJ* 2015;350:h2622.

18. Pinto E. Blood pressure and ageing. *Postgrad Med J* 2007;83(976):109-14.

19. Baker SE, Limberg JK, Ranadive SM, et al. Neurovascular control of blood pressure is influenced by aging, sex, and sex hormones. *Am J Physiol* 2016;311(6):R1271-5.

20. Joyner MJ, Wallin BG, Charkoudian N. Sex differences and blood pressure regulation in humans. *Exp Physiol* 2016;101(3):349-55.

21. Franklin SS, Khan SA, Wong ND, et al. Is pulse pressure useful in predicting risk for coronary heart disease? *Circulation* 1999;100(4):354-60.

22. Hosseini M, Baikpour M, Yousefifard M, et al. Blood pressure percentiles by age and body mass index for adults. *EXCLI J*. 2015;14:465-77

For peer review only

For peer review only - http://bmjopen.bmj.com/site/about/guidelines.xhtml

23. Balijepalli C, Lösch C, Bramlage P, et al. Percentile distribution of blood pressure readings in 35683 men and women aged 18 to 99 years. *J Hum Hypertens* 2014;28(3):193-200

24. Wright JD, Hughes JP, Ostchega Y,et al. Mean systolic and diastolic blood pressure in adults aged 18 and over in the United States, 2001–2008. *Natl Health Stat Report* 2011;35(1-22):24.

25. D. Mancia G, Ferrari A, Gregorini L, et al. Blood pressure and heart rate variabilities in normotensive and hypertensive human beings. *Circ Res* 1983;53(1):96-104.

26. Delgado-Rodriguez M, Llorca J. Bias. *J Epidemiol Community Health* 2004;58(8):635-41.

27. Keyes KM, Utz RL, Robinson W, Li G. What is a cohort effect? Comparison of three statistical methods for modeling cohort effects in obesity prevalence in the United States, 1971–2006. *Soc Sci Med* 2010;70(7):1100-8.

28. Wills AK, Lawlor DA, Matthews FE, et al. Life course trajectories of systolic blood pressure using longitudinal data from eight UK cohorts. PLoS Med 2011;8(6):e1000440.

29. Sakr Y, Elia C, Mascia L, et al. The influence of gender on the epidemiology of and outcome from severe sepsis. *Critical Care* 2013;17(2):R50.

30. Hirakawa Y, Arima H, Webster R, Zoungas S, Li Q, Harrap S, Lisheng L, Hamet P, Mancia G, Poulter N, Neal B. Risks associated with permanent discontinuation of blood pressure-lowering medications in patients with type 2 diabetes. *J Hypertens*. 2016, 34(4):781-7.

31. Prytherch DR, Smith GB, Schmidt PE, et al. ViEWS—towards a national early warning score for detecting adult inpatient deterioration. *Resuscitation* 2010;81(8):932-7.

For peer review only - http://bmjopen.bmj.com/site/about/guidelines.xhtml

32. Tarassenko L, Clifton DA, Pinsky MR, et al. Centile-based early warning scores derived from statistical distributions of vital signs. *Resuscitation* 2011;82(8):1013-8.

33. Agyemang C, Humphry RW, Bhopal R. Divergence with age in blood pressure in African-Caribbean and white populations in England: implications for screening for hypertension. *Am J Hypertens*. 2012; 25(1):89-96

Legends to Figures

Figure 1. Consort diagram showing analysis inclusion criteria

Table 1. Characteristics of the study population

Figure 2. 1st, 5th, 10th, 25th, 50th, 75th, 90th, 95th and 99th centiles of systolic, diastolic and pulse blood pressure for males and females between the ages of 20 and 90. Dashed lines in (a) denote SBP = {90, 100, 110} mmHg

Figure 3. Medians of systolic, diastolic and pulse blood pressure for all males and females between the ages of 20 and 90 (dashed lines) and the sub-group excluding patients with ICD codes for hypertension (solid lines).

Figure 4. 1st, 5th, 10th, 25th, 50th, 75th, 90th, 95th and 99th Systolic blood pressure centiles for Emergency and Elective sub-groups.

Table 2 – percentage of male and female patients with low systolic blood pressure

For peer review only - http://bmjopen.bmj.com/site/about/guidelines.xhtml

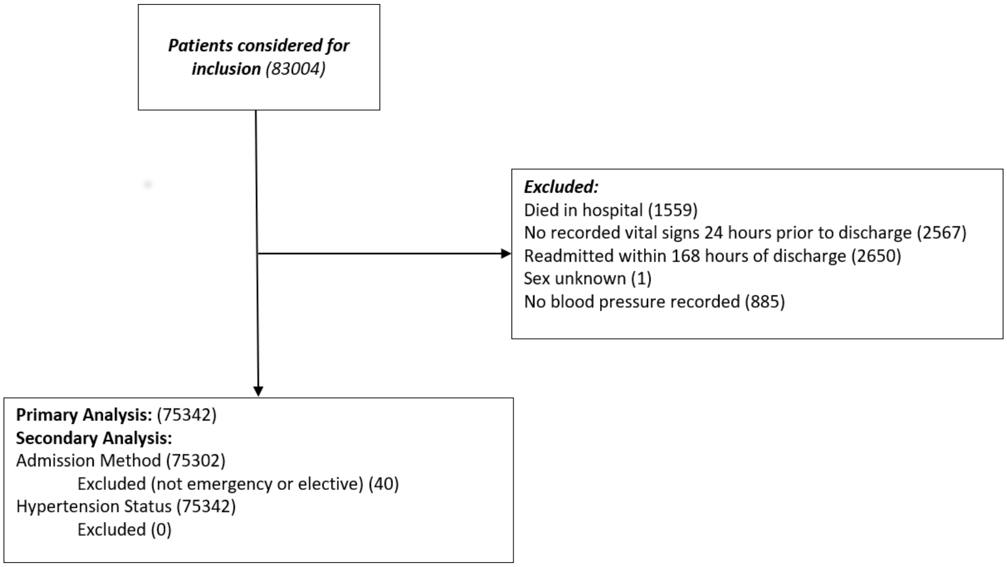

Figure 1. Consort diagram showing analysis inclusion criteria

102x56mm (300 x 300 DPI)

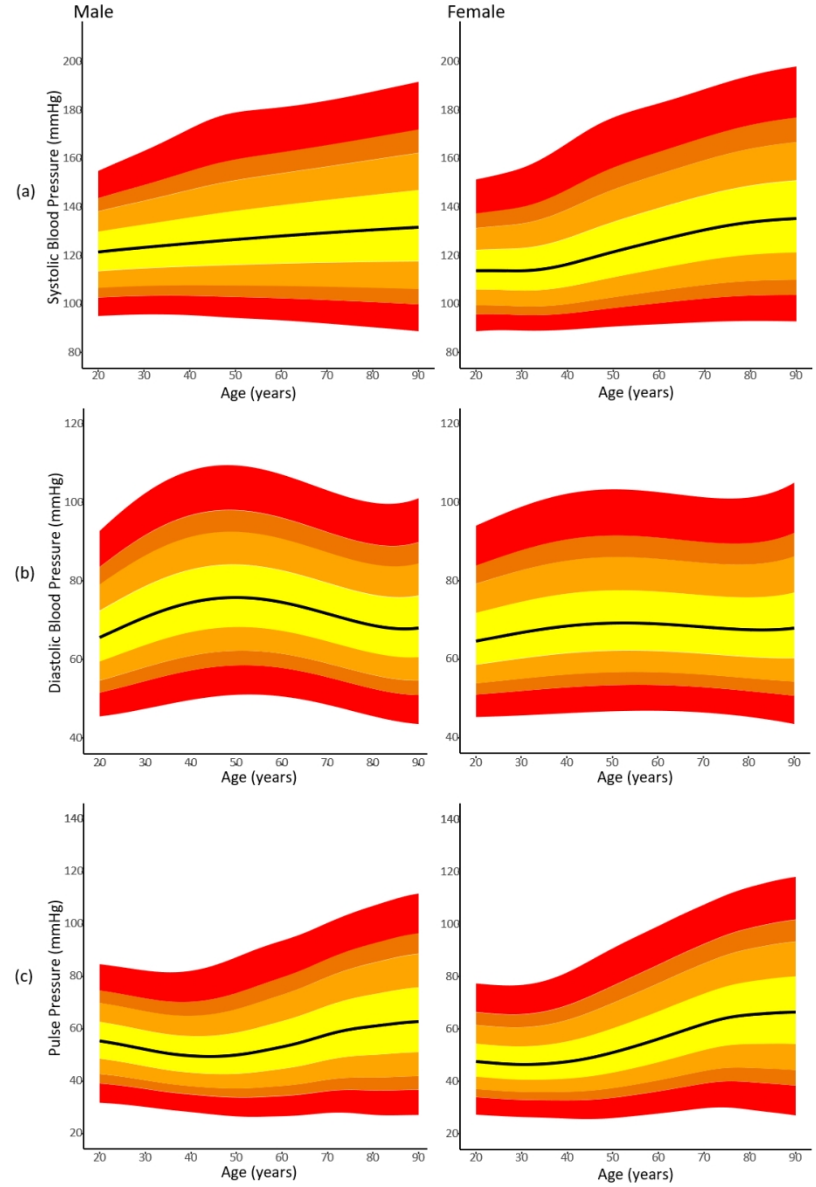

Figure 2. 1st, 5th, 10th, 25th, 50th, 75th, 90th, 95th and 99th centiles of (a) systolic, (b) diastolic and (c) pulse blood pressure for males and females between the ages of 20 and 90. Dashed lines in (a) denote SBP = {90, 100, 110} mmHg

94x139mm (300 x 300 DPI)

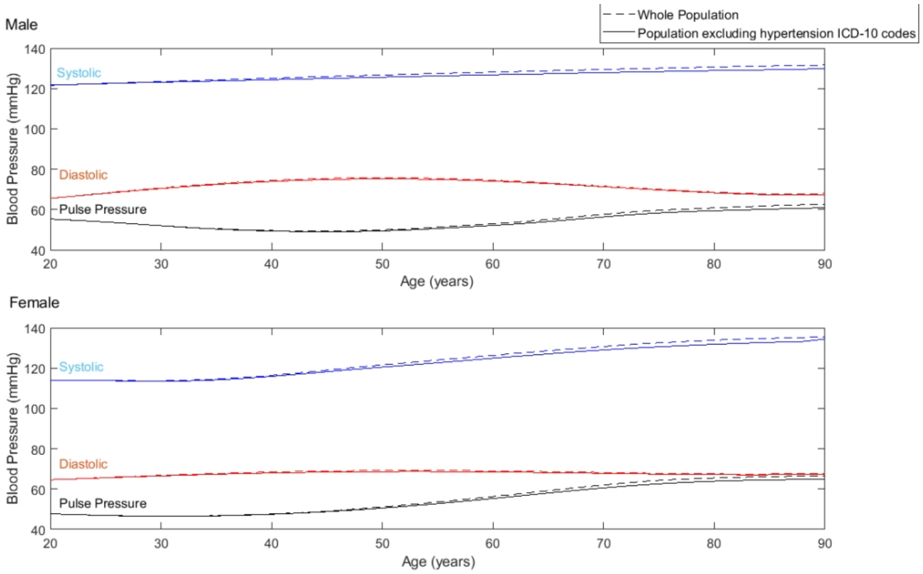

Figure 3. Medians of systolic, diastolic and pulse blood pressure for all males and females between the ages of 20 and 90 (dashed lines) and the sub-group excluding patients with ICD codes for hypertension (solid lines).

105x62mm (300 x 300 DPI)

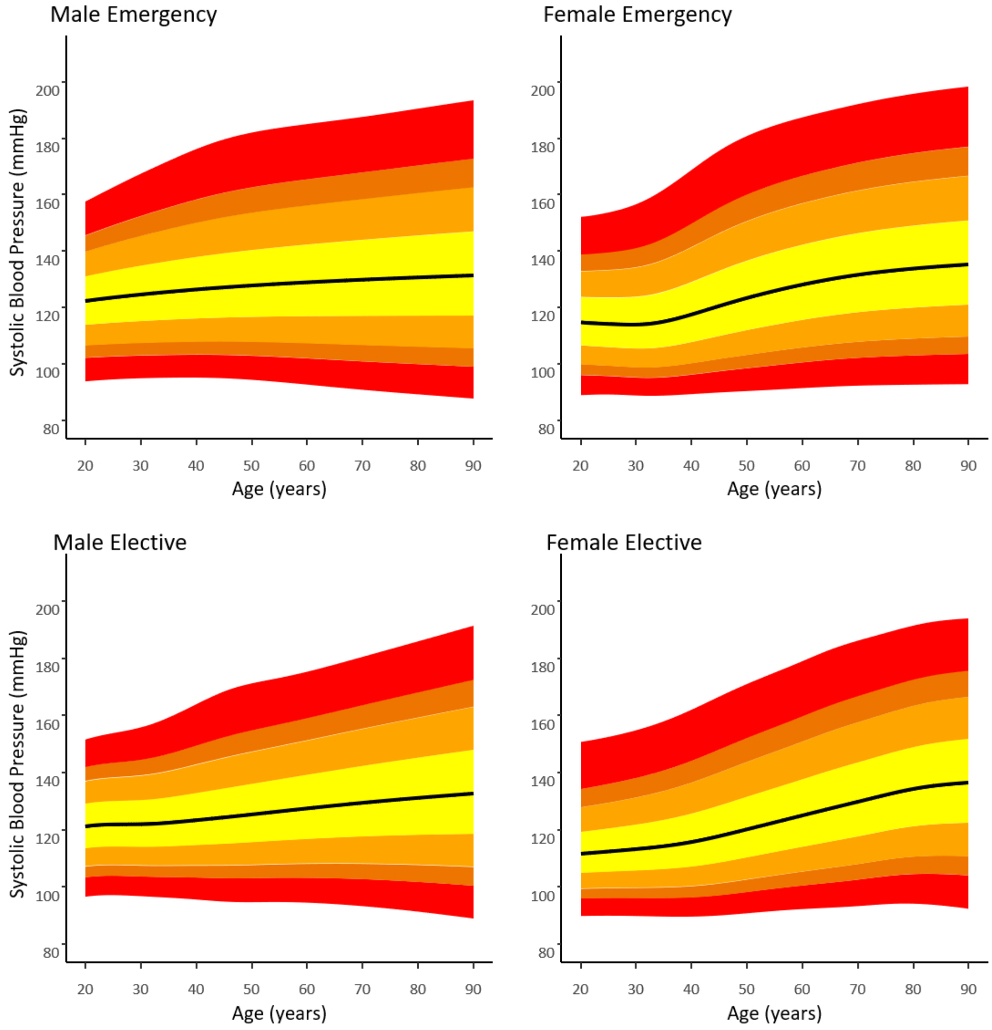

Figure 4. 1st, 5th, 10th, 25th, 50th, 75th, 90th, 95th and 99th Systolic blood pressure centiles for Emergency and Elective sub-groups.

92x97mm (300 x 300 DPI)

Supplementary Material

Appendix A – centiles and confidence intervals

Male systolic blood pressure centiles with 95% CI

	Age	_1st	_5th	_10th	_25th	_50th	_75th	_90th	_95th	_99th
1	20	95.12 (93.60, 96.65)	102.73 (101.90, 103.6)	106.8 (106.1, 107.5)	113.7 (113.1, 114.2)	121.6 (121.1, 122.1)	130.0 (129.4, 130.6)	138.3 (137.5, 139.1)	143.7 (142.7, 144.7)	154.9 (152.9, 157.0)
2	22	95.30 (94.02, 96.57)	102.91 (102.22, 103.6)	107.0 (106.4, 107.6)	113.9 (113.5, 114.4)	122.0 (121.6, 122.4)	130.6 (130.2, 131.1)	139.2 (138.6, 139.8)	144.8 (144.1, 145.5)	156.6 (154.9, 158.3)
3	24	95.46 (94.34, 96.58)	103.08 (102.48, 103.7)	107.2 (106.7, 107.7)	114.2 (113.8, 114.6)	122.4 (122.0, 122.7)	131.2 (130.9, 131.6)	140.1 (139.6, 140.6)	145.9 (145.2, 146.6)	158.3 (156.6, 160.0)
4	26	95.60 (94.56, 96.65)	103.22 (102.67, 103.8)	107.3 (106.9, 107.8)	114.4 (114.1, 114.8)	122.7 (122.5, 123.0)	131.8 (131.5, 132.2)	140.9 (140.4, 141.5)	147.0 (146.2, 147.8)	159.9 (158.0, 161.9)
5	28	95.71 (94.73, 96.69)	103.33 (102.78, 103.9)	107.5 (107.0, 107.9)	114.6 (114.2, 115.0)	123.1 (122.7, 123.5)	132.4 (131.9, 132.9)	141.8 (141.1, 142.5)	148.0 (147.1, 149.0)	161.6 (159.5, 163.6)
6	30	95.78 (94.85, 96.71)	103.42 (102.83, 104.0)	107.6 (107.1, 108.1)	114.8 (114.3, 115.3)	123.4 (122.9, 123.9)	132.9 (132.3, 133.6)	142.6 (141.8, 143.5)	149.1 (148.0, 150.2)	163.2 (161.1, 165.3)
7	32	95.80 (94.90, 96.69)	103.48 (102.88, 104.1)	107.7 (107.1, 108.2)	115.0 (114.5, 115.5)	123.8 (123.2, 124.3)	133.5 (132.8, 134.2)	143.5 (142.6, 144.4)	150.2 (149.0, 151.4)	164.9 (162.8, 167.1)
8	34	95.78 (94.91, 96.65)	103.51 (102.94, 104.1)	107.8 (107.2, 108.3)	115.2 (114.7, 115.7)	124.1 (123.6, 124.6)	134.1 (133.4, 134.8)	144.4 (143.5, 145.3)	151.4 (150.2, 152.5)	166.7 (164.5, 168.9)
9	36	95.72 (94.85, 96.58)	103.52 (102.98, 104.1)	107.8 (107.4, 108.3)	115.4 (114.9, 115.8)	124.5 (124.0, 124.9)	134.7 (134.1, 135.3)	145.3 (144.4, 146.2)	152.5 (151.3, 153.7)	168.6 (166.4, 170.7)
10	38	95.62 (94.74, 96.49)	103.50 (102.97, 104.0)	107.9 (107.4, 108.3)	115.5 (115.1, 115.9)	124.8 (124.4, 125.2)	135.3 (134.7, 135.8)	146.2 (145.3, 147.1)	153.7 (152.5, 154.9)	170.5 (168.2, 172.7)
11	40	95.47 (94.57, 96.36)	103.46 (102.92, 104.0)	107.9 (107.4, 108.3)	115.7 (115.3, 116.0)	125.1 (124.7, 125.5)	135.8 (135.3, 136.4)	147.1 (146.2, 148.0)	154.9 (153.7, 156.0)	172.4 (170.1, 174.6)
12	42	95.27 (94.33, 96.20)	103.38 (102.84, 103.9)	107.9 (107.4, 108.3)	115.8 (115.4, 116.2)	125.4 (125.1, 125.8)	136.4 (135.9, 137.0)	148.0 (147.2, 148.8)	156.0 (154.9, 157.1)	174.2 (172.0, 176.4)
13	44	95.03 (93.99, 96.07)	103.28 (102.72, 103.8)	107.9 (107.4, 108.3)	115.9 (115.6, 116.3)	125.8 (125.4, 126.1)	137.0 (136.5, 137.5)	148.9 (148.1, 149.6)	157.1 (156.0, 158.2)	175.9 (173.5, 178.2)

For peer review only - http://bmjopen.bmj.com/site/about/guidelines.xhtml

	Age	_1st	_5th	_10th	_25th	_50th	_75th	_90th	_95th	_99th
14	46	94.79 (93.62, 95.96)	103.18 (102.59, 103.8)	107.8 (107.4, 108.3)	116.0 (115.7, 116.4)	126.1 (125.7, 126.4)	137.5 (137.0, 138.0)	149.7 (148.9, 150.4)	158.1 (157.0, 159.1)	177.3 (174.7, 179.8)
15	48	94.56 (93.36, 95.76)	103.07 (102.48, 103.7)	107.8 (107.4, 108.2)	116.2 (115.8, 116.5)	126.4 (126.1, 126.7)	138.1 (137.6, 138.5)	150.4 (149.7, 151.1)	158.9 (157.9, 160.0)	178.4 (175.7, 181.0)
16	50	94.35 (93.26, 95.44)	102.97 (102.42, 103.5)	107.8 (107.4, 108.2)	116.3 (116.0, 116.6)	126.7 (126.4, 127.0)	138.6 (138.1, 139.0)	151.1 (150.4, 151.8)	159.7 (158.7, 160.7)	179.1 (176.7, 181.6)
17	52	94.16 (93.22, 95.10)	102.87 (102.37, 103.4)	107.8 (107.4, 108.1)	116.4 (116.1, 116.7)	127.0 (126.7, 127.3)	139.0 (138.6, 139.5)	151.7 (151.0, 152.4)	160.4 (159.4, 161.3)	179.7 (177.6, 181.9)
18	54	93.98 (93.14, 94.82)	102.78 (102.29, 103.3)	107.7 (107.3, 108.1)	116.5 (116.2, 116.8)	127.3 (127.0, 127.6)	139.5 (139.1, 139.9)	152.3 (151.6, 152.9)	161.0 (160.1, 161.9)	180.1 (178.2, 182.1)
19	56	93.79 (92.98, 94.60)	102.68 (102.19, 103.2)	107.7 (107.3, 108.1)	116.6 (116.3, 116.9)	127.6 (127.3, 127.8)	140.0 (139.6, 140.4)	152.8 (152.2, 153.5)	161.5 (160.6, 162.4)	180.5 (178.6, 182.4)
20	58	93.58 (92.79, 94.38)	102.57 (102.09, 103.0)	107.7 (107.3, 108.0)	116.7 (116.4, 117.0)	127.9 (127.6, 128.1)	140.4 (140.1, 140.8)	153.4 (152.8, 154.0)	162.1 (161.2, 163.0)	180.9 (179.0, 182.7)
21	60	93.36 (92.60, 94.13)	102.45 (101.98, 102.9)	107.6 (107.2, 108.0)	116.8 (116.5, 117.1)	128.1 (127.8, 128.4)	140.9 (140.5, 141.3)	153.9 (153.3, 154.5)	162.7 (161.8, 163.5)	181.3 (179.5, 183.1)
22	62	93.12 (92.40, 93.85)	102.31 (101.88, 102.7)	107.6 (107.2, 107.9)	116.9 (116.6, 117.2)	128.4 (128.1, 128.7)	141.3 (141.0, 141.7)	154.5 (153.9, 155.1)	163.2 (162.4, 164.1)	181.7 (180.0, 183.5)
23	64	92.86 (92.16, 93.56)	102.17 (101.76, 102.6)	107.5 (107.1, 107.8)	117.0 (116.7, 117.3)	128.7 (128.4, 129.0)	141.8 (141.4, 142.1)	155.0 (154.5, 155.6)	163.8 (163.0, 164.6)	182.2 (180.6, 183.9)
24	66	92.59 (91.89, 93.30)	102.03 (101.60, 102.4)	107.4 (107.1, 107.8)	117.1 (116.8, 117.4)	128.9 (128.6, 129.3)	142.2 (141.8, 142.6)	155.6 (155.0, 156.2)	164.4 (163.6, 165.2)	182.8 (181.2, 184.4)
25	68	92.31 (91.58, 93.04)	101.87 (101.43, 102.3)	107.3 (107.0, 107.7)	117.2 (116.8, 117.5)	129.2 (128.9, 129.5)	142.6 (142.3, 143.0)	156.2 (155.6, 156.7)	165.0 (164.2, 165.8)	183.4 (181.8, 185.0)
26	70	92.02 (91.25, 92.79)	101.71 (101.24, 102.2)	107.3 (106.9, 107.7)	117.2 (116.9, 117.6)	129.5 (129.1, 129.8)	143.1 (142.7, 143.5)	156.7 (156.1, 157.3)	165.6 (164.8, 166.4)	184.0 (182.5, 185.6)
27	72	91.72 (90.91, 92.54)	101.55 (101.04, 102.1)	107.2 (106.8, 107.6)	117.3 (116.9, 117.7)	129.7 (129.3, 130.1)	143.5 (143.1, 143.9)	157.3 (156.7, 157.9)	166.2 (165.4, 167.1)	184.7 (183.2, 186.3)
28	74	91.42 (90.58, 92.27)	101.39 (100.85, 101.9)	107.1 (106.7, 107.5)	117.4 (117.0, 117.8)	129.9 (129.6, 130.3)	143.9 (143.5, 144.3)	157.8 (157.2, 158.4)	166.9 (166.1, 167.7)	185.4 (183.9, 187.0)

For peer review only - http://bmjopen.bmj.com/site/about/guidelines.xhtml

	Age	_1st	_5th	_10th	_25th	_50th	_75th	_90th	_95th	_99th
29	76	91.12 (90.27, 91.97)	101.22 (100.68, 101.8)	107.0 (106.6, 107.5)	117.4 (117.1, 117.8)	130.2 (129.8, 130.6)	144.3 (143.9, 144.8)	158.4 (157.8, 159.0)	167.5 (166.7, 168.4)	186.2 (184.7, 187.7)
30	78	90.82 (89.99, 91.64)	101.05 (100.51, 101.6)	106.9 (106.5, 107.4)	117.5 (117.1, 117.9)	130.4 (130.0, 130.8)	144.7 (144.2, 145.2)	159.0 (158.3, 159.7)	168.2 (167.3, 169.1)	187.0 (185.4, 188.5)
31	80	90.51 (89.70, 91.31)	100.87 (100.33, 101.4)	106.8 (106.4, 107.3)	117.5 (117.2, 117.9)	130.6 (130.2, 131.1)	145.1 (144.6, 145.7)	159.5 (158.8, 160.3)	168.8 (167.8, 169.8)	187.8 (186.1, 189.5)
32	82	90.19 (89.39, 91.00)	100.70 (100.14, 101.3)	106.7 (106.3, 107.2)	117.6 (117.2, 118.0)	130.9 (130.4, 131.3)	145.5 (145.0, 146.1)	160.1 (159.3, 161.0)	169.5 (168.4, 170.6)	188.6 (186.7, 190.5)
33	84	89.87 (89.05, 90.68)	100.51 (99.94, 101.1)	106.6 (106.1, 107.1)	117.6 (117.2, 118.1)	131.1 (130.6, 131.6)	145.9 (145.3, 146.6)	160.7 (159.7, 161.6)	170.1 (168.9, 171.4)	189.4 (187.3, 191.5)
34	86	89.54 (88.70, 90.37)	100.32 (99.71, 100.9)	106.5 (106.0, 107.1)	117.7 (117.1, 118.2)	131.3 (130.7, 131.9)	146.3 (145.6, 147.0)	161.2 (160.2, 162.2)	170.8 (169.5, 172.1)	190.2 (187.9, 192.5)
35	88	89.20 (88.31, 90.08)	100.13 (99.44, 100.8)	106.4 (105.8, 107.1)	117.7 (117.1, 118.4)	131.5 (130.8, 132.2)	146.7 (146.0, 147.5)	161.8 (160.7, 162.9)	171.5 (170.0, 172.9)	191.0 (188.5, 193.6)
36	90	88.85 (87.84, 89.86)	99.93 (99.11, 100.7)	106.3 (105.5, 107.1)	117.8 (117.0, 118.5)	131.7 (130.9, 132.5)	147.1 (146.2, 148.0)	162.3 (161.1, 163.6)	172.1 (170.5, 173.7)	191.8 (189.0, 194.6)

Female systolic blood pressure centiles with 95% CI

	Age	_1st	_5th	_10th	_25th	_50th	_75th	_90th	_95th	_99th
1	20	88.97 (87.67, 90.27)	95.95 (95.19, 96.70)	99.69 (99.04, 100.34)	106.1 (105.5, 106.7)	113.8 (113.1, 114.4)	122.3 (121.6, 123.1)	131.3 (130.4, 132.1)	137.4 (136.5, 138.4)	151.4 (149.5, 153.4)
2	22	89.22 (88.16, 90.28)	95.99 (95.37, 96.62)	99.69 (99.17, 100.21)	106.1 (105.7, 106.6)	113.8 (113.3, 114.3)	122.5 (121.9, 123.1)	131.6 (130.9, 132.4)	137.9 (137.0, 138.8)	152.2 (150.6, 153.8)
3	24	89.37 (88.44, 90.30)	95.99 (95.40, 96.58)	99.64 (99.13, 100.15)	106.0 (105.6, 106.5)	113.8 (113.3, 114.3)	122.6 (122.0, 123.3)	132.0 (131.1, 132.8)	138.4 (137.4, 139.4)	153.0 (151.2, 154.9)
4	26	89.41 (88.60, 90.22)	95.91 (95.37, 96.45)	99.53 (99.03, 100.02)	105.9 (105.5, 106.4)	113.8 (113.3, 114.3)	122.8 (122.1, 123.4)	132.3 (131.4, 133.1)	138.9 (137.9, 139.9)	153.9 (152.0, 155.9)
5	28	89.35 (88.64, 90.06)	95.76 (95.31, 96.20)	99.36 (98.95, 99.76)	105.8 (105.4, 106.2)	113.7 (113.3, 114.2)	122.9 (122.3, 123.5)	132.6 (131.8, 133.4)	139.4 (138.4, 140.4)	154.9 (153.0, 156.9)
6	30	89.26 (88.58, 89.93)	95.61 (95.22, 96.01)	99.22 (98.84, 99.59)	105.7 (105.3, 106.1)	113.7 (113.3, 114.2)	123.1 (122.5, 123.7)	133.1 (132.3, 133.9)	140.1 (139.1, 141.1)	156.2 (154.1, 158.2)

	Age	_1st	_5th	_10th	_25th	_50th	_75th	_90th	_95th	_99th
7	32	89.20 (88.51, 89.88)	95.55 (95.13, 95.96)	99.17 (98.78, 99.55)	105.7 (105.3, 106.1)	113.9 (113.4, 114.4)	123.5 (122.9, 124.1)	133.8 (133.0, 134.6)	141.0 (140.0, 142.1)	157.7 (155.5, 159.8)
8	34	89.21 (88.52, 89.89)	95.59 (95.17, 96.01)	99.25 (98.87, 99.63)	105.9 (105.5, 106.3)	114.3 (113.8, 114.7)	124.1 (123.5, 124.7)	134.8 (134.0, 135.6)	142.3 (141.2, 143.3)	159.5 (157.3, 161.7)
9	36	89.28 (88.61, 89.95)	95.73 (95.33, 96.14)	99.45 (99.09, 99.81)	106.2 (105.9, 106.6)	114.8 (114.4, 115.2)	125.0 (124.5, 125.5)	136.0 (135.2, 136.7)	143.7 (142.7, 144.8)	161.7 (159.4, 163.9)
10	38	89.42 (88.77, 90.07)	95.97 (95.58, 96.36)	99.77 (99.42, 100.11)	106.7 (106.3, 107.1)	115.5 (115.1, 116.0)	126.0 (125.5, 126.5)	137.4 (136.7, 138.1)	145.4 (144.4, 146.5)	164.0 (161.7, 166.3)
11	40	89.61 (88.97, 90.25)	96.29 (95.91, 96.68)	100.18 (99.84, 100.53)	107.3 (106.9, 107.7)	116.4 (116.0, 116.8)	127.2 (126.7, 127.7)	139.0 (138.2, 139.7)	147.3 (146.2, 148.3)	166.4 (164.1, 168.8)
12	42	89.85 (89.20, 90.49)	96.69 (96.29, 97.09)	100.68 (100.32, 101.04)	108.0 (107.6, 108.4)	117.4 (116.9, 117.8)	128.5 (128.0, 129.1)	140.7 (139.9, 141.5)	149.2 (148.1, 150.3)	168.9 (166.6, 171.2)
13	44	90.12 (89.45, 90.79)	97.13 (96.70, 97.56)	101.23 (100.84, 101.62)	108.8 (108.4, 109.2)	118.4 (118.0, 118.9)	129.9 (129.3, 130.5)	142.4 (141.6, 143.2)	151.2 (150.1, 152.2)	171.3 (169.0, 173.5)
14	46	90.40 (89.70, 91.09)	97.60 (97.14, 98.06)	101.81 (101.40, 102.22)	109.6 (109.2, 110.0)	119.5 (119.1, 119.9)	131.3 (130.7, 131.9)	144.1 (143.3, 144.9)	153.0 (152.0, 154.1)	173.4 (171.3, 175.6)
15	48	90.66 (89.97, 91.36)	98.06 (97.57, 98.54)	102.38 (101.95, 102.81)	110.4 (109.9, 110.8)	120.6 (120.1, 121.0)	132.6 (132.1, 133.2)	145.7 (144.9, 146.4)	154.8 (153.8, 155.7)	175.3 (173.3, 177.4)
16	50	90.91 (90.23, 91.58)	98.50 (98.01, 98.98)	102.94 (102.50, 103.38)	111.1 (110.7, 111.5)	121.6 (121.1, 122.0)	133.9 (133.4, 134.4)	147.1 (146.4, 147.9)	156.3 (155.4, 157.3)	177.0 (175.0, 178.9)
17	52	91.13 (90.48, 91.77)	98.92 (98.45, 99.39)	103.48 (103.03, 103.93)	111.9 (111.4, 112.3)	122.5 (122.0, 123.1)	135.1 (134.5, 135.7)	148.5 (147.8, 149.2)	157.8 (156.8, 158.7)	178.4 (176.6, 180.2)
18	54	91.32 (90.69, 91.96)	99.32 (98.86, 99.79)	104.00 (103.55, 104.45)	112.6 (112.1, 113.1)	123.5 (123.0, 124.0)	136.3 (135.7, 136.9)	149.8 (149.1, 150.6)	159.1 (158.2, 160.0)	179.6 (177.8, 181.4)
19	56	91.51 (90.83, 92.19)	99.72 (99.24, 100.21)	104.52 (104.07, 104.98)	113.3 (112.9, 113.8)	124.5 (124.0, 125.0)	137.4 (136.9, 138.0)	151.1 (150.4, 151.8)	160.4 (159.5, 161.3)	180.7 (178.9, 182.5)
20	58	91.68 (90.92, 92.45)	100.12 (99.60, 100.64)	105.04 (104.58, 105.50)	114.0 (113.6, 114.5)	125.4 (124.9, 125.9)	138.5 (138.0, 139.0)	152.3 (151.7, 152.9)	161.6 (160.7, 162.5)	181.8 (179.9, 183.7)
21	60	91.86 (91.01, 92.71)	100.52 (99.95, 101.08)	105.56 (105.07, 106.04)	114.8 (114.3, 115.2)	126.3 (125.9, 126.7)	139.7 (139.2, 140.1)	153.5 (152.9, 154.1)	162.8 (162.0, 163.7)	182.8 (180.8, 184.8)

For peer review only - http://bmjopen.bmj.com/site/about/guidelines.xhtml

	Age	_1st	_5th	_10th	_25th	_50th	_75th	_90th	_95th	_99th
22	62	92.04 (91.15, 92.92)	100.91 (100.32, 101.50)	106.07 (105.56, 106.59)	115.5 (115.0, 115.9)	127.3 (126.8, 127.7)	140.7 (140.4, 141.1)	154.7 (154.1, 155.2)	164.0 (163.2, 164.9)	183.9 (181.9, 186.0)
23	64	92.22 (91.31, 93.12)	101.30 (100.69, 101.91)	106.58 (106.03, 107.12)	116.2 (115.7, 116.7)	128.2 (127.7, 128.6)	141.8 (141.4, 142.3)	155.9 (155.3, 156.5)	165.2 (164.4, 166.1)	185.1 (183.1, 187.1)
24	66	92.39 (91.43, 93.36)	101.68 (101.06, 102.30)	107.07 (106.50, 107.64)	116.8 (116.3, 117.4)	129.0 (128.5, 129.5)	142.9 (142.4, 143.4)	157.1 (156.4, 157.7)	166.4 (165.6, 167.3)	186.3 (184.3, 188.2)
25	68	92.57 (91.56, 93.58)	102.05 (101.41, 102.69)	107.54 (106.95, 108.13)	117.5 (116.9, 118.1)	129.9 (129.3, 130.4)	143.9 (143.3, 144.5)	158.2 (157.5, 158.9)	167.6 (166.7, 168.6)	187.5 (185.5, 189.4)
26	70	92.73 (91.78, 93.68)	102.39 (101.77, 103.02)	107.99 (107.40, 108.58)	118.1 (117.5, 118.7)	130.7 (130.1, 131.3)	144.9 (144.3, 145.5)	159.3 (158.6, 160.1)	168.8 (167.9, 169.7)	188.7 (186.9, 190.5)
27	72	92.88 (92.07, 93.68)	102.71 (102.13, 103.29)	108.40 (107.83, 108.97)	118.7 (118.1, 119.3)	131.5 (130.9, 132.0)	145.8 (145.2, 146.5)	160.4 (159.6, 161.2)	170.0 (169.0, 170.9)	189.9 (188.3, 191.5)
28	74	93.00 (92.30, 93.69)	102.99 (102.44, 103.54)	108.77 (108.23, 109.32)	119.2 (118.7, 119.8)	132.2 (131.6, 132.7)	146.7 (146.1, 147.4)	161.4 (160.6, 162.3)	171.1 (170.0, 172.1)	191.1 (189.4, 192.8)
29	76	93.09 (92.40, 93.77)	103.23 (102.69, 103.77)	109.10 (108.57, 109.63)	119.7 (119.2, 120.2)	132.8 (132.2, 133.4)	147.5 (146.9, 148.2)	162.4 (161.5, 163.2)	172.1 (171.0, 173.2)	192.2 (190.4, 194.1)
30	78	93.15 (92.39, 93.91)	103.43 (102.86, 103.99)	109.37 (108.84, 109.90)	120.1 (119.6, 120.6)	133.4 (132.8, 133.9)	148.3 (147.6, 148.9)	163.2 (162.4, 164.1)	173.0 (171.9, 174.2)	193.3 (191.3, 195.3)
31	80	93.18 (92.34, 94.01)	103.58 (102.98, 104.17)	109.59 (109.06, 110.12)	120.5 (120.0, 120.9)	133.9 (133.4, 134.4)	148.9 (148.3, 149.5)	164.0 (163.2, 164.9)	173.9 (172.7, 175.0)	194.3 (192.2, 196.3)
32	82	93.18 (92.35, 94.02)	103.69 (103.09, 104.28)	109.76 (109.24, 110.28)	120.7 (120.3, 121.2)	134.3 (133.8, 134.8)	149.5 (148.9, 150.0)	164.7 (163.9, 165.5)	174.7 (173.5, 175.8)	195.2 (193.2, 197.2)
33	84	93.17 (92.34, 93.99)	103.76 (103.16, 104.36)	109.89 (109.36, 110.42)	121.0 (120.5, 121.5)	134.6 (134.1, 135.1)	149.9 (149.3, 150.5)	165.3 (164.5, 166.2)	175.3 (174.2, 176.5)	196.0 (194.0, 198.0)
34	86	93.13 (92.30, 93.96)	103.81 (103.18, 104.43)	109.98 (109.42, 110.55)	121.1 (120.6, 121.7)	134.9 (134.3, 135.5)	150.4 (149.6, 151.1)	165.8 (164.9, 166.8)	175.9 (174.7, 177.2)	196.7 (194.7, 198.8)
35	88	93.08 (92.23, 93.92)	103.82 (103.19, 104.46)	110.05 (109.44, 110.65)	121.3 (120.7, 121.9)	135.2 (134.4, 135.9)	150.7 (149.8, 151.6)	166.3 (165.2, 167.4)	176.5 (175.1, 177.9)	197.4 (195.1, 199.7)
36	90	93.01 (92.14, 93.88)	103.82 (103.16, 104.48)	110.08 (109.44, 110.72)	121.4 (120.7, 122.1)	135.4 (134.5, 136.2)	151.0 (150.0, 152.0)	166.7 (165.4, 168.0)	176.9 (175.3, 178.6)	198.0 (195.3, 200.7)

Male diastolic blood pressure centiles with 95% CI

	Age	_1st	_5th	_10th	_25th	_50th	_75th	_90th	_95th	_99th
1	20	45.53 (44.75, 46.31)	51.56 (50.97, 52.14)	54.58 (54.06, 55.10)	59.55 (59.08, 60.02)	65.51 (65.02, 65.99)	72.36 (71.77, 72.95)	79.18 (78.42, 79.94)	83.60 (82.70, 84.49)	92.75 (91.48, 94.02)
2	22	45.83 (45.20, 46.46)	52.09 (51.66, 52.53)	55.23 (54.85, 55.60)	60.39 (60.06, 60.72)	66.58 (66.23, 66.92)	73.69 (73.25, 74.13)	80.78 (80.20, 81.35)	85.36 (84.67, 86.05)	94.87 (93.85, 95.89)
3	24	46.18 (45.62, 46.75)	52.66 (52.29, 53.03)	55.90 (55.59, 56.22)	61.25 (60.98, 61.51)	67.65 (67.37, 67.92)	75.00 (74.65, 75.36)	82.33 (81.85, 82.81)	87.08 (86.50, 87.66)	96.91 (96.03, 97.79)
4	26	46.58 (46.02, 47.14)	53.26 (52.88, 53.63)	56.60 (56.28, 56.91)	62.10 (61.83, 62.37)	68.70 (68.42, 68.97)	76.28 (75.93, 76.62)	83.83 (83.37, 84.28)	88.72 (88.17, 89.26)	98.85 (98.02, 99.67)
5	28	47.01 (46.43, 47.59)	53.86 (53.46, 54.26)	57.29 (56.95, 57.64)	62.94 (62.64, 63.24)	69.71 (69.41, 70.02)	77.49 (77.13, 77.86)	85.24 (84.78, 85.71)	90.26 (89.71, 90.81)	100.66 (99.85, 101.47)
6	30	47.47 (46.87, 48.07)	54.47 (54.05, 54.89)	57.98 (57.61, 58.35)	63.76 (63.43, 64.09)	70.68 (70.35, 71.01)	78.64 (78.25, 79.02)	86.56 (86.08, 87.04)	91.69 (91.14, 92.25)	102.33 (101.52, 103.14)
7	32	47.93 (47.32, 48.54)	55.07 (54.63, 55.50)	58.64 (58.26, 59.03)	64.53 (64.19, 64.88)	71.59 (71.24, 71.93)	79.69 (79.30, 80.09)	87.77 (87.29, 88.26)	93.00 (92.44, 93.56)	103.84 (103.03, 104.65)
8	34	48.40 (47.80, 49.00)	55.65 (55.22, 56.08)	59.28 (58.89, 59.66)	65.26 (64.91, 65.60)	72.42 (72.08, 72.77)	80.66 (80.27, 81.05)	88.86 (88.38, 89.34)	94.17 (93.62, 94.72)	105.18 (104.38, 105.97)
9	36	48.85 (48.27, 49.43)	56.19 (55.78, 56.61)	59.87 (59.50, 60.24)	65.92 (65.59, 66.26)	73.17 (72.84, 73.51)	81.51 (81.14, 81.89)	89.82 (89.36, 90.27)	95.19 (94.66, 95.72)	106.34 (105.56, 107.11)
10	38	49.29 (48.73, 49.84)	56.70 (56.31, 57.09)	60.41 (60.06, 60.76)	66.52 (66.20, 66.83)	73.84 (73.53, 74.15)	82.25 (81.91, 82.60)	90.64 (90.21, 91.06)	96.06 (95.57, 96.56)	107.31 (106.56, 108.06)
11	40	49.70 (49.17, 50.22)	57.15 (56.79, 57.52)	60.89 (60.57, 61.21)	67.04 (66.75, 67.33)	74.41 (74.13, 74.69)	82.88 (82.57, 83.19)	91.32 (90.93, 91.71)	96.78 (96.32, 97.24)	108.10 (107.38, 108.82)
12	42	50.06 (49.56, 50.56)	57.55 (57.21, 57.89)	61.30 (61.00, 61.60)	67.48 (67.21, 67.74)	74.88 (74.62, 75.13)	83.38 (83.10, 83.66)	91.85 (91.50, 92.21)	97.34 (96.91, 97.77)	108.71 (108.01, 109.40)
13	44	50.38 (49.90, 50.86)	57.88 (57.56, 58.21)	61.64 (61.36, 61.92)	67.83 (67.58, 68.07)	75.24 (75.01, 75.47)	83.76 (83.51, 84.01)	92.25 (91.92, 92.57)	97.74 (97.34, 98.14)	109.13 (108.45, 109.80)
14	46	50.65 (50.18, 51.12)	58.15 (57.84, 58.46)	61.90 (61.63, 62.18)	68.09 (67.85, 68.33)	75.50 (75.28, 75.71)	84.02 (83.79, 84.24)	92.50 (92.20, 92.80)	97.99 (97.61, 98.37)	109.38 (108.71, 110.04)
15	48	50.85 (50.39, 51.31)	58.34 (58.03, 58.65)	62.08 (61.81, 62.36)	68.26 (68.01, 68.50)	75.65 (75.44, 75.86)	84.15 (83.93, 84.37)	92.62 (92.33, 92.91)	98.10 (97.73, 98.47)	109.46 (108.79, 110.12)

For peer review only - http://bmjopen.bmj.com/site/about/guidelines.xhtml

	Age	_1st	_5th	_10th	_25th	_50th	_75th	_90th	_95th	_99th
16	50	50.99 (50.53, 51.45)	58.45 (58.13, 58.77)	62.18 (61.90, 62.47)	68.33 (68.08, 68.58)	75.70 (75.48, 75.92)	84.16 (83.94, 84.39)	92.60 (92.31, 92.89)	98.06 (97.69, 98.43)	109.38 (108.71, 110.05)
17	52	51.06 (50.60, 51.52)	58.48 (58.15, 58.81)	62.19 (61.89, 62.49)	68.31 (68.04, 68.57)	75.64 (75.41, 75.87)	84.07 (83.83, 84.30)	92.46 (92.16, 92.76)	97.89 (97.52, 98.27)	109.15 (108.48, 109.83)
18	54	51.05 (50.59, 51.52)	58.43 (58.09, 58.76)	62.12 (61.81, 62.43)	68.20 (67.92, 68.48)	75.48 (75.24, 75.73)	83.86 (83.62, 84.10)	92.20 (91.90, 92.51)	97.60 (97.22, 97.99)	108.80 (108.12, 109.48)
19	56	50.97 (50.50, 51.44)	58.29 (57.95, 58.64)	61.96 (61.64, 62.28)	68.00 (67.71, 68.29)	75.23 (74.98, 75.49)	83.55 (83.30, 83.81)	91.84 (91.53, 92.15)	97.20 (96.81, 97.59)	108.32 (107.63, 109.00)
20	58	50.81 (50.35, 51.28)	58.08 (57.73, 58.43)	61.72 (61.40, 62.04)	67.72 (67.42, 68.01)	74.90 (74.63, 75.16)	83.15 (82.89, 83.41)	91.38 (91.06, 91.70)	96.70 (96.31, 97.10)	107.74 (107.05, 108.42)
21	60	50.58 (50.13, 51.04)	57.79 (57.45, 58.14)	61.41 (61.09, 61.72)	67.35 (67.06, 67.64)	74.48 (74.22, 74.74)	82.67 (82.41, 82.93)	90.83 (90.52, 91.15)	96.12 (95.72, 96.51)	107.06 (106.37, 107.76)
22	62	50.28 (49.84, 50.73)	57.44 (57.11, 57.77)	61.02 (60.72, 61.33)	66.92 (66.64, 67.20)	73.99 (73.74, 74.25)	82.12 (81.87, 82.38)	90.22 (89.90, 90.54)	95.46 (95.07, 95.85)	106.32 (105.64, 107.01)
23	64	49.91 (49.48, 50.35)	57.02 (56.70, 57.33)	60.57 (60.28, 60.86)	66.43 (66.17, 66.69)	73.45 (73.21, 73.68)	81.51 (81.27, 81.75)	89.55 (89.24, 89.86)	94.75 (94.36, 95.14)	105.53 (104.85, 106.22)
24	66	49.49 (49.06, 49.91)	56.54 (56.24, 56.84)	60.07 (59.80, 60.34)	65.88 (65.64, 66.13)	72.85 (72.63, 73.07)	80.86 (80.63, 81.09)	88.84 (88.54, 89.14)	94.00 (93.62, 94.39)	104.71 (104.02, 105.39)
25	68	49.01 (48.59, 49.42)	56.01 (55.73, 56.29)	59.52 (59.27, 59.77)	65.30 (65.07, 65.52)	72.22 (72.02, 72.42)	80.18 (79.96, 80.39)	88.10 (87.81, 88.40)	93.24 (92.85, 93.62)	103.87 (103.19, 104.55)
26	70	48.48 (48.07, 48.89)	55.45 (55.18, 55.72)	58.94 (58.70, 59.17)	64.68 (64.48, 64.89)	71.57 (71.38, 71.75)	79.48 (79.27, 79.69)	87.37 (87.07, 87.66)	92.47 (92.09, 92.85)	103.04 (102.37, 103.72)
27	72	47.92 (47.51, 48.34)	54.86 (54.59, 55.13)	58.33 (58.10, 58.56)	64.05 (63.85, 64.25)	70.91 (70.73, 71.09)	78.79 (78.58, 79.00)	86.64 (86.34, 86.94)	91.72 (91.34, 92.10)	102.25 (101.57, 102.93)
28	74	47.34 (46.92, 47.76)	54.26 (53.99, 54.53)	57.72 (57.49, 57.96)	63.43 (63.22, 63.63)	70.26 (70.07, 70.46)	78.12 (77.90, 78.34)	85.95 (85.64, 86.26)	91.01 (90.62, 91.41)	101.51 (100.84, 102.19)
29	76	46.75 (46.31, 47.19)	53.66 (53.37, 53.95)	57.12 (56.87, 57.37)	62.82 (62.59, 63.04)	69.64 (69.43, 69.86)	77.49 (77.25, 77.74)	85.31 (84.99, 85.64)	90.37 (89.97, 90.78)	100.86 (100.18, 101.54)
30	78	46.16 (45.71, 46.62)	53.08 (52.77, 53.38)	56.54 (56.27, 56.81)	62.24 (61.99, 62.49)	69.08 (68.83, 69.32)	76.93 (76.66, 77.20)	84.76 (84.41, 85.10)	89.82 (89.40, 90.24)	100.32 (99.63, 101.00)

	Age	_1st	_5th	_10th	_25th	_50th	_75th	_90th	_95th	_99th
31	80	45.59 (45.12, 46.07)	52.53 (52.20, 52.85)	56.00 (55.71, 56.29)	61.72 (61.45, 61.99)	68.57 (68.30, 68.85)	76.45 (76.15, 76.76)	84.30 (83.93, 84.67)	89.38 (88.94, 89.82)	99.91 (99.22, 100.60)
32	82	45.06 (44.56, 45.55)	52.03 (51.69, 52.37)	55.52 (55.21, 55.83)	61.27 (60.98, 61.57)	68.16 (67.87, 68.46)	76.09 (75.75, 76.42)	83.98 (83.57, 84.38)	89.08 (88.61, 89.55)	99.67 (98.95, 100.39)
33	84	44.57 (44.05, 45.09)	51.60 (51.24, 51.97)	55.12 (54.80, 55.45)	60.92 (60.61, 61.23)	67.87 (67.55, 68.19)	75.85 (75.49, 76.22)	83.81 (83.36, 84.26)	88.96 (88.44, 89.48)	99.63 (98.86, 100.40)
34	86	44.16 (43.59, 44.72)	51.27 (50.87, 51.66)	54.83 (54.48, 55.17)	60.69 (60.36, 61.01)	67.71 (67.36, 68.06)	75.79 (75.37, 76.20)	83.83 (83.30, 84.35)	89.03 (88.42, 89.65)	99.83 (98.94, 100.71)
35	88	43.83 (43.18, 44.47)	51.04 (50.59, 51.49)	54.65 (54.25, 55.04)	60.59 (60.23, 60.95)	67.72 (67.32, 68.11)	75.91 (75.40, 76.41)	84.06 (83.42, 84.71)	89.35 (88.58, 90.11)	100.29 (99.22, 101.36)
36	90	43.60 (42.81, 44.39)	50.94 (50.37, 51.50)	54.61 (54.12, 55.10)	60.66 (60.22, 61.10)	67.91 (67.43, 68.40)	76.25 (75.61, 76.88)	84.55 (83.72, 85.39)	89.92 (88.94, 90.91)	101.06 (99.70, 102.43)

Female diastolic blood pressure centiles with 95% CI

	Age	_1st	_5th	_10th	_25th	_50th	_75th	_90th	_95th	_99th
1	20	45.22 (44.71, 45.73)	50.96 (50.58, 51.33)	53.83 (53.49, 54.16)	58.60 (58.29, 58.92)	64.63 (64.29, 64.97)	71.84 (71.40, 72.28)	79.17 (78.57, 79.78)	83.99 (83.25, 84.73)	94.15 (93.05, 95.26)
2	22	45.28 (44.83, 45.73)	51.13 (50.80, 51.46)	54.05 (53.76, 54.35)	58.92 (58.64, 59.21)	65.07 (64.77, 65.37)	72.42 (72.05, 72.79)	79.90 (79.39, 80.40)	84.81 (84.19, 85.42)	95.17 (94.23, 96.11)
3	24	45.35 (44.93, 45.77)	51.31 (51.02, 51.61)	54.29 (54.03, 54.56)	59.25 (59.00, 59.51)	65.51 (65.25, 65.78)	73.00 (72.68, 73.32)	80.62 (80.19, 81.05)	85.62 (85.09, 86.15)	96.18 (95.34, 97.01)
4	26	45.44 (45.04, 45.84)	51.50 (51.23, 51.77)	54.53 (54.29, 54.77)	59.58 (59.36, 59.81)	65.95 (65.72, 66.19)	73.57 (73.28, 73.85)	81.32 (80.94, 81.70)	86.41 (85.94, 86.89)	97.15 (96.38, 97.92)
5	28	45.53 (45.14, 45.92)	51.69 (51.44, 51.95)	54.77 (54.56, 54.99)	59.91 (59.70, 60.11)	66.38 (66.17, 66.59)	74.12 (73.86, 74.38)	82.00 (81.64, 82.36)	87.17 (86.73, 87.62)	98.09 (97.35, 98.82)
6	30	45.63 (45.24, 46.02)	51.89 (51.64, 52.13)	55.01 (54.81, 55.21)	60.22 (60.04, 60.40)	66.79 (66.59, 66.99)	74.65 (74.39, 74.90)	82.64 (82.30, 82.99)	87.89 (87.46, 88.33)	98.97 (98.25, 99.69)
7	32	45.74 (45.35, 46.13)	52.08 (51.84, 52.32)	55.25 (55.06, 55.44)	60.52 (60.35, 60.69)	67.18 (66.99, 67.37)	75.14 (74.90, 75.38)	83.24 (82.91, 83.58)	88.57 (88.14, 88.99)	99.79 (99.09, 100.49)
8	34	45.85 (45.46, 46.25)	52.27 (52.03, 52.50)	55.47 (55.29, 55.66)	60.81 (60.65, 60.97)	67.55 (67.36, 67.73)	75.60 (75.36, 75.84)	83.80 (83.46, 84.13)	89.18 (88.77, 89.59)	100.54 (99.85, 101.23)

For peer review only - http://bmjopen.bmj.com/site/about/guidelines.xhtml

	Age	_1st	_5th	_10th	_25th	_50th	_75th	_90th	_95th	_99th
9	36	45.97 (45.58, 46.37)	52.45 (52.21, 52.68)	55.68 (55.50, 55.87)	61.08 (60.91, 61.24)	67.88 (67.70, 68.07)	76.01 (75.78, 76.25)	84.29 (83.97, 84.62)	89.73 (89.33, 90.14)	101.20 (100.53, 101.88)
10	38	46.09 (45.69, 46.48)	52.62 (52.38, 52.86)	55.88 (55.69, 56.07)	61.32 (61.15, 61.49)	68.18 (68.00, 68.37)	76.38 (76.15, 76.62)	84.73 (84.42, 85.05)	90.22 (89.83, 90.61)	101.78 (101.13, 102.44)
11	40	46.20 (45.81, 46.60)	52.78 (52.54, 53.02)	56.07 (55.87, 56.26)	61.54 (61.37, 61.72)	68.45 (68.26, 68.64)	76.70 (76.47, 76.94)	85.11 (84.80, 85.42)	90.63 (90.25, 91.01)	102.28 (101.64, 102.92)
12	42	46.32 (45.91, 46.72)	52.93 (52.68, 53.18)	56.23 (56.03, 56.43)	61.73 (61.55, 61.92)	68.68 (68.48, 68.87)	76.98 (76.74, 77.21)	85.42 (85.12, 85.73)	90.98 (90.60, 91.35)	102.68 (102.05, 103.31)
13	44	46.43 (46.02, 46.83)	53.06 (52.80, 53.32)	56.37 (56.16, 56.59)	61.90 (61.71, 62.09)	68.87 (68.67, 69.06)	77.19 (76.96, 77.43)	85.67 (85.38, 85.97)	91.24 (90.88, 91.61)	102.99 (102.36, 103.62)
14	46	46.53 (46.11, 46.94)	53.17 (52.91, 53.44)	56.50 (56.28, 56.71)	62.03 (61.83, 62.23)	69.01 (68.81, 69.22)	77.36 (77.13, 77.59)	85.86 (85.56, 86.16)	91.44 (91.08, 91.80)	103.21 (102.59, 103.84)
15	48	46.62 (46.20, 47.04)	53.27 (53.00, 53.54)	56.59 (56.37, 56.82)	62.13 (61.93, 62.33)	69.12 (68.92, 69.33)	77.48 (77.24, 77.71)	85.98 (85.68, 86.28)	91.57 (91.20, 91.93)	103.35 (102.72, 103.98)
16	50	46.69 (46.27, 47.12)	53.34 (53.07, 53.62)	56.67 (56.44, 56.90)	62.20 (62.00, 62.41)	69.19 (68.99, 69.40)	77.54 (77.31, 77.77)	86.04 (85.74, 86.34)	91.63 (91.26, 92.00)	103.41 (102.76, 104.05)
17	52	46.76 (46.33, 47.18)	53.40 (53.12, 53.67)	56.72 (56.49, 56.95)	62.24 (62.04, 62.45)	69.22 (69.02, 69.43)	77.56 (77.32, 77.79)	86.05 (85.74, 86.35)	91.62 (91.25, 92.00)	103.38 (102.73, 104.04)
18	54	46.80 (46.38, 47.23)	53.43 (53.16, 53.70)	56.74 (56.51, 56.97)	62.25 (62.05, 62.46)	69.21 (69.01, 69.42)	77.53 (77.30, 77.77)	86.00 (85.69, 86.31)	91.56 (91.18, 91.95)	103.30 (102.63, 103.96)
19	56	46.83 (46.41, 47.25)	53.44 (53.17, 53.70)	56.74 (56.51, 56.96)	62.23 (62.04, 62.43)	69.17 (68.97, 69.37)	77.46 (77.23, 77.70)	85.91 (85.60, 86.22)	91.45 (91.06, 91.84)	103.15 (102.47, 103.83)
20	58	46.84 (46.43, 47.25)	53.42 (53.16, 53.68)	56.71 (56.49, 56.92)	62.19 (61.99, 62.38)	69.10 (68.90, 69.30)	77.36 (77.13, 77.60)	85.77 (85.46, 86.09)	91.30 (90.90, 91.69)	102.95 (102.27, 103.64)
21	60	46.82 (46.43, 47.22)	53.38 (53.13, 53.63)	56.65 (56.45, 56.86)	62.11 (61.92, 62.30)	69.00 (68.80, 69.19)	77.23 (76.99, 77.46)	85.61 (85.29, 85.92)	91.11 (90.71, 91.51)	102.72 (102.02, 103.41)
22	62	46.79 (46.40, 47.17)	53.32 (53.08, 53.55)	56.58 (56.38, 56.77)	62.01 (61.83, 62.19)	68.87 (68.68, 69.06)	77.07 (76.83, 77.30)	85.41 (85.09, 85.73)	90.89 (90.49, 91.30)	102.45 (101.76, 103.15)
23	64	46.72 (46.35, 47.09)	53.23 (53.00, 53.45)	56.48 (56.29, 56.66)	61.89 (61.72, 62.06)	68.72 (68.53, 68.91)	76.89 (76.65, 77.12)	85.20 (84.88, 85.52)	90.66 (90.26, 91.07)	102.18 (101.48, 102.88)

	Age	_1st	_5th	_10th	_25th	_50th	_75th	_90th	_95th	_99th
24	66	46.63 (46.28, 46.99)	53.12 (52.90, 53.33)	56.35 (56.18, 56.53)	61.75 (61.58, 61.92)	68.56 (68.37, 68.75)	76.70 (76.46, 76.93)	84.98 (84.66, 85.31)	90.43 (90.02, 90.83)	101.90 (101.20, 102.61)
25	68	46.52 (46.17, 46.87)	52.98 (52.78, 53.19)	56.21 (56.04, 56.38)	61.60 (61.43, 61.76)	68.39 (68.20, 68.58)	76.50 (76.26, 76.74)	84.77 (84.44, 85.09)	90.19 (89.78, 90.61)	101.64 (100.93, 102.35)
26	70	46.38 (46.03, 46.73)	52.83 (52.63, 53.04)	56.06 (55.89, 56.22)	61.43 (61.27, 61.59)	68.21 (68.02, 68.40)	76.31 (76.07, 76.56)	84.56 (84.23, 84.90)	89.98 (89.56, 90.40)	101.41 (100.69, 102.13)
27	72	46.21 (45.85, 46.57)	52.66 (52.45, 52.87)	55.89 (55.71, 56.06)	61.26 (61.09, 61.42)	68.03 (67.85, 68.22)	76.13 (75.89, 76.38)	84.38 (84.04, 84.72)	89.80 (89.37, 90.23)	101.23 (100.50, 101.95)
28	74	46.02 (45.65, 46.38)	52.48 (52.25, 52.70)	55.70 (55.52, 55.88)	61.08 (60.92, 61.25)	67.87 (67.68, 68.06)	75.98 (75.73, 76.23)	84.24 (83.89, 84.58)	89.66 (89.23, 90.10)	101.10 (100.37, 101.84)
29	76	45.79 (45.40, 46.18)	52.28 (52.04, 52.51)	55.51 (55.32, 55.71)	60.91 (60.74, 61.08)	67.72 (67.53, 67.91)	75.86 (75.61, 76.11)	84.14 (83.79, 84.50)	89.59 (89.14, 90.03)	101.07 (100.32, 101.81)
30	78	45.54 (45.13, 45.96)	52.06 (51.80, 52.32)	55.32 (55.11, 55.53)	60.75 (60.57, 60.92)	67.60 (67.41, 67.78)	75.78 (75.53, 76.03)	84.11 (83.76, 84.47)	89.59 (89.14, 90.04)	101.13 (100.38, 101.88)
31	80	45.27 (44.83, 45.71)	51.84 (51.56, 52.13)	55.13 (54.89, 55.36)	60.60 (60.41, 60.79)	67.51 (67.32, 67.69)	75.76 (75.51, 76.00)	84.16 (83.81, 84.51)	89.68 (89.24, 90.13)	101.32 (100.57, 102.08)
32	82	44.96 (44.49, 45.44)	51.61 (51.30, 51.93)	54.94 (54.68, 55.19)	60.47 (60.27, 60.67)	67.46 (67.27, 67.65)	75.81 (75.56, 76.05)	84.31 (83.96, 84.66)	89.89 (89.45, 90.33)	101.67 (100.92, 102.41)
33	84	44.63 (44.12, 45.14)	51.38 (51.03, 51.73)	54.75 (54.47, 55.04)	60.37 (60.15, 60.59)	67.46 (67.27, 67.66)	75.94 (75.69, 76.18)	84.57 (84.22, 84.91)	90.23 (89.79, 90.67)	102.19 (101.45, 102.92)
34	86	44.27 (43.72, 44.81)	51.14 (50.76, 51.53)	54.58 (54.26, 54.90)	60.31 (60.06, 60.55)	67.53 (67.31, 67.75)	76.17 (75.91, 76.43)	84.96 (84.60, 85.32)	90.73 (90.29, 91.18)	102.91 (102.18, 103.65)
35	88	43.87 (43.27, 44.46)	50.90 (50.47, 51.34)	54.42 (54.06, 54.79)	60.28 (59.99, 60.57)	67.67 (67.42, 67.93)	76.51 (76.21, 76.81)	85.51 (85.11, 85.90)	91.42 (90.93, 91.90)	103.88 (103.12, 104.64)
36	90	43.43 (42.76, 44.10)	50.66 (50.17, 51.16)	54.28 (53.86, 54.70)	60.30 (59.96, 60.64)	67.90 (67.59, 68.22)	76.99 (76.61, 77.36)	86.24 (85.75, 86.73)	92.32 (91.73, 92.90)	105.13 (104.27, 105.99)

Male pulse pressure centiles with 95% CI

	Age	1st	5th	10th	25th	50th	75th	90th	95th	99th
1	20	31.75 (30.29, 33.22)	39.16 (38.33, 39.99)	42.78 (42.07, 43.49)	48.59 (47.94, 49.25)	55.24 (54.63, 55.85)	62.62 (62.06, 63.19)	69.94 (69.29, 70.58)	74.70 (73.92, 75.48)	84.71 (83.29, 86.13)
2	22	31.54 (30.33, 32.75)	38.84 (38.21, 39.48)	42.40 (41.87, 42.93)	48.12 (47.62, 48.61)	54.69 (54.21, 55.18)	62.06 (61.55, 62.56)	69.38 (68.77, 69.99)	74.17 (73.44, 74.90)	84.28 (83.02, 85.54)
3	24	31.28 (30.24, 32.32)	38.47 (37.96, 38.99)	41.97 (41.55, 42.38)	47.59 (47.19, 47.99)	54.09 (53.67, 54.51)	61.43 (60.94, 61.93)	68.78 (68.12, 69.43)	73.59 (72.79, 74.39)	83.81 (82.46, 85.15)
4	26	30.95 (29.96, 31.95)	38.04 (37.51, 38.57)	41.47 (41.04, 41.91)	47.00 (46.59, 47.41)	53.43 (53.00, 53.87)	60.75 (60.22, 61.29)	68.12 (67.41, 68.82)	72.96 (72.10, 73.82)	83.29 (81.85, 84.74)
5	28	30.57 (29.63, 31.52)	37.56 (37.05, 38.06)	40.93 (40.52, 41.35)	46.37 (45.99, 46.74)	52.74 (52.34, 53.14)	60.05 (59.56, 60.54)	67.43 (66.77, 68.09)	72.31 (71.49, 73.14)	82.78 (81.31, 84.24)
6	30	30.16 (29.32, 30.99)	37.05 (36.62, 37.47)	40.37 (40.02, 40.72)	45.72 (45.38, 46.06)	52.04 (51.66, 52.41)	59.34 (58.88, 59.80)	66.76 (66.15, 67.38)	71.69 (70.89, 72.49)	82.30 (80.80, 83.80)
7	32	29.72 (28.96, 30.48)	36.53 (36.12, 36.94)	39.81 (39.44, 40.18)	45.08 (44.69, 45.47)	51.36 (50.93, 51.80)	58.68 (58.18, 59.18)	66.15 (65.50, 66.80)	71.13 (70.31, 71.96)	81.92 (80.35, 83.49)
8	34	29.29 (28.56, 30.02)	36.04 (35.64, 36.43)	39.28 (38.93, 39.62)	44.49 (44.14, 44.84)	50.75 (50.37, 51.13)	58.10 (57.65, 58.54)	65.64 (65.04, 66.24)	70.69 (69.90, 71.47)	81.67 (80.10, 83.24)
9	36	28.89 (28.17, 29.62)	35.60 (35.19, 36.02)	38.81 (38.45, 39.17)	43.98 (43.65, 44.31)	50.24 (49.90, 50.58)	57.64 (57.24, 58.04)	65.27 (64.72, 65.83)	70.40 (69.65, 71.14)	81.62 (80.09, 83.15)
10	38	28.53 (27.82, 29.25)	35.23 (34.78, 35.67)	38.42 (38.04, 38.81)	43.57 (43.22, 43.92)	49.85 (49.50, 50.21)	57.32 (56.91, 57.74)	65.07 (64.51, 65.63)	70.29 (69.56, 71.03)	81.79 (80.31, 83.26)
11	40	28.18 (27.51, 28.85)	34.89 (34.44, 35.34)	38.09 (37.69, 38.49)	43.24 (42.87, 43.61)	49.57 (49.19, 49.96)	57.15 (56.70, 57.60)	65.04 (64.44, 65.64)	70.38 (69.62, 71.14)	82.18 (80.74, 83.63)
12	42	27.82 (27.18, 28.45)	34.58 (34.13, 35.03)	37.79 (37.38, 38.20)	42.98 (42.59, 43.37)	49.40 (48.98, 49.81)	57.11 (56.62, 57.61)	65.18 (64.54, 65.82)	70.66 (69.86, 71.46)	82.82 (81.38, 84.26)
13	44	27.44 (26.81, 28.07)	34.29 (33.84, 34.75)	37.54 (37.13, 37.96)	42.79 (42.40, 43.18)	49.33 (48.91, 49.74)	57.22 (56.72, 57.72)	65.50 (64.85, 66.15)	71.14 (70.33, 71.94)	83.69 (82.25, 85.13)
14	46	27.09 (26.43, 27.74)	34.05 (33.59, 34.51)	37.35 (36.94, 37.76)	42.69 (42.32, 43.06)	49.38 (48.99, 49.77)	57.49 (57.02, 57.96)	66.00 (65.39, 66.62)	71.81 (71.04, 72.58)	84.77 (83.35, 86.19)

For peer review only - http://bmjopen.bmj.com/site/about/guidelines.xhtml

	Age	1st	5th	10th	25th	50th	75th	90th	95th	99th
15	48	26.77 (26.09, 27.46)	33.86 (33.40, 34.32)	37.23 (36.83, 37.63)	42.69 (42.34, 43.05)	49.56 (49.20, 49.93)	57.91 (57.47, 58.35)	66.69 (66.11, 67.26)	72.67 (71.95, 73.40)	86.02 (84.64, 87.40)
16	50	26.52 (25.81, 27.23)	33.75 (33.30, 34.21)	37.20 (36.81, 37.59)	42.80 (42.46, 43.15)	49.88 (49.52, 50.24)	58.49 (58.06, 58.93)	67.54 (66.98, 68.09)	73.69 (73.00, 74.38)	87.38 (86.02, 88.74)
17	52	26.37 (25.65, 27.09)	33.73 (33.29, 34.18)	37.26 (36.89, 37.64)	43.03 (42.69, 43.36)	50.33 (49.97, 50.69)	59.21 (58.77, 59.66)	68.52 (67.96, 69.08)	74.83 (74.14, 75.52)	88.79 (87.43, 90.15)
18	54	26.33 (25.62, 27.04)	33.81 (33.37, 34.24)	37.42 (37.05, 37.79)	43.36 (43.03, 43.68)	50.90 (50.54, 51.26)	60.06 (59.60, 60.51)	69.61 (69.03, 70.18)	76.05 (75.34, 76.75)	90.15 (88.78, 91.52)
19	56	26.39 (25.70, 27.09)	33.96 (33.53, 34.39)	37.66 (37.30, 38.02)	43.78 (43.46, 44.09)	51.56 (51.20, 51.91)	60.98 (60.53, 61.43)	70.74 (70.16, 71.33)	77.28 (76.55, 78.00)	91.43 (90.06, 92.79)
20	58	26.49 (25.80, 27.17)	34.14 (33.71, 34.58)	37.93 (37.57, 38.30)	44.23 (43.91, 44.55)	52.25 (51.91, 52.60)	61.93 (61.49, 62.37)	71.89 (71.30, 72.48)	78.49 (77.75, 79.23)	92.59 (91.25, 93.93)
21	60	26.58 (25.90, 27.26)	34.32 (33.88, 34.76)	38.19 (37.82, 38.56)	44.68 (44.36, 45.00)	52.95 (52.61, 53.29)	62.88 (62.44, 63.32)	73.01 (72.41, 73.61)	79.66 (78.91, 80.42)	93.68 (92.38, 94.98)
22	62	26.71 (26.04, 27.39)	34.54 (34.09, 34.99)	38.51 (38.13, 38.89)	45.20 (44.87, 45.52)	53.71 (53.38, 54.05)	63.89 (63.46, 64.33)	74.18 (73.58, 74.79)	80.88 (80.13, 81.63)	94.79 (93.54, 96.04)
23	64	26.94 (26.28, 27.60)	34.86 (34.41, 35.32)	38.93 (38.54, 39.32)	45.82 (45.49, 46.15)	54.58 (54.26, 54.91)	65.01 (64.59, 65.42)	75.46 (74.88, 76.04)	82.21 (81.49, 82.93)	96.03 (94.86, 97.20)
24	66	27.24 (26.62, 27.86)	35.28 (34.84, 35.73)	39.45 (39.07, 39.84)	46.55 (46.21, 46.88)	55.57 (55.24, 55.89)	66.23 (65.84, 66.63)	76.86 (76.31, 77.40)	83.66 (82.98, 84.33)	97.42 (96.33, 98.52)
25	68	27.58 (26.98, 28.18)	35.76 (35.33, 36.19)	40.04 (39.66, 40.41)	47.35 (47.02, 47.68)	56.62 (56.30, 56.94)	67.53 (67.14, 67.92)	78.33 (77.81, 78.86)	85.19 (84.55, 85.84)	98.95 (97.89, 100.01)
26	70	27.85 (27.23, 28.47)	36.19 (35.76, 36.62)	40.59 (40.21, 40.96)	48.11 (47.79, 48.44)	57.63 (57.31, 57.95)	68.80 (68.41, 69.18)	79.78 (79.25, 80.31)	86.71 (86.05, 87.37)	100.50 (99.41, 101.59)
27	72	27.98 (27.30, 28.65)	36.50 (36.04, 36.96)	41.01 (40.62, 41.40)	48.76 (48.43, 49.10)	58.54 (58.20, 58.87)	69.94 (69.53, 70.35)	81.11 (80.55, 81.67)	88.13 (87.43, 88.82)	101.97 (100.83, 103.12)
28	74	27.93 (27.22, 28.63)	36.65 (36.17, 37.13)	41.29 (40.88, 41.70)	49.27 (48.90, 49.64)	59.29 (58.92, 59.67)	70.95 (70.51, 71.39)	82.32 (81.73, 82.90)	89.42 (88.70, 90.15)	103.37 (102.17, 104.56)
29	76	27.72 (27.03, 28.42)	36.65 (36.18, 37.12)	41.42 (41.01, 41.82)	49.62 (49.25, 50.00)	59.91 (59.52, 60.29)	71.82 (71.37, 72.26)	83.38 (82.78, 83.98)	90.60 (89.84, 91.35)	104.67 (103.41, 105.92)

	Age	1st	5th	10th	25th	50th	75th	90th	95th	99th
30	78	27.42 (26.71, 28.12)	36.54 (36.05, 37.04)	41.43 (41.01, 41.85)	49.86 (49.47, 50.24)	60.38 (59.98, 60.78)	72.54 (72.07, 73.02)	84.32 (83.65, 84.99)	91.64 (90.78, 92.50)	105.86 (104.46, 107.27)
31	80	27.13 (26.35, 27.91)	36.43 (35.85, 37.00)	41.42 (40.91, 41.93)	50.04 (49.57, 50.50)	60.79 (60.32, 61.26)	73.19 (72.63, 73.75)	85.17 (84.39, 85.95)	92.60 (91.61, 93.59)	106.99 (105.41, 108.57)
32	82	26.96 (26.14, 27.78)	36.40 (35.77, 37.02)	41.47 (40.91, 42.04)	50.25 (49.73, 50.77)	61.21 (60.69, 61.73)	73.83 (73.23, 74.42)	86.00 (85.18, 86.83)	93.54 (92.50, 94.58)	108.10 (106.45, 109.75)
33	84	26.93 (26.07, 27.78)	36.46 (35.83, 37.09)	41.60 (41.04, 42.16)	50.50 (49.98, 51.03)	61.63 (61.11, 62.16)	74.46 (73.86, 75.05)	86.82 (86.00, 87.64)	94.46 (93.43, 95.49)	109.19 (107.55, 110.82)
34	86	26.97 (25.99, 27.95)	36.57 (35.90, 37.24)	41.76 (41.19, 42.33)	50.76 (50.23, 51.29)	62.04 (61.49, 62.59)	75.05 (74.41, 75.69)	87.58 (86.70, 88.47)	95.32 (94.21, 96.44)	110.21 (108.50, 111.92)
35	88	27.03 (25.79, 28.27)	36.67 (35.88, 37.45)	41.89 (41.26, 42.51)	50.95 (50.42, 51.49)	62.35 (61.79, 62.92)	75.52 (74.80, 76.24)	88.20 (87.15, 89.25)	96.02 (94.71, 97.34)	111.05 (109.09, 113.02)
36	90	27.07 (25.45, 28.70)	36.72 (35.70, 37.74)	41.96 (41.18, 42.74)	51.07 (50.45, 51.69)	62.56 (61.94, 63.18)	75.85 (75.03, 76.66)	88.65 (87.41, 89.89)	96.54 (94.96, 98.11)	111.68 (109.35, 114.00)

Female PP centiles with 95% CI

	Age	1st	5th	10th	25th	50th	75th	90th	95th	99th
1	20	27.29 (26.29, 28.28)	33.96 (33.35, 34.58)	37.06 (36.57, 37.55)	41.94 (41.54, 42.34)	47.71 (47.21, 48.21)	54.50 (53.81, 55.19)	61.58 (60.71, 62.45)	66.42 (65.43, 67.42)	77.33 (75.64, 79.01)
2	22	27.09 (26.25, 27.93)	33.72 (33.20, 34.24)	36.80 (36.38, 37.22)	41.65 (41.32, 41.98)	47.40 (47.04, 47.76)	54.18 (53.70, 54.65)	61.26 (60.63, 61.88)	66.10 (65.34, 66.86)	77.01 (75.55, 78.46)
3	24	26.89 (26.14, 27.64)	33.48 (33.02, 33.94)	36.54 (36.17, 36.91)	41.37 (41.08, 41.66)	47.11 (46.82, 47.40)	53.88 (53.52, 54.25)	60.97 (60.45, 61.49)	65.82 (65.14, 66.49)	76.74 (75.40, 78.09)
4	26	26.70 (26.00, 27.40)	33.26 (32.84, 33.68)	36.31 (35.97, 36.65)	41.12 (40.85, 41.39)	46.86 (46.59, 47.13)	53.64 (53.30, 53.98)	60.74 (60.23, 61.25)	65.61 (64.92, 66.29)	76.57 (75.26, 77.88)
5	28	26.54 (25.86, 27.21)	33.08 (32.68, 33.48)	36.12 (35.80, 36.43)	40.93 (40.66, 41.19)	46.67 (46.40, 46.94)	53.49 (53.13, 53.84)	60.63 (60.09, 61.16)	65.52 (64.81, 66.23)	76.54 (75.25, 77.84)
6	30	26.41 (25.75, 27.07)	32.95 (32.56, 33.34)	35.99 (35.68, 36.30)	40.81 (40.55, 41.07)	46.59 (46.32, 46.86)	53.46 (53.10, 53.82)	60.67 (60.12, 61.21)	65.60 (64.88, 66.32)	76.73 (75.46, 78.01)
7	32	26.30 (25.66, 26.95)	32.87 (32.48, 33.26)	35.92 (35.61, 36.24)	40.77 (40.50, 41.04)	46.61 (46.34, 46.88)	53.57 (53.23, 53.91)	60.87 (60.35, 61.39)	65.88 (65.19, 66.57)	77.17 (75.91, 78.43)

For peer review only - http://bmjopen.bmj.com/site/about/guidelines.xhtml

	Age	1st	5th	10th	25th	50th	75th	90th	95th	99th
8	34	26.20 (25.58, 26.83)	32.82 (32.43, 33.21)	35.90 (35.57, 36.23)	40.80 (40.51, 41.09)	46.73 (46.45, 47.01)	53.81 (53.49, 54.14)	61.26 (60.77, 61.74)	66.36 (65.71, 67.01)	77.86 (76.62, 79.11)
9	36	26.09 (25.46, 26.71)	32.79 (32.39, 33.18)	35.91 (35.57, 36.26)	40.89 (40.57, 41.20)	46.94 (46.65, 47.23)	54.19 (53.88, 54.51)	61.82 (61.37, 62.27)	67.05 (66.44, 67.66)	78.83 (77.56, 80.11)
10	38	25.96 (25.29, 26.62)	32.77 (32.36, 33.17)	35.95 (35.59, 36.31)	41.03 (40.70, 41.36)	47.25 (46.95, 47.54)	54.71 (54.41, 55.01)	62.56 (62.15, 62.98)	67.95 (67.35, 68.55)	80.07 (78.66, 81.49)
11	40	25.81 (25.07, 26.55)	32.76 (32.35, 33.17)	36.02 (35.65, 36.38)	41.23 (40.90, 41.56)	47.64 (47.35, 47.94)	55.36 (55.06, 55.66)	63.48 (63.05, 63.92)	69.05 (68.38, 69.71)	81.56 (79.84, 83.29)
12	42	25.68 (24.86, 26.51)	32.79 (32.37, 33.21)	36.13 (35.77, 36.49)	41.50 (41.18, 41.82)	48.14 (47.85, 48.43)	56.14 (55.82, 56.47)	64.57 (64.05, 65.09)	70.33 (69.52, 71.15)	83.27 (81.15, 85.39)
13	44	25.62 (24.75, 26.50)	32.89 (32.47, 33.31)	36.33 (35.98, 36.68)	41.86 (41.56, 42.17)	48.74 (48.45, 49.03)	57.06 (56.69, 57.43)	65.80 (65.19, 66.42)	71.77 (70.82, 72.72)	85.12 (82.74, 87.50)
14	46	25.65 (24.75, 26.55)	33.08 (32.65, 33.52)	36.61 (36.26, 36.96)	42.33 (42.03, 42.62)	49.46 (49.15, 49.76)	58.09 (57.67, 58.51)	67.15 (66.47, 67.83)	73.32 (72.30, 74.33)	87.04 (84.66, 89.43)
15	48	25.79 (24.85, 26.72)	33.37 (32.89, 33.84)	36.99 (36.62, 37.36)	42.88 (42.58, 43.18)	50.27 (49.94, 50.59)	59.21 (58.74, 59.67)	68.56 (67.83, 69.30)	74.91 (73.89, 75.94)	88.95 (86.75, 91.16)
16	50	26.01 (25.03, 27.00)	33.73 (33.21, 34.26)	37.45 (37.05, 37.84)	43.51 (43.21, 43.82)	51.15 (50.81, 51.48)	60.38 (59.88, 60.89)	70.01 (69.23, 70.79)	76.51 (75.47, 77.55)	90.80 (88.81, 92.78)
17	52	26.31 (25.27, 27.35)	34.16 (33.59, 34.73)	37.97 (37.55, 38.39)	44.22 (43.91, 44.52)	52.09 (51.76, 52.43)	61.61 (61.08, 62.14)	71.48 (70.66, 72.30)	78.12 (77.06, 79.17)	92.58 (90.77, 94.38)
18	54	26.65 (25.59, 27.71)	34.64 (34.03, 35.24)	38.54 (38.09, 38.99)	44.97 (44.65, 45.29)	53.09 (52.76, 53.42)	62.87 (62.34, 63.40)	72.98 (72.15, 73.80)	79.72 (78.67, 80.78)	94.31 (92.64, 95.97)
19	56	27.01 (25.99, 28.03)	35.14 (34.54, 35.74)	39.15 (38.69, 39.60)	45.77 (45.43, 46.11)	54.13 (53.79, 54.46)	64.17 (63.64, 64.69)	74.48 (73.66, 75.30)	81.33 (80.30, 82.36)	96.00 (94.44, 97.56)
20	58	27.38 (26.43, 28.33)	35.66 (35.10, 36.23)	39.78 (39.33, 40.22)	46.60 (46.24, 46.96)	55.20 (54.85, 55.55)	65.49 (64.97, 66.00)	76.00 (75.21, 76.79)	82.94 (81.94, 83.93)	97.66 (96.17, 99.16)
21	60	27.76 (26.88, 28.63)	36.21 (35.68, 36.73)	40.43 (40.00, 40.87)	47.47 (47.10, 47.84)	56.31 (55.94, 56.68)	66.84 (66.34, 67.35)	77.55 (76.81, 78.28)	84.57 (83.65, 85.49)	99.34 (97.93, 100.75)
22	62	28.14 (27.32, 28.96)	36.78 (36.28, 37.28)	41.12 (40.70, 41.55)	48.38 (47.99, 48.77)	57.46 (57.08, 57.85)	68.24 (67.75, 68.73)	79.13 (78.45, 79.80)	86.23 (85.40, 87.05)	101.05 (99.76, 102.35)

	Age	1st	5th	10th	25th	50th	75th	90th	95th	99th
23	64	28.54 (27.74, 29.34)	37.37 (36.88, 37.86)	41.84 (41.42, 42.26)	49.31 (48.92, 49.70)	58.64 (58.24, 59.03)	69.64 (69.16, 70.12)	80.70 (80.07, 81.33)	87.87 (87.12, 88.63)	102.75 (101.55, 103.94)
24	66	28.96 (28.17, 29.76)	37.99 (37.51, 38.47)	42.58 (42.17, 42.98)	50.26 (49.88, 50.63)	59.80 (59.42, 60.19)	71.01 (70.55, 71.47)	82.22 (81.64, 82.81)	89.47 (88.78, 90.15)	104.37 (103.28, 105.46)
25	68	29.38 (28.58, 30.19)	38.60 (38.12, 39.07)	43.30 (42.91, 43.69)	51.19 (50.84, 51.54)	60.95 (60.58, 61.31)	72.35 (71.92, 72.79)	83.71 (83.16, 84.26)	91.01 (90.37, 91.65)	105.95 (104.93, 106.96)
26	70	29.75 (28.92, 30.58)	39.16 (38.68, 39.65)	43.99 (43.59, 44.39)	52.08 (51.73, 52.44)	62.05 (61.68, 62.42)	73.65 (73.20, 74.10)	85.15 (84.59, 85.72)	92.52 (91.85, 93.18)	107.50 (106.43, 108.58)
27	72	30.01 (29.12, 30.89)	39.63 (39.10, 40.16)	44.57 (44.13, 45.02)	52.88 (52.47, 53.30)	63.07 (62.64, 63.51)	74.88 (74.37, 75.38)	86.54 (85.90, 87.18)	93.98 (93.22, 94.74)	109.05 (107.74, 110.35)
28	74	30.08 (29.12, 31.04)	39.92 (39.34, 40.51)	45.00 (44.50, 45.50)	53.53 (53.06, 54.01)	63.96 (63.47, 64.46)	75.99 (75.42, 76.56)	87.84 (87.11, 88.56)	95.37 (94.48, 96.26)	110.55 (108.98, 112.12)
29	76	29.93 (28.92, 30.95)	40.01 (39.37, 40.65)	45.22 (44.68, 45.76)	53.99 (53.50, 54.47)	64.66 (64.15, 65.17)	76.93 (76.33, 77.53)	88.97 (88.19, 89.74)	96.60 (95.65, 97.56)	111.93 (110.29, 113.57)
30	78	29.60 (28.51, 30.70)	39.90 (39.18, 40.63)	45.25 (44.65, 45.85)	54.24 (53.75, 54.73)	65.16 (64.65, 65.66)	77.67 (77.05, 78.28)	89.90 (89.10, 90.71)	97.65 (96.65, 98.64)	113.13 (111.50, 114.76)
31	80	29.18 (27.99, 30.36)	39.69 (38.87, 40.50)	45.15 (44.48, 45.83)	54.35 (53.83, 54.88)	65.51 (65.00, 66.03)	78.26 (77.64, 78.87)	90.69 (89.88, 91.50)	98.54 (97.55, 99.53)	114.18 (112.57, 115.79)
32	82	28.73 (27.53, 29.93)	39.43 (38.58, 40.28)	45.01 (44.30, 45.71)	54.40 (53.86, 54.95)	65.79 (65.28, 66.29)	78.75 (78.17, 79.34)	91.38 (90.63, 92.13)	99.33 (98.41, 100.25)	115.12 (113.58, 116.66)
33	84	28.31 (27.16, 29.47)	39.18 (38.36, 40.01)	44.86 (44.17, 45.55)	54.44 (53.91, 54.98)	66.03 (65.55, 66.51)	79.21 (78.67, 79.75)	92.02 (91.31, 92.72)	100.06 (99.19, 100.93)	116.00 (114.50, 117.51)
34	86	27.92 (26.76, 29.07)	38.94 (38.14, 39.74)	44.72 (44.04, 45.39)	54.46 (53.93, 55.00)	66.25 (65.76, 66.74)	79.62 (79.06, 80.19)	92.60 (91.85, 93.35)	100.73 (99.80, 101.67)	116.81 (115.23, 118.39)
35	88	27.50 (26.22, 28.78)	38.67 (37.81, 39.53)	44.53 (43.81, 45.26)	54.44 (53.84, 55.05)	66.41 (65.84, 66.98)	79.97 (79.31, 80.62)	93.09 (92.22, 93.96)	101.31 (100.24, 102.38)	117.50 (115.72, 119.27)
36	90	27.00 (25.48, 28.51)	38.33 (37.33, 39.33)	44.28 (43.44, 45.12)	54.35 (53.63, 55.07)	66.49 (65.80, 67.18)	80.22 (79.47, 80.96)	93.48 (92.50, 94.46)	101.76 (100.54, 102.98)	118.05 (116.04, 120.07)

For peer review only - http://bmjopen.bmj.com/site/about/guidelines.xhtml

Appendix B - Diastolic and Pulse Pressure Centiles for Emergency/Elective cohorts

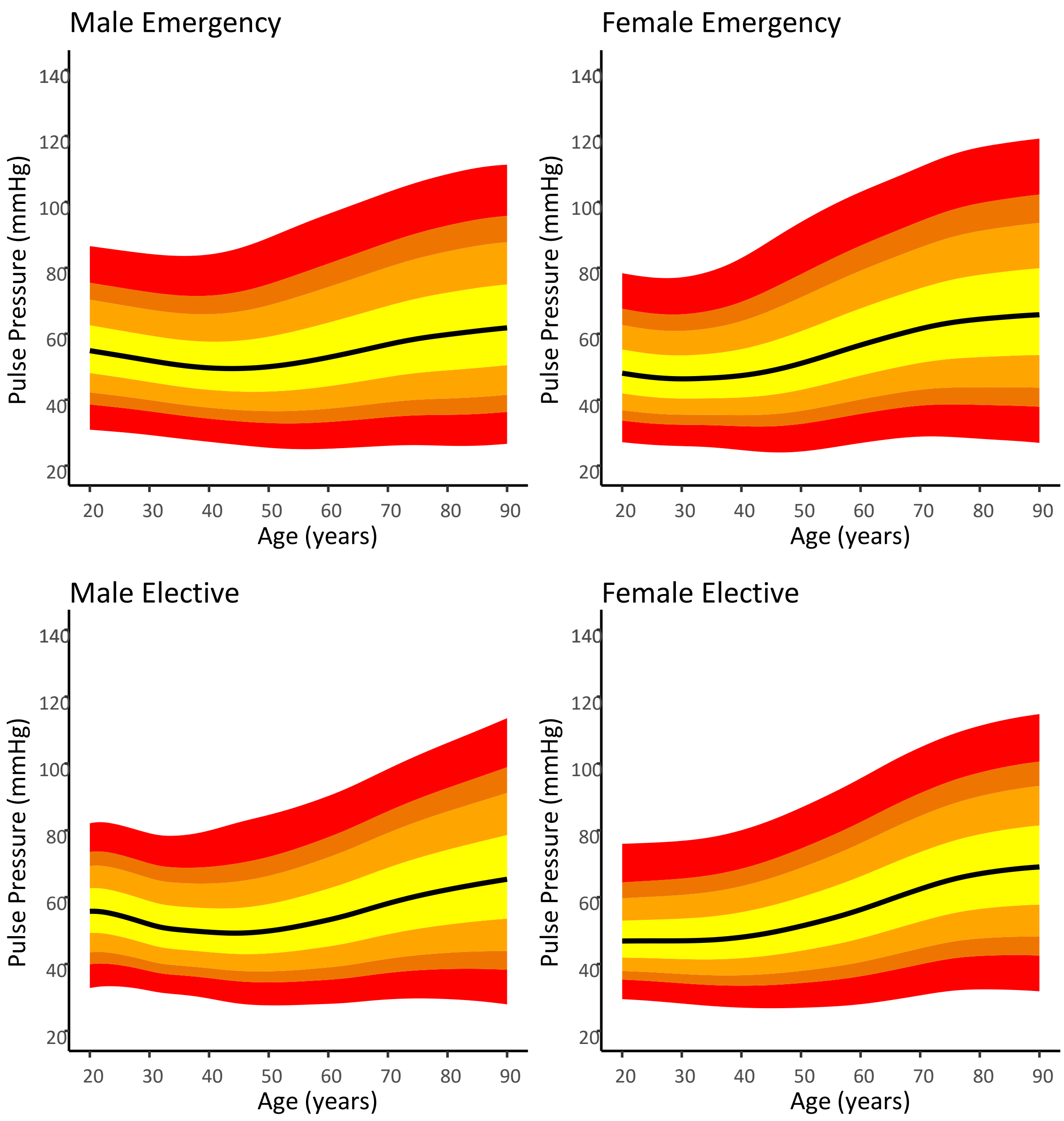

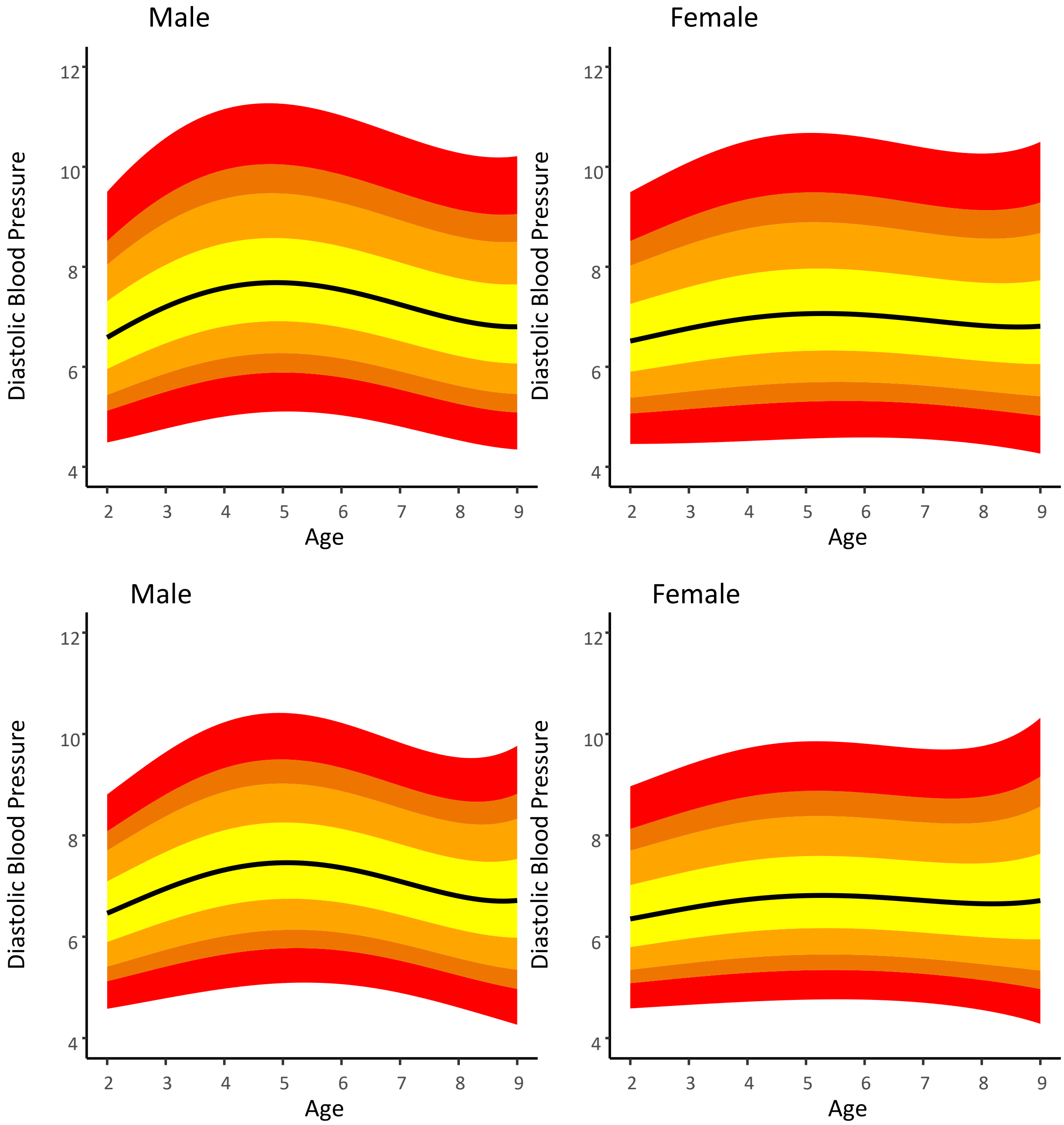
Male
Female
Diastolic Blood Pressure
12
10
8
6
4
2
3
4
5
6
7
8
9
Age
Male
Female
Diastolic Blood Pressure
Age

The RECORD statement – checklist of items, extended from the STROBE statement, that should be reported in observational studies using routinely collected health data.

	Item No.	STROBE items	Location in manuscript where items are reported	RECORD items	Location in manuscript where items are reported
**Title and abstract**					
	1	(a) Indicate the study's design with a commonly used term in the title or the abstract (b) Provide in the abstract an informative and balanced summary of what was done and what was found	yes	RECORD 1.1: The type of data used should be specified in the title or abstract. When possible, the name of the databases used should be included.<br><br>RECORD 1.2: If applicable, the geographic region and timeframe within which the study took place should be reported in the title or abstract.<br><br>RECORD 1.3: If linkage between databases was conducted for the study, this should be clearly stated in the title or abstract.	yes<br><br>Abstract<br><br>N/A
**Introduction**					
Background rationale	2	Explain the scientific background and rationale for the investigation being reported			Introduction
Objectives	3	State specific objectives, including any prespecified hypotheses			Introduction
**Methods**					
Study Design	4	Present key elements of study design early in the paper			Methods – paragraph 1
Setting	5	Describe the setting, locations, and relevant dates, including periods of recruitment, exposure, follow-up, and data collection			Methods – paragraph 1, 3

For peer review only

Participants	6	*(a) Cohort study* - Give the eligibility criteria, and the sources and methods of selection of participants. Describe methods of follow-up<br>*Case-control study* - Give the eligibility criteria, and the sources and methods of case ascertainment and control selection. Give the rationale for the choice of cases and controls<br>*Cross-sectional study* - Give the eligibility criteria, and the sources and methods of selection of participants<br><br>*(b) Cohort study* - For matched studies, give matching criteria and number of exposed and unexposed<br>*Case-control study* - For matched studies, give matching criteria and the number of controls per case	Methods – paragraph 2	RECORD 6.1: The methods of study population selection (such as codes or algorithms used to identify subjects) should be listed in detail. If this is not possible, an explanation should be provided.<br><br>RECORD 6.2: Any validation studies of the codes or algorithms used to select the population should be referenced. If validation was conducted for this study and not published elsewhere, detailed methods and results should be provided.<br><br>RECORD 6.3: If the study involved linkage of databases, consider use of a flow diagram or other graphical display to demonstrate the data linkage process, including the number of individuals with linked data at each stage.	Methods – paragraph 2, 4<br><br>Method – paragraph 4 (ref 12)<br><br>N/A
Variables	7	Clearly define all outcomes, exposures, predictors, potential confounders, and effect modifiers. Give diagnostic criteria, if applicable.		RECORD 7.1: A complete list of codes and algorithms used to classify exposures, outcomes, confounders, and effect modifiers should be provided. If these cannot be reported, an explanation should be provided.	Methods paragraph 3-4 (no codes required for blood pressure, as retrieved from curated system, which was itself taken from our own in-house system, SEND)
Data sources/ measurement	8	For each variable of interest, give sources of data and details of methods of assessment (measurement).			Methods Paragraph 3

For peer review only

		Describe comparability of assessment methods if there is more than one group			
Bias	9	Describe any efforts to address potential sources of bias			N/A – whole population is used (no sampling)
Study size	10	Explain how the study size was arrived at			Methods -> sample size
Quantitative variables	11	Explain how quantitative variables were handled in the analyses. If applicable, describe which groupings were chosen, and why			Methods -> Analysis
Statistical methods	12	(a) Describe all statistical methods, including those used to control for confounding<br>(b) Describe any methods used to examine subgroups and interactions<br>(c) Explain how missing data were addressed<br>(d) *Cohort study* - If applicable, explain how loss to follow-up was addressed<br>*Case-control study* - If applicable, explain how matching of cases and controls was addressed<br>*Cross-sectional study* - If applicable, describe analytical methods taking account of sampling strategy<br>(e) Describe any sensitivity analyses			Methods -> Analysis
Data access and cleaning methods		..		RECORD 12.1: Authors should describe the extent to which the investigators had access to the database	Methods: Paragraph 3

For peer review only

				population used to create the study population.<br><br>RECORD 12.2: Authors should provide information on the data cleaning methods used in the study.	Methods: Paragraph 3
Linkage		..		RECORD 12.3: State whether the study included person-level, institutional-level, or other data linkage across two or more databases. The methods of linkage and methods of linkage quality evaluation should be provided.	N/A
**Results**					
Participants	13	(a) Report the numbers of individuals at each stage of the study (*e.g.*, numbers potentially eligible, examined for eligibility, confirmed eligible, included in the study, completing follow-up, and analysed)<br>(b) Give reasons for non-participation at each stage.<br>(c) Consider use of a flow diagram		RECORD 13.1: Describe in detail the selection of the persons included in the study (*i.e.,* study population selection) including filtering based on data quality, data availability and linkage. The selection of included persons can be described in the text and/or by means of the study flow diagram.	Figure 1
Descriptive data	14	(a) Give characteristics of study participants (*e.g.*, demographic, clinical, social) and information on exposures and potential confounders<br>(b) Indicate the number of participants with missing data for each variable of interest<br>(c) *Cohort study* - summarise follow-up time (*e.g.*, average and total amount)			Table 1

For peer review only

Outcome data	15	*Cohort study* - Report numbers of outcome events or summary measures over time<br>*Case-control study* - Report numbers in each exposure category, or summary measures of exposure<br>*Cross-sectional study* - Report numbers of outcome events or summary measures			N/A (one measurement per admission)<br><br>Primary result: Fig 1
Main results	16	(a) Give unadjusted estimates and, if applicable, confounder-adjusted estimates and their precision (e.g., 95% confidence interval). Make clear which confounders were adjusted for and why they were included<br>(b) Report category boundaries when continuous variables were categorized<br>(c) If relevant, consider translating estimates of relative risk into absolute risk for a meaningful time period			(a) 95CIs reported throughout Results for pertinent values. Full table of CIs in supplementary material<br>(b) No categorisation for centile plots<br>(c) N/A
Other analyses	17	Report other analyses done—e.g., analyses of subgroups and interactions, and sensitivity analyses			Results Fig 3, 4
**Discussion**					
Key results	18	Summarise key results with reference to study objectives			Discussion
Limitations	19	Discuss limitations of the study, taking into account sources of		RECORD 19.1: Discuss the implications of using data that were not	Discussion -> Limitations

For peer review only

		potential bias or imprecision. Discuss both direction and magnitude of any potential bias		created or collected to answer the specific research question(s). Include discussion of misclassification bias, unmeasured confounding, missing data, and changing eligibility over time, as they pertain to the study being reported.	
Interpretation	20	Give a cautious overall interpretation of results considering objectives, limitations, multiplicity of analyses, results from similar studies, and other relevant evidence			Discussion -> Interpretation
Generalisability	21	Discuss the generalisability (external validity) of the study results			Discussion -> Generalisability
**Other Information**					
Funding	22	Give the source of funding and the role of the funders for the present study and, if applicable, for the original study on which the present article is based			Sources of Funding
Accessibility of protocol, raw data, and programming code		..		RECORD 22.1: Authors should provide information on how to access any supplemental information such as the study protocol, raw data, or programming code.	The raw data used for this research are not openly available.

*Reference: Benchimol EI, Smeeth L, Guttmann A, Harron K, Moher D, Petersen I, Sørensen HT, von Elm E, Langan SM, the RECORD Working Committee. The REporting of studies Conducted using Observational Routinely-collected health Data (RECORD) Statement. *PLoS Medicine* 2015; in press.

*Checklist is protected under Creative Commons Attribution (CC BY) license.

For peer review only

BMJ Open

Prevalence of Multimorbidity with Frailty and Associations with Socioeconomic Position in an Adult Population: Findings from the Cross-sectional HUNT Study in Norway.

Journal:	*BMJ Open*
Manuscript ID	bmjopen-2019-035070.R2
Article Type:	Original research
Date Submitted by the Author:	11-Mar-2020
Complete List of Authors:	Vinjerui, Kristin Hestmann; Norwegian University of Science and Technology Faculty of Medicine and Health Sciences, Department of Public Health and Nursing/HUNT Research Centre; Nord-Trøndelag Hospital Trust, Psychiatric Department<br>Boeckxstaens, Pauline; Ghent University, Department of Public Health and Primary Care<br>Douglas, Kirsty; Australian National University, Australian National University Medical School<br>Sund, Erik; Norwegian University of Science and Technology Faculty of Medicine and Health Sciences, Department of Public Health and Nursing/HUNT Research Centre
**Primary Subject Heading**:	Epidemiology
Secondary Subject Heading:	Public health
Keywords:	PUBLIC HEALTH, EPIDEMIOLOGY, MENTAL HEALTH

SCHOLARONE™
Manuscripts

I, the Submitting Author has the right to grant and does grant on behalf of all authors of the Work (as defined in the below author licence), an exclusive licence and/or a non-exclusive licence for contributions from authors who are: i) UK Crown employees; ii) where BMJ has agreed a CC-BY licence shall apply, and/or iii) in accordance with the terms applicable for US Federal Government officers or employees acting as part of their official duties; on a worldwide, perpetual, irrevocable, royalty-free basis to BMJ Publishing Group Ltd ("BMJ") its licensees and where the relevant Journal is co-owned by BMJ to the co-owners of the Journal, to publish the Work in this journal and any other BMJ products and to exploit all rights, as set out in our licence.

The Submitting Author accepts and understands that any supply made under these terms is made by BMJ to the Submitting Author unless you are acting as an employee on behalf of your employer or a postgraduate student of an affiliated institution which is paying any applicable article publishing charge ("APC") for Open Access articles. Where the Submitting Author wishes to make the Work available on an Open Access basis (and intends to pay the relevant APC), the terms of reuse of such Open Access shall be governed by a Creative Commons licence – details of these licences and which Creative Commons licence will apply to this Work are set out in our licence referred to above.

Other than as permitted in any relevant BMJ Author's Self Archiving Policies, I confirm this Work has not been accepted for publication elsewhere, is not being considered for publication elsewhere and does not duplicate material already published. I confirm all authors consent to publication of this Work and authorise the granting of this licence.

For peer review only

Prevalence of Multimorbidity with Frailty and Associations with Socioeconomic Position in an Adult Population: Findings from the Cross-sectional HUNT Study in Norway

Kristin H Vinjerui[a,b,c*], Pauline Boeckxstaens[d], Kirsty Douglas[b], Erik R Sund[a,e].

[a]HUNT Research centre, Department of Public Health and Nursing, Faculty of Medicine and Health Sciences, NTNU – Norwegian University of Science and Technology, Trondheim. Forskningsvegen 2, 7600 Levanger, Norway.

[b]Academic Unit of General Practice, Australian National University Medical School, Building 4, Level 2, The Canberra Hospital, Woden ACT 2606, Canberra, Australia

[c]Psychiatric Department, Levanger Hospital, Nord-Trøndelag Hospital Trust, Norway.

[d]Department of Public Health and Primary Care, Ghent University (UG), UZ Gent Corneel Heymanslaan 10 9000 Ghent, Belgium.

[e]Faculty of Nursing and Health Sciences, Nord University, Levanger, Norway

*Corresponding author: Kristin Hestmann Vinjerui, HUNT Research centre, Forskningsvegen 2, 7600 Levanger, Norway. E-mail: Kristin.vinjerui@ntnu.no.Tel.: 0047-90173201/74019240/74075180.

WORD COUNT

Abstract: 296.

Manuscript: 3619.

KEYWORDS

Multimorbidity. Frailty. Socioeconomic status. Occupations. Public health. Health inequality. The HUNT Study.

For peer review only

ABSTRACT

Objectives: To explore prevalences and occupational group inequalities of two measures of multimorbidity with frailty.

Design: Cross-sectional study.

Setting: The Nord-Trøndelag Health Study (HUNT), Norway, a total county population health survey, 2006-2008.

Participants: Participants older than 25 years, with complete questionnaires, measurements and occupation data, were included.

Outcomes: ≥2 of 51 multimorbid conditions with ≥1 of 4 frailty measures (poor health, mental illness, physical impairment or social impairment) and ≥3 of 51 multimorbid conditions with ≥2 of 4 frailty measures.

Analysis: Logistic regression models with age and occupational group, were specified for each sex separately.

Results: Of 41193 adults, 38027 (55% women; 25-100 years old) were included. 39% had ≥2 multimorbid conditions with ≥1 frailty measure, and 17% had ≥3 multimorbid conditions with ≥2 frailty measures. Prevalence differences in percentage points of those in high vs low occupational group with ≥2 multimorbid conditions and ≥1 frailty measure, were 17 (95% CI,14 to 20) in women and 5 (1 to 9) in men at 30 years; 15 (13 to 17) in both sexes at 55 years; and 3 (-3 to 9) in women and 14 (9 to 18) in men at 80 years. In those with ≥3 multimorbid conditions and ≥2 frailty measures, prevalence differences were 8 (6 to 10) in women and 2 (0 to 4) in men at 30 years; 10 (8 to 11) in women and 9 (8 to 11) in men at 55 years, and 4 (-1 to 10) in women and 6 (1 to 10) in men at 80 years.

Conclusion: Multimorbidity with frailty is common and social inequalities persist until age 80 years in women and throughout the lifespan in men. To manage complex multimorbidity, strategies for proportionate universalism in medical education, health care, public health prevention and promotion seem necessary.

ARTICLE SUMMARY

Strengths and limitations of this study

1. The HUNT Study is a large total county population general health survey with a multitude of variables, suitable to estimate prevalences of multimorbidity and frailty by self-reports and clinical measurements.
2. Occupation is used as a marker for socioeconomic position, enabling international comparison.
3. Sex-specific occupational group differences in multimorbidity with frailty are reported as both absolute and relative measures of inequality
4. As a secondary analysis, the measures in this study need to be adjusted to fit previously collected data.
5. In particular, the original data lacked information of chronicity of conditions, which may lead to overestimation of multimorbidity.

For peer review only

INTRODUCTION

Multimorbidity, the co-occurrence of multiple, chronic conditions, where none is more central,[1] is increasingly prevalent and becoming the norm.[2-4] Multimorbidity is associated with high health care utilization[5] and challenges clinicians in a fragmented health care system, aided by single disease guidelines.[6] The treatment burden to patients is often substantial including lowered ability to self-care.[6] Ways to harmonize guidelines to fit multimorbidity[7 8] and manage patients with multimorbidity in clinical practice[6] have been explored, and specific multimorbidity care guidelines are emerging.[9 10]

Multimorbidity alone may not imply a need for complex, multidisciplinary care.[1] Sociodemographic characteristics, individual health and social experiences, and mental and somatic health characteristics,[11] increase patient complexity. The British National Institute for Health and Care Excellence (NICE) guideline,[10] defines multimorbidity as two or more long-term, single-count health conditions and recommends a multimorbid approach to care in various contexts, including mixed mental and somatic multimorbidity and multimorbidity with frailty.

Frailty increases the vulnerability for adverse outcomes. It has been understood as characterized by loss of biophysical reserves in elderly,[12] operationalized as the frailty phenotype.[12] Another approach is the frailty index,[13] which calculate a ratio of accumulation of numerous deficits in several domains. An opinion of experts, further emphasize the latter multidimensional view and defines frailty as a dynamic state of multicausality, involving loss of function in spheres such as physical, psychological, and social domains.[14] This can be regarded as a biopsychosocial frailty model.[15]The NICE guideline proposes identification of frailty through observation of a low gait speed or poor self-rated health or by scoring a frailty scale combining demographic characteristics and multidimensional impairments.[10]

Social health inequalities are established; low socioeconomic position is associated with poorer health outcomes in Nordic countries[16] and globally.[17] Multimorbidity and frailty are no exception. Common determinants are socioeconomic deprivation,[18 19] female sex,[18 20] and higher age.[18 20] In descriptive studies, any indicator of socioeconomic position will detect occurring differences.[21] Socioeconomic gradients in prevalence of multimorbidity and frailty, has been explored by education,[18 19 22 23] income,[22 23] occupation,[3] and deprivation indexes.[18 19] Occupation is associated with education and income and may have an impact on health outcomes through biopsychosocial work exposures.[21] Although proportions with multimorbidity and frailty increase with higher age, more multimorbid are young and middle aged than old[4 24] and frailty is associated with multimorbidity and mortality from middle age.[25] The NICE guideline emphasizes assessment of a multimorbid approach to care for adults of all ages but does not take into account social position.

There are numerous operational definitions of both multimorbidity and frailty and prevalence vary by setting, definitions and methods.[18 26-28] The literature suggests that multimorbidity, defined as three or more single health conditions, increases specificity especially in older age groups.[26 29] Common frailty scales require multidimensional loss of function to identify frail individuals[20] and share ability to show associations to age, sex and mortality.[20]

The overall purpose of this study is to identify how many in a general adult population is likely to need complex, multidisciplinary care as given by one of the contexts suggested by the NICE guideline; multimorbidity with frailty. Two measures will be assessed, one in line

For peer review only - http://bmjopen.bmj.com/site/about/guidelines.xhtml

with the guideline (two conditions of multimorbidity plus one dimension of frailty) and the other with expected increased specificity (three conditions of multimorbidity plus two dimensions of frailty). The second aim is to examine associations of these measures according to age, sex, and socioeconomic position.

MATERIALS AND METHODS

Reporting statement

The STROBE cross sectional reporting guidelines[30] were used for reporting of this observational study.

Study design and population

This cross-sectional study use data from the third wave in the Norwegian HUNT Study (the HUNT3 Survey, 2006-2008). Details on data collection and the cohort profile of this total county population health survey was published previously.[31] In brief, 93860 residents older than 20 years were invited. 54% (n=50807 of 93860) completed the main questionnaire, meeting the minimum requirement for HUNT3 Survey attendance.[31] Figure 1 presents the sample selection for this analysis.

81% (41193 of 50807) eligible participants completed all major parts of the HUNT3 Survey; the main, age- and sex-specific questionnaires; interviews; and measurements. Incomplete participation excluded 9610 individuals, while four missed complete information on participation. 1569 respondents were younger than 25 years and were excluded on the assumption that the highest level of occupational group may not yet be obtained by those in this age category. One missed information on age. 1571 individuals missed information on occupation, while 25 people had “unspecified occupation” and was excluded. 38027 of 41193 (92%) participants were included in the final sample.

Overall, lower socioeconomic position was associated with lower participation rate in the HUNT3 Survey.[32] In this study, the distribution of occupational groups was 24% (high), 27% (middle) and 49% (low) in the sample and 17% (high), 20% (middle), 52% (low) and 11% (missing) among non-eligible. 100% of the missing were due to missing classifiable occupational data. Women constituted 55%, 51% and 81%, of the sample, non-eligible and missing, respectively. The mean (standard deviation) age was 55 (14) years in the sample, 44 (18) years among non-eligible and 66 (18) years among those missing data.

Demographic and Sociodemographic Characteristics

Sex and age at participation in the HUNT3 Survey was constructed by the HUNT Databank. Occupational group was used as indicator of socioeconomic position.[21] In the HUNT3 Survey interview, all participants were asked, “What is/was the title of your main occupation?” Free-text answers were manually categorized corresponding to Standard Classifications of Occupations by Statistics Norway,[33] which is based on the International Standard Classification of Occupations-88.[34] Occupational socioeconomic position was operationalized using occupation only, corresponding to a simplified version of the European Socio-economic Classification scheme.[35] The scheme aims to differentiate occupational groups on employment relationships and is not hierarchical per se. Still, the higher occupational groups are likely to have higher and more secure income.[35] Collapsed to a 3-class version, the high level represents large employers, higher-grade and lower-grade professionals, administrative and managerial occupations, and higher-grade technician and

For peer review only

supervisory occupations. The middle group consist of small employers, self-employed individuals, and lower-grade supervisory and technician occupations. The low level contains lower-grade service positions, sales and clerical occupations, and lower-grade technical and routine occupations. Details are provided in appendix A.

Outcomes

Multimorbidity

The construction of 51 single, chronic conditions from the HUNT3 Survey data, is described in appendix B. Table 1 lists the 51 conditions by 14 ICD-10 chapters, a disease classification system in major organized by organ systems. In this study, a simple, non-weighted summary score was generated and two multimorbidity variables created, with cutoff values of at least 2 of 51 and 3 of 51 conditions.

For peer review only

Table 1. Conditions grouped by ICD-10 chapter.

**ICD-10 chapter** Conditions		**ICD-10 chapter** Conditions	
II	**Neoplasms**	X	**Respiratory system**
	Cancer		Chronic bronchitis, emphysema or COPD[1,2]
III	**Blood/blood-forming organs/**		Asthma
	**immune mechanism**	XI	**Digestive system**
	Sarcoidosis		Dental health status
IV	**Endocrine/nutritional/metabolic**		Gastro-oesophageal reflux disease
	Obesity		Irritable bowel syndrome
	Hypercholesterolemia	XII	**Skin/subcutaneous tissue**
	Diabetes		Hand eczema
	Hypothyroidism		Psoriasis
	Hyperthyroidism	XIII	**Musculoskeletal/connective tissue**
V	**Mental/behavioural**		Rheumatoid arthritis
	Alcohol problem		Osteoarthritis
	Depression		Ankylosing spondylitis
	Anxiety		Fibromyalgia
	Insomnia		Osteoporosis
VI	**Nervous system**		Local musculoskeletal pain/stiffness in:
	Epilepsy		- Neck
	Migraine		- Upper back
	Chronic headache, other		- Lower back
VII	**Eye/adnexa**		- Shoulder
	Cataract		- Elbow
	Macula degeneration		- Hand
	Glaucoma		- Hip
VIII	**Ear/mastoid**		- Knee
	Hearing impairment		- Foot/ancle
IX	**Circulatory system**	XIV	**Genitourinary system**
	Hypertension		Kidney disease
	Angina pectoris		Urine incontinence
	Myocardial infarction		Prostate symptoms
	Heart failure		Menopausal hot flashes
	Other heart disease[1]	XVIII	**Symptoms/signs/abnormal clinical/**
	Stroke or brain haemorrhage[1]		**laboratory findings**
			Nocturia
			Chronic widespread pain

[1] = Exception to single entity.

[2]COPD = Chronic Obstructive Pulmonary Disease.

Frailty

Original data did not match any exact frailty scale. A qualitative judgement of available data was undertaken and general, mental, physical and social dimensions[10 14 20] of frailty were operationalized from six original variables:

For peer review only

1. General health status, defined as those reporting the answers “poor” or “not so good” (vs “good” and “very good”) to the single question “How is your health at the moment?”
2. Mental health status, included those reporting symptoms of anxiety and/or depression, on the Hospital Anxiety and Depression Scale. The HUNT Databank calculated a total score for subscales of anxiety and depression, if all items for anxiety and depression, respectively, were answered. In this study, cutoff was set at 8/21 points for both conditions[36] and a combined variable was created.
3. Physical impairment was identified by combining those reporting “yes” (vs “no”) in response to the question, “Do you suffer from any long-term (at least 1 year) illness or injury of a physical or psychological nature that impairs your functioning in your daily life?” and reporting either motor ability, vision, or hearing impairment to a moderate or severe degree.
4. Social impairment was derived from answers to the single question, “To what extent has your physical health or emotional problems limited you in your usual socializing with family or friends during the last 4 weeks?” Included were those reporting “much” and “not able to socialize” (vs “not at all,” “very little,” or “somewhat”).

A summary score was generated and two frailty variables created, with cutoff values of at least 1 of 4 and 2 of 4 frailty measures with impairment.

Multimorbidity with frailty

The two final outcome variables, were created by combining self-reported multimorbidity and frailty as at least 2 of 51 chronic health conditions plus impairment in 1 of 4 dimensions of frailty and 3 of 51 chronic health conditions plus impairments in 2 of 4 dimensions of frailty.

Statistical analysis

We used cross-tables to identify sociodemographic characteristics by occupational group (table 2) and by multimorbidity with frailty, stratified by sex (table 3).

Associations between occupational group and the two measures of multimorbidity with frailty were analyzed using logistic regression, adjusted for age and sex. All models were stratified by sex and included occupational group, continuous age, age squared, and an interaction term between occupational group and age. Likelihood ratio tests were used to compare models.

Given the high prevalence of multimorbidity with frailty and the knowledge that odds ratios will deviate from relative risks,[37] we used postestimation commands to obtain prevalence differences and prevalence ratios[38] between the occupational groups with high occupational group as the reference category. The prevalence difference is the difference in mean predicted probability, and prevalence ratio is the ratio between the mean predicted probabilities while holding other covariates constant.[38] Prevalence difference and prevalence ratio between occupational groups were calculated at age 25 to 100 years in 5-year intervals (appendix C). Calculations (with 95% confidence intervals) are presented at the ages 30, 55 and 80 to reflect young adults, middle aged and elderly (table 4).

We performed complete case analysis and used Stata version 15.1 (StataCorp. College Station, TX, USA) to analyze the data.

For peer review only

Patient and public involvement

During the preparation of the HUNT3 Survey, there was a wide citizen and stakeholder participation. This study is a secondary analysis of data collected in 2006-2008. Multimorbidity is a universal topic, not represented by any particular patient group, thus no patient or public representative were involved in designing the study.

RESULTS

38027 individuals, older than 25 years, who had completed all major parts of the HUNT3 Survey and had data on occupation, comprised the final sample for this study (fig. 1). Further sociodemographic characteristics is presented in table 2.

Table 2. Sex and age distribution by occupational group.

	**Occupational group**							
	**High**		**Middle**		**Low**		**Total**	
	**Frequency**	**(%)**	**Frequency**	**(%)**	**Frequency**	**(%)**	**Frequency**	**(%)**
**Total**	8 970	(100)	10 243	(100)	18 814	(100)	38 027	(100)
**Sex**								
Female	4 505	(50)	5 386	(53)	10 922	(58)	20 813	(55)
Male	4 465	(50)	4 857	(47)	7 892	(42)	17 214	(45)
**Age, years**								
25-44	2 837	(32)	2 600	(25)	4 487	(24)	9 924	(26)
45-64	4 468	(50)	4 787	(47)	8 951	(48)	18 206	(48)
65-74	1 118	(12)	1 846	(18)	3 297	(18)	6 261	(16)
75-100	547	(6)	1 010	(10)	2 079	(11)	3 636	(10)

Most participants, 49% (n=18814 of 38027), are categorized as low occupational group, which is comprised of 58% (n=10922 of 18814) women, while women constitute 55% (n=20813 of 38027) of the total sample.

For peer review only

Table 3. Frequency distribution of two definitions of multimorbidity with frailty across occupational groups and age categories, stratified by sex.

		Women						Men					
		Two conditions of multimorbidity and one dimension of frailty*						Two conditions of multimorbidity and one dimension of frailty*					
		No, freq.	(%)	Yes, freq.	(%)	Total, freq.	(%)	No, freq.	(%)	Yes, freq.	(%)	Total, freq.	(%)
**Total**		12 304	(59)	8 482	(41)	20 813	(100)	10 826	(63)	6 378	(37)	17 214	(100)
**Occupational group**													
	High	3 222	(72)	1 282	(28)	4 505	(100)	3 220	(72)	1 242	(28)	4 465	(100)
	Middle	3 370	(63)	2 009	(37)	5 386	(100)	2 995	(62)	1 860	(38)	4 857	(100)
	Low	5 712	(52)	5 191	(48)	10 922	(100)	4 611	(58)	3 276	(42)	7 892	(100)
**Age, years**													
	25-44	4 298	(72)	1 680	(28)	5 981	(100)	3 075	(78)	867	(22)	3 943	(100)
	45-64	5 712	(58)	4 122	(42)	9 840	(100)	5 398	(65)	2 967	(35)	8 366	(100)
	65-74	1 615	(51)	1 548	(49)	3 168	(100)	1 681	(54)	1 409	(46)	3 093	(100)
	75-100	679	(37)	1 132	(62)	1 824	(100)	672	(37)	1 135	(63)	1 812	(100)
Mean (SD)		52	(14)	58	(14)	54	(14)	54	(14)	61	(14)	56	(14)
		**Three conditions of multimorbidity and two dimensions of frailty***						**Three conditions of multimorbidity and two dimensions of frailty***					
		No, freq.	(%)	Yes, freq.	(%)	Total, freq.	(%)	No, freq.	(%)	Yes, freq.	(%)	Total, freq.	(%)
**Total**		16 983	(82)	3 803	(18)	20 813	(100)	14 367	(83)	2 837	(16)	17 214	(100)
**Occupational group**													
	High	4 029	(89)	475	(11)	4 505	(100)	3 977	(89)	485	(11)	4 465	(100)
	Middle	4 491	(83)	888	(16)	5 386	(100)	3 995	(82)	860	(18)	4 857	(100)
	Low	8 463	(77)	2 440	(22)	10 922	(100)	6 395	(81)	1 492	(19)	7 892	(100)
**Age, years**													
	25-44	5 378	(90)	600	(10)	5 981	(100)	3 651	(93)	291	(7)	3 943	(100)
	45-64	7 920	(80)	1 914	(19)	9 840	(100)	7 024	(84)	1 341	(16)	8 366	(100)
	65-74	2 449	(77)	714	(23)	3 168	(100)	2 472	(80)	618	(20)	3 093	(100)
	75-100	1 236	(68)	575	(32)	1 824	(100)	1 220	(67)	587	(32)	1 812	(100)
Mean (SD)		53	(14)	60	(14)	54	(14)	55	(14)	63	(13)	56	(14)

Abbreviations: freq., frequency; SD, standard deviation

*In total, 27 women and 10 men miss data on both measures of multimorbidity with frailty.

For peer review only

In total, 77% reported more than two and 62% more than three conditions of multimorbidity. Frailty with one impairment was identified in 41% and with two impairments in 18%. Table 3 shows the distribution of the combined measures across occupational groups stratified by sex.

Overall, 39% met the criteria of having at least two conditions of multimorbidity with one dimension of frailty (41% [n=8482 of 20813] of women, 37% [n=6378 of 17214] of men) and 17% met the criteria of three-condition multimorbidity with two dimensions of frailty (18% [n=3803 of 20813] of women, 16% [n=2837 of 17214] of men).

Proportions of multimorbidity with frailty increased with lower occupational rank and increasing age, in both sexes, regardless of definition. Most individuals with any definition of multimorbidity with frailty, were younger than 64 years.

For peer review only

Table 4. Prevalence ratios (PR) and prevalence differences (PD) with 95% confidence intervals (CI) between occupational groups and multimorbidity with frailty, stratified by sex.

		Women				Men			
Age,	Occupational	Two conditions of multimorbidity and one dimension of frailty							
years	group	PR	(95% CI)	PD	(95% CI)	PR	(95% CI)	PD	(95% CI)
30	High	1.00	(Ref.)	0.00	(Ref.)	1.00	(Ref.)	0.00	(Ref.)
	Middle	1.36	(1.11, 1.65)	0.06	(0.02, 0.09)	0.93	(0.70, 1.23)	-0.01	(-0.06, 0.03)
	Low	2.09	(1.76, 2.47)	0.17	(0.14, 0.20)	1.32	(1.04, 1.67)	0.05	(0.01, 0.09)
55	High	1.00	(Ref.)	0.00	(Ref.)	1.00	(Ref.)	0.00	(Ref.)
	Middle	1.22	(1.13, 1.31)	0.07	(0.04, 0.09)	1.34	(1.23, 1.45)	0.08	(0.06, 0.11)
	Low	1.48	(1.38, 1.58)	0.15	(0.13, 0.17)	1.60	(1.48, 1.72)	0.15	(0.13, 0.17)
80	High	1.00	(Ref.)	0.00	(Ref.)	1.00	(Ref.)	0.00	(Ref.)
	Middle	0.96	(0.86, 1.08)	-0.02	(-0.09, 0.05)	1.23	(1.12, 1.35)	0.12	(0.06, 0.17)
	Low	1.05	(0.95, 1.16)	0.03	(-0.03, 0.09)	1.27	(1.15, 1.39)	0.14	(0.09, 0.18)
Age,	Occupational	Three conditions of multimorbidity and two dimensions of frailty							
years	group	PR	(95% CI)	PD	(95% CI)	PR	(95% CI)	PD	(95% CI)
30	High	1.00	(Ref.)	0.00	(Ref.)	1.00	(Ref.)	0.00	(Ref.)
	Middle	2.31	(1.56, 3.40)	0.04	(0.02, 0.06)	1.29	(0.77, 2.17)	0.01	(-0.01, 0.03)
	Low	3.59	(2.53, 5.08)	0.08	(0.06, 0.10)	1.60	(1.02, 2.51)	0.02	(0.00, 0.04)
55	High	1.00		0.00		1.00		0.00	
	Middle	1.31	(1.14, 1.50)	0.04	(0.02, 0.06)	1.62	(1.40, 1.87)	0.06	(0.04, 0.07)
	Low	1.78	(1.59, 2.00)	0.10	(0.08, 0.11)	2.05	(1.80, 2.33)	0.09	(0.08, 0.11)
80	High	1.00	(Ref.)	0.00	(Ref.)	1.00	(Ref.)	0.00	(Ref.)
	Middle	1.17	(0.94, 1.47)	0.05	(-0.02, 0.11)	1.26	(1.06, 1.50)	0.07	(0.02, 0.11)
	Low	1.16	(0.94, 1.42)	0.04	(-0.01, 0.10)	1.22	(1.04, 1.44)	0.06	(0.01, 0.10)

Table 4 shows prevalence differences and prevalence ratios for each definition of multimorbidity with frailty between occupational groups for women and men at the ages 30, 55, and 80 years.

Prevalence differences in percentage points (pp) for two-condition multimorbidity with one dimension of frailty between high and low occupational groups were largest in women at 30 years, 17 (14 to 20) pp and 55 years, 15 (13 to 17) pp, and for men at 55 years, 15 (13 to 17) pp and 80 years 14 (9 to 18) pp. The prevalence ratio for the low occupational group compared with the high occupational group, for two-condition multimorbidity with one dimension of frailty, was greatest in women at 30 years, 2.09 (1.76 to 2.47) and in men at 55 years, 1.60 (1.48 to 1.72). The prevalence ratio decreased in both sexes in high age and was at 80 years 1.05 (0.95 to 1.16) for women and 1.27 (1.15 to 1.39) for men.

Correspondingly, prevalence differences in percentage points between high and low occupational groups for three-condition multimorbidity with two dimensions of frailty, were largest in women at 30 years, 8 (CI: 6 to 10) pp and 55 years, 10 (CI: 8 to 11) pp and in men at 55 years 9 (CI: 8 to 11) pp and 80 years 6 (CI: 1 to 10) pp. Prevalence ratio, comparing the low

For peer review only

occupational group with the highest occupational group for three-conditions multimorbidity with two conditions of frailty, was greatest in women at 30 years, 3.59 (1.43 to 5.08) and in men at 55 years 2.05 (1.80 to 2.33). The prevalence ratio decreased in both sexes in high age and was at 80 years 1.16 (0.94 to 1.42) for women and 1.22 (1.04 to 1.44) for men.

DISCUSSION

Main results

In this adult population health study, multimorbidity with frailty was common as 39% met the criteria of two-condition multimorbidity plus one dimension of frailty and 17% met the criteria of three-condition multimorbidity plus two dimensions of frailty. Proportions increased with lower occupational group, higher age and female sex from 25 to 74 years, but was common across age groups in both sexes. Occupational inequalities were consistent in both sexes until high age, diminishing in women, while still present in men at age 80 years.

Comparison with existing literature

Investigating two measures of multimorbidity with frailty in one sample offers a unique direct comparison of occurrences and socioeconomic gradients. Lower overall prevalence for the stricter measure three-condition multimorbidity with two dimensions of frailty, is expected. Defining multimorbidity by three or more conditions differentiates into older age.[26 29] The joint measure multimorbidity and frailty, show the same tendency, as 62% of 75- to 100-year-olds met the criteria of at least two-condition multimorbidity with one dimension of frailty, while 32% reported three-condition multimorbidity with two dimensions of frailty. In line with individual studies on multimorbidity[4 24] and frailty,[25] most individuals with co-present multimorbidity and frailty are younger than 64 years.

A recent commentary[1] emphasized exploring multimorbidity guidelines and frailty as part of multimorbidity's complexity and overlap of multimorbidity and frailty has newly been reviewed.[28] A pooled prevalence of 16% (95% CI 12-21%) was reported for two conditions multimorbidity with the frailty phenotype among elderly,[28] while 39% in our study reported at least two conditions of multimorbidity with one dimension of frailty. The prevalence differences are likely explained by differences in methods. The articles included in the review studied age 60 years and older. Still, the prevalence of multimorbidity are low. All but one defined multimorbidity from lists of less than 12 conditions and prevalences are probably underestimated.[26 29] Frailty too was only operationalized with the biophysical model, while more people are expected to be detected using a multidimensional measure.

We have not identified studies on prevalence and social determinants of multimorbidity with frailty. Low social position,[18 19] older age,[18 20] and female sex[18 20] are known common determinants of multimorbidity and frailty. We therefore argue that the direction of the sociodemographic determinants in this study are as expected. The magnitudes of these gradients, however, have not been comparable with other studies.

Mechanisms to explain findings

The aggregation of ill health, multimorbidity and frailty included, in lower socioeconomic positions is explained by numerous theories. Overall, unequal distribution of power, income and

resources, result in fundamental different conditions of daily life yielding inequalities in health.[17] With regards to occupation, several mechanisms can explain associations to health outcomes. The higher occupational group is expected to have higher, more stable income,[35 39] more beneficial social networks,[39] and more autonomy and control[35 39] at work. Adverse working conditions such as exposure to toxic work environments[21] or demanding physical requirements[39] tend to cluster in lower occupational groups.[17] Persisting health inequalities in assumed egalitarian Nordic countries, is partly understood as mortality selection, where, given the well-developed health care and welfare systems, frail individuals survive, but likely end up in a low social position.[16] Further, smoking, overall morbidity and mortality decreases at a higher rate among higher than lower social groups.[16] In this study, the demographic age distribution explain the high number of 45- to 64-years old with co-present multimorbidity and frailty. Additionally, incidence of new conditions, is associated with count of conditions at baseline,[4] as well as age,[4] thus individuals in lower occupational groups may aggregate conditions faster. The bidirectional association of health and occupation, may explain higher occupational group prevalence ratios in younger individuals,[21] while lower ratios by increasing age are expected, since multimorbidity with frailty is more common[40] with advancing age. Finally, survival bias justifies diminishing occupational differences at age 80 years.

Strengths and limitations

Materials and methods meet the standards of studies on multimorbidity, frailty, and social health inequalities, strengthening this study. In multimorbidity studies, population-based health surveys are the most frequent study design,[41] and prevalence estimates from self-reports are justified when studying large samples.[26] Deriving the condition count multimorbidity measures from a complete list of single-entity conditions, is shown to yield proper prevalence estimates.[29] A multidimensional frailty measure agrees with an holistic, unrestricted on age, conceptual definition of frailty[14] and with common frailty scales, which share ability to show associations to age, sex and mortality.[20] In descriptive studies, any measure of socioeconomic position will reveal health inequalities, if such exists.[21] Occupation is an established marker for socioeconomic position,[21] in which this study had individual data classified to facilitate international comparison. Finally, socioeconomic differences are explored as both absolute and relative measures[16] and presented by sex.[18]

There are always limitations in secondary analysis of data collected a priori and not for the purpose of the current study. Measures of multimorbidity and frailty are also manifold, and operationalizations were adjusted to fit the available data. This challenges the external validity and comparability between studies, however, is sought reduced through transparency of morbidities included and construction of variables. A majority of included multimorbidity conditions do not contain information regarding duration. Thus, reported prevalence of multimorbidity may be overestimated and not represent true chronicity. It is recognized that frailty scales may differ in accuracy of detecting frailty in younger age groups,[10 20] however, frailty symptoms are of great clinical value regardless of age.[10 42] The accuracy of the frailty variables were not explored and frailty was measured solely as self-report, an approach that may underestimate overall prevalence[43] and overestimate proportion among women compared to men.[43]

For peer review only

Lastly, in the HUNT3 Survey participants were asked for their "main" occupation, which is not necessarily the current or longest lasting occupation, more commonly studied.[39] Younger than middle-aged may to some extent be misclassified in the lower occupational group, which will underestimate social differences in health among younger subjects. Occupational data may obscure current social context,[39] and underestimate socioeconomic inequalities. Thus, the study would have benefitted from exploring socioeconomic position with several indicators,[44] such as individual education and income or a household measure.

Attendance in the HUNT3 Survey varied by age, sex, and social position,[32] still, the HUNT study is considered representative for Norway as a whole[45] and the cohort follows trends in health development in western high-income countries.[46-48] Depression hindered participation,[32] which may yield underestimation of both multimorbidity and frailty. An overall bias towards healthy elders is probable, since eligibility depended on attendance at a screening station.

Implications for clinical practice and policy makers

This study aimed to quantify the total prevalence of adults in the general population who might need complex, multidisciplinary care assessed as the joint measure multimorbidity with frailty. In a clinical context, the definition of at least three-condition multimorbidity with two dimensions of frailty to detect individuals for whom to initiate a multimorbid approach to care, seems more feasible. Despite acknowledgement of the association of multimorbidity and frailty with age, sex, and socioeconomic position, guidelines and interventions have yet to take this into account in assessment and management for multimorbidity.[49] Based on literature and reproduction of social gradients in our study, we suggest that clinicians consider evaluation of multimorbidity and frailty in younger age groups with social context in mind. Further research on implementation of the multimorbid approach to care model and mortality is needed before recommending changing inclusion criteria in a guideline. Since multimorbidity is becoming the norm, the organization of health care should reform to fit person-centred, coordinated, multidisciplinary care.[6 10 50]To prevent cases of multimorbidity and frailty and minimize social discrepancies, both universal and targeted life cycle approaches seem necessary.[51]
Frailty is independently associated with mortality, adjusted for multimorbidity,[25] and is reversible.[52] Thus detection of frailty is relevant for both public health and clinical purposes.

Future research

Some forms of biases are possible for both multimorbidity, frailty and social position, and a careful interpretation of findings is warranted. However, multimorbidity with frailty is common in this general population and with occupational inequalities throughout adulthood, even with stricter definitions. This adds knowledge to the public health literature about the sociodemographic distribution of multimorbidity with frailty in younger age groups, as well as very old individuals. On this background, we recommend exploring the sociodemographic distribution of alternative measures on multimorbidity, including patterns, aiming to detect individuals suspected in high need of complex, multidisciplinary health care. Furthermore, such measurements can be compared as prognostic factors for health care utilization and mortality.

For peer review only

CONCLUSION

Multimorbidity with frailty are common from young adulthood onward, with consistent socioeconomic inequalities until 80 years old. Prevention will require a proportionate universal approach on social determinants of health throughout the entire life span. The crucial need for person-centered multimorbid approach to care that acknowledges social context, demands reforms in health care organizational structure, medical education, and treatment. Further research on competing measures of high-need multimorbidity and the association of these factors with health care utilization and mortality should be explored by socioeconomic position, age and sex.

FIGURES

Figure 1: Flowchart for sample selection: inclusion and exclusion criteria and missing data.

ACKNOWLEDGEMENTS

L Getz, J Sigurdsson and C Harrison for through discussions on measures of multimorbidity.
E Solheim for guidance in the use of the European Socio-economic Classification scheme.
MS Newman for writing assistance
The Nord-Trøndelag Health Study (The HUNT Study) is a collaboration between HUNT Research Centre (Faculty of Medicine and Health Sciences, Norwegian University of Science and Technology (NTNU)), Nord-Trøndelag County Council, Central Norway Regional Health Authority, and the Norwegian Institute of Public Health.

COMPETING INTERESTS

None declared.

FUNDING STATEMENT

Funding source: This study was funded by the Faculty of Medicine and Health Science at the Norwegian University of Science and Technology (NTNU) through the PhD program in Behavior and Health (KHV). NTNU has partly funded the HUNT3 Survey and have funded open access for this article. The Liaison Committee for Education, Research and Innovation in Central Norway (17/38297) supported a research stay at the Australian National University, Canberra. The funding sources have had no role in conceptualization this study, its design and methods, analysis and interpretation of data, writing of the article or the decision to submit the article for publication.

AUTHOR CONTRIBUTIONS

KHV, ERS and KD conceptualized the study and all authors contributed to its design. KHV has analysed the data under supervision of ERS and all authors have contributed to interpreting the data. KHV wrote the original draft, which has been revised critically by ERS, KD and PB. All authors have read and approved the final version of the manuscript to be published and agree to be accountable for all aspects of the work in ensuring that questions related to the accuracy or integrity of any part of the work are appropriately investigated and resolved.

For peer review only

LICENSE STATEMENT

I, the Submitting Author has the right to grant and does grant on behalf of all authors of the Work (as defined in the below author licence), an exclusive licence and/or a non-exclusive licence for contributions from authors who are: i) UK Crown employees; ii) where BMJ has agreed a CC-BY licence shall apply, and/or iii) in accordance with the terms applicable for US Federal Government officers or employees acting as part of their official duties; on a worldwide, perpetual, irrevocable, royalty-free basis to BMJ Publishing Group Ltd ("BMJ") its licensees and where the relevant Journal is co-owned by BMJ to the co-owners of the Journal, to publish the Work in BMJ Open and any other BMJ products and to exploit all rights, as set out in our licence.

The Submitting Author accepts and understands that any supply made under these terms is made by BMJ to the Submitting Author unless you are acting as an employee on behalf of your employer or a postgraduate student of an affiliated institution which is paying any applicable article publishing charge ("APC") for Open Access articles. Where the Submitting Author wishes to make the Work available on an Open Access basis (and intends to pay the relevant APC), the terms of reuse of such Open Access shall be governed by a Creative Commons licence – details of these licences and which Creative Commons licence will apply to this Work are set out in our licence referred to above.

PATIENT CONSENT

Participation in all parts of the HUNT3 Survey was voluntary, and written informed consent was obtained from all participants.

ETHICS APPROVAL

The Regional Committee for Medical and Health Research Ethics in Norway approved the current study (project no. 2014/2265).

DATA SHARING STATEMENT

To protect participants' privacy, HUNT Research Centre aims to limit storage of data outside HUNT databank and cannot deposit data in open repositories. HUNT databank has precise information on all data exported to different projects and are able to reproduce these on request. There are no restrictions regarding data export given approval of applications to HUNT Research Centre. For more information see: http://www.ntnu.edu/hunt/data

SUPPLEMENTARY FILES

Appendix A: Operationalizing socioeconomic position.

Appendix B: Construction of chronic, single-entities conditions from data in the HUNT3 Survey, by questionnaires and measurements.

Appendix C: Table C1. Prevalence ratios (PR) and prevalence differences (PD) with 95% confidence intervals (CI) for the association between occupational group and multimorbidity with frailty, stratified by sex, age 25 to 100 years in 5-year intervals.

For peer review only

REFERENCES

1. Nicholson K, Makovski TT, Griffith LE, et al. Multimorbidity and comorbidity revisited: refining the concepts for international health research. *J Clin Epidemiol* 2019;105:142-46. doi: 10.1016/j.jclinepi.2018.09.008 [published Online First: 2018/09/27]
2. van Oostrom SH, Gijsen R, Stirbu I, et al. Time Trends in Prevalence of Chronic Diseases and Multimorbidity Not Only due to Aging: Data from General Practices and Health Surveys. *PLoS One* 2016;11(8):e0160264. doi: 10.1371/journal.pone.0160264 [published Online First: 2016/08/03]
3. Uijen AA, van de Lisdonk EH. Multimorbidity in primary care: prevalence and trend over the last 20 years. *Eur J Gen Pract* 2008;14 Suppl 1(sup1):28-32. doi: 10.1080/13814780802436093 [published Online First: 2008/10/31]
4. van den Akker M, Buntinx F, Metsemakers JF, et al. Multimorbidity in general practice: prevalence, incidence, and determinants of co-occurring chronic and recurrent diseases. *J Clin Epidemiol* 1998;51(5):367-75. doi: 10.1016/s0895-4356(97)00306-5 [published Online First: 1998/06/10]
5. Glynn LG, Valderas JM, Healy P, et al. The prevalence of multimorbidity in primary care and its effect on health care utilization and cost. *Fam Pract* 2011;28(5):516-23. doi: 10.1093/fampra/cmr013 [published Online First: 2011/03/26]
6. Wallace E, Salisbury C, Guthrie B, et al. Managing patients with multimorbidity in primary care. *BMJ* 2015;350:h176. doi: 10.1136/bmj.h176 [published Online First: 2015/02/04]
7. Guthrie B, Payne K, Alderson P, et al. Adapting clinical guidelines to take account of multimorbidity. *BMJ* 2012;345:e6341. doi: 10.1136/bmj.e6341 [published Online First: 2012/10/06]
8. Muth C, Kirchner H, van den Akker M, et al. Current guidelines poorly address multimorbidity: pilot of the interaction matrix method. *J Clin Epidemiol* 2014;67(11):1242-50. doi: 10.1016/j.jclinepi.2014.07.004 [published Online First: 2014/09/14]
9. Palmer K, Marengoni A, Forjaz MJ, et al. Multimorbidity care model: Recommendations from the consensus meeting of the Joint Action on Chronic Diseases and Promoting Healthy Ageing across the Life Cycle (JA-CHRODIS). *Health Policy* 2018;122(1):4-11. doi: 10.1016/j.healthpol.2017.09.006 [published Online First: 2017/10/03]
10. National Institute for Health and Care Excellence. Multimorbidity: clinical assessment and management. London: National Institute for Health and Care Excellence (UK). 2016.
11. Schaink AK, Kuluski K, Lyons RF, et al. A scoping review and thematic classification of patient complexity: offering a unifying framework. *Journal of comorbidity* 2012;2:1-9. doi: 10.15256/joc.2012.2.15 [published Online First: 2012/10/10]
12. Fried LP, Tangen CM, Walston J, et al. Frailty in older adults: evidence for a phenotype. *J Gerontol A Biol Sci Med Sci* 2001;56(3):M146-56. doi: 10.1093/gerona/56.3.m146 [published Online First: 2001/03/17]
13. Mitnitski AB, Mogilner AJ, Rockwood K. Accumulation of deficits as a proxy measure of aging. *ScientificWorldJournal* 2001;1:323-36. doi: 10.1100/tsw.2001.58 [published Online First: 2003/06/14]
14. Gobbens RJ, Luijkx KG, Wijnen-Sponselee MT, et al. In search of an integral conceptual definition of frailty: opinions of experts. *J Am Med Dir Assoc* 2010;11(5):338-43. doi: 10.1016/j.jamda.2009.09.015 [published Online First: 2010/06/01]
15. Solfrizzi V, Scafato E, Lozupone M, et al. Biopsychosocial frailty and the risk of incident dementia: The Italian longitudinal study on aging. *Alzheimers Dement* 2019;15(8):1019-28. doi: 10.1016/j.jalz.2019.04.013 [published Online First: 2019/07/07]
16. Huijts T, Eikemo TA. Causality, social selectivity or artefacts? Why socioeconomic inequalities in health are not smallest in the Nordic countries. *Eur J Public Health* 2009;19(5):452-3. doi: 10.1093/eurpub/ckp103 [published Online First: 2009/07/10]
17. Commission on Social Determinants of Health. Closing the gap in a generation: health equity through action on the social determinants of health: final report of the commission on social determinants of health. Geneva2008:9.
18. Violan C, Foguet-Boreu Q, Flores-Mateo G, et al. Prevalence, determinants and patterns of multimorbidity in primary care: a systematic review of observational studies. *PLoS One* 2014;9(7):e102149. doi: 10.1371/journal.pone.0102149 [published Online First: 2014/07/23]

For peer review only

19. Franse CB, van Grieken A, Qin L, et al. Socioeconomic inequalities in frailty and frailty components among community-dwelling older citizens. *PLoS One* 2017;12(11):e0187946. doi: 10.1371/journal.pone.0187946 [published Online First: 2017/11/10]
20. Theou O, Brothers TD, Pena FG, et al. Identifying common characteristics of frailty across seven scales. *J Am Geriatr Soc* 2014;62(5):901-6. doi: 10.1111/jgs.12773 [published Online First: 2014/04/05]
21. Galobardes B, Lynch J, Smith GD. Measuring socioeconomic position in health research. *Br Med Bull* 2007;81-82(1):21-37. doi: 10.1093/bmb/ldm001 [published Online First: 2007/02/08]
22. Agborsangaya CB, Lau D, Lahtinen M, et al. Multimorbidity prevalence and patterns across socioeconomic determinants: a cross-sectional survey. *BMC Public Health* 2012;12:201. doi: 10.1186/1471-2458-12-201 [published Online First: 2012/03/21]
23. Szanton SL, Seplaki CL, Thorpe RJ, Jr., et al. Socioeconomic status is associated with frailty: the Women's Health and Aging Studies. *J Epidemiol Community Health* 2010;64(1):63-7. doi: 10.1136/jech.2008.078428 [published Online First: 2009/08/21]
24. Barnett K, Mercer SW, Norbury M, et al. Epidemiology of multimorbidity and implications for health care, research, and medical education: a cross-sectional study. *Lancet* 2012;380(9836):37-43. doi: 10.1016/S0140-6736(12)60240-2 [published Online First: 2012/05/15]
25. Hanlon P, Nicholl BI, Jani BD, et al. Frailty and pre-frailty in middle-aged and older adults and its association with multimorbidity and mortality: a prospective analysis of 493 737 UK Biobank participants. *The Lancet Public health* 2018;3(7):e323-e32. doi: 10.1016/S2468-2667(18)30091-4 [published Online First: 2018/06/18]
26. Fortin M, Stewart M, Poitras ME, et al. A systematic review of prevalence studies on multimorbidity: toward a more uniform methodology. *Ann Fam Med* 2012;10(2):142-51. doi: 10.1370/afm.1337 [published Online First: 2012/03/14]
27. O'Caoimh R, Galluzzo L, Rodriguez-Laso A, et al. Prevalence of frailty at population level in European ADVANTAGE Joint Action Member States: a systematic review and meta-analysis. *Ann Ist Super Sanita* 2018;54(3):226-38. doi: 10.4415/ANN_18_03_10 [published Online First: 2018/10/05]
28. Vetrano DL, Palmer K, Marengoni A, et al. Frailty and Multimorbidity: A Systematic Review and Meta-analysis. *J Gerontol A Biol Sci Med Sci* 2019;74(5):659-66. doi: 10.1093/gerona/gly110 [published Online First: 2018/05/05]
29. Harrison C, Britt H, Miller G, et al. Examining different measures of multimorbidity, using a large prospective cross-sectional study in Australian general practice. *BMJ Open* 2014;4(7):e004694. doi: 10.1136/bmjopen-2013-004694 [published Online First: 2014/07/13]
30. von Elm E, Altman DG, Egger M, et al. The Strengthening the Reporting of Observational Studies in Epidemiology (STROBE) Statement: guidelines for reporting observational studies. *Int J Surg* 2014;12(12):1495-9. doi: 10.1016/j.ijsu.2014.07.013 [published Online First: 2014/07/22]
31. Krokstad S, Langhammer A, Hveem K, et al. Cohort Profile: the HUNT Study, Norway. *Int J Epidemiol* 2013;42(4):968-77. doi: 10.1093/ije/dys095 [published Online First: 2012/08/11]
32. Langhammer A, Krokstad S, Romundstad P, et al. The HUNT study: participation is associated with survival and depends on socioeconomic status, diseases and symptoms. *BMC Med Res Methodol* 2012;12:143. doi: 10.1186/1471-2288-12-143 [published Online First: 2012/09/18]
33. Statistics Norway. Standard Classification of Occupations. Oslo/Kongsvinger: Statistics Norway 1998.
34. International Labour Organization (ILO). The International Standard Classification of Occupations, ISCO-88 [Webpage]. 1988 [updated 18.09.2004. Available from: https://www.ilo.org/public/english/bureau/stat/isco/isco88/index.htm accessed 24.05. 2019.
35. Rose D, Harrison E. The european socio-economic classification: A new social class schema for comparative European research. *Eur Soc* 2007;9(3):459-90. doi: 10.1080/14616690701336518
36. Bjelland I, Dahl AA, Haug TT, et al. The validity of the Hospital Anxiety and Depression Scale. An updated literature review. *J Psychosom Res* 2002;52(2):69-77. doi: 10.1016/s0022-3999(01)00296-3 [published Online First: 2002/02/08]
37. Sedgwick P. Relative risks versus odds ratios. *Bmj-British Medical Journal* 2014;348(feb07 2):g1407-g07. doi: ARTN g1407

10.1136/bmj.g1407

For peer review only

38. Norton EC, Miller MM, Kleinman LC. Computing Adjusted Risk Ratios and Risk Differences in Stata. *The Stata Journal: Promoting communications on statistics and Stata* 2018;13(3):492-509. doi: 10.1177/1536867x1301300304
39. Galobardes B, Shaw M, Lawlor DA, et al. Indicators of socioeconomic position (part 1). *J Epidemiol Community Health* 2006;60(1):7-12. doi: 10.1136/jech.2004.023531 [published Online First: 2005/12/20]
40. Scanlan JP. Guest Editorial. *Chance* 2013;19(2):47-51. doi: 10.1080/09332480.2006.10722787 [published Online First: 02 Aug 2013]
41. Willadsen TG, Bebe A, Koster-Rasmussen R, et al. The role of diseases, risk factors and symptoms in the definition of multimorbidity - a systematic review. *Scand J Prim Health Care* 2016;34(2):112-21. doi: 10.3109/02813432.2016.1153242 [published Online First: 2016/03/10]
42. Schuurmans H, Steverink N, Lindenberg S, et al. Old or frail: what tells us more? *J Gerontol A Biol Sci Med Sci* 2004;59(9):M962-5. doi: 10.1093/gerona/59.9.m962 [published Online First: 2004/10/09]
43. Theou O, O'Connell MD, King-Kallimanis BL, et al. Measuring frailty using self-report and test-based health measures. *Age Ageing* 2015;44(3):471-7. doi: 10.1093/ageing/afv010 [published Online First: 2015/02/18]
44. Braveman PA, Cubbin C, Egerter S, et al. Socioeconomic status in health research: one size does not fit all. *JAMA* 2005;294(22):2879-88.
45. Holmen J, Midthjell K, Krüger Ø, et al. The Nord-Trøndelag Health Study 1995–97 (HUNT 2): objectives, contents, methods and participation. *Norsk epidemiologi* 2003;13(1):19-32.
46. NCD Risk Factor Collaboration (NCD-RisC). Rising rural body-mass index is the main driver of the global obesity epidemic in adults. *Nature* 2019;569(7755):260-64. doi: 10.1038/s41586-019-1171-x [published Online First: 2019/05/10]
47. NCD Risk Factor Collaboration (NCD-RisC). Worldwide trends in blood pressure from 1975 to 2015: a pooled analysis of 1479 population-based measurement studies with 19.1 million participants. *Lancet* 2017;389(10064):37-55. doi: 10.1016/S0140-6736(16)31919-5 [published Online First: 2016/11/20]
48. NCD Risk Factor Collaboration (NCD-RisC). Worldwide trends in body-mass index, underweight, overweight, and obesity from 1975 to 2016: a pooled analysis of 2416 population-based measurement studies in 128.9 million children, adolescents, and adults. *Lancet* 2017;390(10113):2627-42. doi: 10.1016/S0140-6736(17)32129-3 [published Online First: 2017/10/17]
49. Smith SM, Soubhi H, Fortin M, et al. Managing patients with multimorbidity: systematic review of interventions in primary care and community settings. *BMJ* 2012;345:e5205. doi: 10.1136/bmj.e5205 [published Online First: 2012/09/05]
50. World Health Organization. Multimorbidity: Technical Series on Safer Primary Care. In: Organization WH, ed. Licence: CC BY-NC-SA 3.0 IGO. ed. Geneva.: World Health Organization, 2016:p. 4-5.
51. Marmot M, Goldblatt P, Allen J, et al. Fair Society, Healthy Lives.: The Marmot Review 2010.
52. Gill TM, Gahbauer EA, Allore HG, et al. Transitions between frailty states among community-living older persons. *Arch Intern Med* 2006;166(4):418-23. doi: 10.1001/archinte.166.4.418 [published Online First: 2006/03/01]

For peer review only

Fig. 1. Flowchart sample selection: inclusion and exclusion criteria and missing data.

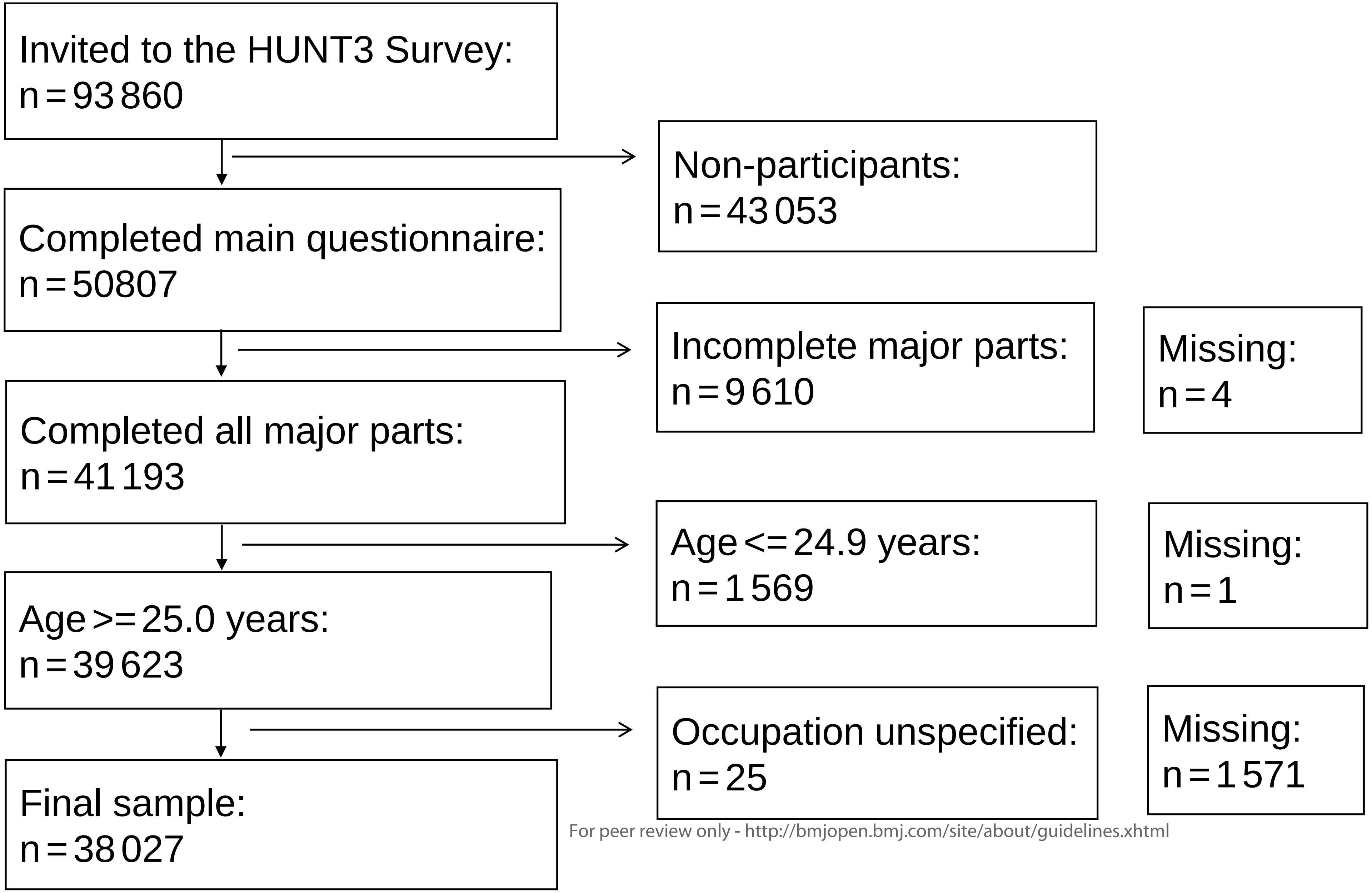

For peer review only - http://bmjopen.bmj.com/site/about/guidelines.xhtml

Appendix A
Operationalizing socioeconomic position using occupation.

In the HUNT3 Survey interview, all participants were asked: "What is/was the title of your main occupation?" Free-text answers were manually classified according to the *Standard Classifications of Occupations* by Statistics Norway,[1] which is based on the European Union's version of the *International Standard Classification of Occupations-88.*[2]

The standard categorize occupations according to skill level and specialization, degree of independence, and manual labor but not social position.[1] Occupations are coded with up to four digits, with increasing detail. One digit indicates major groups; two digits, submajor groups; three digits, minor groups; and four digits, unit groups. The minor occupational group was the highest level of detail available in the HUNT3 Survey.

Occupational socioeconomic position was operationalized using the European Socio-economic Classification scheme.[3] The full version of the scheme requires employment status and size of organization in addition to occupation to assign a class position. We used the simplified class scheme, based on minor occupational group only[3], as the HUNT3 Survey did not have data corresponding to employment status and size of organization. It is shown that the agreement between three-digit full and simplified version of this scheme is 79.7% for the total workforce.[3]

The syntax is available from https://www.iser.essex.ac.uk/archives/esec/matrices-and-syntax. It was performed using SPSS 25.0 (SPSS Inc., Chicago, IL, USA).

Table 1 gives details of transformation of data, discrepancies between the Norwegian and European Union standard and the allocated position in the full classification scheme. 2179 individuals had alterations to their occupational data to fit the syntax, 5.7% (2179/38027) of the total sample.

In the HUNT3 Survey data, the minor occupational group was a string variable. To perform the syntax, it had to be altered to a numeric variable. The string "011" changed to numeric value "11," which was manually corrected in the syntax. In the 3-digit variable, some participants were classified with 1 digit and 2 digits only. These were transformed to the corresponding 3-digit minor group, at the lowest level of detail, by manually adding suffix digits 0 or 00. This is in line with operationalizing of European Socio-economic Classification (see footnote table 1).[3]

Norwegian minor groups, which were not found in the European Union standard, were altered to the level of detail in which corresponding groups could be identified. These were *Standard Classifications of Occupations* by Statistics Norway codes: 112 (corresponding to 2 digits), 25 (corresponding to 1 digit), 251-6 (corresponding to 1 digit), 349 (corresponding to 2 digits), 631 (corresponding to 1 digit), 641 (corresponding to 1 digit), 735 (corresponding to 2 digits), and 745 (corresponding to 2 digits).

For peer review only

In total, 9 classes were created. To increase power and simplify interpretation, the full scheme was collapsed into a 3-class version, with "high" combining class 1 and 2, "middle" combining 3 to 6, and "low" combining 7 to 9. [3] The high occupational class represents large employers, higher-grade and lower-grade professionals, administrative and managerial occupations, higher-grade technician occupations, and supervisory occupations. The middle occupational class consist of small employers, self-employed individuals, lower supervisory occupations, and lower technician occupations. The low occupational class contain lower services, sales and clerical occupations, lower technical occupations, and routine occupations.

For peer review only

Table A1. The distribution of transformed occupational data and discrepancies between the Norwegian and International Standard Classifications of Occupations, and allocation in the European Socio-economic Classification scheme.

**Standard Classifications of Occupations**		**European Socio-economic**		
**Norwegian**	**International**	**Classification scheme**	***n***	**%**
1	100	1	262	(0.69)
011 (=num 11)	011=11	3	134	(0.35)
**112***	→ 11=110	1	31	(0.08)
12	120	1	73	(0.19)
13	130	4	20	(0.05)
2	200	1	10	(0.03)
21	210	1	10	(0.03)
22	220	1	1	(0.00)
23	230	2	27	(0.07)
24	240	1	9	(0.02)
25	→ 2=200	1	4	(0.01)
**251***	→ 2=200	1	296	(0.78)
**252***	→ 2=200	1	48	(0.13)
**253***	→ 2=200	1	20	(0.05)
**254***	→ 2=200	1	138	(0.36)
**255***	→ 2=200	1	64	(0.17)
**256***	→ 2=200	1	46	(0.12)
3	300	3	39	(0.10)
31	310	2	37	(0.10)
33	330	3	241	(0.63)
34	340	3	45	(0.12)
**349***	→34=340	3	160	(0.42)
4	400	3	1	(0.00)
41	410	3	1	(0.00)
42	420	3	1	(0.00)
5	500	7	1	(0.00)
51	510	7	8	(0.02)
61	610	5	4	(0.01)
**631***	→6=600	5	93	(0.24)
**641***	→6=600	5	99	(0.26)
7	700	8	20	(0.05)
71	710	8	1	(0.00)
72	720	8	6	(0.02)
73	730	6	1	(0.00)
**735***	→73=730	6	38	(0.10)
74	740	8	1	(0.00)
**745***	→74=740	8	46	(0.12)
8	800	9	62	(0.16)
81	810	9	38	(0.10)
82	820	9	35	(0.09)
83	830	9	6	(0.02)
9	900	9	1	(0.00)
93	930	9	1	(0.00)
**Sum**			2179	(5.73)

For peer review only - http://bmjopen.bmj.com/site/about/guidelines.xhtml

Bold* = Divergence of *Standard Classifications of Occupations* by Statistics Norway from the European Union's version of *The International Standard Classification of Occupations-88*.

References

1. Statistics Norway. Standard Classification of Occupations. Oslo/Kongsvinger: Statistics Norway, 1998.
2. International Labour Organization (ILO). The International Standard Classification of Occupations, ISCO-88 [Webpage]. 1988 [updated 18.09.2004. Available from: https://www.ilo.org/public/english/bureau/stat/isco/isco88/index.htm accessed 24.05. 2019.
3. Rose D, Harrison E. The european socio-economic classification: A new social class schema for comparative European research. *Eur Soc* 2007;9(3):459-90. doi: 10.1080/14616690701336518

For peer review only

Appendix B

Construction of chronic, single-entities conditions from data in the HUNT3 Survey, by questionnaires and measurements.

ORIGINAL QUESTIONNAIRE, ENGLISH VERSION

Main questionnaire

https://www.ntnu.edu/c/document_library/get_file?uuid=129b68c3-520c-457f-8b98-02c49219b2ee&groupId=140075

Sex- and age-specific questionnaire

https://www.ntnu.edu/c/document_library/get_file?uuid=35ae2816-4155-4b64-a259-770946fa46d4&groupId=140075

GENERAL COMMENTS

Chronicity

Chronicity was defined by either 1: duration (3 months or longer), 2: causing functional limitation (physical, mental, social) or 3: requiring health care management (pharmacological or not, primary or specialist care),[1] or 4: chronicity was assumed based on medical knowledge and clinical experience.

Missing

In variables with index questions and cluster text, missing was in general corrected for affirmed index question and regarded as “no” if replied to any alternative to any of the other questions in the block. Information on missing is also collected from the HUNT Databank.

MAIN QUESTIONNAIRE

Hearing impairment

Index question: “Do you suffer from longstanding (at least 1 year) illness or injury of a physical or psychological nature that impairs your functioning in your daily life?” Yes, no. Options on follow-up question combined condition type (motor, vision, hearing, somatic, and psychiatric) and severity (slight, moderate, and severe).

For peer review only

Included with hearing impairment were those who reported chronic disease and moderate to severe hearing impairment.

"20 Diseases": Myocardial infarction, angina pectoris, heart failure, other heart disease, stroke or brain haemorrhage, kidney disease, asthma, chronic bronchitis, emphysema or chronic obstructive pulmonary disease, diabetes, psoriasis, eczema on hands, cancer, epilepsy, rheumatoid arthritis, ankylosing spondylitis, sarcoidosis, osteoporosis, fibromyalgia and osteoarthritis

Cluster text: "Have you had or do you have any of the following:
Myocardial infarction, angina pectoris, heart failure, other heart disease, stroke or brain haemorrhage, kidney disease, asthma, chronic bronchitis, emphysema or chronic obstructive pulmonary disease, diabetes, psoriasis, eczema on hands, cancer, epilepsy, rheumatoid arthritis, ankylosing spondylitis, sarcoidosis, osteoporosis, fibromyalgia and osteoarthritis?"
Separate tick boxes for each diagnosis: Yes, no.
For each diagnosis, included were those who affirmed to have or have had the diagnosis.
Chronicity is assumed based on medical knowledge and clinical experience.

Sex- and age-differentiated questionnaire

Headache

Seven questions in one block. Question 1: "Have you had headaches in the last year?" Yes/no.

Migraine without aura

Of those who affirmed headache last year, migraine without aura was constructed from three of seven questions:

1. "What is the average strength of your headaches?" 1=Mild, 2=Moderate, 3=Strong. Recoded to dichotomous variable, where 1=Moderate/Strong.
2. "How long does the headache usually last?" 1=Less than 4 hours, 2=4 hours - 1 day, 3=1 - 3 days, 4= More than 3 days.
   Recoded to dichotomous variable, where 1= Less than 4 hours – 3 days.
3. Cluster text: "Are the headaches usually characterized or accompanied by
   - Throbbing/thumping pain?" Yes, no.
   - Pain on one side of the head?" Yes, no.
   - Worsening with physical activity?" Yes, no.
   - Nausea and/or vomiting?" Yes, no.
   - Hypersensitivity to light and/or noise?" Yes, no.

Included with migraine: were those who affirmed to headache lasting 0 to 72 hours and at least two of four characteristics (pulsating quality, unilateral location, moderate/severe pain intensity, or aggravation by physical activity) and during headache having at least one of two accompanying symptoms (nausea and/or vomiting or increased sensitivity to light and/or noise).[2]
Chronicity is assumed based on medical knowledge and clinical experience.

Chronic headache

Of those who affirmed headache last year, chronic headache was constructed from two of seven questions:

1. "If yes (headache in the last year): What type of headache? Migraine, other."
   The HUNT Databank created two variables with range 1: 1) migraine and 2) other headache.
2. "Average number of days a month with headaches:"
   1=Less than 1 day, 2=1-6 days, 3=7-14 days, 4=More than 14 days.
   Recoded to dichotomous variable, where 1= More than 14 days.

Included as case with chronic headache were those reporting "other" type of headache and an average frequency of more than 14 days per month.
Chronicity is assumed based on medical knowledge and clinical experience.

Pain

Index question: "In the last year, have you had pain or stiffness in muscles or joints that has lasted at least 3 consecutive months?" Yes, no.
The follow-up question "If yes: Where have you had this pain or stiffness?" was combined with a figure with arrows and tick boxes at nine locations (neck, upper back, lower back, shoulder, elbow, hand, hip, knee and ankle/foot).

Chronic widespread pain

Dichotomous variables were made for each major body area: 1) Trunk (neck, upper and lower back),
2) Upper limb (shoulder, elbow, hand), and 3) Lower limb (hip, knee, foot/ancle), where 1=At least one painful location. A sum (row total) score variable was made for the major body areas and dichotomized, where 1=3, that is one pain in each major body area.
Of those who affirmed to pain or stiffness that has lasted more than three consecutive months, chronic widespread pain was defined as pain at more than three sites in all major body areas (trunk, upper and lower limbs) for more than three months in the last year.[3]

Chronic, local pain

Of those who affirmed to pain or stiffness that has lasted more than three consecutive months,
chronic, local pain was defined as pain in the neck or upper back or lower back or shoulder or elbow or hand or hip or knee or ancle/foot, excluding presence of chronic widespread pain, generating nine dichotomous variables.

Thyroidal disease

Cluster text: "Has it ever been verified that you have/have had hypothyroidism or hyperthyroidism?" Separate tick boxes for each condition (yes, no), generating two dichotomous variables, 1=Yes.
For each diagnosis, included were those who affirmed to have or have had the diagnosis.
Chronicity is assumed based on medical knowledge and clinical experience.

For peer review only

Irritable bowel syndrome

Index question: “Have you had stomach pain or discomfort in the last 12 months?” Answers: Yes, much; yes, a little; no. Irritable bowel syndrome was further constructed from four of six follow-up questions: “If yes:
“In the last 3 months, have you had this as often as 1 day a week for at least 3 weeks?” Yes, no.
“Is the pain/discomfort relieved by having a bowel movement?” Yes, no.
“Is the pain/discomfort related to more frequent or less frequent bowel movements than normal?” Yes,no.
“Is the pain/discomfort related to the stool being softer or harder than usual?” Yes, no.

Included with irritable bowel syndrome were those who affirmed little or much stomach pain or discomfort in the last year, who for as often as 1 day a week for at least 3 weeks in the last 3 months have had at least two of the following: pain/discomfort relieved by having a bowel movement, related to altered frequency of bowel movements, or related to altered stool appearance, resembling a modified version of the Rome criteria.[4 5]

Gastro-oesophageal reflux disease

Cluster text: “To what degree have you had the following problems in the last 12 months?” Options combined type (nausea, heartburn/acid regurgitation, diarrhea, constipation, alternating constipation and diarrhea, and bloating) and frequency (never, a little, or much). Generated one dichotomous variable, heartburn, where 1=Much.
Gastro-oesophageal reflux disease is defined as much heartburn/acid regurgitation in the last 12 months.[6]

Anxiety

Instrument variable: Hospital Anxiety and Depression Scale.[7] Every other statement of 14 statements covers symptoms on anxiety and depression and is scored 0-3. The HUNT Databank constructed a total score for anxiety (HADS-A), if all 7 anxiety items were answered.
Anxiety was defined as HADS-A score >=8/21, indicating mild or possible anxiety.[8-10]
Chronicity is assumed based on medical knowledge and clinical experience.

Depression

Instrument variable: Hospital Anxiety and Depression Scale.[7] Every other statement of 14 statements covers symptoms on anxiety and depression and is scored 0-3. The HUNT Databank constructed total score depression (HADS-D), if all 7 depression items were answered.
Depression was defined as HADS-D score >=8/21, indicating mild or possible depression.[8-10]
Chronicity is assumed based on medical knowledge and clinical experience.

Chronic insomnia

There were nine questions on sleeping pattern in one cluster, including three concerning insomnia. Initial text: “How often in the last 3 months have you
“Had difficulty falling asleep at night?” Never/seldom, sometimes, several times a week.
“Woken up repeatedly during the night?” Never/seldom, sometimes, several times a week.

For peer review only

"Woken too early and couldn't get back to sleep?" Never/seldom, sometimes, several times a week.
Chronic insomnia was defined as in the last 3 months, several times a week, having difficulty falling asleep at night and waking up repeatedly during the night, and waking up too early. A modified version of the diagnostic criteria for insomnia in the International Classification of Sleep Disorders.[11]

Alcohol use disorder

Instrument variable: Cut down/Annoyed/Guilty/Eye-opener, also known as the CAGE questionnaire.12 The CAGE questionnaire is a 4-item scale with scores of 0-1. A summary variable was created and dichotomized in which a score of 1 indicates >=2 positive answers. Alcohol use disorder was defined as CAGE score greater than 2.[13]
Chronicity is assumed based on medical knowledge and clinical experience.

Dental health problem

"How would you say your dental health is?" Very, bad, ok, good, very good.
Dental health problems were defined as self-reported bad or very bad dental health.
Chronicity is assumed based on medical knowledge and clinical experience.

Menopausal hot flashes

Asked to women older than 30 years only.
Two questions were used to define menopausal illness:
"Do you have/have you had hot flashes due to menopause?" During the day, during the night, day and night, haven't had any.
"If you have had hot flashes, how would you describe them?" Very intense, moderately intense, hardly noticeable.
Included with menopausal hot flashes were those who reported hot flashes occurring daily and/or nightly and of at least moderate severity.
Chronicity is assumed based on medical knowledge and clinical experience.

Nocturia

Age group 20-29 years were excluded.
One question on nocturia, identical to that of the International Prostate Symptom Scale (IPSS), was asked to men and women older than 30 years.
"How many times do you get up during the night to urinate?" None, 1 time, 2 times, 3 times, 4 times, 5 times or more.
Nocturia was defined as two or more voids per night.[14]
Chronicity is assumed based on medical knowledge and clinical experience.

Urine incontinence

Men 20-29 years were excluded.
Instrument variable: The Epidemiology of Incontinence in the County of Nord-Trøndelag (EPINCONT) questionnaire.[15]
Index question: Do you have involuntary loss of urine? Yes, no.
Urine incontinence was constructed from two of six follow up questions. "If yes":

For peer review only

“How often do you have involuntary loss of urine?” Less than once a month, once or more per month, once or more per week, every day and/or night
“How much urine do you leak each time?” Drops or little, small amount, large amounts.

Self-reported frequency and volume of leakage were multiplied to obtain the validated 4-level Sandvik Severity Index, categorizing incontinence as slight, moderate, severe, and very severe.[15]
Urine incontinence were included if severe to very severe.
Chronicity is assumed based on medical knowledge and clinical experience.

Prostate symptoms

Asked of men older than 30 years only.
Instrument variable: The International Prostate Symptom Scale [16] was slightly modified in HUNT3,[17] becoming a 7-item scale with scores of 0-5 per question.
Included were prostate symptoms of at least moderate severity, i.e. summary score >= 8 points.[16]
Chronicity is assumed based on medical knowledge and clinical experience.

Eye diseases

The age group 20-29 years were excluded.
Cluster text: “Do you have any of the following eye conditions?” Cataract, glaucoma, and macula degeneration. Separate tick boxes, yes, no.
For each diagnosis, included were those who affirmed to have or have had the diagnosis.

Measurements

Obesity

HUNT Databank constructed the BMI variable, defined as (weight in kg)/(height in m2).
Obesity was defined as either BMI>=35 or a BMI 25-34.9 and an increased waist circumference (>= 88 cm for females; >= 102 cm for males).18 19
Chronicity is assumed based on medical knowledge and clinical experience.

Hypertension

Blood pressure in HUNT3 is measured three times at one consultation. The mean of measurement 2 and 3 is calculated by HUNT Databank.
Hypertension was defined as measured mean systolic BP>= 180 mmHg or diastolic BP >= 110 mmHg or reporting use of antihypertensive medications, excluding self-reported cardiovascular disease, diabetes, or kidney disease, and excluding extreme measures.
Chronicity is assumed based on medical knowledge and clinical experience.

Hypercholesterolemia

Hypercholesterolemia was defined as total-cholesterol >= 8 mmol/L.[20]
Chronicity is assumed based on medical knowledge and clinical experience.

For peer review only

References

1. Goodman RA, Posner SF, Huang ES, et al. Defining and measuring chronic conditions: imperatives for research, policy, program, and practice. *Prev Chronic Dis* 2013;10:E66. doi: 10.5888/pcd10.120239 [published Online First: 2013/04/27]
2. Hagen K, Zwart JA, Aamodt AH, et al. The validity of questionnaire-based diagnoses: the third Nord-Trondelag Health Study 2006-2008. *J Headache Pain* 2010;11(1):67-73. doi: 10.1007/s10194-009-0174-7 [published Online First: 2009/12/01]
3. Mundal I, Grawe RW, Bjorngaard JH, et al. Prevalence and long-term predictors of persistent chronic widespread pain in the general population in an 11-year prospective study: the HUNT study. *BMC Musculoskelet Disord* 2014;15:213. doi: 10.1186/1471-2474-15-213 [published Online First: 2014/06/22]
4. Hammer J, Talley NJ. Diagnostic criteria for the irritable bowel syndrome. *Am J Med* 1999;107(5A):5S-11S. doi: 10.1016/s0002-9343(99)00276-4 [published Online First: 1999/12/10]
5. Longstreth GF, Thompson WG, Chey WD, et al. Functional bowel disorders. *Gastroenterology* 2006;130(5):1480-91. doi: 10.1053/j.gastro.2005.11.061 [published Online First: 2006/05/09]
6. Ness-Jensen E, Lindam A, Lagergren J, et al. Changes in prevalence, incidence and spontaneous loss of gastro-oesophageal reflux symptoms: a prospective population-based cohort study, the HUNT study. *Gut* 2012;61(10):1390-7. doi: 10.1136/gutjnl-2011-300715 [published Online First: 2011/12/23]
7. Zigmond AS, Snaith RP. The hospital anxiety and depression scale. *Acta Psychiatr Scand* 1983;67(6):361-70. doi: 10.1111/j.1600-0447.1983.tb09716.x [published Online First: 1983/06/01]
8. Mykletun A, Stordal E, Dahl AA. Hospital Anxiety and Depression (HAD) scale: factor structure, item analyses and internal consistency in a large population. *Br J Psychiatry* 2001;179:540-4. doi: 10.1192/bjp.179.6.540 [published Online First: 2001/12/04]
9. Bjelland I, Dahl AA, Haug TT, et al. The validity of the Hospital Anxiety and Depression Scale. An updated literature review. *J Psychosom Res* 2002;52(2):69-77. doi: 10.1016/s0022-3999(01)00296-3 [published Online First: 2002/02/08]
10. Herrmann C. International experiences with the Hospital Anxiety and Depression Scale--a review of validation data and clinical results. *J Psychosom Res* 1997;42(1):17-41. [published Online First: 1997/01/01]
11. Medicine AAoS. The international classification of sleep disorders: diagnostic and coding manual: American Acad. of Sleep Medicine 2005.
12. Ewing JA. Detecting alcoholism. The CAGE questionnaire. *JAMA* 1984;252(14):1905-7. doi: 10.1001/jama.252.14.1905 [published Online First: 1984/10/12]
13. Skogen JC, Overland S, Knudsen AK, et al. Concurrent validity of the CAGE questionnaire. The Nord-Trondelag Health Study. *Addict Behav* 2011;36(4):302-7. doi: 10.1016/j.addbeh.2010.11.010 [published Online First: 2010/12/21]
14. Tikkinen KA, Johnson TM, 2nd, Tammela TL, et al. Nocturia frequency, bother, and quality of life: how often is too often? A population-based study in Finland. *Eur Urol* 2010;57(3):488-96. doi: 10.1016/j.eururo.2009.03.080 [published Online First: 2009/04/14]
15. Sandvik H, Seim A, Vanvik A, et al. A severity index for epidemiological surveys of female urinary incontinence: comparison with 48-hour pad-weighing tests. *Neurourol Urodyn* 2000;19(2):137-45. [published Online First: 2000/02/19]
16. Barry MJ, Fowler FJ, Jr., O'Leary MP, et al. The American Urological Association symptom index for benign prostatic hyperplasia. The Measurement Committee of the American Urological Association. *J Urol* 1992;148(5):1549-57; discussion 64. doi: 10.1016/s0022-5347(17)36966-5 [published Online First: 1992/11/11]
17. HUNT Databank. International Prostate Symptom Scale in HUNT3 Questionnaire 2 [Webpage]. Levanger: HUNT Databank; 2019 [cited 2019 05.20.]. Available from: https://hunt-db.medisin.ntnu.no/hunt-db/#/instrumentpart/45_11 2019.
18. Janssen I, Katzmarzyk PT, Ross R. Waist circumference and not body mass index explains obesity-related health risk. *Am J Clin Nutr* 2004;79(3):379-84. doi: 10.1093/ajcn/79.3.379 [published Online First: 2004/02/27]
19. Perreault L. Obesity in adults: Prevalence, screening, and evaluation. Waltham, MA: UpToDate 2018.
20. Helsedirektoratet. Nasjonal faglig retningslinje for individuell primærforebygging av hjerte-og karsykdommer, kortversjon, IS-1675. In: Helsedirektoratet, ed., 2009.

Appendix C

Table C1. Prevalence ratios (PR) and prevalence differences (PD) with 95% confidence intervals (CI) for the association between occupational group and multimorbidity with frailty, stratified by sex, age 25 to 100 years in 5-year intervals.

*Occup. = occupational.

For peer review only

Two conditions of multimorbidity and one dimension of frailty

Age, years	Occup.* group	Female PR	95% CI	PD	95% CI	Men PR	95% CI	PD	95% CI
25	High	1.0	(Ref.)	0.0	(Ref.)	1.0	(Ref.)	0.0	(Ref.)
	Middle	1.34	(1.01, 1.79)	0.05	(0.00, 0.09)	0.81	(0.55, 1.20)	-0.03	(-0.08, 0.03)
	Low	2.20	(1.73, 2.81)	0.17	(0.12, 0.21)	1.19	(0.86, 1.65)	0.03	(-0.02, 0.08)
30	High	1.0	(Ref.)	0.0	(Ref.)	1.0	(Ref.)	0.0	(Ref.)
	Middle	1.36	(1.11, 1.65)	0.06	(0.02, 0.09)	0.93	(0.70, 1.23)	-0.01	(-0.06, 0.03)
	Low	2.09	(1.76, 2.47)	0.17	(0.14, 0.20)	1.32	(1.04, 1.67)	0.05	(0.01, 0.09)
35	High	1.0	(Ref.)	0.0	(Ref.)	1.0	(Ref.)	0.0	(Ref.)
	Middle	1.36	(1.19, 1.55)	0.06	(0.04, 0.09)	1.04	(0.85, 1.27)	0.01	(-0.03, 0.04)
	Low	1.97	(1.75, 2.20)	0.17	(0.15, 0.20)	1.43	(1.22, 1.68)	0.07	(0.04, 0.10)
40	High	1.0	(Ref.)	0.0	(Ref.)	1.0	(Ref.)	0.0	(Ref.)
	Middle	1.34	(1.22, 1.47)	0.07	(0.05, 0.09)	1.14	(0.99, 1.31)	0.03	(0.00, 0.05)
	Low	1.84	(1.70, 2.00)	0.17	(0.15, 0.19)	1.52	(1.35, 1.70)	0.09	(0.07, 0.12)
45	High	1.0	(Ref.)	0.0	(Ref.)	1.0	(Ref.)	0.0	(Ref.)
	Middle	1.31	(1.21, 1.42)	0.07	(0.05, 0.09)	1.23	(1.11, 1.36)	0.04	(0.02, 0.07)
	Low	1.72	(1.60, 1.84)	0.17	(0.15, 0.19)	1.58	(1.44, 1.72)	0.11	(0.09, 0.13)
50	High	1.0	(Ref.)	0.0	(Ref.)	1.0	(Ref.)	0.0	(Ref.)
	Middle	1.27	(1.17, 1.37)	0.07	(0.05, 0.10)	1.29	(1.18, 1.41)	0.06	(0.04, 0.09)
	Low	1.59	(1.49, 1.70)	0.16	(0.14, 0.18)	1.60	(1.48, 1.73)	0.13	(0.11, 0.15)
55	High	1.0	(Ref.)	0.0	(Ref.)	1.0	(Ref.)	0.0	(Ref.)
	Middle	1.22	(1.13, 1.31)	0.07	(0.04, 0.09)	1.34	(1.23, 1.45)	0.08	(0.06, 0.11)
	Low	1.48	(1.38, 1.58)	0.15	(0.13, 0.17)	1.60	(1.48, 1.72)	0.15	(0.13, 0.17)
60	High	1.0	(Ref.)	0.0	(Ref.)	1.0	(Ref.)	0.0	(Ref.)
	Middle	1.16	(1.08, 1.25)	0.06	(0.03, 0.09)	1.35	(1.25, 1.46)	0.10	(0.08, 0.13)
	Low	1.37	(1.29, 1.46)	0.13	(0.11, 0.16)	1.56	(1.46, 1.68)	0.16	(0.14, 0.18)
65	High	1.0	(Ref.)	0.0	(Ref.)	1.0	(Ref.)	0.0	(Ref.)
	Middle	1.11	(1.03, 1.19)	0.04	(0.02, 0.07)	1.35	(1.26, 1.45)	0.11	(0.09, 0.14)
	Low	1.27	(1.20, 1.35)	0.11	(0.09, 0.14)	1.51	(1.41, 1.61)	0.17	(0.14, 0.19)
70	High	1.0	(Ref.)	0.0	(Ref.)	1.0	(Ref.)	0.0	(Ref.)
	Middle	1.05	(0.98, 1.14)	0.03	(-0.01, 0.06)	1.32	(1.24, 1.42)	0.12	(0.09, 0.15)
	Low	1.19	(1.11, 1.27)	0.09	(0.06, 0.12)	1.43	(1.35, 1.53)	0.16	(0.14, 0.19)
75	High	1.0	(Ref.)	0.0	(Ref.)	1.0	(Ref.)	0.0	(Ref.)
	Middle	1.01	(0.92, 1.10)	0.00	(-0.05, 0.05)	1.28	(1.19, 1.38)	0.12	(0.09, 0.16)
	Low	1.11	(1.03, 1.21)	0.06	(0.02, 0.10)	1.35	(1.25, 1.45)	0.15	(0.12, 0.19)
80	High	1.0	(Ref.)	0.0	(Ref.)	1.0	(Ref.)	0.0	(Ref.)
	Middle	0.96	(0.86, 1.08)	-0.02	(-0.09, 0.05)	1.23	(1.12, 1.35)	0.12	(0.06, 0.17)
	Low	1.05	(0.95, 1.16)	0.03	(-0.03, 0.09)	1.27	(1.15, 1.39)	0.14	(0.09, 0.18)
85	High	1.0	(Ref.)	0.0	(Ref.)	1.0	(Ref.)	0.0	(Ref.)
	Middle	0.93	(0.81, 1.06)	-0.05	(-0.14, 0.04)	1.17	(1.04, 1.32)	0.10	(0.03, 0.17)
	Low	1.00	(0.89, 1.13)	0.00	(-0.08, 0.08)	1.19	(1.06, 1.33)	0.11	(0.04, 0.18)
90	High	1.0	(Ref.)	0.0	(Ref.)	1.0	(Ref.)	0.0	(Ref.)
	Middle	0.90	(0.77, 1.05)	-0.07	(-0.18, 0.04)	1.12	(0.98, 1.29)	0.08	(-0.01, 0.17)
	Low	0.96	(0.85, 1.10)	-0.03	(-0.12, 0.07)	1.12	(0.98, 1.27)	0.08	(-0.01, 0.16)
95	High	1.0	(Ref.)	0.0	(Ref.)	1.0	(Ref.)	0.0	(Ref.)
	Middle	0.88	(0.74, 1.05)	-0.09	(-0.22, 0.03)	1.08	(0.93, 1.24)	0.06	(-0.05, 0.16)
	Low	0.94	(0.82, 1.08)	-0.05	(-0.15, 0.06)	1.06	(0.93, 1.22)	0.05	(-0.06, 0.15)
100	High	1.0	(Ref.)	0.0	(Ref.)	1.0	(Ref.)	0.0	(Ref.)
	Middle	0.86	(0.72, 1.04)	-0.11	(-0.25, 0.03)	1.04	(0.90, 1.20)	0.03	(-0.08, 0.15)
	Low	0.92	(0.80, 1.06)	-0.07	(-0.18, 0.05)	1.02	(0.89, 1.17)	0.02	(-0.09, 0.13)

For peer review only

Three conditions of multimorbidity and two dimensions of frailty

Age,	Occup.*	Female				Men			
years	group	PR	95% CI	PD	95% CI	PR	95% CI	PD	95% CI
25	High	1.0	(Ref.)	0.0	(Ref.)	1.0	(Ref.)	0.0	(Ref.)
	Middle	2.74	(1.60, 4.71)	0.04	(0.02, 0.06)	1.15	(0.57, 2.32)	0.01	(-0.02, 0.03)
	Low	4.24	(2.61, 6.89)	0.07	(0.05, 0.10)	1.36	(0.74, 2.51)	0.01	(-0.01, 0.04)
30	High	1.0	(Ref.)	0.0	(Ref.)	1.0	(Ref.)	0.0	(Ref.)
	Middle	2.31	(1.56, 3.40)	0.04	(0.02, 0.06)	1.29	(0.77, 2.17)	0.01	(-0.01, 0.03)
	Low	3.59	(2.53, 5.08)	0.08	(0.06, 0.10)	1.60	(1.02, 2.51)	0.02	(0.00, 0.04)
35	High	1.0	(Ref.)	0.0	(Ref.)	1.0	(Ref.)	0.0	(Ref.)
	Middle	1.98	(1.51, 2.59)	0.04	(0.03, 0.06)	1.41	(0.97, 2.05)	0.02	(0.00, 0.04)
	Low	3.06	(2.41, 3.90)	0.09	(0.07, 0.11)	1.81	(1.31, 2.50)	0.04	(0.02, 0.05)
40	High	1.0	(Ref.)	0.0	(Ref.)	1.0	(Ref.)	0.0	(Ref.)
	Middle	1.73	(1.43, 2.09)	0.04	(0.03, 0.06)	1.51	(1.16, 1.96)	0.03	(0.01, 0.04)
	Low	2.63	(2.23, 3.11)	0.10	(0.08, 0.11)	1.97	(1.57, 2.47)	0.05	(0.04, 0.07)
45	High	1.0	(Ref.)	0.0	(Ref.)	1.0	(Ref.)	0.0	(Ref.)
	Middle	1.55	(1.33, 1.79)	0.04	(0.03, 0.06)	1.58	(1.30, 1.91)	0.04	(0.02, 0.05)
	Low	2.29	(2.01, 2.60)	0.10	(0.09, 0.11)	2.07	(1.75, 2.44)	0.07	(0.05, 0.08)
50	High	1.0	(Ref.)	0.0	(Ref.)	1.0	(Ref.)	0.0	(Ref.)
	Middle	1.41	(1.23, 1.61)	0.04	(0.02, 0.06)	1.62	(1.38, 1.89)	0.05	(0.03, 0.06)
	Low	2.01	(1.78, 2.26)	0.10	(0.09, 0.11)	2.09	(1.82, 2.40)	0.08	(0.07, 0.09)
55	High	1.0	(Ref.)	0.0	(Ref.)	1.0	(Ref.)	0.0	(Ref.)
	Middle	1.31	(1.14, 1.50)	0.04	(0.02, 0.06)	1.62	(1.40, 1.87)	0.06	(0.04, 0.07)
	Low	1.78	(1.59, 2.00)	0.10	(0.08, 0.11)	2.05	(1.80, 2.33)	0.09	(0.08, 0.11)
60	High	1.0	(Ref.)	0.0	(Ref.)	1.0	(Ref.)	0.0	(Ref.)
	Middle	1.24	(1.09, 1.41)	0.04	(0.01, 0.06)	1.59	(1.39, 1.83)	0.07	(0.05, 0.08)
	Low	1.60	(1.43, 1.79)	0.09	(0.07, 0.11)	1.94	(1.71, 2.20)	0.10	(0.09, 0.12)
65	High	1.0	(Ref.)	0.0	(Ref.)	1.0	(Ref.)	0.0	(Ref.)
	Middle	1.19	(1.05, 1.35)	0.03	(0.01, 0.06)	1.54	(1.35, 1.75)	0.07	(0.05, 0.09)
	Low	1.45	(1.30, 1.62)	0.08	(0.06, 0.10)	1.79	(1.59, 2.01)	0.11	(0.09, 0.13)
70	High	1.0	(Ref.)	0.0	(Ref.)	1.0	(Ref.)	0.0	(Ref.)
	Middle	1.17	(1.02, 1.34)	0.04	(0.01, 0.06)	1.46	(1.29, 1.65)	0.08	(0.05, 0.10)
	Low	1.33	(1.18, 1.50)	0.07	(0.04, 0.10)	1.61	(1.44, 1.80)	0.10	(0.08, 0.12)
75	High	1.0	(Ref.)	0.0	(Ref.)	1.0	(Ref.)	0.0	(Ref.)
	Middle	1.16	(0.98, 1.37)	0.04	(0.00, 0.08)	1.36	(1.19, 1.56)	0.07	(0.04, 0.11)
	Low	1.23	(1.06, 1.44)	0.06	(0.02, 0.09)	1.41	(1.25, 1.60)	0.09	(0.06, 0.11)
80	High	1.0	(Ref.)	0.0	(Ref.)	1.0	(Ref.)	0.0	(Ref.)
	Middle	1.17	(0.94, 1.47)	0.05	(-0.02, 0.11)	1.26	(1.06, 1.50)	0.07	(0.02, 0.11)
	Low	1.16	(0.94, 1.42)	0.04	(-0.01, 0.10)	1.22	(1.04, 1.44)	0.06	(0.01, 0.10)
85	High	1.0	(Ref.)	0.0	(Ref.)	1.0	(Ref.)	0.0	(Ref.)
	Middle	1.19	(0.88, 1.61)	0.06	(-0.04, 0.15)	1.16	(0.92, 1.46)	0.05	(-0.03, 0.13)
	Low	1.09	(0.83, 1.44)	0.03	(-0.05, 0.11)	1.05	(0.83, 1.31)	0.01	(-0.06, 0.09)
90	High	1.0	(Ref.)	0.0	(Ref.)	1.0	(Ref.)	0.0	(Ref.)
	Middle	1.23	(0.83, 1.82)	0.07	(-0.06, 0.21)	1.06	(0.79, 1.43)	0.02	(-0.09, 0.14)
	Low	1.04	(0.72, 1.50)	0.01	(-0.10, 0.13)	0.89	(0.66, 1.19)	-0.04	(-0.15, 0.07)
95	High	1.0	(Ref.)	0.0	(Ref.)	1.0	(Ref.)	0.0	(Ref.)
	Middle	1.28	(0.77, 2.10)	0.09	(-0.09, 0.27)	0.97	(0.68, 1.40)	-0.01	(-0.18, 0.15)
	Low	1.00	(0.63, 1.59)	0.00	(-0.16, 0.16)	0.76	(0.53, 1.09)	-0.11	(-0.27, 0.04)
100	High	1.0	(Ref.)	0.0	(Ref.)	1.0	(Ref.)	0.0	(Ref.)
	Middle	1.34	(0.72, 2.47)	0.12	(-0.12, 0.35)	0.90	(0.60, 1.36)	-0.05	(-0.27, 0.16)
	Low	0.96	(0.54, 1.73)	-0.01	(-0.22, 0.19)	0.65	(0.42, 0.99)	-0.19	(-0.39, 0.01)

For peer review only

For peer review only - http://bmjopen.bmj.com/site/about/guidelines.xhtml

Reporting checklist for cross sectional study.

Based on the STROBE cross sectional guidelines.

Instructions to authors

Complete this checklist by entering the page numbers from your manuscript where readers will find each of the items listed below.

Your article may not currently address all the items on the checklist. Please modify your text to include the missing information. If you are certain that an item does not apply, please write "n/a" and provide a short explanation.

Upload your completed checklist as an extra file when you submit to a journal.

In your methods section, say that you used the STROBE cross sectionalreporting guidelines, and cite them as:

von Elm E, Altman DG, Egger M, Pocock SJ, Gotzsche PC, Vandenbroucke JP. The Strengthening the Reporting of Observational Studies in Epidemiology (STROBE) Statement: guidelines for reporting observational studies.

		Reporting Item	Page Number
**Title and abstract**			
Title	#1a	Indicate the study's design with a commonly used term in the title or the abstract	1
Abstract	#1b	Provide in the abstract an informative and balanced summary of what was done and what was found	2
**Introduction**			
Background / rationale	#2	Explain the scientific background and rationale for the investigation being reported	3
Objectives	#3	State specific objectives, including any prespecified hypotheses	3
**Methods**			

For peer review only

Study design	#4	Present key elements of study design early in the paper	3-4
Setting	#5	Describe the setting, locations, and relevant dates, including periods of recruitment, exposure, follow-up, and data collection	3-4
Eligibility criteria	#6a	Give the eligibility criteria, and the sources and methods of selection of participants.	3-4
	#7	Clearly define all outcomes, exposures, predictors, potential confounders, and effect modifiers. Give diagnostic criteria, if applicable	4
Data sources / measurement	#8	For each variable of interest give sources of data and details of methods of assessment (measurement). Describe comparability of assessment methods if there is more than one group. Give information separately for for exposed and unexposed groups if applicable.	4 + appendix B
Bias	#9	Describe any efforts to address potential sources of bias	5
Study size	#10	Explain how the study size was arrived at	NA, data collected a priori, informal assesment
Quantitative variables	#11	Explain how quantitative variables were handled in the analyses. If applicable, describe which groupings were chosen, and why	5
Statistical methods	#12a	Describe all statistical methods, including those used to control for confounding	5
Statistical methods	#12b	Describe any methods used to examine subgroups and interactions	5
Statistical methods	#12c	Explain how missing data were addressed	5
Statistical methods	#12d	If applicable, describe analytical methods taking account of sampling strategy	N/A

For peer review only

Statistical methods	#12e	Describe any sensitivity analyses	N/A

Results

Participants	#13a	Report numbers of individuals at each stage of study—eg numbers potentially eligible, examined for eligibility, confirmed eligible, included in the study, completing follow-up, and analysed. Give information separately for for exposed and unexposed groups if applicable.	3-5, fig. 1
Participants	#13b	Give reasons for non-participation at each stage	Fig. 1
Participants	#13c	Consider use of a flow diagram	Fig. 1
Descriptive data	#14a	Give characteristics of study participants (eg demographic, clinical, social) and information on exposures and potential confounders. Give information separately for exposed and unexposed groups if applicable.	5-6
Descriptive data	#14b	Indicate number of participants with missing data for each variable of interest	6, Tab. 2
Outcome data	#15	Report numbers of outcome events or summary measures. Give information separately for exposed and unexposed groups if applicable.	4
Main results	#16a	Give unadjusted estimates and, if applicable, confounder-adjusted estimates and their precision (eg, 95% confidence interval). Make clear which confounders were adjusted for and why they were included	We only gave adjusted estimates, p.6
Main results	#16b	Report category boundaries when continuous variables were categorized	6
Main results	#16c	If relevant, consider translating estimates of relative risk into absolute risk for a meaningful time period	N/A, we used postestimation commands to obtain ratios and differences

For peer review only

Other analyses	#17	Report other analyses done—e.g., analyses of subgroups and interactions, and sensitivity analyses	5, Appendix c
**Discussion**			
Key results	#18	Summarise key results with reference to study objectives	8
Limitations	#19	Discuss limitations of the study, taking into account sources of potential bias or imprecision. Discuss both direction and magnitude of any potential bias.	9
Interpretation	#20	Give a cautious overall interpretation considering objectives, limitations, multiplicity of analyses, results from similar studies, and other relevant evidence.	9
Generalisability	#21	Discuss the generalisability (external validity) of the study results	9
**Other Information**			
Funding	#22	Give the source of funding and the role of the funders for the present study and, if applicable, for the original study on which the present article is based	10

None The STROBE checklist is distributed under the terms of the Creative Commons Attribution License CC-BY. This checklist can be completed online using https://www.goodreports.org/, a tool made by the EQUATOR Network in collaboration with Penelope.ai

For peer review only
